# Supplementary material for: Defining hypoxia in cancer: A landmark evaluation of hypoxia gene expression signatures
Source: Cell Genom. 2025 Jan 31;5(2):100764. doi: 10.1016/j.xgen.2025.100764 (PMC11872601; doi:10.1016/j.xgen.2025.100764)
Supplement: Document S1. Figures S1–S25 [file mmc1.pdf]

Cell Genomics, Volume *5*

Supplemental information

Defining hypoxia in cancer: A landmark evaluation of hypoxia gene expression signatures

Matteo Di Giovannantonio, Fiona Hartley, Badran Elshenawy, Alessandro Barberis, Dan Hudson, Hana S. Shafique, Vincent E.S. Allott, David A. Harris, Simon R. Lord, Syed Haider, Adrian L. Harris, Francesca M. Buffa, and Benjamin H.L. Harris

Supplemental information

Defining hypoxia in cancer: a landmark evaluation of hypoxia gene expression signatures

Matteo Di Giovannantonio, Fiona Hartley, Badran Elshenawy, Alessandro Barberis, Dan Hudson, Hana Shafique, Vincent E.S Allott, David A. Harris, Simon R. Lord, Syed Haider, Adrian L. Harris, Francesca M. Buffa, Benjamin H.L Harris

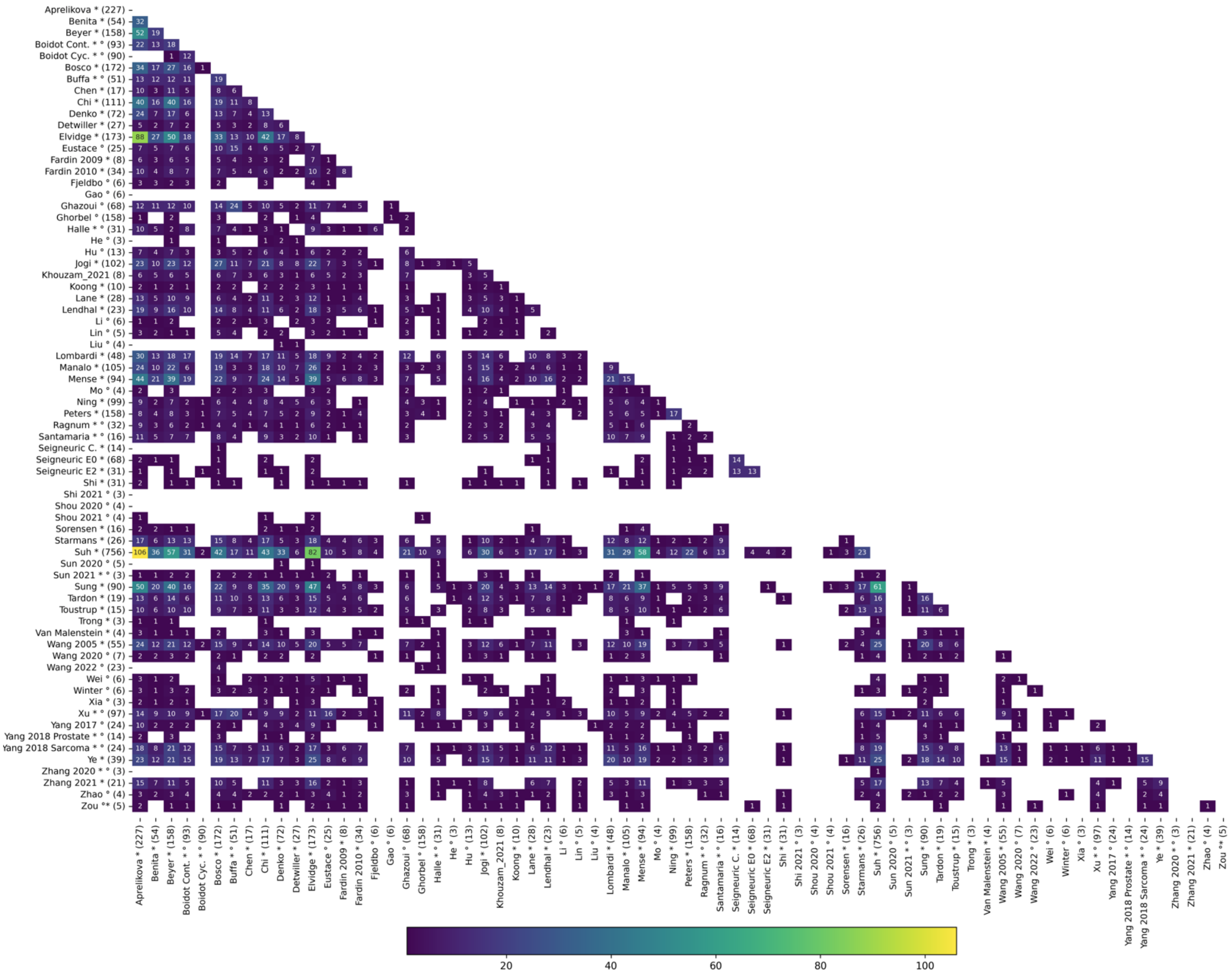

Aprelikova * (227)
Benita * (54)
Beyer * (158)
Boidot Cont. * ° (93)
Boidot Cyc. * ° (90)
Bosco * (172)
Buffa * ° (51)
Chen * (17)
Chi * (111)
Denko * (72)
Detwiller * (27)
Elvidge * (173)
Eustace ° (25)
Fardin 2009 * (8)
Fardin 2010 * (34)
Fjeldbo ° (6)
Gao ° (6)
Ghazoui ° (68)
Ghorbel ° (158)
Halle * ° (31)
He ° (3)
Hu ° (13)
Jogi * (102)
Khouzam_2021 (8)
Koong * (10)
Lane * (28)
Lendhal * (23)
Li ° (6)
Lin ° (5)
Liu ° (4)
Lombardi * (48)
Manalo * (105)
Mense * (94)
Mo ° (4)
Ning * (99)
Peters * (158)
Ragnum * ° (32)
Santamaria * ° (16)
Seigneuric C. * (14)
Seigneuric E0 * (68)
Seigneuric E2 * (31)
Shi * (31)
Shi 2021 ° (3)
Shou 2020 ° (4)
Shou 2021 ° (4)
Sorensen * (16)
Starmans * (26)
Suh * (756)
Sun 2020 ° (5)
Sun 2021 * ° (3)
Sung * (90)
Tardon * (19)
Toustrup * (15)
Trong * (3)
Van Malenstein * (4)
Wang 2005 * (55)
Wang 2020 ° (7)
Wang 2022 ° (23)
Wei ° (6)
Winter ° (6)
Xia ° (3)
Xu * ° (97)
Yang 2017 ° (24)
Yang 2018 Prostate * ° (14)
Yang 2018 Sarcoma * ° (24)
Ye * (39)
Zhang 2020 * ° (3)
Zhang 2021 * (21)
Zhao ° (4)
Zou °* (5)
20
40
60
80
100

Supplementary Figure S1: The number of overlapping genes between signatures

Heatmap of gene overlap across investigated signatures. The colour intensity indicates the number of shared genes between each pair of signatures. Signatures with no overlapping genes are represented in white. The numbers in parentheses following each signature name report the final gene count for that signature after quality check, related to Figure 1.

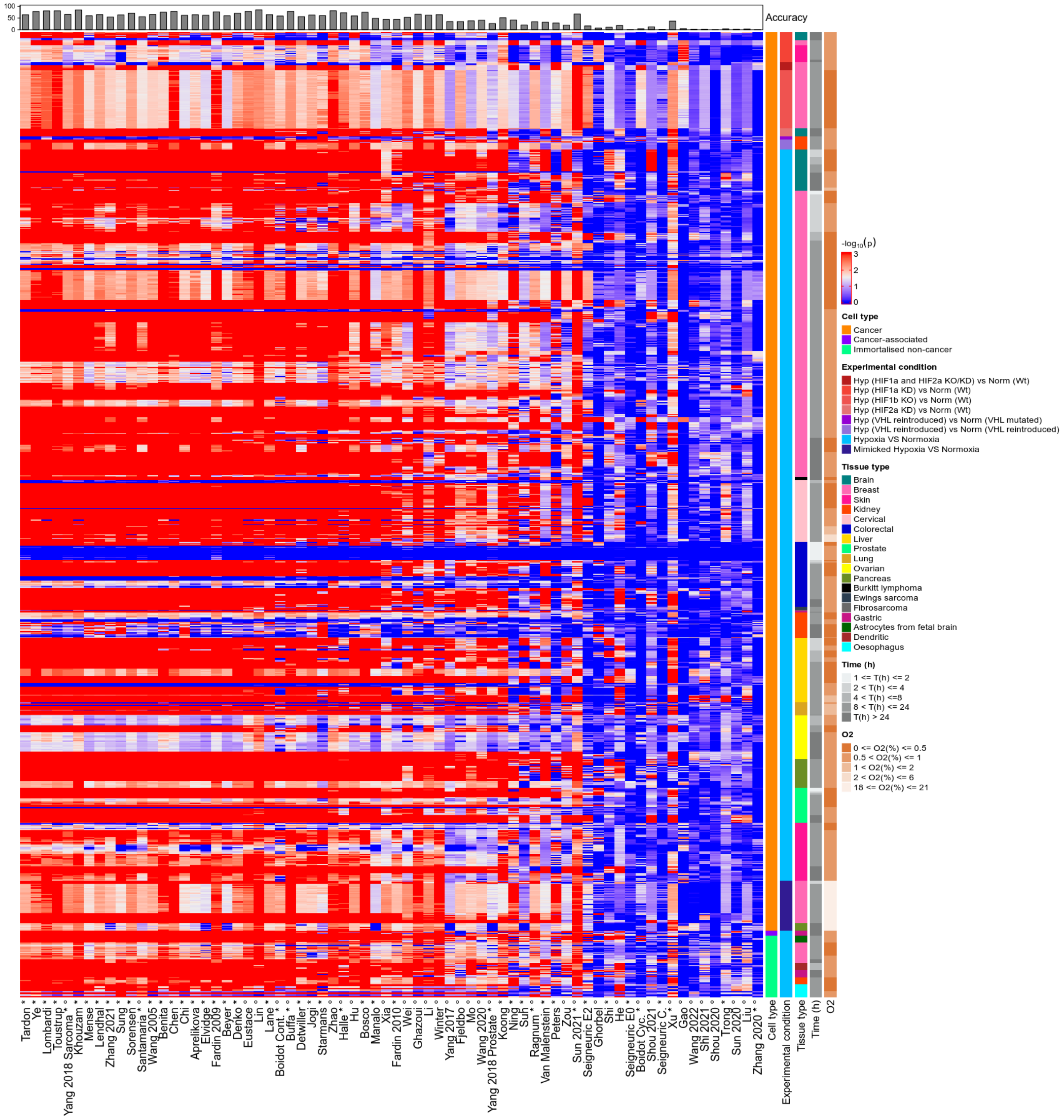

100
50
0
Accuracy
-log10(p)
3
2
1
0
Cell type
Cancer
Cancer-associated
Immortalised non-cancer
Experimental condition
Hyp (HIF1a and HIF2a KO/KD) vs Norm (Wt)
Hyp (HIF1a KD) vs Norm (Wt)
Hyp (HIF1b KO) vs Norm (Wt)
Hyp (HIF2a KD) vs Norm (Wt)
Hyp (VHL reintroduced) vs Norm (VHL mutated)
Hyp (VHL reintroduced) vs Norm (VHL reintroduced)
Hypoxia VS Normoxia
Mimicked Hypoxia VS Normoxia
Tissue type
Brain
Breast
Skin
Kidney
Cervical
Colorectal
Liver
Prostate
Lung
Ovarian
Pancreas
Burkitt lymphoma
Ewings sarcoma
Fibrosarcoma
Gastric
Astrocytes from fetal brain
Dendritic
Oesophagus
Time (h)
1 <= T(h) <= 2
2 < T(h) <= 4
4 < T(h) <=8
8 < T(h) <= 24
T(h) > 24
O2
0 <= O2(%) <= 0.5
0.5 < O2(%) <= 1
1 < O2(%) <= 2
2 < O2(%) <= 6
18 <= O2(%) <= 21
Tardon *
Ye *
Lombardi *
Toustrup *
Yang 2018 Sarcoma *
Khouzam *
Mense *
Lendhal *
Zhang 2021 *
Sung *
Sorensen *
Santamaria °
Wang 2005 *
Benita *
Chen *
Chi *
Aprelikova *
Elvidge *
Fardin 2009 *
Beyer *
Denko °
Eustace °
Lin *
Lane *
Boidot Cont. *
Buffa °
Detwiller *
Jogi *
Starmans °
Zhao *
Halle °
Hu *
Bosco *
Manalo °
Xia °
Fardin 2010 *
Wei °
Ghazoui *
Li °
Winter °
Yang 2017 °
Fjeldbo °
Mo °
Wang 2020 *
Yang 2018 Prostate *
Koong *
Ning *
Suh °
Ragnum *
Van Malenstein *
Peters °
Zou *
Sun 2021 *
Seigneuric E2 *
Ghorbel °
Shi *
He °
Seigneuric E0 *
Boidot Cyc. °
Shou 2021 °
Seigneuric C. *
Xu *
Gao °
Wang 2022 °
Shi 2021 °
Shou 2020 °
Trong *
Sun 2020 °
Liu °
Zhang 2020 *
Cell type
Experimental condition
Tissue type
Time (h)
O2

Supplementary Figure S2: Complex heatmap (mean)

Complex heatmaps of signatures p-values in hypoxic vs. non-hypoxic samples across GEO cell-line conditions in bulk RNAseq and microarray data. Shades of red and blue denote low and high p-values, respectively, as derived from permutation tests and represent the likelihood that a random gene signature would outperform a hypoxia signature. The top bar plot summarises signature accuracy, calculated as the number of pairwise combinations with p-values < 0.05 over the total number of pairwise combinations. The right-hand legend provides additional sample features, with titles located at the beginning of the x-axis, related to Figure 3.

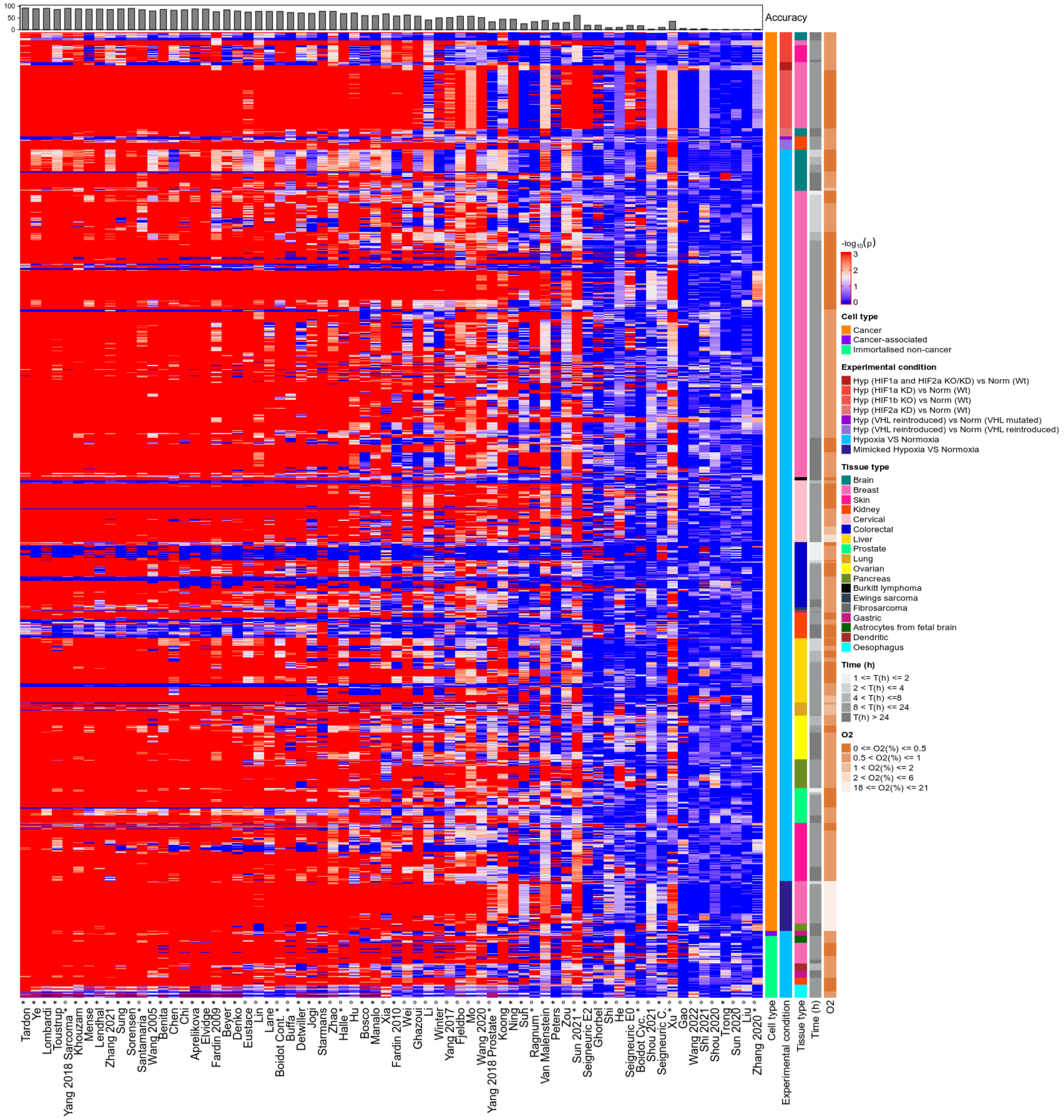

Accuracy
100
50
0
-log10(p)
3
2
1
0
Cell type
Cancer
Cancer-associated
Immortalised non-cancer
Experimental condition
Hyp (HIF1a and HIF2a KO/KD) vs Norm (Wt)
Hyp (HIF1a KD) vs Norm (Wt)
Hyp (HIF1b KO) vs Norm (Wt)
Hyp (HIF2a KD) vs Norm (Wt)
Hyp (VHL reintroduced) vs Norm (VHL mutated)
Hyp (VHL reintroduced) vs Norm (VHL reintroduced)
Hypoxia VS Normoxia
Mimicked Hypoxia VS Normoxia
Tissue type
Brain
Breast
Skin
Kidney
Cervical
Colorectal
Liver
Prostate
Lung
Ovarian
Pancreas
Burkitt lymphoma
Ewings sarcoma
Fibrosarcoma
Gastric
Astrocytes from fetal brain
Dendritic
Oesophagus
Time (h)
1 <= T(h) <= 2
2 < T(h) <= 4
4 < T(h) <=8
8 < T(h) <= 24
T(h) > 24
O2
0 <= O2(%) <= 0.5
0.5 < O2(%) <= 1
1 < O2(%) <= 2
2 < O2(%) <= 6
18 <= O2(%) <= 21
Tardon
Ye
Lombardi
Toustrup
Yang 2018 Sarcoma
Khouzam
Mense
Lendahl
Zhang 2021
Sung
Sorensen
Santamaria
Wang 2005
Benita
Chen
Chi
Aprelikova
Elvidge
Fardin 2009
Beyer
Denko
Eustace
Lin
Lane
Boidot Cont.
Buffa
Detwiller
Jogi
Starmans
Zhao
Halle
Hu
Bosco
Manalo
Xia
Fardin 2010
Wei
Ghazoui
Li
Winter
Yang 2017
Fjeldbo
Mo
Wang 2020
Yang 2018 Prostate
Koong
Ning
Suh
Ragnum
Van Malenstein
Peters
Zou
Sun 2021
Seigneuric E2
Ghorbel
Shi
He
Seigneuric E0
Boidot Cyc.
Shou 2021
Seigneuric C.
Xu
Gao
Wang 2022
Shi 2021
Shou 2020
Trong
Sun 2020
Liu
Zhang 2020
Cell type
Experimental condition
Tissue type
Time (h)
O2

Supplementary Figure S3: Complex heatmap (median)

Complex heatmap of signature p-values in hypoxic vs. non-hypoxic samples across GEO cell-line conditions in bulk RNAseq and microarray data, related to Figure 3. Conventions as in S2.

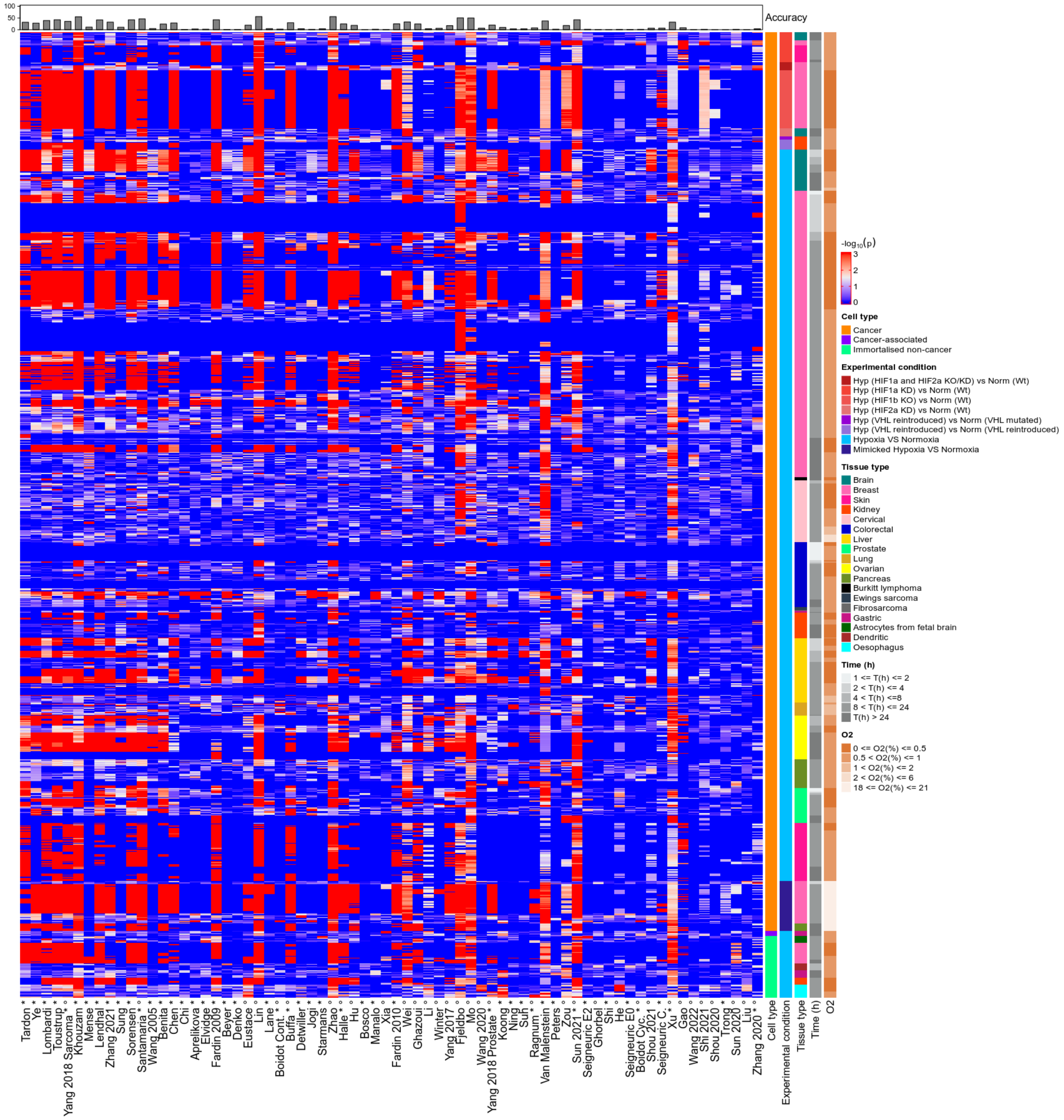

100
50
0
Accuracy
-log10(p)
3
2
1
0
Cell type
Cancer
Cancer-associated
Immortalised non-cancer
Experimental condition
Hyp (HIF1a and HIF2a KO/KD) vs Norm (Wt)
Hyp (HIF1a KD) vs Norm (Wt)
Hyp (HIF1b KO) vs Norm (Wt)
Hyp (HIF2a KD) vs Norm (Wt)
Hyp (VHL reintroduced) vs Norm (VHL mutated)
Hyp (VHL reintroduced) vs Norm (VHL reintroduced)
Hypoxia VS Normoxia
Mimicked Hypoxia VS Normoxia
Tissue type
Brain
Breast
Skin
Kidney
Cervical
Colorectal
Liver
Prostate
Lung
Ovarian
Pancreas
Burkitt lymphoma
Ewings sarcoma
Fibrosarcoma
Gastric
Astrocytes from fetal brain
Dendritic
Oesophagus
Time (h)
1 <= T(h) <= 2
2 < T(h) <= 4
4 < T(h) <=8
8 < T(h) <= 24
T(h) > 24
O2
0 <= O2(%) <= 0.5
0.5 < O2(%) <= 1
1 < O2(%) <= 2
2 < O2(%) <= 6
18 <= O2(%) <= 21
Tardon
Ye
Lombardi
Toustrup
Yang 2018 Sarcoma
Khouzam
Mense
Lendhal
Zhang 2021
Sung
Sorensen
Santamaria
Wang 2005
Benita
Chen
Chi
Aprelikova
Elvidge
Fardin 2009
Beyer
Denko
Eustace
Lin
Lane
Boidot Cont.
Buffa
Detwiller
Jogi
Starmans
Zhao
Halle
Hu
Bosco
Manalo
Xia
Fardin 2010
Wei
Ghazoui
Li
Winter
Yang 2017
Fjeldbo
Mo
Wang 2020
Yang 2018 Prostate
Koong
Ning
Suh
Ragnum
Van Malenstein
Peters
Zou
Sun 2021
Seigneuric E2
Ghorbel
Shi
He
Seigneuric E0
Boidot Cyc.
Shou 2021
Seigneuric C.
Xu
Gao
Wang 2022
Shi 2021
Shou 2020
Trong
Sun 2020
Liu
Zhang 2020
Cell type
Experimental condition
Tissue type
Time (h)
O2

Supplementary Figure S4: Complex heatmap (mode)

Complex heatmap of signature p-values in hypoxic vs. non-hypoxic samples across GEO cell-line conditions in bulk RNAseq and microarray data, related to Figure 3. Conventions as in S2.

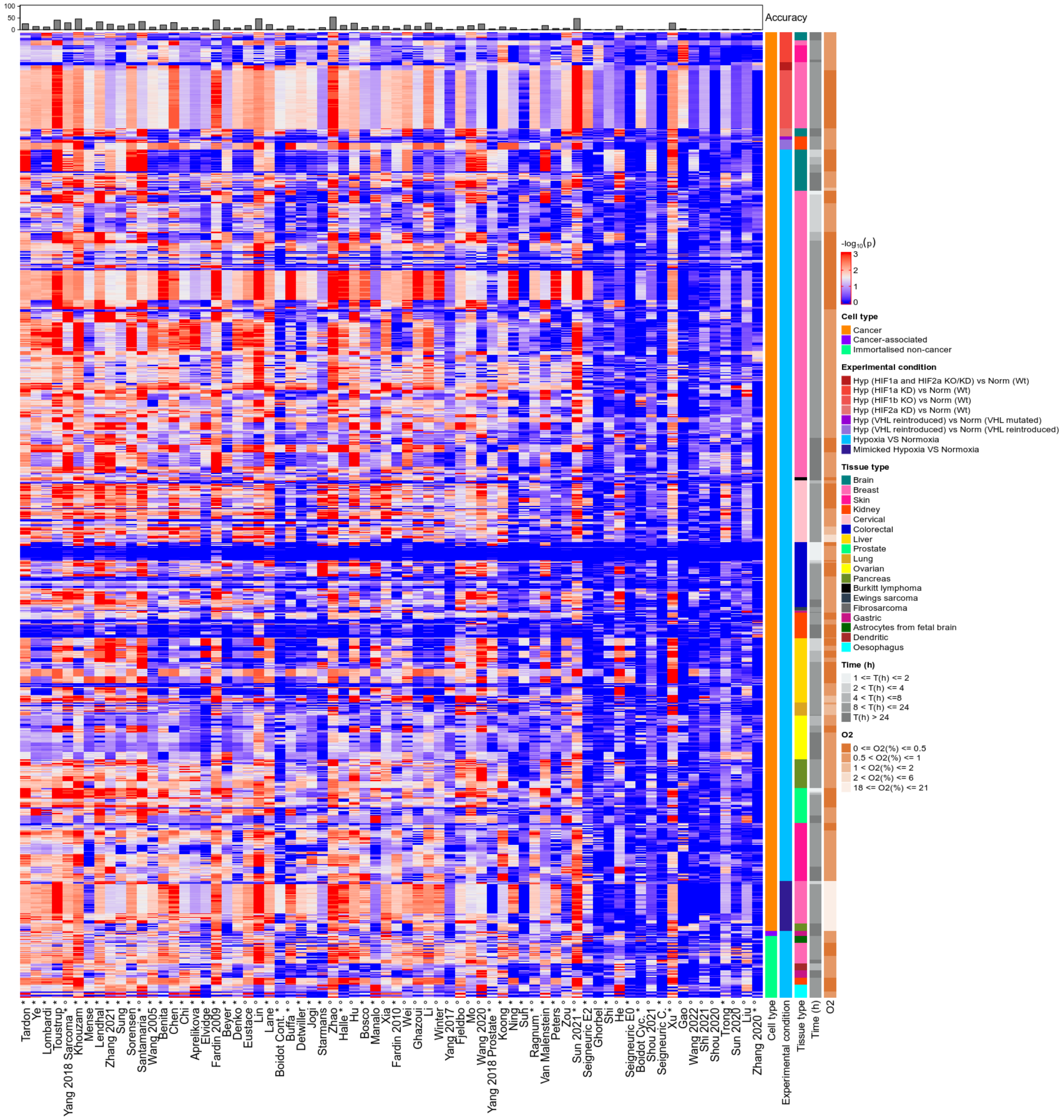

100
50
0
Accuracy
Tardon *
Ye *
Lombardi *
Toustrup *
Yang 2018 Sarcoma °
Khouzam *
Mense *
Lendhal *
Zhang 2021 *
Sung *
Sorensen *
Santamaria °
Wang 2005 *
Benita *
Chen *
Chi *
Aprelikova *
Elvidge *
Fardin 2009 *
Beyer *
Denko *
Eustace °
Lin °
Lane *
Boidot Cont. °
Buffa °
Detwiller *
Jogi *
Starmans *
Zhao °
Halle °
Hu °
Bosco *
Manalo *
Xia °
Fardin 2010 *
Wei °
Ghazoui °
Li °
Winter °
Yang 2017 °
Fjeldbo °
Mo °
Wang 2020 °
Yang 2018 Prostate °
Koong *
Ning *
Suh *
Ragnum °
Van Malenstein *
Peters *
Zou °
Sun 2021 °
Seigneuric E2 *
Ghorbel °
Shi *
He °
Seigneuric E0 *
Boidot Cyc. °
Shou 2021 °
Seigneuric C. *
Xu °
Gao °
Wang 2022 °
Shi 2021 °
Shou 2020 °
Trong *
Sun 2020 °
Liu °
Zhang 2020 °
Cell type
Experimental condition
Tissue type
Time (h)
O2
-log10(p)
3
2
1
0
Cell type
Cancer
Cancer-associated
Immortalised non-cancer
Experimental condition
Hyp (HIF1a and HIF2a KO/KD) vs Norm (Wt)
Hyp (HIF1a KD) vs Norm (Wt)
Hyp (HIF1b KO) vs Norm (Wt)
Hyp (HIF2a KD) vs Norm (Wt)
Hyp (VHL reintroduced) vs Norm (VHL mutated)
Hyp (VHL reintroduced) vs Norm (VHL reintroduced)
Hypoxia VS Normoxia
Mimicked Hypoxia VS Normoxia
Tissue type
Brain
Breast
Skin
Kidney
Cervical
Colorectal
Liver
Prostate
Lung
Ovarian
Pancreas
Burkitt lymphoma
Ewings sarcoma
Fibrosarcoma
Gastric
Astrocytes from fetal brain
Dendritic
Oesophagus
Time (h)
1 <= T(h) <= 2
2 < T(h) <= 4
4 < T(h) <=8
8 < T(h) <= 24
T(h) > 24
O2
0 <= O2(%) <= 0.5
0.5 < O2(%) <= 1
1 < O2(%) <= 2
2 < O2(%) <= 6
18 <= O2(%) <= 21

Supplementary Figure S5: Complex heatmap (midrange)

Complex heatmap of signature p-values in hypoxic vs. non-hypoxic samples across GEO cell-line conditions in bulk RNAseq and microarray data, related to Figure 3. Conventions as in S2.

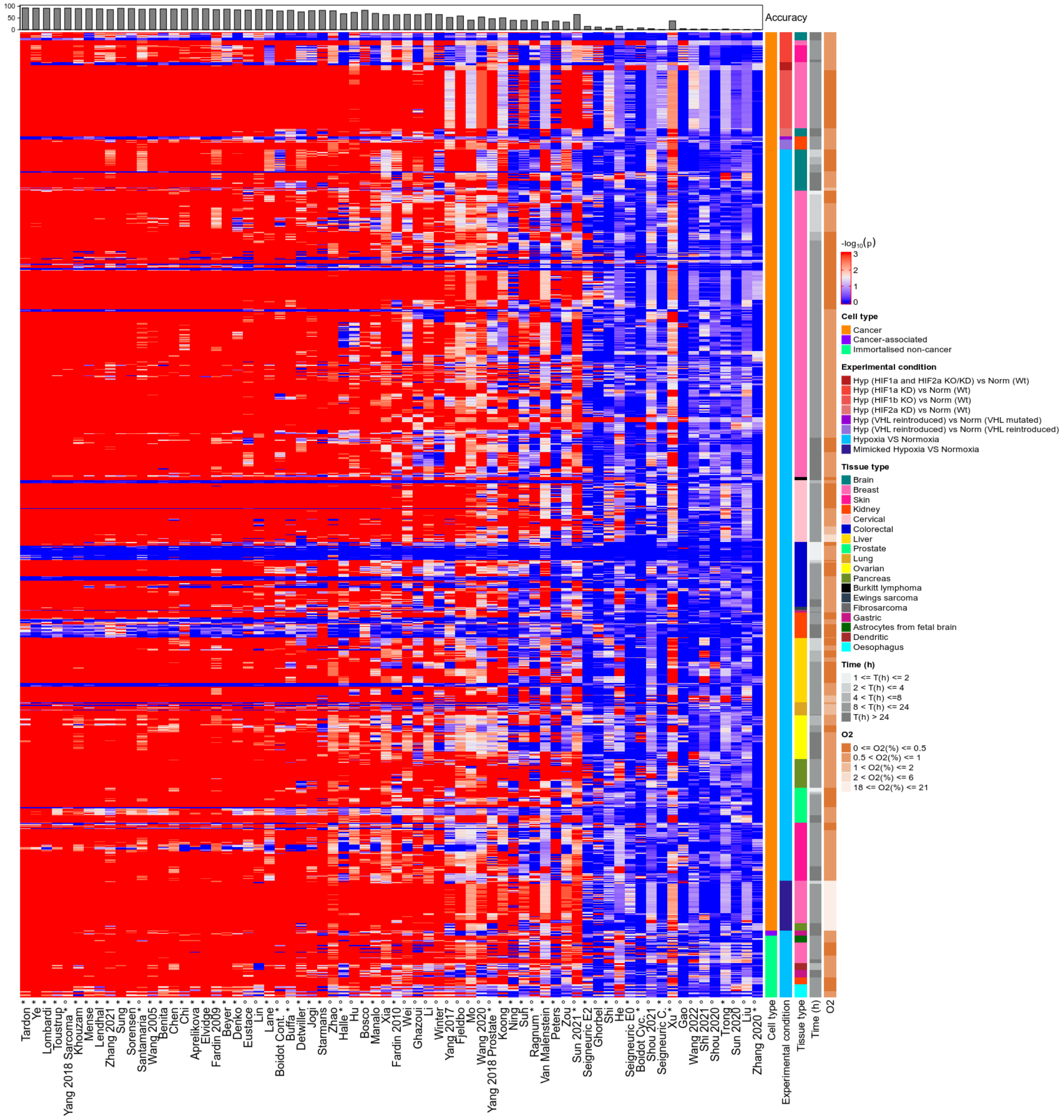
Accuracy
100
50
0
Tardon
Ye
Lombardi
Toustrup
Yang 2018 Sarcoma
Khouzam
Mense
Lendahl
Zhang 2021
Sung
Sorensen
Santamaria
Wang 2005
Benita
Chen
Chi
Aprelikova
Elvidge
Fardin 2009
Beyer
Denko
Eustace
Lin
Lane
Boidot Cont.
Buffa
Detwiller
Jogi
Starmans
Zhao
Halle
Hu
Bosco
Manalo
Xia
Fardin 2010
Wei
Ghazoui
Li
Winter
Yang 2017
Fjeldbo
Mo
Wang 2020
Yang 2018 Prostate
Koong
Ning
Suh
Ragnum
Van Malenstein
Peters
Zou
Sun 2021
Seigneuric E2
Ghorbel
Shi
He
Seigneuric E0
Boidot Cyc.
Shou 2021
Seigneuric C.
Xu
Gao
Wang 2022
Shi 2021
Shou 2020
Trong
Sun 2020
Liu
Zhang 2020
Cell type
Experimental condition
Tissue type
Time (h)
O2
-log10(p)
3
2
1
0
Cell type
Cancer
Cancer-associated
Immortalised non-cancer
Experimental condition
Hyp (HIF1a and HIF2a KO/KD) vs Norm (Wt)
Hyp (HIF1a KD) vs Norm (Wt)
Hyp (HIF1b KO) vs Norm (Wt)
Hyp (HIF2a KD) vs Norm (Wt)
Hyp (VHL reintroduced) vs Norm (VHL mutated)
Hyp (VHL reintroduced) vs Norm (VHL reintroduced)
Hypoxia VS Normoxia
Mimicked Hypoxia VS Normoxia
Tissue type
Brain
Breast
Skin
Kidney
Cervical
Colorectal
Liver
Prostate
Lung
Ovarian
Pancreas
Burkitt lymphoma
Ewings sarcoma
Fibrosarcoma
Gastric
Astrocytes from fetal brain
Dendritic
Oesophagus
Time (h)
1 <= T(h) <= 2
2 < T(h) <= 4
4 < T(h) <=8
8 < T(h) <= 24
T(h) > 24
O2
0 <= O2(%) <= 0.5
0.5 < O2(%) <= 1
1 < O2(%) <= 2
2 < O2(%) <= 6
18 <= O2(%) <= 21

Supplementary Figure S6: Complex heatmap (midhinge)

Complex heatmap of signature p-values in hypoxic vs. non-hypoxic samples across GEO cell-line conditions in bulk RNAseq and microarray data, related to Figure 3. Conventions as in S2.

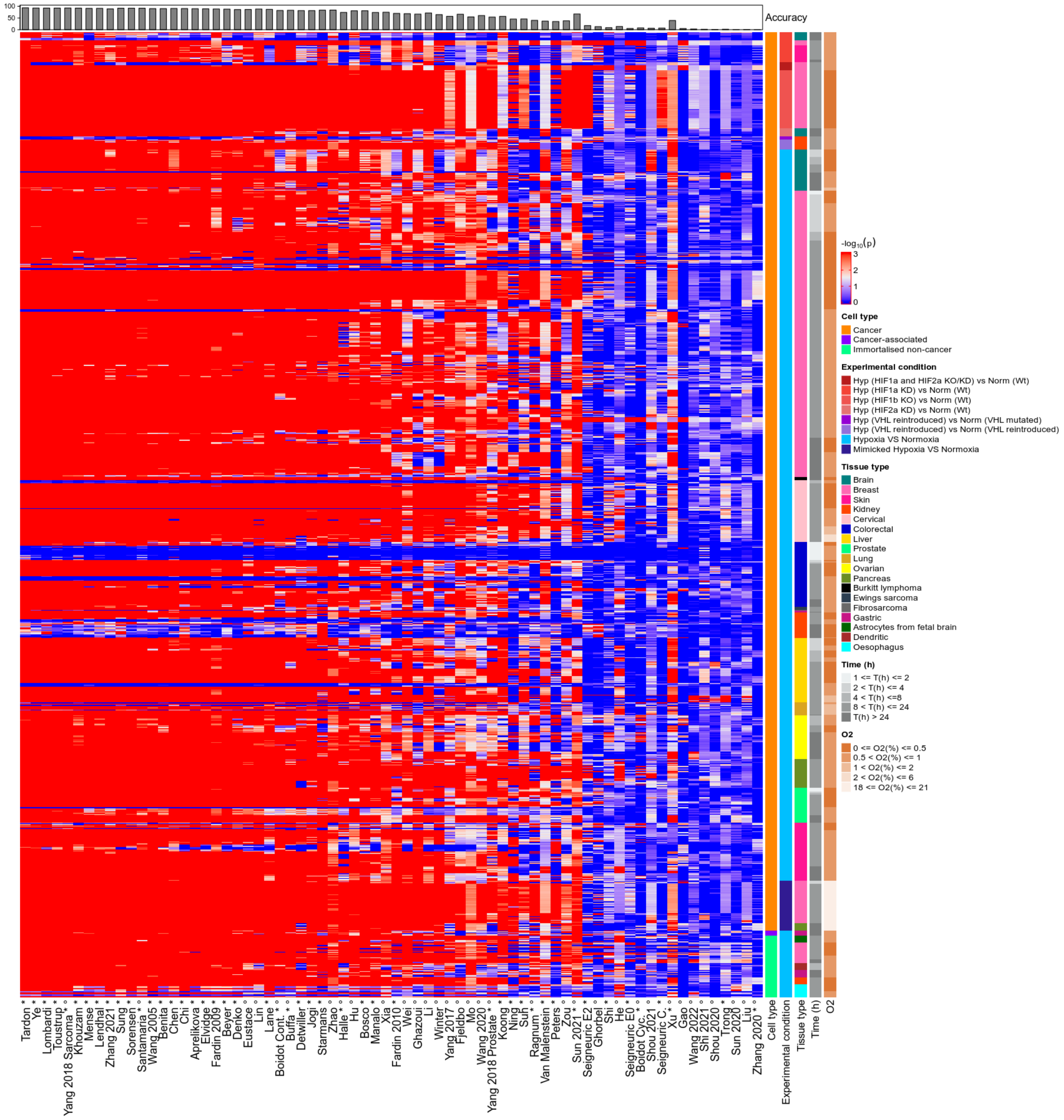

100
50
0
Accuracy
-log10(p)
3
2
1
0
Cell type
Cancer
Cancer-associated
Immortalised non-cancer
Experimental condition
Hyp (HIF1a and HIF2a KO/KD) vs Norm (Wt)
Hyp (HIF1a KD) vs Norm (Wt)
Hyp (HIF1b KO) vs Norm (Wt)
Hyp (HIF2a KD) vs Norm (Wt)
Hyp (VHL reintroduced) vs Norm (VHL mutated)
Hyp (VHL reintroduced) vs Norm (VHL reintroduced)
Hypoxia VS Normoxia
Mimicked Hypoxia VS Normoxia
Tissue type
Brain
Breast
Skin
Kidney
Cervical
Colorectal
Liver
Prostate
Lung
Ovarian
Pancreas
Burkitt lymphoma
Ewings sarcoma
Fibrosarcoma
Gastric
Astrocytes from fetal brain
Dendritic
Oesophagus
Time (h)
1 <= T(h) <= 2
2 < T(h) <= 4
4 < T(h) <=8
8 < T(h) <= 24
T(h) > 24
O2
0 <= O2(%) <= 0.5
0.5 < O2(%) <= 1
1 < O2(%) <= 2
2 < O2(%) <= 6
18 <= O2(%) <= 21
Tardon
Ye
Lombardi
Toustrup
Yang 2018 Sarcoma
Khouzam
Mense
Lendahl
Zhang 2021
Sung
Sorensen
Santamaria
Wang 2005
Benita
Chen
Chi
Aprelikova
Elvidge
Fardin 2009
Beyer
Denko
Eustace
Lin
Lane
Boidot Cont.
Buffa
Detwiller
Jogi
Starmans
Zhao
Halle
Hu
Bosco
Manalo
Xia
Fardin 2010
Wei
Ghazoui
Li
Winter
Yang 2017
Fjeldbo
Mo
Wang 2020
Yang 2018 Prostate
Koong
Ning
Suh
Ragnum
Van Malenstein
Peters
Zou
Sun 2021
Seigneuric E2
Ghorbel
Shi
He
Seigneuric E0
Boidot Cyc.
Shou 2021
Seigneuric C.
Xu
Gao
Wang 2022
Shi 2021
Shou 2020
Trong
Sun 2020
Liu
Zhang 2020
Cell type
Experimental condition
Tissue type
Time (h)
O2

Supplementary Figure S7: Complex heatmap (trimean)

Complex heatmap of signature p-values in hypoxic vs. non-hypoxic samples across GEO cell-line conditions in bulk RNAseq and microarray data, related to Figure 3. Conventions as in S2.

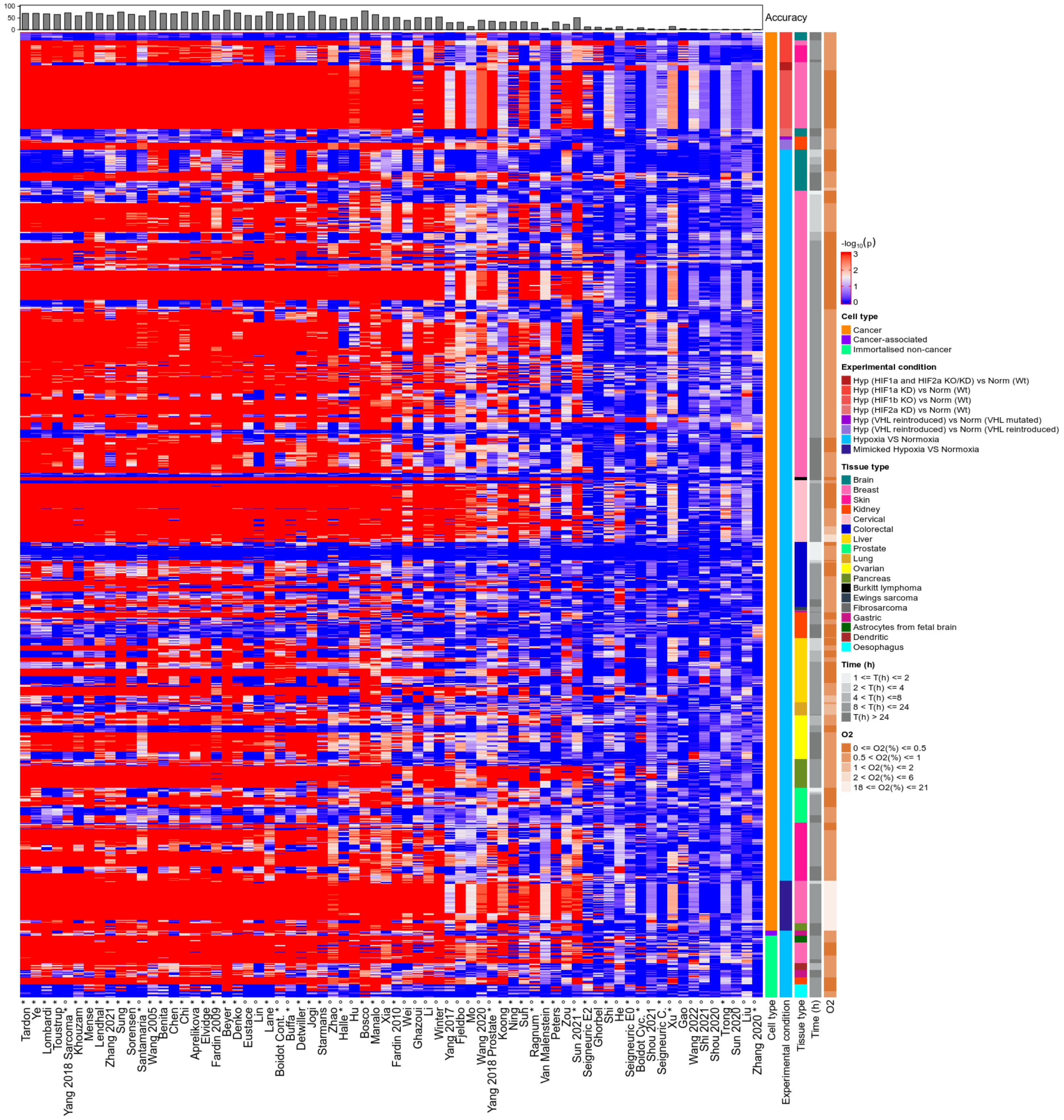

Accuracy
100
50
0
-log10(p)
3
2
1
0
Cell type
Cancer
Cancer-associated
Immortalised non-cancer
Experimental condition
Hyp (HIF1a and HIF2a KO/KD) vs Norm (Wt)
Hyp (HIF1a KD) vs Norm (Wt)
Hyp (HIF1b KO) vs Norm (Wt)
Hyp (HIF2a KD) vs Norm (Wt)
Hyp (VHL reintroduced) vs Norm (VHL mutated)
Hyp (VHL reintroduced) vs Norm (VHL reintroduced)
Hypoxia VS Normoxia
Mimicked Hypoxia VS Normoxia
Tissue type
Brain
Breast
Skin
Kidney
Cervical
Colorectal
Liver
Prostate
Lung
Ovarian
Pancreas
Burkitt lymphoma
Ewings sarcoma
Fibrosarcoma
Gastric
Astrocytes from fetal brain
Dendritic
Oesophagus
Time (h)
1 <= T(h) <= 2
2 < T(h) <= 4
4 < T(h) <=8
8 < T(h) <= 24
T(h) > 24
O2
0 <= O2(%) <= 0.5
0.5 < O2(%) <= 1
1 < O2(%) <= 2
2 < O2(%) <= 6
18 <= O2(%) <= 21
Tardon *
Ye *
Lombardi *
Toustrup *
Yang 2018 Sarcoma *
Khouzam *
Mense *
Lendhal *
Zhang 2021 *
Sung *
Sorensen *
Santamaria *
Wang 2005 *
Benita *
Chen *
Chi *
Aprelikova *
Elvidge *
Fardin 2009 *
Beyer *
Denko *
Eustace *
Lin *
Lane *
Boidot Cont. *
Buffa *
Detwiller *
Jogi *
Starmans *
Zhao *
Halle *
Hu *
Bosco *
Manalo *
Xia *
Fardin 2010 *
Wei *
Ghazoui *
Li *
Winter *
Yang 2017 *
Fjeldbo *
Mo *
Wang 2020 *
Yang 2018 Prostate *
Koong *
Ning *
Suh *
Ragnum *
Van Malenstein *
Peters *
Zou *
Sun 2021 *
Seigneuric E2 *
Ghorbel *
Shi *
He *
Seigneuric E0 *
Boidot Cyc. *
Shou 2021 *
Seigneuric C. *
Xu *
Gao *
Wang 2022 *
Shi 2021 *
Shou 2020 *
Trong *
Sun 2020 *
Liu *
Zhang 2020 *
Cell type
Experimental condition
Tissue type
Time (h)
O2

Supplementary Figure S8: Complex heatmap (IQR)

Complex heatmap of signature p-values in hypoxic vs. non-hypoxic samples across GEO cell-line conditions in bulk RNAseq and microarray data, related to Figure 3. Conventions as in S2.

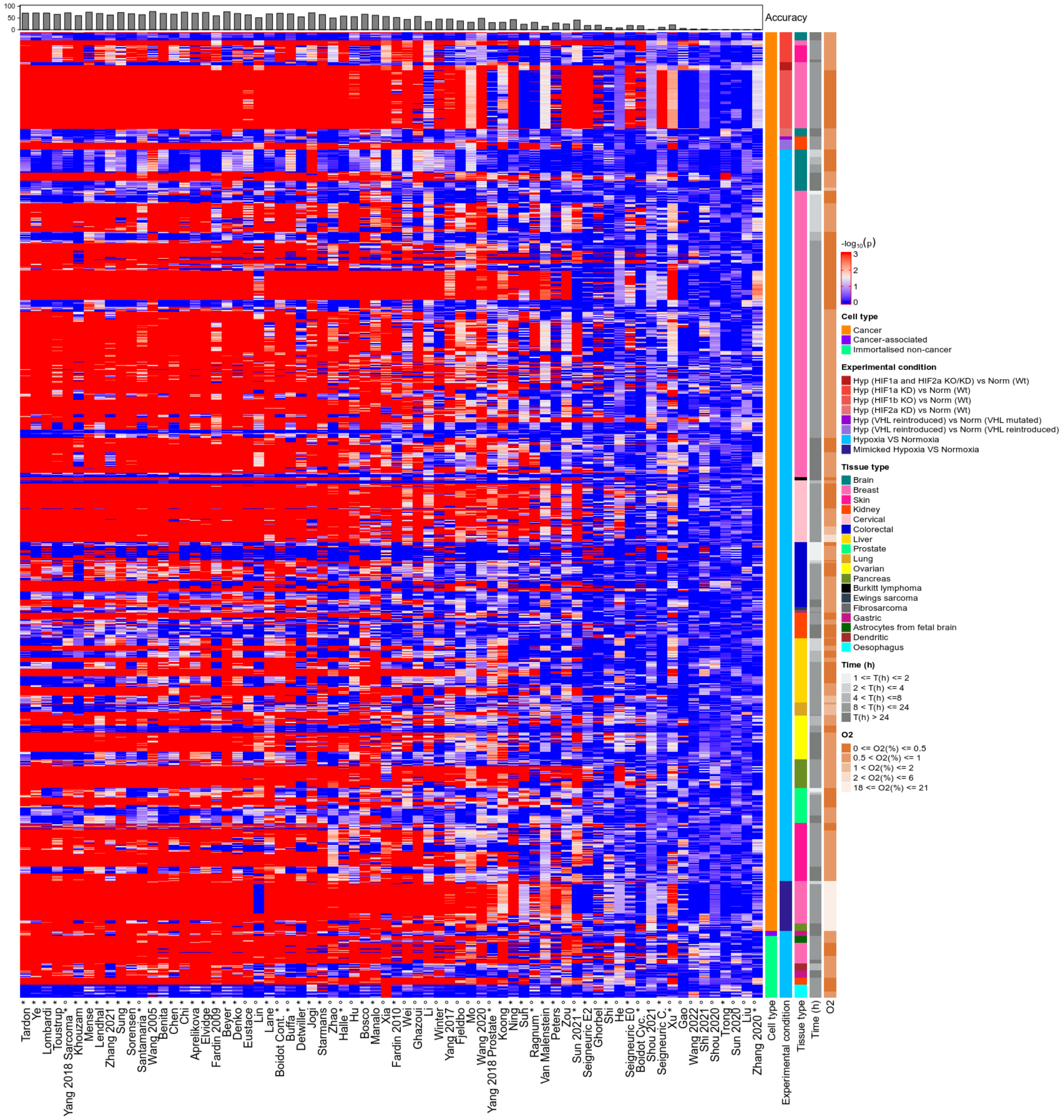

100
50
0
Accuracy
-log10(p)
3
2
1
0
Cell type
Cancer
Cancer-associated
Immortalised non-cancer
Experimental condition
Hyp (HIF1a and HIF2a KO/KD) vs Norm (Wt)
Hyp (HIF1a KD) vs Norm (Wt)
Hyp (HIF1b KO) vs Norm (Wt)
Hyp (HIF2a KD) vs Norm (Wt)
Hyp (VHL reintroduced) vs Norm (VHL mutated)
Hyp (VHL reintroduced) vs Norm (VHL reintroduced)
Hypoxia VS Normoxia
Mimicked Hypoxia VS Normoxia
Tissue type
Brain
Breast
Skin
Kidney
Cervical
Colorectal
Liver
Prostate
Lung
Ovarian
Pancreas
Burkitt lymphoma
Ewings sarcoma
Fibrosarcoma
Gastric
Astrocytes from fetal brain
Dendritic
Oesophagus
Time (h)
1 <= T(h) <= 2
2 < T(h) <= 4
4 < T(h) <=8
8 < T(h) <= 24
T(h) > 24
O2
0 <= O2(%) <= 0.5
0.5 < O2(%) <= 1
1 < O2(%) <= 2
2 < O2(%) <= 6
18 <= O2(%) <= 21
Tardon
Ye
Lombardi
Toustrup
Yang 2018 Sarcoma
Khouzam
Mense
Lendhal
Zhang 2021
Sung
Sorensen
Santamaria
Wang 2005
Benita
Chen
Chi
Aprelikova
Elvidge
Fardin 2009
Beyer
Denko
Eustace
Lin
Lane
Boidot Cont.
Buffa
Detwiller
Jogi
Starmans
Zhao
Halle
Hu
Bosco
Manalo
Xia
Fardin 2010
Wei
Ghazoui
Li
Winter
Yang 2017
Fjeldbo
Mo
Wang 2020
Yang 2018 Prostate
Koong
Ning
Suh
Ragnum
Van Malenstein
Peters
Zou
Sun 2021
Seigneuric E2
Ghorbel
Shi
He
Seigneuric E0
Boidot Cyc.
Shou 2021
Seigneuric C.
Xu
Gao
Wang 2022
Shi 2021
Shou 2020
Trong
Sun 2020
Liu
Zhang 2020
Cell type
Experimental condition
Tissue type
Time (h)
O2

Supplementary Figure S9: Complex heatmap (MAD)

Complex heatmap of signature p-values in hypoxic vs. non-hypoxic samples across GEO cell-line conditions in bulk RNAseq and microarray data, related to Figure 3. Conventions as in S2.

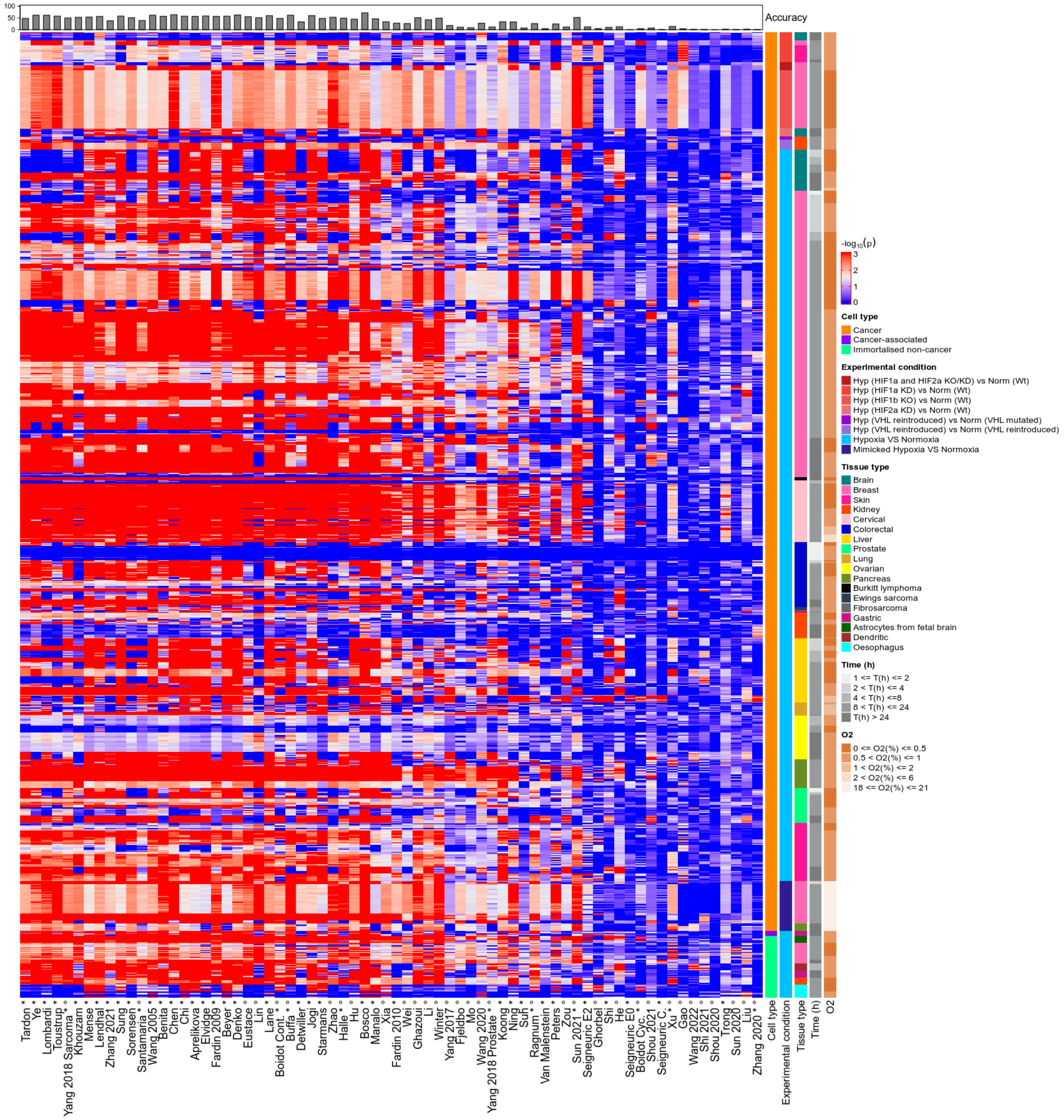

100
50
0
Accuracy
-log10(p)
3
2
1
0
Cell type
Cancer
Cancer-associated
Immortalised non-cancer
Experimental condition
Hyp (HIF1a and HIF2a KO/KD) vs Norm (Wt)
Hyp (HIF1a KD) vs Norm (Wt)
Hyp (HIF1b KO) vs Norm (Wt)
Hyp (HIF2a KD) vs Norm (Wt)
Hyp (VHL reintroduced) vs Norm (VHL mutated)
Hyp (VHL reintroduced) vs Norm (VHL reintroduced)
Hypoxia VS Normoxia
Mimicked Hypoxia VS Normoxia
Tissue type
Brain
Breast
Skin
Kidney
Cervical
Colorectal
Liver
Prostate
Lung
Ovarian
Pancreas
Burkitt lymphoma
Ewings sarcoma
Fibrosarcoma
Gastric
Astrocytes from fetal brain
Dendritic
Oesophagus
Time (h)
1 <= T(h) <= 2
2 < T(h) <= 4
4 < T(h) <=8
8 < T(h) <= 24
T(h) > 24
O2
0 <= O2(%) <= 0.5
0.5 < O2(%) <= 1
1 < O2(%) <= 2
2 < O2(%) <= 6
18 <= O2(%) <= 21
Tardon
Ye
Lombardi
Toustrup
Yang 2018 Sarcoma
Khouzam
Mense
Lendhal
Zhang 2021
Sung
Sorensen
Santamaria
Wang 2005
Benita
Chen
Chi
Aprelikova
Elvidge
Fardin 2009
Beyer
Denko
Eustace
Lin
Lane
Boidot Cont.
Buffa
Detwiller
Jogi
Starmans
Zhao
Halle
Hu
Bosco
Manalo
Xia
Fardin 2010
Wei
Ghazoui
Li
Winter
Yang 2017
Fjeldbo
Mo
Wang 2020
Yang 2018 Prostate
Koong
Ning
Suh
Ragnum
Van Malenstein
Peters
Zou
Sun 2021
Seigneuric E2
Ghorbel
Shi
He
Seigneuric E0
Boidot Cyc.
Shou 2021
Seigneuric C.
Xu
Gao
Wang 2022
Shi 2021
Shou 2020
Trong
Sun 2020
Liu
Zhang 2020
Cell type
Experimental condition
Tissue type
Time (h)
O2

Supplementary Figure S10: Complex heatmap (AAD)

Complex heatmap of signature p-values in hypoxic vs. non-hypoxic samples across GEO cell-line conditions in bulk RNAseq and microarray data, related to Figure 3. Conventions as in S2.

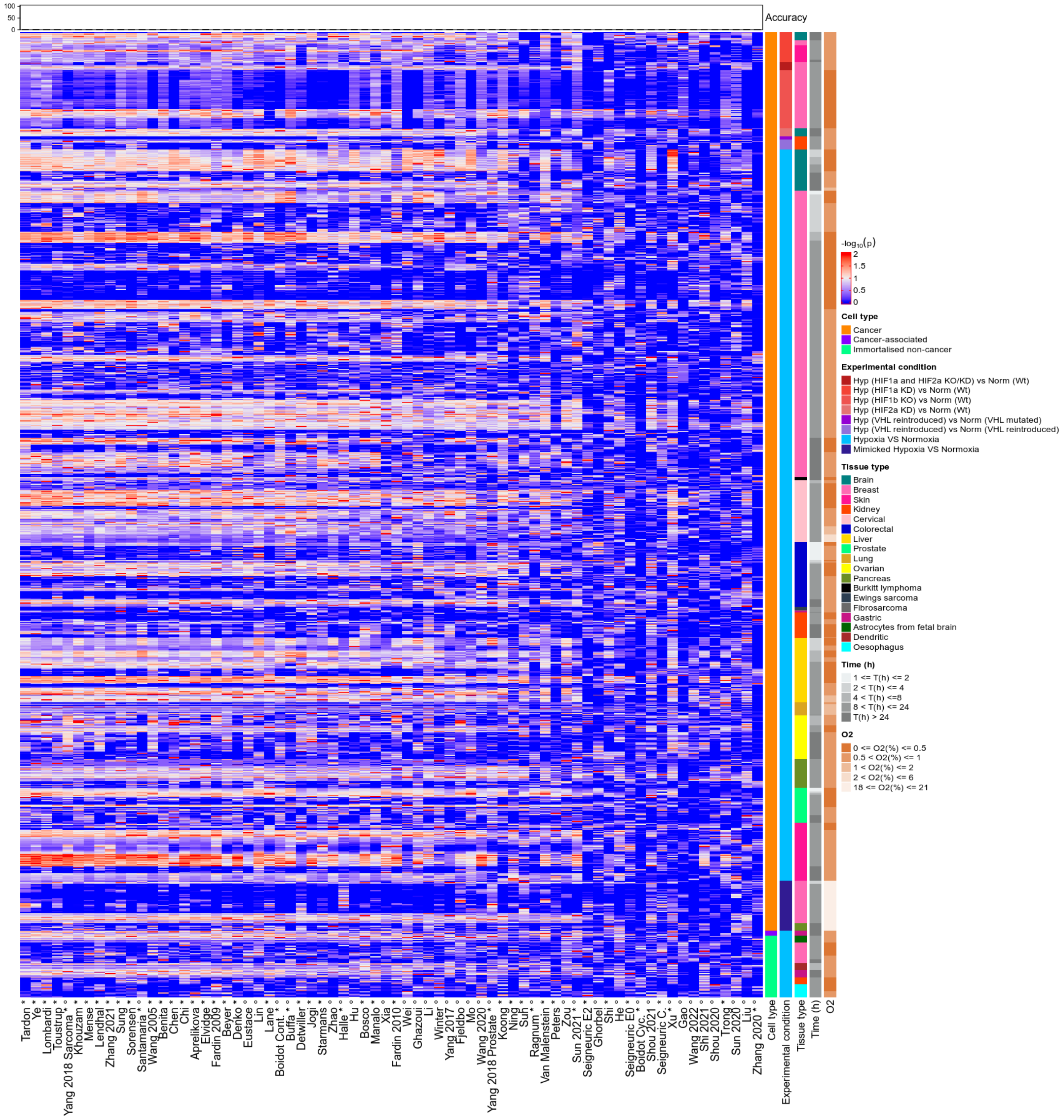

100
50
0
Accuracy
-log10(p)
2
1.5
1
0.5
0
Cell type
Cancer
Cancer-associated
Immortalised non-cancer
Experimental condition
Hyp (HIF1a and HIF2a KO/KD) vs Norm (Wt)
Hyp (HIF1a KD) vs Norm (Wt)
Hyp (HIF1b KO) vs Norm (Wt)
Hyp (HIF2a KD) vs Norm (Wt)
Hyp (VHL reintroduced) vs Norm (VHL mutated)
Hyp (VHL reintroduced) vs Norm (VHL reintroduced)
Hypoxia VS Normoxia
Mimicked Hypoxia VS Normoxia
Tissue type
Brain
Breast
Skin
Kidney
Cervical
Colorectal
Liver
Prostate
Lung
Ovarian
Pancreas
Burkitt lymphoma
Ewings sarcoma
Fibrosarcoma
Gastric
Astrocytes from fetal brain
Dendritic
Oesophagus
Time (h)
1 <= T(h) <= 2
2 < T(h) <= 4
4 < T(h) <=8
8 < T(h) <= 24
T(h) > 24
O2
0 <= O2(%) <= 0.5
0.5 < O2(%) <= 1
1 < O2(%) <= 2
2 < O2(%) <= 6
18 <= O2(%) <= 21
Tardon *
Ye *
Lombardi *
Toustrup *
Yang 2018 Sarcoma *
Khouzam *
Mense *
Lendhal *
Zhang 2021 *
Sung *
Sorensen *
Santamaria °
Wang 2005 *
Benita *
Chen *
Chi *
Aprelikova *
Elvidge *
Fardin 2009 *
Beyer *
Denko *
Eustace °
Lin *
Lane *
Boidot Cort. *
Buffa °
Detwiller *
Jogi *
Starmans *
Zhao °
Halle *
Hu °
Bosco *
Manalo *
Xia °
Fardin 2010 °
Wei °
Ghazoui °
Li °
Winter °
Yang 2017 °
Fjeldbo °
Mo °
Wang 2020 °
Yang 2018 Prostate *
Koong *
Ning *
Suh °
Ragnum *
Van Malenstein *
Peters *
Zou °
Sun 2021 *
Seigneuric E2 *
Ghorbel °
Shi *
He °
Seigneuric E0 *
Boidot Cyc. *
Shou 2021 °
Seigneuric C. *
Xu *
Gao °
Wang 2022 °
Shi 2021 °
Shou 2020 °
Trong *
Sun 2020 °
Liu °
Zhang 2020 *
Cell type
Experimental condition
Tissue type
Time (h)
O2

Supplementary Figure S11: Complex heatmap (ssGSEA)

Complex heatmap of signature p-values in hypoxic vs. non-hypoxic samples across GEO cell-line conditions in bulk RNAseq and microarray data, related to Figure 3. Conventions as in S2.

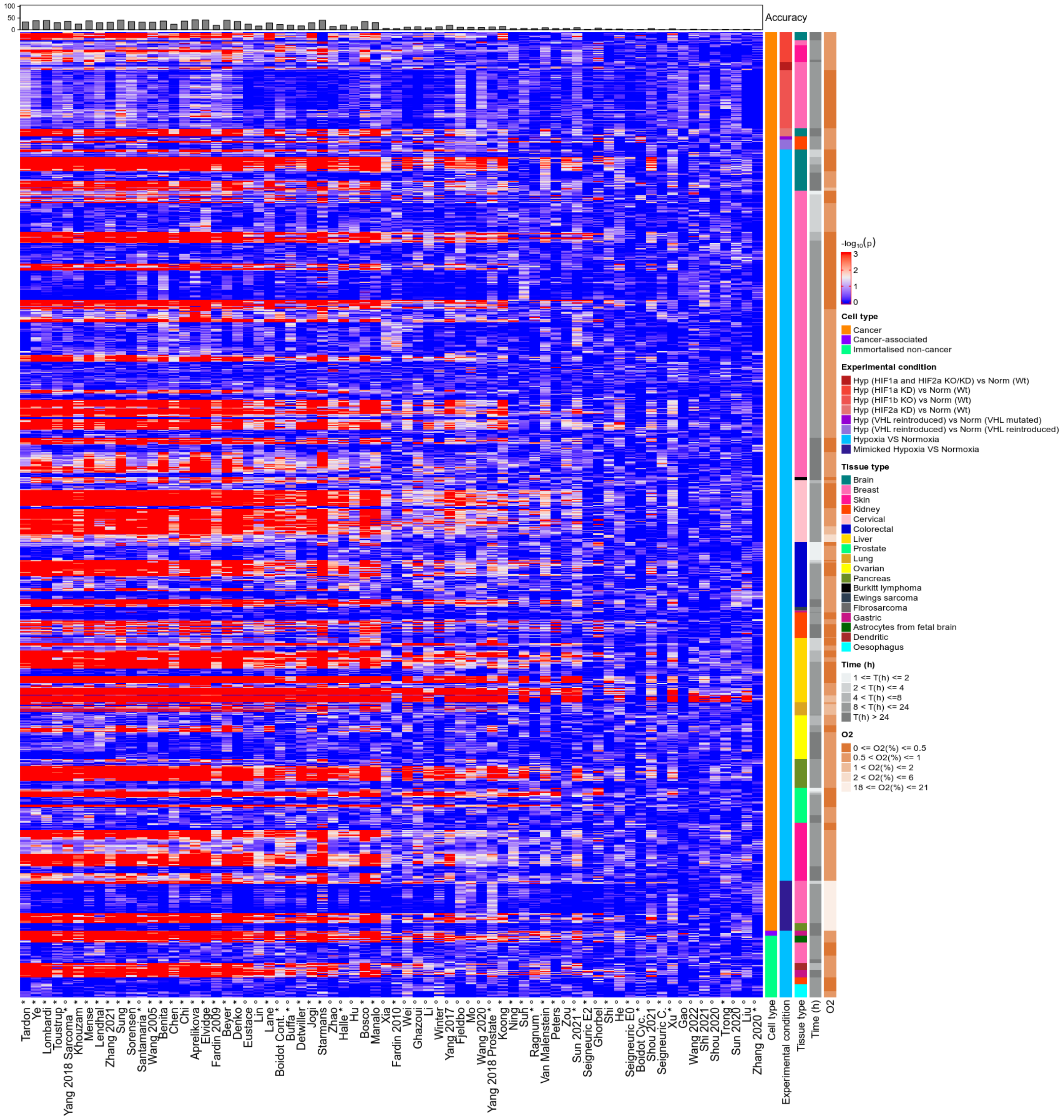

Accuracy
100
50
0
-log10(p)
3
2
1
0
Cell type
Cancer
Cancer-associated
Immortalised non-cancer
Experimental condition
Hyp (HIF1a and HIF2a KO/KD) vs Norm (Wt)
Hyp (HIF1a KD) vs Norm (Wt)
Hyp (HIF1b KO) vs Norm (Wt)
Hyp (HIF2a KD) vs Norm (Wt)
Hyp (VHL reintroduced) vs Norm (VHL mutated)
Hyp (VHL reintroduced) vs Norm (VHL reintroduced)
Hypoxia VS Normoxia
Mimicked Hypoxia VS Normoxia
Tissue type
Brain
Breast
Skin
Kidney
Cervical
Colorectal
Liver
Prostate
Lung
Ovarian
Pancreas
Burkitt lymphoma
Ewings sarcoma
Fibrosarcoma
Gastric
Astrocytes from fetal brain
Dendritic
Oesophagus
Time (h)
1 <= T(h) <= 2
2 < T(h) <= 4
4 < T(h) <=8
8 < T(h) <= 24
T(h) > 24
O2
0 <= O2(%) <= 0.5
0.5 < O2(%) <= 1
1 < O2(%) <= 2
2 < O2(%) <= 6
18 <= O2(%) <= 21
Tardon
Ye
Lombardi
Yang 2018 Sarcoma
Toustrup
Khouzam
Mense
Lendhal
Zhang 2021
Sung
Sorensen
Santamaria
Wang 2005
Benita
Chen
Chi
Aprelikova
Elvidge
Fardin 2009
Beyer
Denko
Eustace
Lin
Lane
Boidot Cont.
Buffa
Detwiller
Jogi
Starmans
Zhao
Halle
Hu
Bosco
Manalo
Xia
Fardin 2010
Wei
Ghazoui
Li
Winter
Yang 2017
Fjeldbo
Mo
Wang 2020
Yang 2018 Prostate
Koong
Ning
Suh
Ragnum
Van Malenstein
Peters
Zou
Sun 2021
Seigneuric E2
Ghorbel
Shi
He
Seigneuric E0
Boidot Cyc.
Shou 2021
Seigneuric C.
Xu
Gao
Wang 2022
Shi 2021
Shou 2020
Trong
Sun 2020
Liu
Zhang 2020
Cell type
Experimental condition
Tissue type
Time (h)
O2

Supplementary Figure S12: Complex heatmap (GSVA)

Complex heatmap of signature p-values in hypoxic vs. non-hypoxic samples across GEO cell-line conditions in bulk RNAseq and microarray data, related to Figure 3. Conventions as in S2.

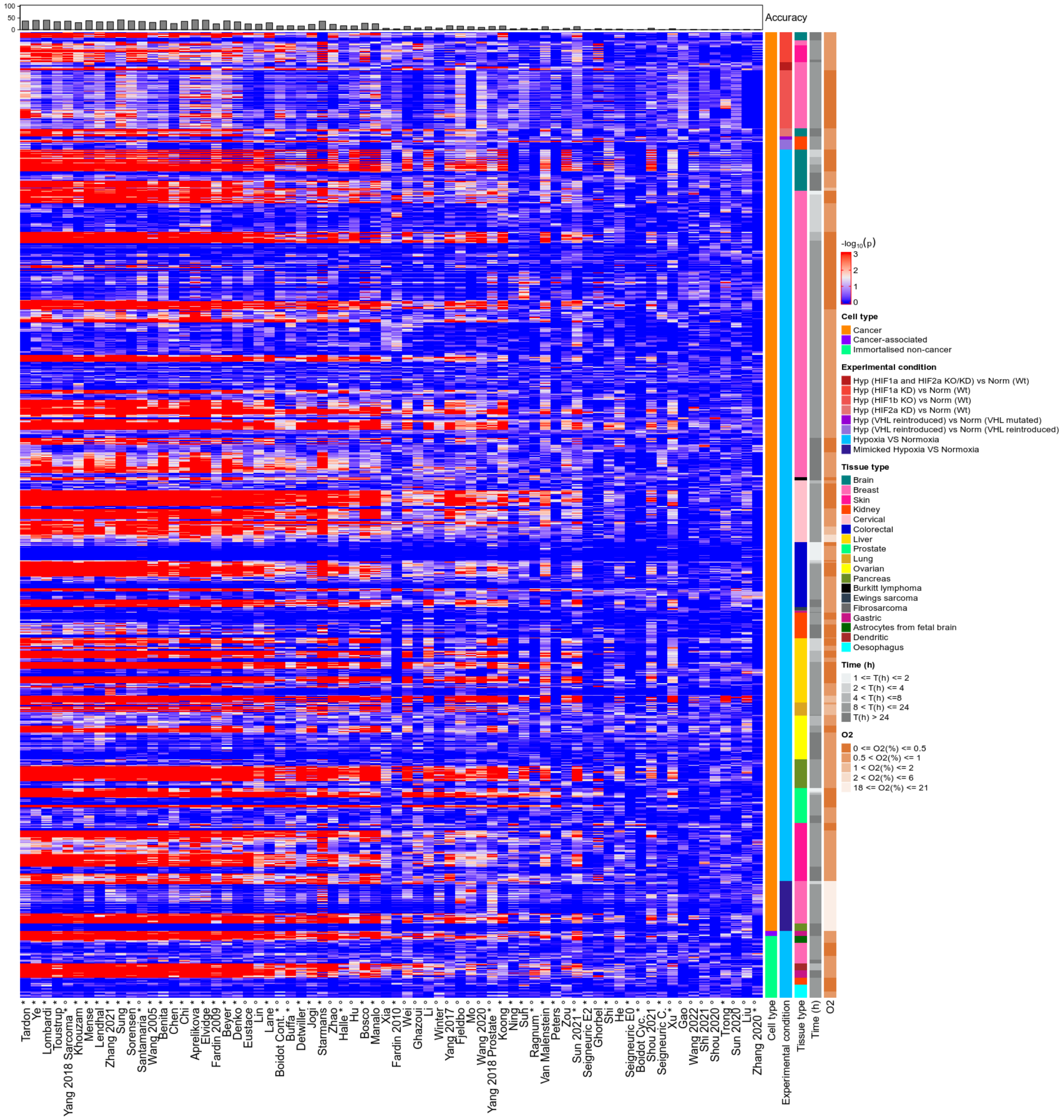

100
50
0
Accuracy
-log10(p)
3
2
1
0
Cell type
Cancer
Cancer-associated
Immortalised non-cancer
Experimental condition
Hyp (HIF1a and HIF2a KO/KD) vs Norm (Wt)
Hyp (HIF1a KD) vs Norm (Wt)
Hyp (HIF1b KO) vs Norm (Wt)
Hyp (HIF2a KD) vs Norm (Wt)
Hyp (VHL reintroduced) vs Norm (VHL mutated)
Hyp (VHL reintroduced) vs Norm (VHL reintroduced)
Hypoxia VS Normoxia
Mimicked Hypoxia VS Normoxia
Tissue type
Brain
Breast
Skin
Kidney
Cervical
Colorectal
Liver
Prostate
Lung
Ovarian
Pancreas
Burkitt lymphoma
Ewings sarcoma
Fibrosarcoma
Gastric
Astrocytes from fetal brain
Dendritic
Oesophagus
Time (h)
1 <= T(h) <= 2
2 < T(h) <= 4
4 < T(h) <=8
8 < T(h) <= 24
T(h) > 24
O2
0 <= O2(%) <= 0.5
0.5 < O2(%) <= 1
1 < O2(%) <= 2
2 < O2(%) <= 6
18 <= O2(%) <= 21
Tardon *
Ye *
Lombardi *
Toustrup *
Yang 2018 Sarcoma * °
Khouzam *
Mense *
Lendhal *
Zhang 2021 *
Sung *
Sorensen *
Santamaria * °
Wang 2005 *
Benita *
Chen *
Chi *
Aprelikova *
Elvidge *
Fardin 2009 *
Beyer *
Denko *
Eustace °
Lin °
Lane *
Boidot Cont. * °
Buffa * °
Detwiller *
Jogi *
Starmans *
Zhao °
Halle * °
Hu °
Bosco *
Manalo *
Xia °
Fardin 2010 *
Wei °
Ghazoui °
Li °
Winter °
Yang 2017 °
Fjeldbo °
Mo °
Wang 2020 °
Yang 2018 Prostate * °
Koong *
Ning *
Suh *
Ragnum * °
Van Malenstein *
Peters *
Zou °
Sun 2021 * °
Seigneuric E2 *
Ghorbel °
Shi *
He °
Seigneuric E0 *
Boidot Cyc. * °
Shou 2021 °
Seigneuric C. *
Xu * °
Gao °
Wang 2022 °
Shi 2021 °
Shou 2020 °
Trong *
Sun 2020 °
Liu °
Zhang 2020 * °
Cell type
Experimental condition
Tissue type
Time (h)
O2

Supplementary Figure S13: Complex heatmaps (Z-score)

Complex heatmap of signature p-values in hypoxic vs. non-hypoxic samples across GEO cell-line conditions in bulk RNAseq and microarray data, related to Figure 3. Conventions as in S2.

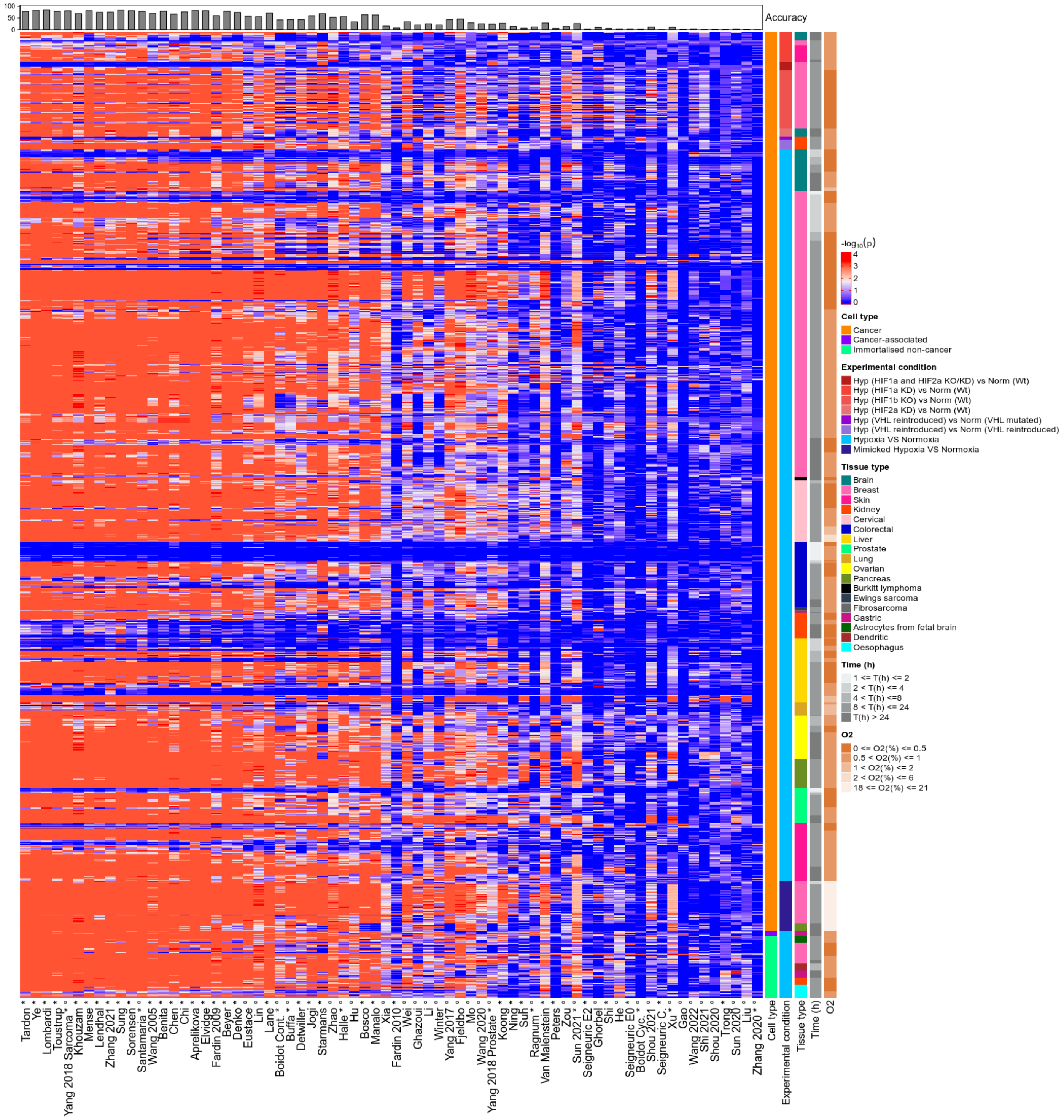

Accuracy
100
50
0
-log10(p)
4
3
2
1
0
Cell type
Cancer
Cancer-associated
Immortalised non-cancer
Experimental condition
Hyp (HIF1a and HIF2a KO/KD) vs Norm (Wt)
Hyp (HIF1a KD) vs Norm (Wt)
Hyp (HIF1b KO) vs Norm (Wt)
Hyp (HIF2a KD) vs Norm (Wt)
Hyp (VHL reintroduced) vs Norm (VHL mutated)
Hyp (VHL reintroduced) vs Norm (VHL reintroduced)
Hypoxia VS Normoxia
Mimicked Hypoxia VS Normoxia
Tissue type
Brain
Breast
Skin
Kidney
Cervical
Colorectal
Liver
Prostate
Lung
Ovarian
Pancreas
Burkitt lymphoma
Ewings sarcoma
Fibrosarcoma
Gastric
Astrocytes from fetal brain
Dendritic
Oesophagus
Time (h)
1 <= T(h) <= 2
2 < T(h) <= 4
4 < T(h) <=8
8 < T(h) <= 24
T(h) > 24
O2
0 <= O2(%) <= 0.5
0.5 < O2(%) <= 1
1 < O2(%) <= 2
2 < O2(%) <= 6
18 <= O2(%) <= 21
Tardon *
Ye *
Lombardi *
Toustrup *
Yang 2018 Sarcoma *
Khouzam *
Mense *
Lendhal *
Zhang 2021 *
Sung *
Sorensen *
Santamaria *
Wang 2005 *
Benita *
Chen *
Chi *
Aprelikova *
Elvidge *
Fardin 2009 *
Beyer *
Denko *
Eustace
Lin
Lane *
Boidot Cort. *
Buffa *
Detwiller *
Jogi *
Starmans
Zhao *
Halle
Hu
Bosco *
Manalo
Xia
Fardin 2010
Wei
Ghazoui
Li
Winter
Yang 2017
Fjeldbo
Mo
Wang 2020
Yang 2018 Prostate *
Koong *
Ning *
Suh *
Ragnum *
Van Malenstein
Peters *
Zou
Sun 2021 *
Seigneuric E2 *
Ghorbel
Shi *
He
Seigneuric E0
Boidot Cyc. *
Shou 2021
Seigneuric C. *
Xu *
Gao
Wang 2022
Shi 2021
Shou 2020
Trong *
Sun 2020
Liu
Zhang 2020 *
Cell type
Experimental condition
Tissue type
Time (h)
O2

Supplementary Figure S14: Complex heatmap (NCS)

Complex heatmap of signature p-values in hypoxic vs. non-hypoxic samples across GEO cell-line conditions in bulk RNAseq and microarray data, related to Figure 3. Conventions as in S2.

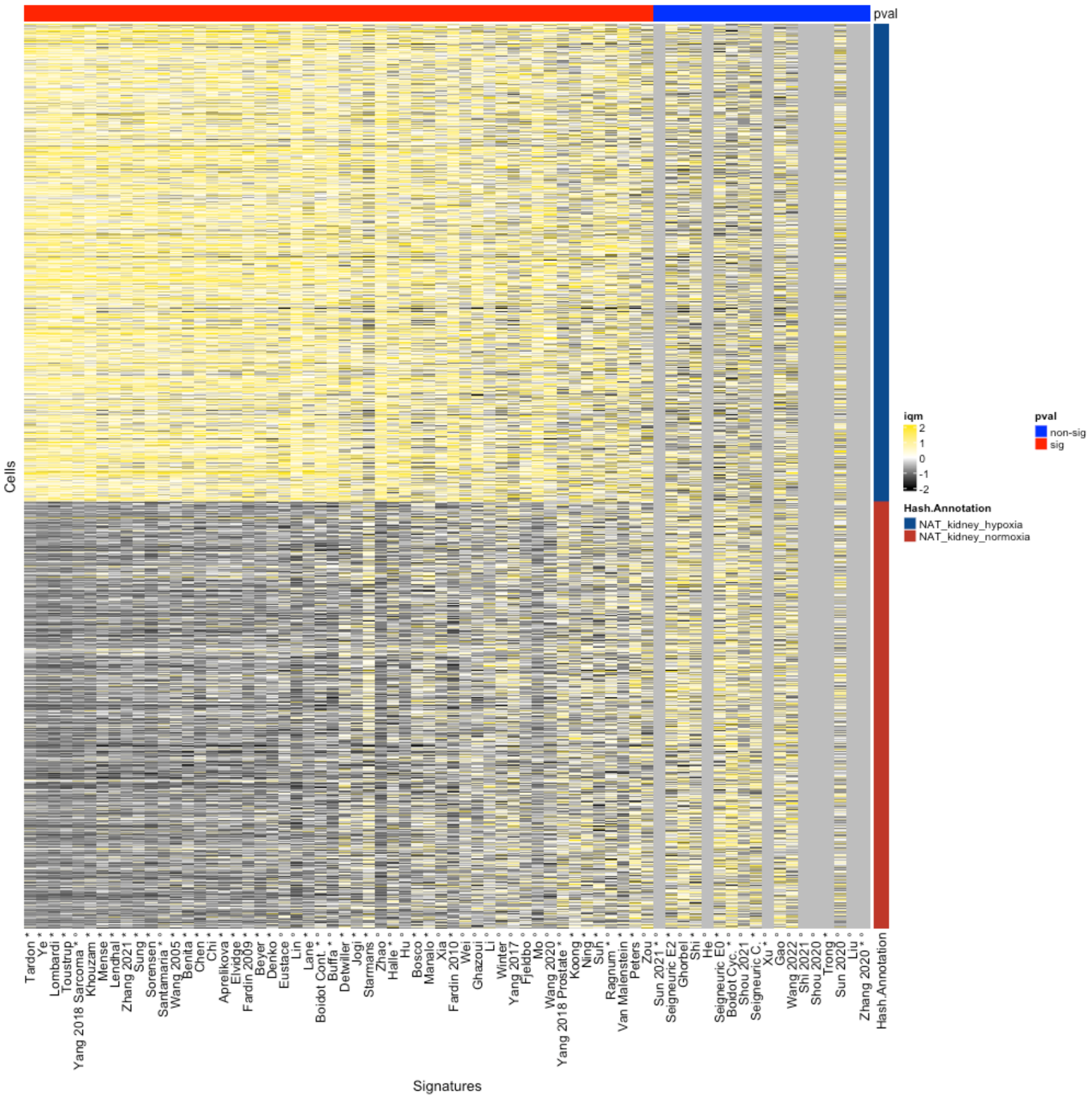

pval
Cells
Signatures
iqm
2
1
0
-1
-2
pval
non-sig
sig
Hash.Annotation
NAT_kidney_hypoxia
NAT_kidney_normoxia
Tardon *
Ye *
Lombardi *
Toustrup *
Yang 2018 Sarcoma * °
Khouzam *
Mense *
Lendhal *
Zhang 2021 *
Sung *
Sorensen *
Santamaria * °
Wang 2005 *
Benita *
Chen *
Chi *
Aprelikova *
Elvidge *
Fardin 2009 *
Beyer *
Denko *
Eustace °
Lin °
Lane *
Boidot Cont. *
Buffa *
Detwiller *
Jogi *
Starmans *
Zhao °
Halle *
Hu °
Bosco *
Manalo *
Xia *
Fardin 2010 *
Wei °
Ghazoui °
Li °
Winter °
Yang 2017 °
Fjeldbo °
Mo °
Wang 2020 °
Yang 2018 Prostate *
Koong *
Ning *
Sun *
Ragnum *
Van Malenstein *
Peters *
Zou °
Sun 2021 *
Seigneuric E2 *
Ghorbel °
Shi *
He °
Seigneuric E0 *
Boidot Cyc. *
Shou 2021 °
Seigneuric C. *
Xu *
Gao °
Wang 2022 °
Shi 2021 °
Shou 2020 °
Trong *
Sun 2020 °
Liu °
Zhang 2020 * °
Hash.Annotation

Supplementary Figure S15: Performance of the Tardon signature using the IQM in scRNA-Seq data for NAT human kidney (GSE200207) under normoxic and hypoxic conditions

Complex heatmap of IQM hypoxia score for hypoxic vs. normoxic cells in Normal Adjacent Tissue (NAT) from human kidney (GSE200207). Signatures on the x-axis are ordered based on their accuracy in the IQM score from Figure 3 for ease of comparison, related to Figure 4. The heatmap displays ranked-normalised hypoxia score values for each signature.

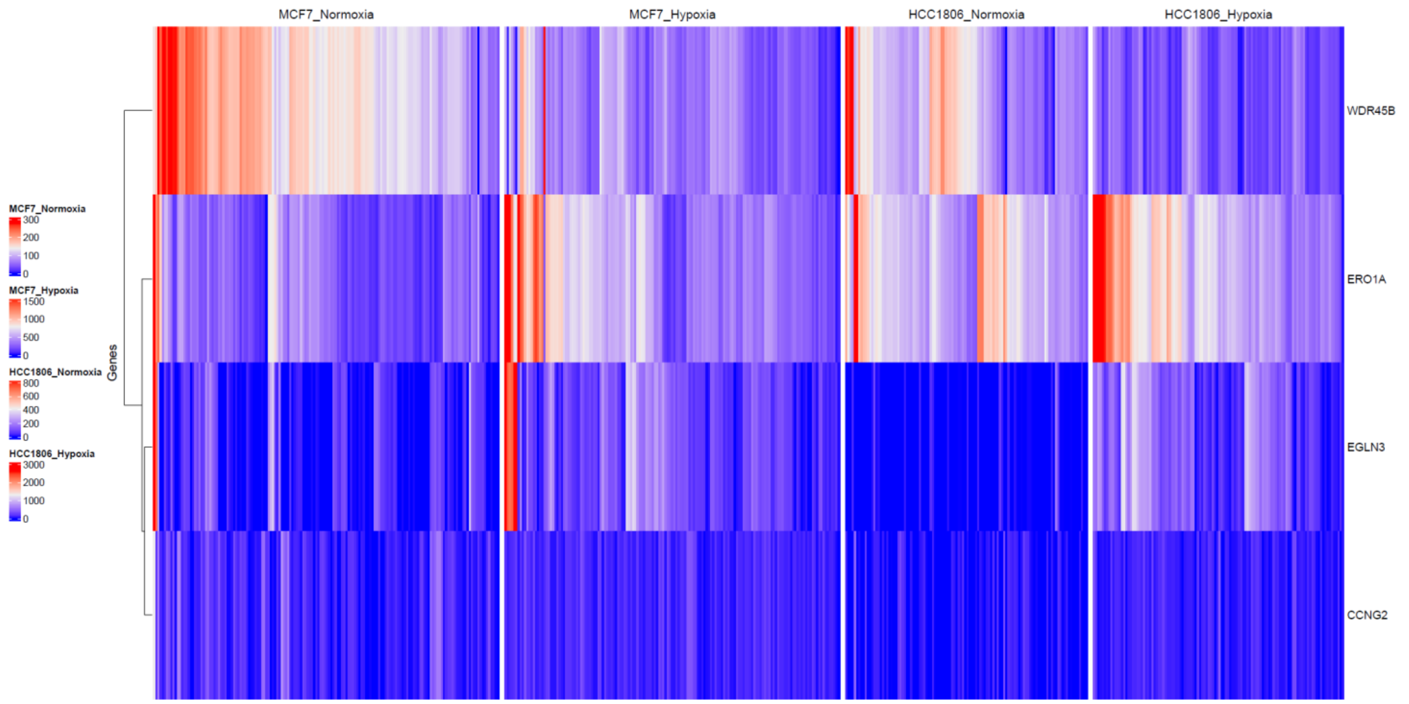
MCF7_Normoxia
MCF7_Hypoxia
HCC1806_Normoxia
HCC1806_Hypoxia
WDR45B
ERO1A
EGLN3
CCNG2
Genes
MCF7_Normoxia
300
200
100
0
MCF7_Hypoxia
1500
1000
500
0
HCC1806_Normoxia
800
600
400
200
0
HCC1806_Hypoxia
3000
2000
1000
0

Supplementary Figure S16: van Malenstein expression heatmaps clearly show dominance of ERO1A and WDR45B

Heatmaps depicting expression levels of van Malestein features in normoxic and hypoxic subsets from MCF7 and HCC1806 single-cell datasets. Rows represent signature features, columns represent individual cells, and colour intensities indicate library size-adjusted expression values. Notably, ERO1A exhibits elevated expression in hypoxia and reduced in normoxia, while WDR45B displays the inverse. This contrasting expression pattern leads to a diminished intra-signature correlation, affecting the signature's performance in single-cell data, related to Figure 4.

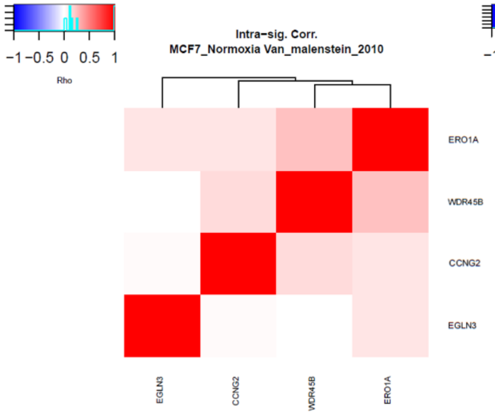
Intra−sig. Corr.
MCF7_Normoxia Van_malenstein_2010
−1 −0.5 0 0.5 1
Rho
ERO1A
WDR45B
CCNG2
EGLN3

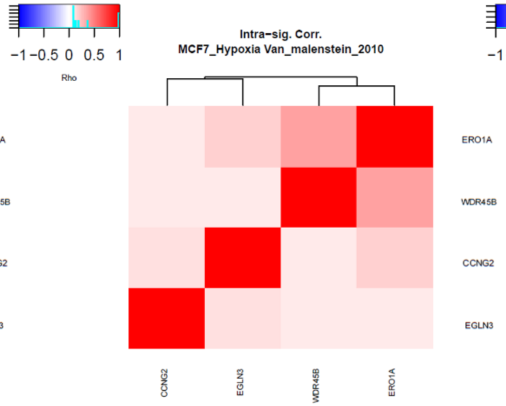
Intra−sig. Corr.
MCF7_Hypoxia Van_malenstein_2010
−1 −0.5 0 0.5 1
Rho
ERO1A
WDR45B
CCNG2
EGLN3

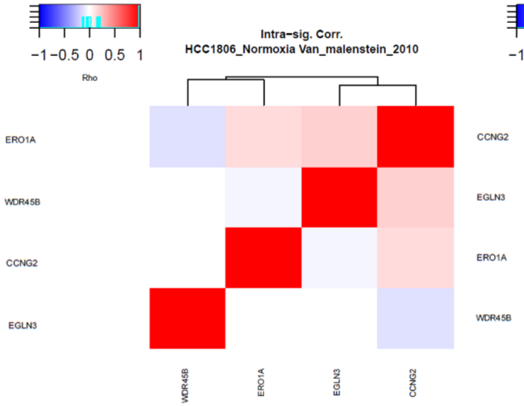
Intra−sig. Corr.
HCC1806_Normoxia Van_malenstein_2010
−1 −0.5 0 0.5 1
Rho
CCNG2
EGLN3
ERO1A
WDR45B

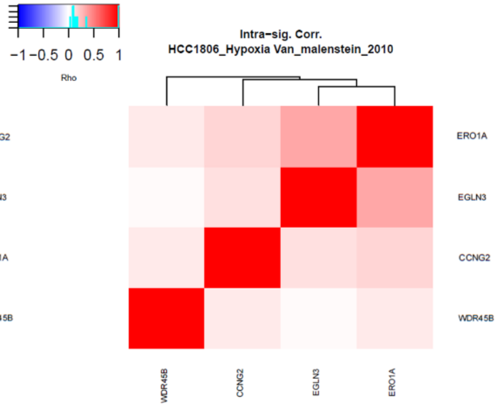
Intra−sig. Corr.
HCC1806_Hypoxia Van_malenstein_2010
−1 −0.5 0 0.5 1
Rho
ERO1A
EGLN3
CCNG2
WDR45B

Supplementary Figure S17: Poor intra-signature correlation is the primary driver of aberrant signature performance in single-cell data.

Heatmaps displaying intra-signature auto-correlation for normoxic and hypoxic subsets from MCF7 and HCC1806 single-cell datasets. Both rows and columns represent van Malestein signature features, while colour intensities indicate correlation levels. The evident weak intra-signature correlation greatly influences the disparity in signature performance between bulk and single-cell datasets, related to Figure 4.

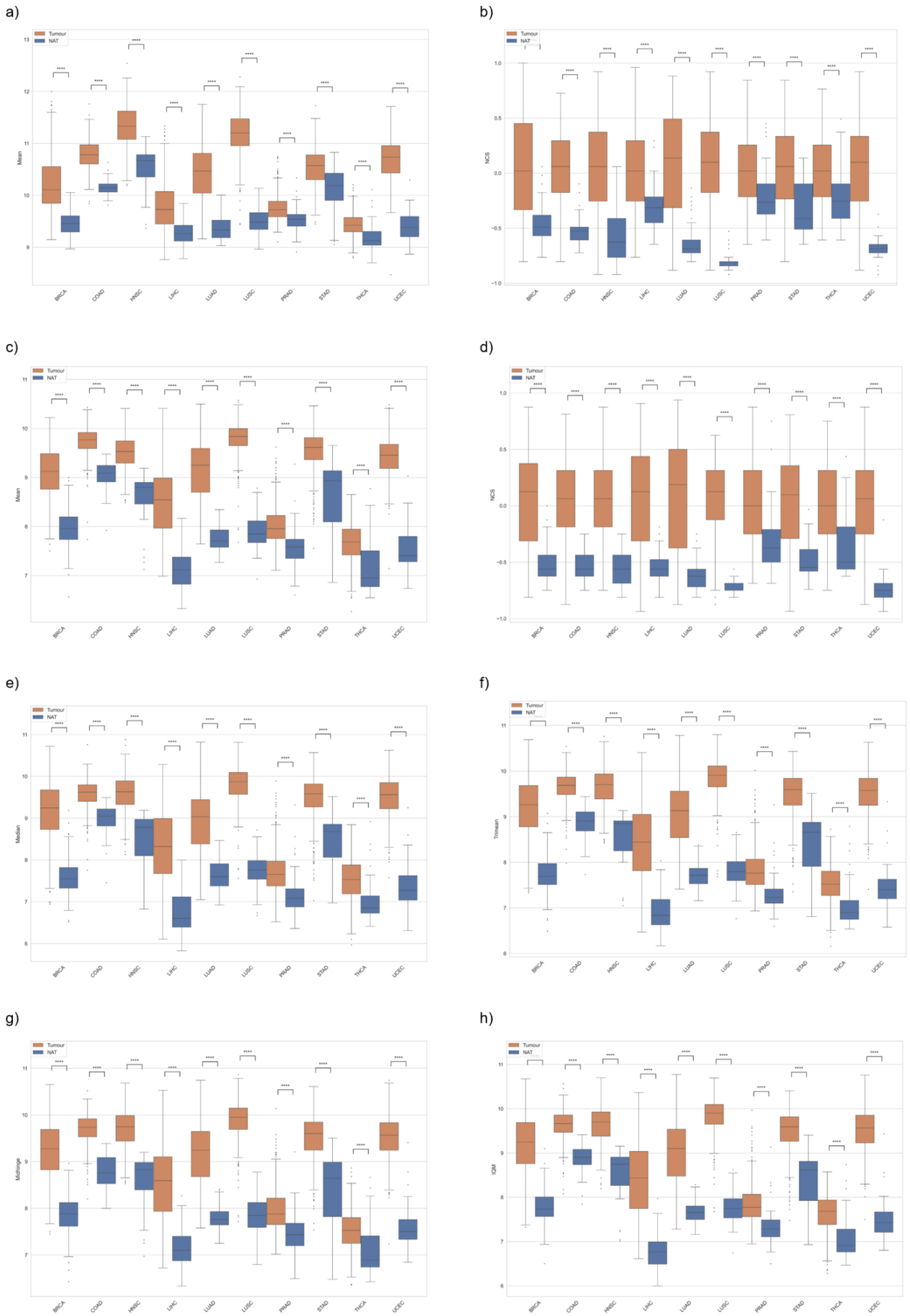

a)
b)
c)
d)
e)
f)
g)
h)
Tumour
NAT
Mean
NCS
Median
Trimean
Midhinge
IQM
BRCA
COAD
HNSC
LIHC
LUAD
LUSC
PRAD
STAD
THCA
UCEC

Supplementary Fig. S18: Two signatures, Buffa and Ragnum, showed higher scores in tumours compared to NAT across all 10 cancer types

Boxplots depicting hypoxia score differences in Buffa and Ragnum hypoxia signatures in 10 tumour types and normal adjacent tissue (NAT) in the The Cancer Genome Atlas (TCGA), related to Figure 5. Two signatures, Buffa and Ragnum, showed higher scores in tumours compared to NAT across all 10 cancer types using six scoring metrics. These results are shown in the eight panels, with orange denoting tumours and blue NAT: a) Buffa mean, b) Buffa NCS, c) Ragnum mean, d) Ragnum NCS, e) Ragnum median, f) Ragnum trimean, g) Ragnum midhinge, h) Ragnum NCS. The p-values were calculated using the Mann-Whitney-Wilcoxon test (****, $p <= 1.00E-04$).

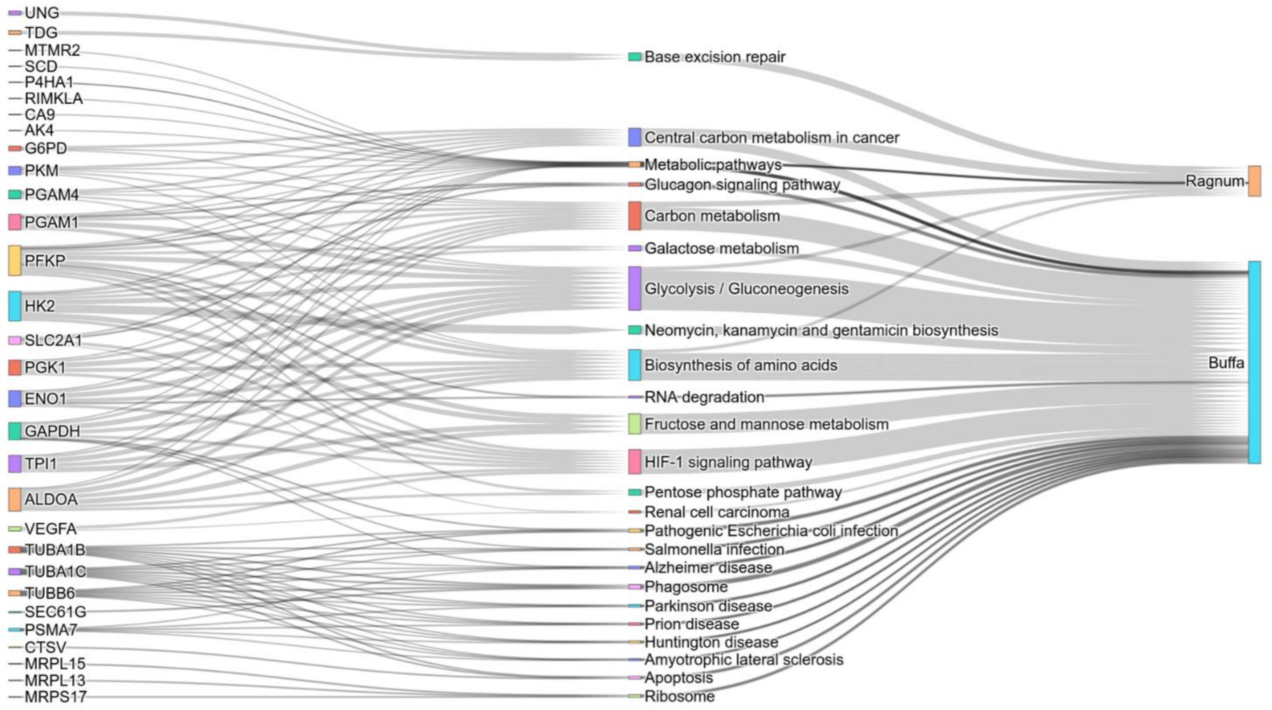
UNG
TDG
MTMR2
SCD
P4HA1
RIMKLA
CA9
AK4
G6PD
PKM
PGAM4
PGAM1
PFKP
HK2
SLC2A1
PGK1
ENO1
GAPDH
TPI1
ALDOA
VEGFA
TUBA1B
TUBA1C
TUBB6
SEC61G
PSMA7
CTSV
MRPL15
MRPL13
MRPS17
Base excision repair
Central carbon metabolism in cancer
Metabolic pathways
Glucagon signaling pathway
Carbon metabolism
Galactose metabolism
Glycolysis / Gluconeogenesis
Neomycin, kanamycin and gentamicin biosynthesis
Biosynthesis of amino acids
RNA degradation
Fructose and mannose metabolism
HIF-1 signaling pathway
Pentose phosphate pathway
Renal cell carcinoma
Pathogenic Escherichia coli infection
Salmonella infection
Alzheimer disease
Phagosome
Parkinson disease
Prion disease
Huntington disease
Amyotrophic lateral sclerosis
Apoptosis
Ribosome
Ragnum
Buffa

Supplementary Fig. S19: Gene-pathway associations in the Buffa and Ragnum hypoxia signatures

The Sankey diagram delineates the relationships between individual genes from Buffa and Ragnum hypoxia signatures and the biological pathways they are involved in. Each gene is linked to one or more pathways, denoting its functional role within the cellular processes. The pathways, in turn, converge into the hypoxia signatures of Buffa or Ragnum, highlighting the contribution of each pathway to the respective signature. The width of the bands reflects the number of genes shared between the entities, emphasising the significance of each gene and pathway to the hypoxia signatures' composition. The diagram reveals a significant enrichment of both signatures in pathways such as glycolysis/gluconeogenesis, central carbon metabolism in cancer, and broader carbon metabolism processes, related to Figure 5.

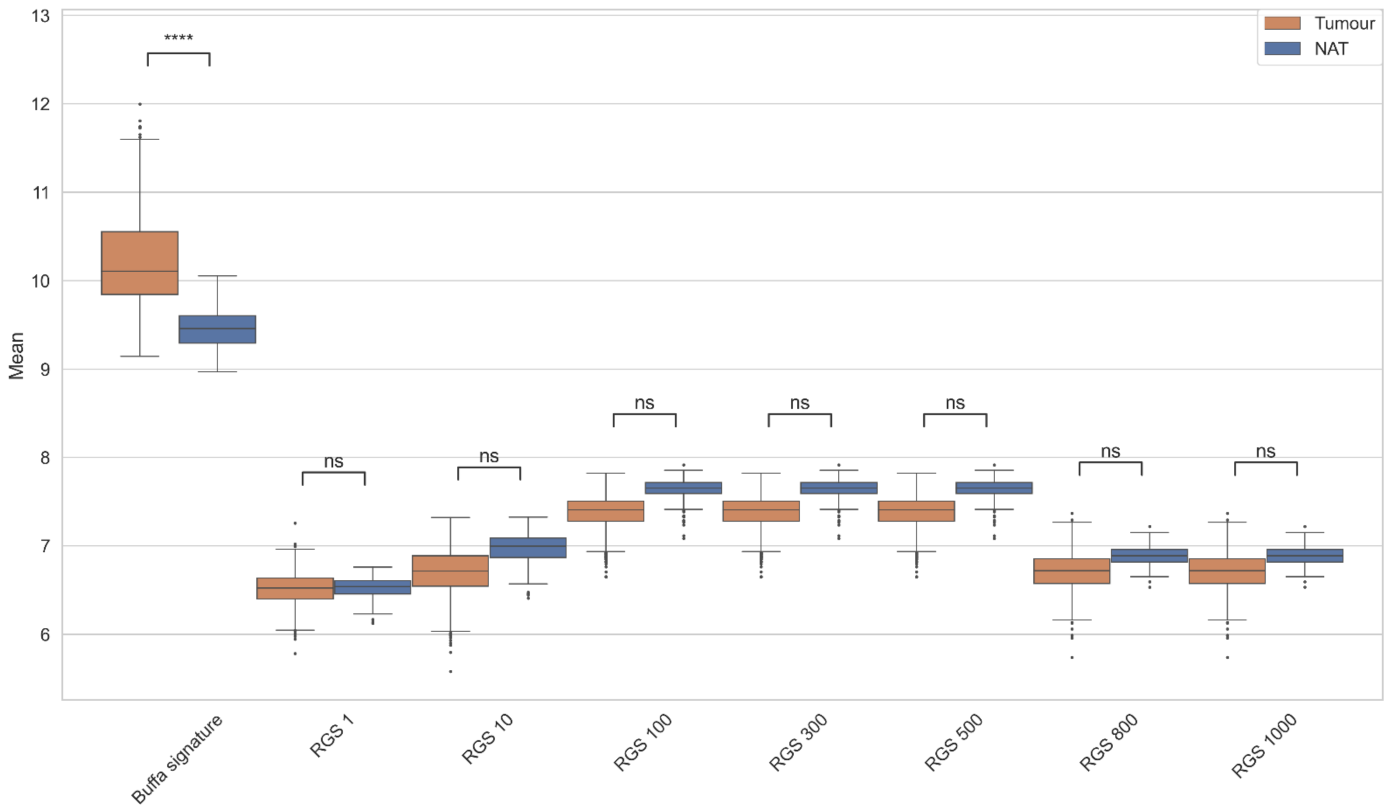

Tumour
NAT
Mean
13
12
11
10
9
8
7
6
****
ns
ns
ns
ns
ns
ns
ns
Buffa signature
RGS 1
RGS 10
RGS 100
RGS 300
RGS 500
RGS 800
RGS 1000

Supplementary Fig. S20: Hypoxia scores differences between hypoxia signatures and random gene sets in clinical samples

Boxplots depict the differences in hypoxia score distributions between Buffa signature and RGS using mean score in the BRCA cohort in TGCA. Hypoxia scores from RGS in tumour samples vs. normal adjacent tissue (NAT) show a lower score and a non-significant p-value as opposed to the original signature. The p-values were calculated using the Mann-Whitney-Wilcoxon test (****, $p <= 1.00E-04$), related to Figure 5.

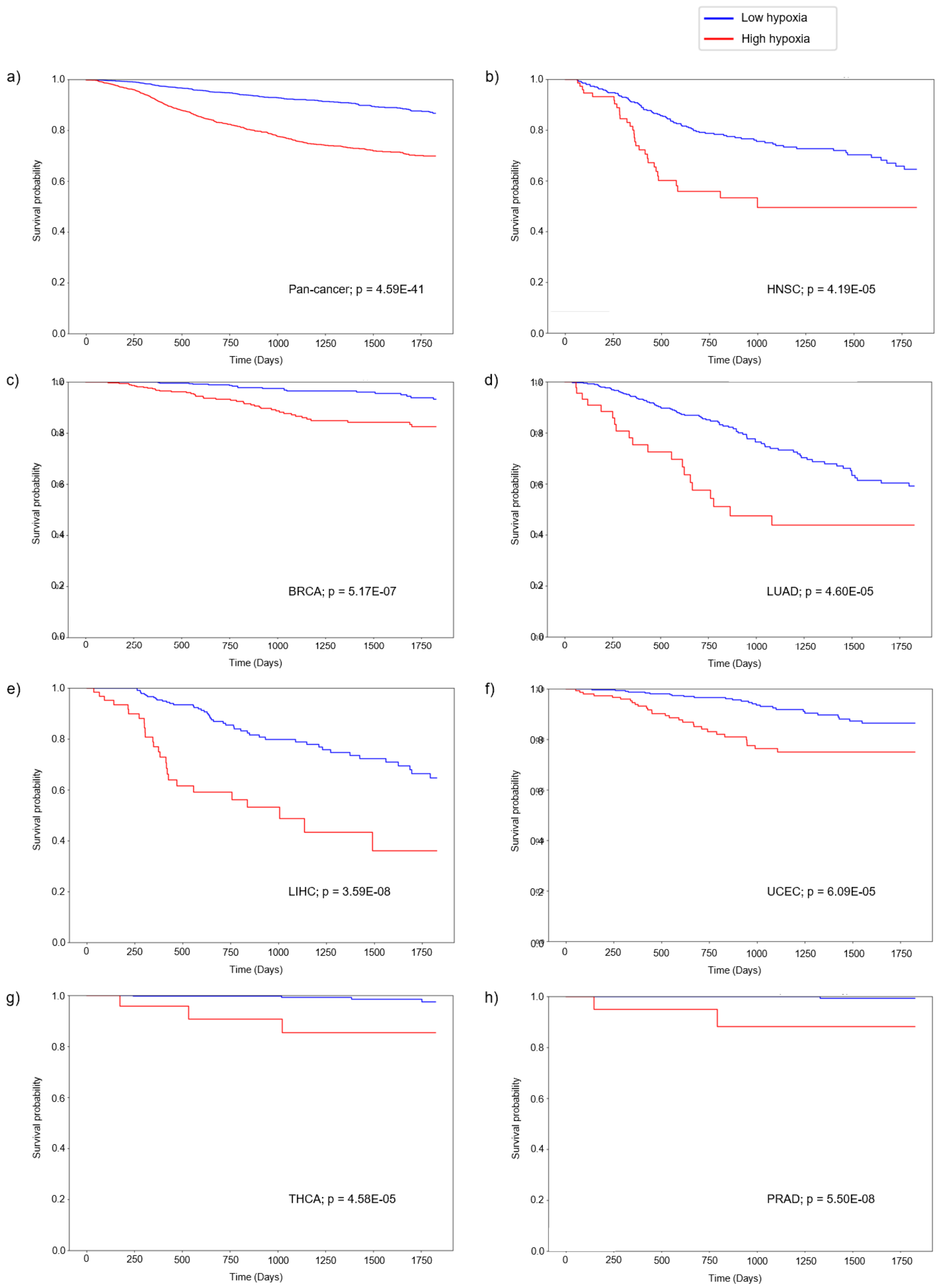
Low hypoxia
High hypoxia
a)
Pan-cancer; p = 4.59E-41
b)
HNSC; p = 4.19E-05
c)
BRCA; p = 5.17E-07
d)
LUAD; p = 4.60E-05
e)
LIHC; p = 3.59E-08
f)
UCEC; p = 6.09E-05
g)
THCA; p = 4.58E-05
h)
PRAD; p = 5.50E-08
Survival probability
Time (Days)

Supplementary Fig. S21: Buffa/mean shows prognostic efficacy across the TCGA

Kaplan–Meier survival estimates using the Buffa hypoxia signature and the mean score were investigated across pan-cancer and across ten individual cancer types. A point of contention in the field is what type of thresholds should be used across tumour types to denote low and high hypoxic samples. Previous works have used above and below the median however this is not necessarily the most comprehensive approach. Thus, to be more thorough, the prognostic ability of Buffa/mean was examined every fifth percentile using disease specific survival (DSS). The cohorts are dichotomised into high (red) and low (blue) hypoxia groups according to the percentile that yields the most significant Log-rank p from the Kaplan-Meier survival analysis. Buffa/mean was significantly prognostic in 7/10 tumour types, including (b) HNSC [85th percentile], (c) BRCA [60th percentile], (d) LUAD [90th percentile], (e) LIHC [75th percentile], (f) UCEC [70th percentile], (g) THCA [95th percentile] and (h) PRAD [90th percentile]. Furthermore, Buffa/mean signature maintained its prognostic value in a combined pan-cancer dataset (a) [50th percentile], related to Figure 5.

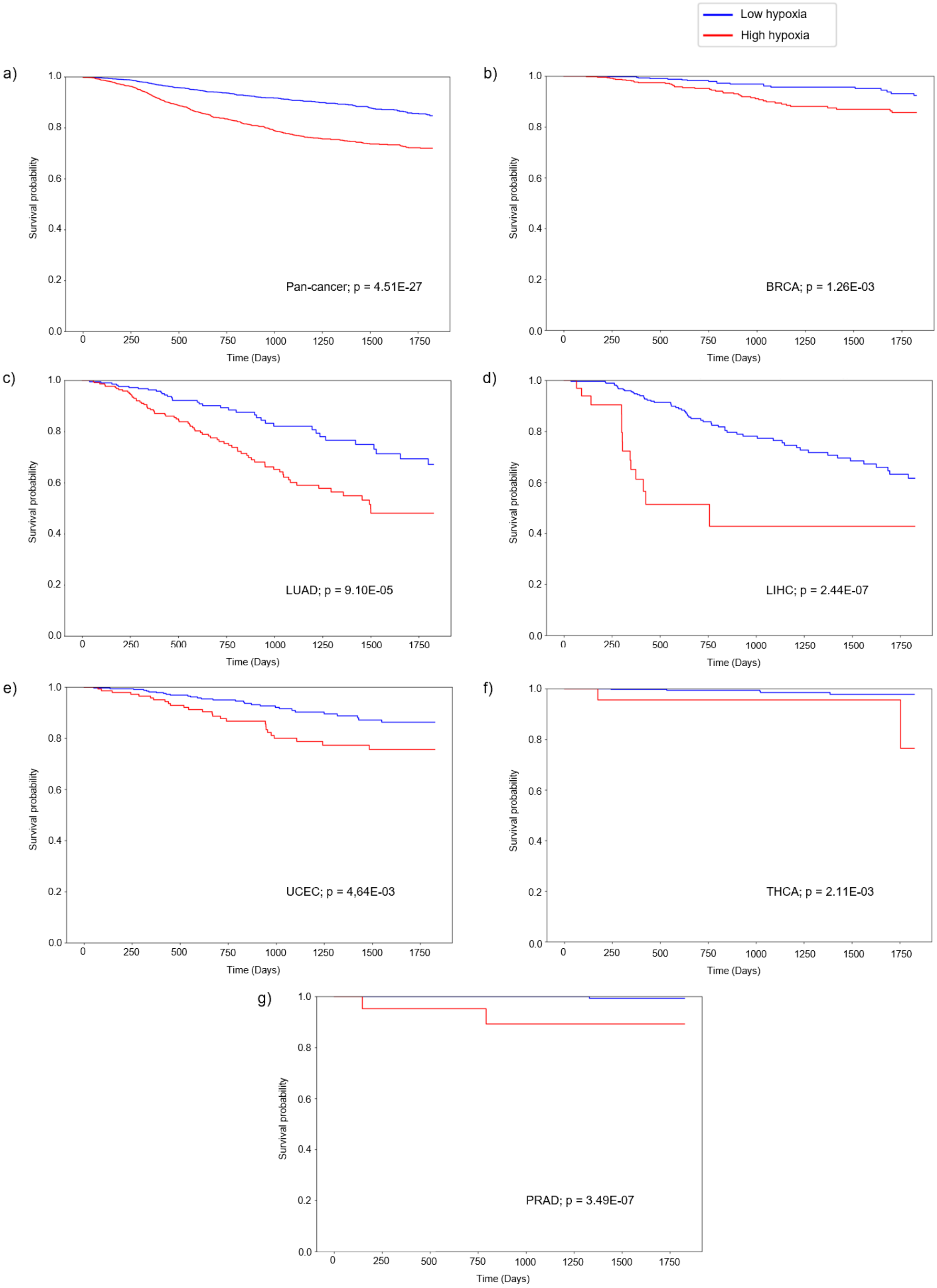

Low hypoxia
High hypoxia
a)
Pan-cancer; p = 4.51E-27
b)
BRCA; p = 1.26E-03
c)
LUAD; p = 9.10E-05
d)
LIHC; p = 2.44E-07
e)
UCEC; p = 4,64E-03
f)
THCA; p = 2.11E-03
g)
PRAD; p = 3.49E-07
Survival probability
Time (Days)
0.0
0.2
0.4
0.6
0.8
1.0
0
250
500
750
1000
1250
1500
1750

Supplementary Fig. S22: Ragnum/IQM shows prognostic efficacy across the TCGA

Kaplan–Meier survival estimates using the Ragnum hypoxia signature and the IQM score were investigated across pan-cancer and across ten individual cancer types across ten cancer types (conventions as in S47). Ragnum/IQM was significantly prognostic in 6/10 tumour types, including (b) BRCA [50th percentile], (c) LUAD [50th percentile], (d) LIHC [90th percentile], (e) UCEC [70th percentile], (f) THCA [50th percentile] and (g) PRAD [95th percentile]. Ragnum/IQM also maintained its prognostic value in a combined pan-cancer dataset (a) [50th percentile], related to Figure 5.

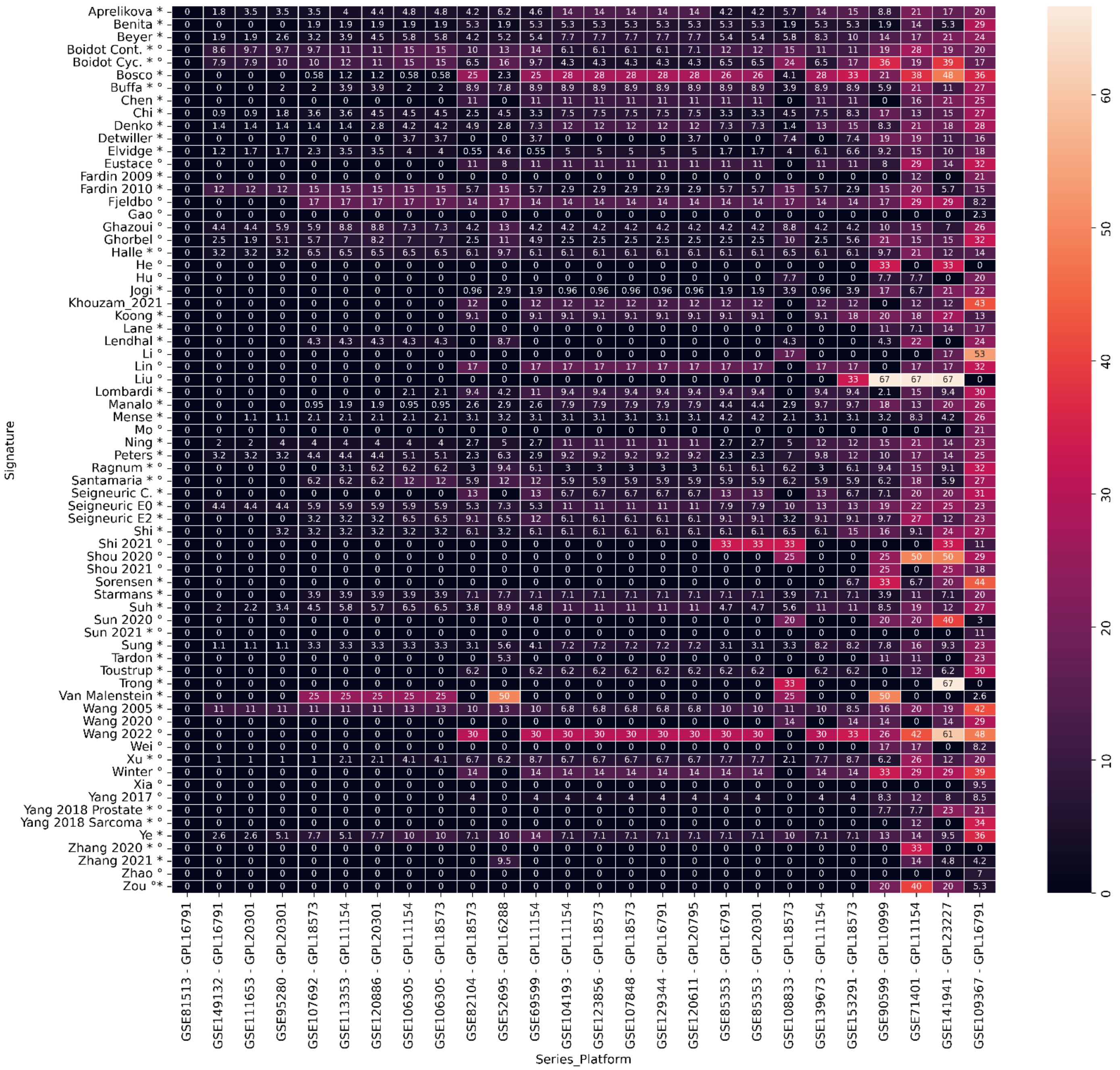
Signature
Aprelikova *
Benita *
Beyer *
Boidot Cont. * °
Boidot Cyc. * °
Bosco *
Buffa * °
Chen *
Chi *
Denko *
Detwiller *
Elvidge *
Eustace °
Fardin 2009 *
Fardin 2010 *
Fjeldbo °
Gao °
Ghazoui °
Ghorbel °
Halle * °
He °
Hu °
Jogi *
Khouzam_2021
Koong *
Lane *
Lendhal *
Li °
Lin °
Liu °
Lombardi *
Manalo *
Mense *
Mo °
Ning *
Peters *
Ragnum * °
Santamaria * °
Seigneuric C. *
Seigneuric E0 *
Seigneuric E2 *
Shi *
Shi 2021 °
Shou 2020 °
Shou 2021 °
Sorensen *
Starmans *
Suh *
Sun 2020 °
Sun 2021 * °
Sung *
Tardon *
Toustrup *
Trong *
Van Malenstein *
Wang 2005 *
Wang 2020 °
Wang 2022 °
Wei °
Xu * °
Winter °
Xia °
Yang 2017 °
Yang 2018 Prostate * °
Yang 2018 Sarcoma * °
Ye *
Zhang 2020 * °
Zhang 2021 *
Zhao °
Zou °*
Series_Platform
GSE81513 - GPL16791
GSE149132 - GPL16791
GSE111653 - GPL20301
GSE95280 - GPL20301
GSE107692 - GPL18573
GSE113353 - GPL11154
GSE120886 - GPL20301
GSE106305 - GPL11154
GSE106305 - GPL18573
GSE82104 - GPL18573
GSE52695 - GPL16288
GSE69599 - GPL11154
GSE104193 - GPL11154
GSE123856 - GPL18573
GSE107848 - GPL18573
GSE129344 - GPL16791
GSE120611 - GPL20795
GSE85353 - GPL16791
GSE85353 - GPL20301
GSE108833 - GPL18573
GSE139673 - GPL11154
GSE153291 - GPL18573
GSE90599 - GPL10999
GSE71401 - GPL11154
GSE141941 - GPL23227
GSE109367 - GPL16791
0
10
20
30
40
50
60

Supplementary Fig. S23: Percentage of genes missing in each signature in hypoxia-related bulk RNASeq experiments

Summary of the percentage of missing genes in each signature for all the bulk RNASeq platforms in GEO hypoxia experiments. Signature names are displayed on the y-axis, while GEO Platform (GPL) and Series (GSE) IDs are located on the x-axis. Lighter square colours indicate a higher percentage of missing genes for the respective signature, platform, and series. Individual missing-gene percentages for each signature and series are provided in the corresponding box, related to Table 1.

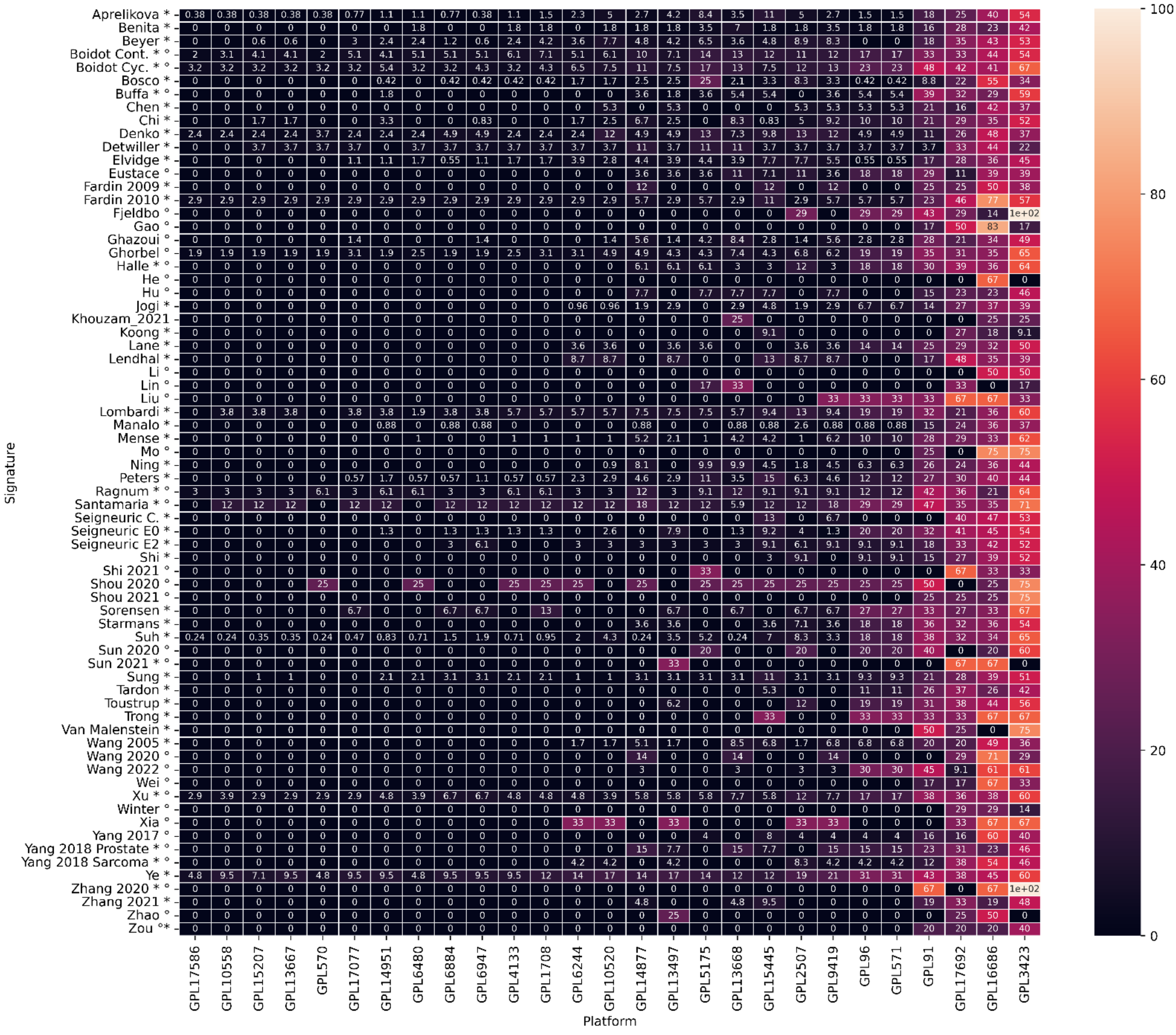

Aprelikova *
Benita *
Beyer *
Boidot Cont. * °
Boidot Cyc. * °
Bosco *
Buffa * °
Chen *
Chi *
Denko *
Detwiller *
Elvidge *
Eustace °
Fardin 2009 *
Fardin 2010 *
Fjeldbo °
Gao °
Ghazoui °
Ghorbel °
Halle * °
He °
Hu °
Jogi *
Khouzam_2021
Koong *
Lane *
Lendhal *
Li °
Lin °
Liu °
Lombardi *
Manalo *
Mense *
Mo °
Ning *
Peters *
Ragnum * °
Santamaria * °
Seigneuric C. *
Seigneuric E0 *
Seigneuric E2 *
Shi *
Shi 2021 °
Shou 2020 °
Shou 2021 °
Sorensen *
Starmans *
Suh *
Sun 2020 °
Sun 2021 * °
Sung *
Tardon *
Toustrup *
Trong *
Van Malenstein *
Wang 2005 *
Wang 2020 °
Wang 2022 °
Wei °
Xu * °
Winter °
Xia °
Yang 2017 °
Yang 2018 Prostate * °
Yang 2018 Sarcoma * °
Ye *
Zhang 2020 * °
Zhang 2021 *
Zhao °
Zou °*
Signature
GPL17586
GPL10558
GPL15207
GPL13667
GPL570
GPL17077
GPL14951
GPL6480
GPL6884
GPL6947
GPL4133
GPL1708
GPL6244
GPL10520
GPL14877
GPL13497
GPL5175
GPL13668
GPL15445
GPL2507
GPL9419
GPL96
GPL571
GPL91
GPL17692
GPL16686
GPL3423
Platform
100
80
60
40
20
0

Supplementary Fig. S24: Percentage of genes missing in each signature in hypoxia-related microarray experiments

Summary of percentage missing genes in each signature for all the microarray platforms in GEO hypoxia experiments. Signature names are displayed on the y-axis, while GEO Platform (GPL) IDs are located on the x-axis. Since microarray data for each platform share the same manifest, Series (GSE) IDs are not reported. Lighter square colours indicate a higher percentage of missing genes for the respective signature and platform. Individual missing-gene percentages for each signature and series are provided in the corresponding box, related to Table 1.

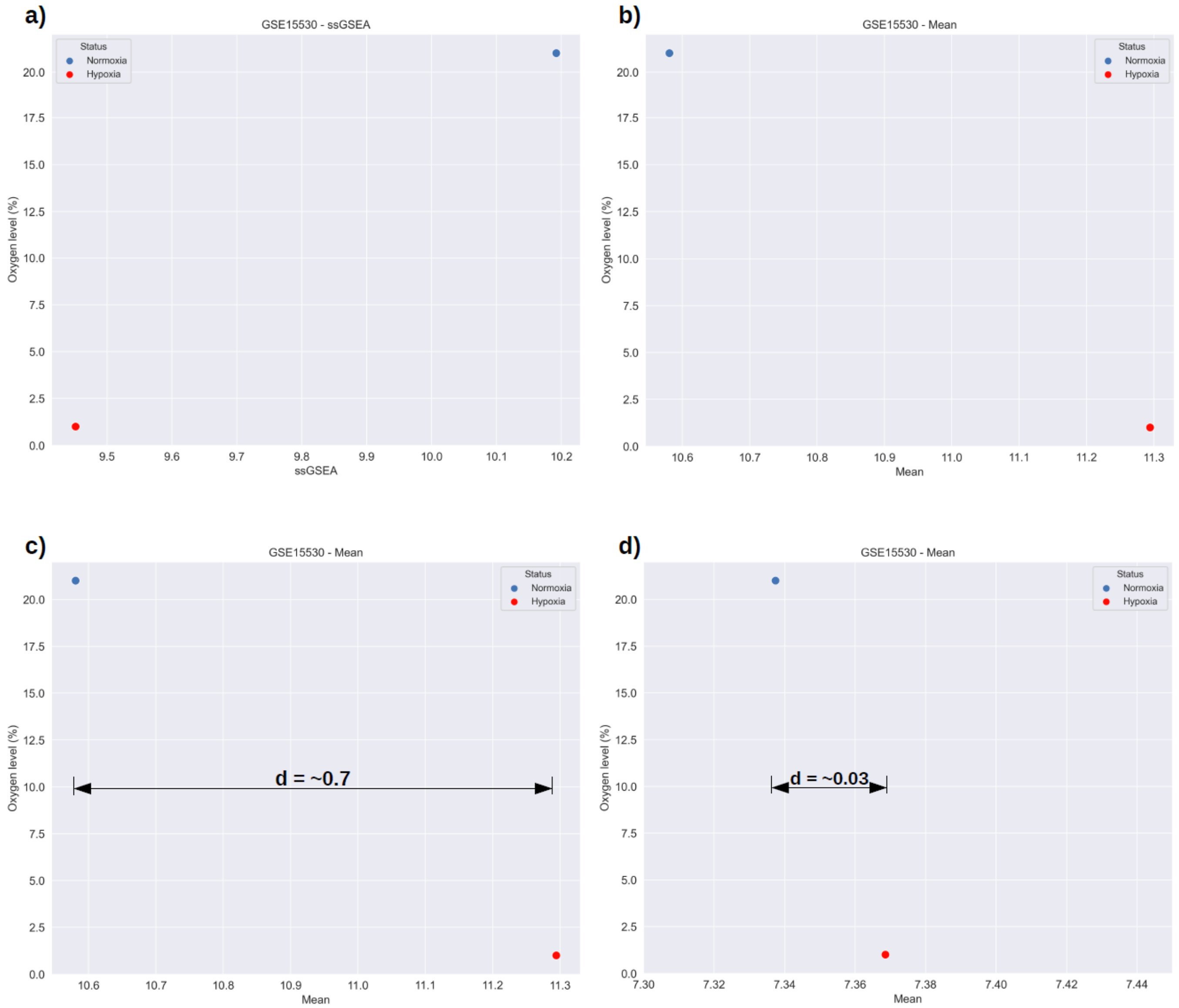
a)
GSE15530 - ssGSEA
Status
Normoxia
Hypoxia
Oxygen level (%)
ssGSEA
b)
GSE15530 - Mean
Status
Normoxia
Hypoxia
Oxygen level (%)
Mean
c)
GSE15530 - Mean
Status
Normoxia
Hypoxia
d = ~0.7
Oxygen level (%)
Mean
d)
GSE15530 - Mean
Status
Normoxia
Hypoxia
d = ~0.03
Oxygen level (%)
Mean

Supplementary Fig. S25: Hypoxia scores and distance calculation against RGS

The scatterplots were generated using Buffa signature on one normoxic (GSM390200, highlighted in blue) and one hypoxic (GSM390196, highlighted in red) sample from the Series GSE15530. In *a)* the mean and b) the ssGSEA scores are plotted on the x-axis against the percentage of oxygen. The mean score of the hypoxic sample is greater than the score of the normoxic sample. Viceversa, the ssGSEA score of the hypoxic sample is lower than the score of the normoxic sample, resulting in a wrong classification. Panel *c)* shows that the distance $d_{SIG}(hyp, norm)$ calculated using the mean score between the two samples is ~24x higher (left image, $d_{SIG} = \sim 0.7$) than the distance observed between the same samples using a RGS of the same length (Panel d, $d_{RGS} = \sim 0.03$), related to Figure 2.
