# Supplementary material for: Defining hypoxia in cancer: A landmark evaluation of hypoxia gene expression signatures
Source: Cell Genom. 2025 Jan 31;5(2):100764. doi: 10.1016/j.xgen.2025.100764 (PMC11872601; doi:10.1016/j.xgen.2025.100764)
Supplement: Document S2. Article plus supplemental information [file mmc12.pdf]

Cell Genomics

Article

Defining hypoxia in cancer: A landmark evaluation of hypoxia gene expression signatures

Graphical abstract

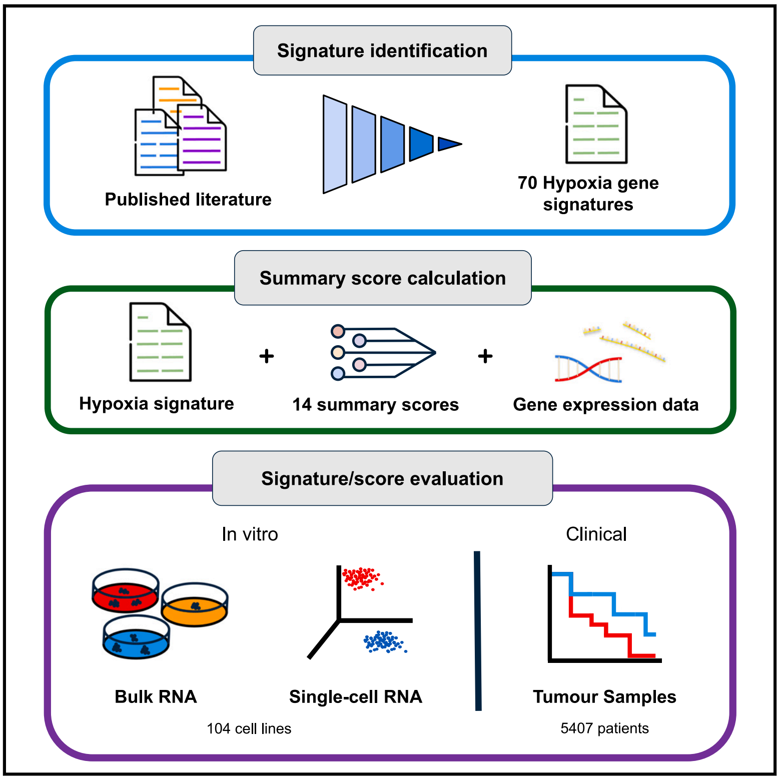

Authors

Matteo Di Giovannantonio, Fiona Hartley, Badran Elshenawy, ..., Adrian L. Harris, Francesca M. Buffa, Benjamin H.L. Harris

Correspondence

francesca.buffa@unibocconi.it (F.M.B.),
benjamin.harris@oncology.ox.ac.uk
(B.H.L.H.)

In brief

Di Giovannantonio et al. present a pan-cancer evaluation of 70 hypoxia signatures and 14 summary scores in 104 cell lines and 5,407 tumor samples using 472 million length-matched random gene signatures. The study concludes with recommendations aimed at enhancing experimental hypoxia detection and refining patient stratification for hypoxia-targeting therapies.

Highlights

- Challenges in tumor hypoxia quantification hinder therapeutic exploitation
- Hypoxia gene signatures have shown promise, but their optimal application is unclear
- Pan-cancer analysis shows that the choice of signature and score is key in hypoxia detection
- Guidance for signature and score use in laboratory and clinical contexts is provided

Di Giovannantonio et al., 2025, Cell Genomics *5*, 100764
February 12, 2025 © 2025 The Authors. Published by Elsevier Inc.
https://doi.org/10.1016/j.xgen.2025.100764

CellPress

Cell Genomics

CellPress
OPEN ACCESS

Article

Defining hypoxia in cancer: A landmark evaluation of hypoxia gene expression signatures

Matteo Di Giovannantonio,[1,11] Fiona Hartley,[1] Badran Elshenawy,[1] Alessandro Barberis,[1] Dan Hudson,[2,3] Hana S. Shafique,[4] Vincent E.S. Allott,[5] David A. Harris,[6] Simon R. Lord,[1] Syed Haider,[7] Adrian L. Harris,[1] Francesca M. Buffa,[1,8,9,12,*] and Benjamin H.L. Harris[1,5,10,11,12,13,*]

[1]Computational Biology and Integrative Genomics Lab, Department of Oncology, University of Oxford, Oxford, UK
[2]Chinese Academy of Medical Sciences Oxford Institute, University of Oxford, Oxford, UK
[3]The Rosalind Franklin Institute, Didcot, UK
[4]Duke University School of Medicine, Durham, NC, USA
[5]St. Catherine's College, University of Oxford, Oxford, UK
[6]Merton College, University of Oxford, Oxford, UK
[7]Breast Cancer Now Toby Robins Breast Cancer Research Centre, The Institute of Cancer Research, London, UK
[8]CompBio Lab, Department of Computing Sciences, Bocconi University, Milan, Italy
[9]AI and Systems Biology Lab, IFOM - Istituto Fondazione di Oncologia Molecolare ETS, Milan, Italy
[10]Cutrale Perioperative and Ageing Group, Imperial College London, London, UK
[11]These authors contributed equally
[12]Senior author
[13]Lead contact
*Correspondence: francesca.buffa@unibocconi.it (F.M.B.), benjamin.harris@oncology.ox.ac.uk (B.H.L.H.)
https://doi.org/10.1016/j.xgen.2025.100764

SUMMARY

Tumor hypoxia drives metabolic shifts, cancer progression, and therapeutic resistance. Challenges in quantifying hypoxia have hindered the exploitation of this potential "Achilles' heel." While gene expression signatures have shown promise as surrogate measures of hypoxia, signature usage is heterogeneous and debated. Here, we present a systematic pan-cancer evaluation of 70 hypoxia signatures and 14 summary scores in 104 cell lines and 5,407 tumor samples using 472 million length-matched random gene signatures. Signature and score choice strongly influenced the prediction of hypoxia *in vitro* and *in vivo*. In cell lines, the Tardon signature was highly accurate in both bulk and single-cell data (94% accuracy, interquartile mean). In tumors, the Buffa and Ragnum signatures demonstrated superior performance, with Buffa/mean and Ragnum/interquartile mean emerging as the most promising for prospective clinical trials. This work delivers recommendations for experimental hypoxia detection and patient stratification for hypoxia-targeting therapies, alongside a generalizable framework for signature evaluation.

INTRODUCTION

Hypoxia is a decrease in the normal level of oxygen in tissues, commonly observed in vascular and pulmonary diseases, as well as cancer.[1] Hypoxia emerges in the tumor microenvironment as cancer cells proliferate and aberrant angiogenesis fails to keep pace with increased oxygen demand.[2] This environment exerts a strong selective pressure, favoring adaptations associated with resistance to chemotherapy and radiotherapy,[3,4] increased genomic instability,[5] protection from antitumor immune responses,[6] creation of protective stem cell niches,[7] and enhanced metastatic potential.[8] Tumor hypoxia is therefore associated with poor prognosis in tumors across tissues, including breast,[9] bladder,[10] brain,[11] gastric,[12] head and neck,[13] liver,[14] lung,[15] esophageal,[16] and prostate.[17]

Over several decades, cancer hypoxia has been the focus of research aiming at therapy. However, hypoxia-targeted therapies have had limited integration into medical practice. A rare exception is the hypoxic radiosensitizer, nimorazole, which has been made standard of care in Denmark following the seminal the Danish Head and Neck Cancer Group (DAHANCA) study.[18] However, other countries have not followed suit, awaiting results of further studies.

Overall, hypoxia-targeted therapies have produced inconsistent results in clinical trials.[19] For instance, the promising hypoxia-activated prodrug tirapazamine, although successful in multiple phase I and II trials,[20–29] failed to improve overall survival or progression-free survival in phase III in cervical and head and neck cancers.[30,31] Mixed results were seen in non-small cell lung cancer.[32,33]

The lack of stratification has been cited to be sufficient to account for the failure of phase III trials for hypoxia-activated prodrugs.[34] This effect is not likely just limited to these prodrugs. Indeed, the failure to accurately identify patients with hypoxic tumors, and the lack of integration of validated hypoxia biomarkers into clinical trials, has contributed to disappointing clinical trial results across the field.[35–39]

© 2025 The Authors. Published by Elsevier Inc.
This is an open access article under the CC BY license (http://creativecommons.org/licenses/by/4.0/).

Reliably and accurately identifying hypoxia in tumors at scale may hold the key to unlocking the potential of hypoxia-targeting therapies. The development of predictive tools for patient stratification has been described as the most crucial step to the successful integration of these treatments.[40] One promising approach is the use of hypoxia gene expression signatures (hypoxia signatures): sets of genes whose expression is altered in the context of hypoxic environments and that can be applied to quantify the response to hypoxia in tumor tissue.[41] As well as being used prospectively to select patients for hypoxia-modifying/targeting agents, hypoxia gene expression signatures can be used in retrospective datasets, opening up other data sources not specifically aimed at studying hypoxia.[42]

Hypoxia signatures have been developed using a variety of approaches.[41] At present, there is no consensus in the field on how hypoxia signatures should be applied (*in vitro* or *in vivo*). Previous studies have found heterogeneity both in gene content and signature performance.[41,43] A central problem that limits the applicability of gene signatures to newly generated independent datasets is the difficulty of summarizing the expression of a disparate set of genes as a robust and transferable score.[44] Thus, two principal challenges exist: selecting the correct signature and finding the most effective method to represent the gene expression within it.

Currently, there is no agreement on which signature to use in which context, nor a systematic evaluation of differential signature performance with different summarization methods (scores). This work intends to bridge this lacuna in the literature and address the pressing questions tied to the hypoxia signature application: (1) which are the most appropriate signatures and most effective scoring methods (e.g., median, mean, gene set variation analysis [GSVA]) for measurement of hypoxia in cell lines; (2) which are the most promising signatures for stratifying patients for treatment with hypoxia-targeting agents in clinical practice? If a single signature/score combination proves promising in one or both areas, it would provide substantial benefits to both laboratory and clinical researchers by improving consistency and streamlining research efforts.

Here, we systematically assess published hypoxia signatures and common scoring methods in the largest analysis to date. We investigate the performance of 70 hypoxia signatures in hypoxia vs. normoxia experiments across 104 cell lines, as well as in over 5,000 clinical samples from 10 solid tumor types. Since no consensus exists about how hypoxia signatures should be applied to the burgeoning field of single-cell data, we investigate signature effectiveness in this high-resolution technology. Furthermore, we present a novel approach to solving one of the key controversies emerging in the field of gene signatures as a whole, testing whether signatures truly differ in performance to random gene sets/signatures (RGSs).[45–47] This work provides much-needed clarity to the field and helps establish a new foundation in how to apply hypoxia signatures so we can enhance our understanding of tumor microenvironmental biology, elucidate new pathways and biomarkers, and, ultimately, drive effective patient stratification for hypoxia modifiers and other treatment solutions.

RESULTS

Systematic review reveals 70 published hypoxia signatures

A systematic search of the three major databases for academic publishing yielded 70 publications on hypoxia gene expression signatures (Figure 1A), 38 more than were identified in a 2015 study.[41] The size of these signatures ranged from 759 genes (Starmans[48]) to three genes (Sun,[49] Xu,[50] Zhang 2020[51]), and their makeup and annotations are given in Table S1 (S1a, symbol annotation; S1b, Entrez annotation; S1c, Ensembl annotation). Their mean and median signature sizes were 55 and 24 genes respectively. 35 signatures were derived using clinical samples, leaving 35 derived from *in vitro* approaches alone. Signatures derived using clinical samples ranged from three to 158 in length with a median number of 14 genes. *In vitro* signatures tended to be longer, with a median number of genes of 42. Surprisingly, no individual gene was found in all 70 signatures. This could reflect their origin in terms of the derived tissues' response to hypoxia (different cell lines/tumor types), or this might reflect differences in the experimental conditions used (percentage of oxygen, length of time under hypoxia, etc.; Table 1). The number of overlapping genes between signatures is shown in (Figure S1).

Nonetheless, several genes occurred frequently across signatures (top occurring genes highlighted in Figure 1B). The most frequently occurring genes across signatures were NDRG1 and VEGFA (both present in 28 out of the 70 signatures) and PGK1 (27 out of 70). Frequencies for all genes are found in Table S2.

Pathway enrichment analysis of all genes occurring in any of the 70 signatures confirmed several enriched pathways known to be activated in response to hypoxia (Figure 1C; Table S3). Reassuringly, the most enriched pathways were HIF-1 signaling (4.5-fold enrichment, $p = 3.18E-17$) and renal cell carcinoma (4-fold enrichment, $p = 5.65E-09$), the latter a disease where the HIF transcriptional response is active due to VHL inactivation by mutation.[118] Protein processing in the endoplasmic reticulum (ER) was also enriched (3.7-fold enrichment, $p = 3.18E-17$) driven by several genes known to be induced in response to ER stress (including ATF4 and ATF6), three members of the ER degradation-enhancing alpha-mannosidase-like protein family (EDEM1, EDEM2, and EDEM3) and genes coding for components of coat protein-complex II (SEC23Aand SEC24A). This might be linked to enrichment of autophagy (2.9-fold enrichment, $p = 1.56E-08$) as both ER stress and autophagy are known to be linked.[119] Several diseases previously associated with the expression of genes regulated in the hypoxic response were also found to be enriched, including diabetes, non-alcoholic liver disease, Parkinson's disease, and prion disease.[120–123]

Signature and summary score choices strongly influence predictive value for hypoxic exposure

Gene signatures can be informative of the status of a biological sample, from cell cultures to tissue. For example, if all the genes associated with the hypoxia response are highly expressed in one sample and not expressed in another sample, we would conclude that the first sample is experiencing a hypoxia response. However, this clear dichotomy is rarely observed, as transcriptional readouts are intrinsically noisy and tissue cultures

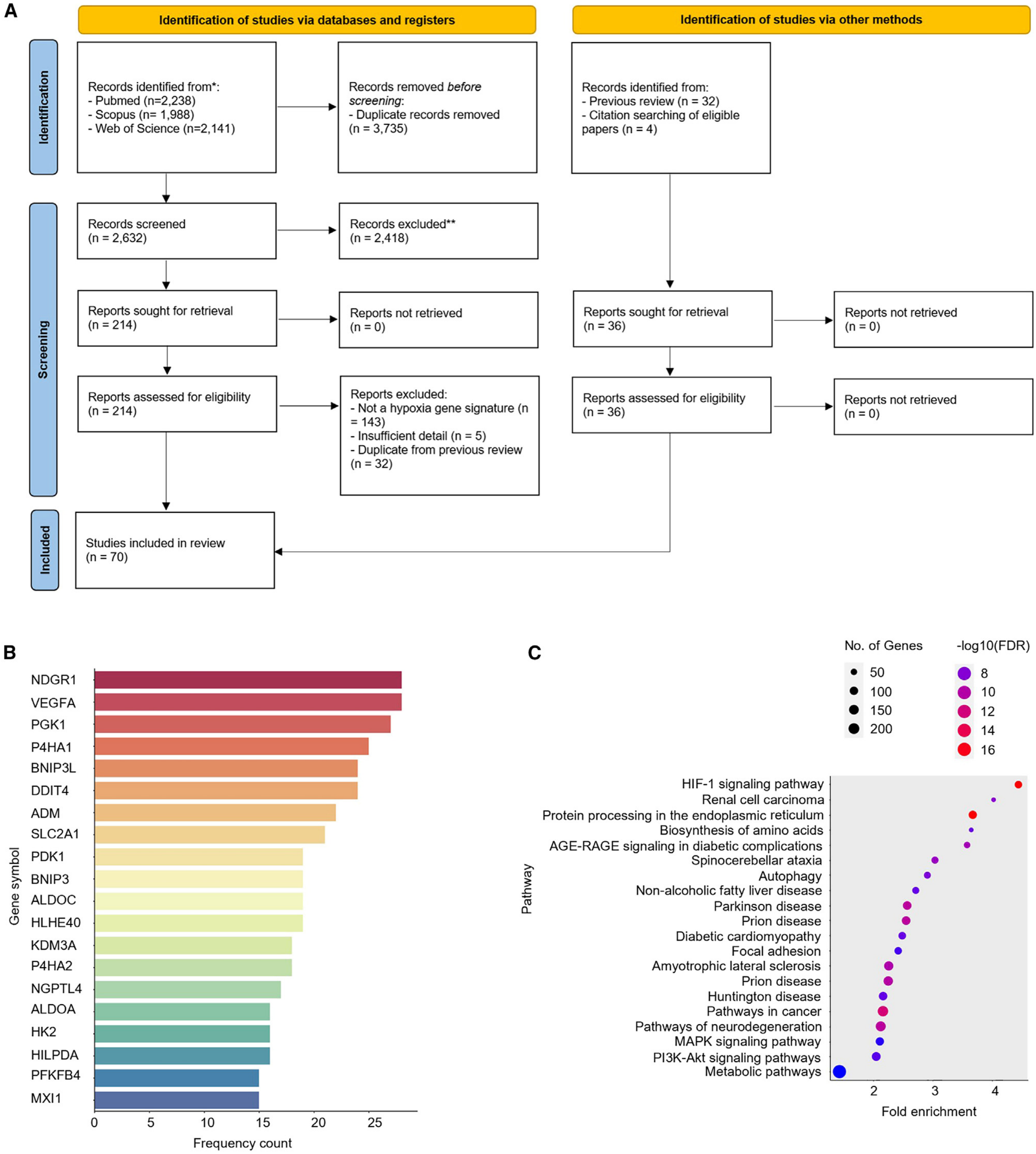

Figure 1. Identification of hypoxia gene expression signatures

(A) The approach taken in the systematic review. 70 hypoxia signatures were discovered across the interrogated databases (PubMed, Scopus, and Web of Science). Most frequently occurring genes across the 70 signatures are reported in (B) and pathways enriched across all signatures are reported in (C).

and tissues are not uniform. Nevertheless, we could expect that, overall, the expression of genes that are canonically regulated by hypoxia will tend to be higher in a sample cultured in hypoxia, with respect to a sample cultured in normoxia. Thus, hypoxia gene expression signatures need to be summarized optimally to infer a "hypoxic phenotype" in a biological system/clinical setting of interest. Similarly, for other phenotypes, gene signatures can be used as a proxy.

Table 1. Published hypoxia signatures assessed in this study

PubMed ID	Signature	Clinical/ *in vitro*	Cell line	Cell/tissue origin*	# Gene symbols	Final num. genes	Experimental conditions	Reference
10706099	Koong 2000	*in vitro*	FaDu, SiHa	pharynx (transformed keratinocytes), cervix (transformed keratinocytes)	10	10	0.05% O_2, 24 h	Koong et al.[52]
12947397	Denko 2003	*in vitro*	NCK, NDK, NCF, SiHa, C33a, FaDu	cervix (keratinocytes and stromal fibroblasts)	80 → 72	72	0.05% O_2, 24 h (FaDu, SiHa) 0.1% O_2, 16 h Detailed elsewhere[52–55]	Denko et al.[56]
15093745	Jögi 2004	*in vitro*	SK-N-BE(2), SH-SY5Y, SK-N-F1, IMR-32, LA-N-2, LA-N-5, SK-N-RA	brain (neuroblastoma)	107 → 103	103	1% O_2, 72 h	Jögi et al.[57]
15100389	Ning 2004	*in vitro*	HAECs	aortic endothelial cells	104 → 99	99	1% O_2, 8–24 h	Ning et al.[58]
15374877	Manalo 2005	*in vitro*	ECs	coronary artery endothelial cells	107 → 105	105	1% O_2, 24 h	Manalo et al.[59]
15833863	Wang 2005	*in vitro*	HEK293T	kidney	56 → 55	55	1% O_2, 16 h	Wang et al.[60]
15994966	Detwiller 2005	*in vitro*	HT1080, 10T1/2, SVR, HEK293	fibrosarcoma fibroblast (*Mus musculus*), Pancreas (*M. musculus*), kidney	27	27	1% O_2, 48 h	Detwiller et al.[61]
16417408	Chi 2006	*in vitro*	ECs, SMCs, HMECs, RPTECs	coronary artery endothelial cells, smooth muscle cells, mammalian epithelial cells, renal proximal tubule epithelial cells	111	111	0.1–2% O_2, 1–24 h	Chi et al.[62]
16507782	Mense 2006	*in vitro*	HFAs	fetal astrocytes	111 → 94	94	1% O_2, 24 h	Mense et al.[63]
16565084	Elvidge 2006	*in vitro*	MCF7	breast	181 → 173	173	1% O_2, 16 h DMOG, 16 h	Elvidge et al.[64]
16595741	Peters 2006	*in vitro*	HPAECs	pulmonary artery endothelial cells	159 → 158	158	1% O_2, 8–24 h	Peters et al.[65]
16740701	Aprelikova 2006	*in vitro*	MCF7	breast	236 → 230	230	0.5% O_2, 8 h	Aprelikova et al.[66]
16849508	Bosco 2006	*in vitro*	PBMC	peripheral blood monocytes	177 → 173	173	1% O_2, 16 h	Bosco et al.[67]
17187782	Shi 2007	*in vitro*	LX-2	hepatic stellate cells	32 → 31	31	1% O_2, 8-24 h	Shi et al.[68]
17320280	Sung 2007	*in vitro*	CNE-2, C666-1, HONE-1, HK1	head and neck (nasopharyngeal carcinoma)	90	90	0.1% O_2, 16 h	Sung et al.[69]
17409455	Winter 2007*	clinical	clinical samples	head and neck (squamous cell carcinoma)	99 → 97	97	–	Winter et al.[70]
17532074	Seigneuric 2007 (common)	*in vitro*	HMECs from Chi 2006	mammary epithelial cell	14	14	0%–2% O_2, 1–24 h	Seigneuric et al.[71]

(*Continued on next page*)

Table 1. *Continued*

PubMed ID	Signature	Clinical/ *in vitro*	Cell line	Cell/tissue origin*	# Gene symbols	Final num. genes	Experimental conditions	Reference
17532074	Seigneuric 2007 (early0)	*in vitro*	HMECs from Chi 2006	mammary epithelial cell	71 → 68	68	0%–0.02% O_2, 1–6 h	Seigneuric et al.[71]
17532074	Seigneuric 2007 (early2)	*in vitro*	HMECs from Chi 2006	mammary epithelial cell	34 → 31	31	2% O_2, 12–24 h	Seigneuric et al.[71]
18984585	Beyer 2008	*in vitro*	HeLa, HEK293, 786–0	cervix, kidney, renal cancer	159 → 158	158	0.2%–1%, 24 h	Beyer et al.[72]
19291283	Hu 2009*	clinical	clinical samples	breast	13	13	–	Hu et al.[73]
19491311	Benita 2009	*in vitro*	DLD-1, HCT116, SW480, Lovo Panc-1, HeLa, MCF7	colorectal, colon, pancreas, cervix, breast	57 → 54	54	1% O_2, 18 h	Benita et al.[74]
19832978	Fardin 2009	*in vitro*	GI-LI-N, ACN, GI-ME-N, IMR-32, LAN-1, SK-N-BE(2)C, SK-N-F1, SK-N-SH	brain (neuroblastoma)	8	8	1% O_2, 18 h	Fardin et al.[75]
19884889	Lendahl 2009	*in vitro*	HeLa, P493-6, HCT116, Hep3B, MCF7, RCC4, SK-N-BE(2)C, (E-MEXP-836)	cervix, Burkitt's lymphoma, colon, liver, breast, kidney (VHL mutated), brain (neuroblastoma)	23	23	different conditions	Lendahl et al.[76]
20087356	Buffa 2010*	both	HeLa, P493-6, HCT116, Hep3B, MCF7, RCC4, SK-N-BE(2)C, (E-MEXP-836)	head and neck (squamous cell carcinoma), breast	51	51	–	Buffa et al.[77]
20416888	Ghorbel 2010*	clinical	clinical samples	cyanotic tetralogy of Fallot	166 → 158	158	–	Ghorbel et al.[78]
20429727	Sørensen 2010	*in vitro*	SiHa, FaDuDD, UTSCC5, UTSCC14, UTSCC15	cervix head and neck (squamous cell carcinomas)	27 → 26	26	0%–5% O_2, 24 h	Sørensen et al.[79]
20592013	van Malenstein 2010	*in vitro*	HepG2	liver	4	4	2% O_2, 72 h	van Malenstein et al.[80]
20652058	Fardin 2010	*in vitro*	GI-LI-N, ACN, GI-ME-N, IMR-32, LAN-1, SK-N-BE(2)C, SK-N-F1, SK-N-SH	brain (neuroblastoma)	35	35	1% O_2, 18 h	Fardin et al.[81]
21325071	Ghazoui 2011*	clinical	clinical samples	breast	70 → 68	68	–	Ghazoui et al.[82]
21846821	Toustrup 2011	*in vitro*	UTSCC5, UTSCC14, UTSCC15, FaDu, SiHa	head and neck, cervix	15	15	O_2 < 2.5 mm Hg (electrode)	Toustrup et al.[83]
22356756	Starmans 2012	*in vitro*	DU145, HT29, MCF7,	prostate, colon, breast	759 → 756	756	0% O_2, 1–24 h	Starmans et al.[48]
22890239	Halle 2012*	both	HeLa, SiHa, CaSki, clinical samples	cervix	31	31	0.2%, 24 h	Halle et al.[84]
23820108	Eustace 2013*	clinical	clinical samples	laryngeal cancer, bladder cancer	26 → 25	25	–	Eustace et al.[85]

(*Continued on next page*)

Table 1. *Continued*

PubMed ID	Signature	Clinical/ *in vitro*	Cell line	Cell/tissue origin*	# Gene symbols	Final num. genes	Experimental conditions	Reference
25216520	Boidot 2014* (continuous hypoxia)	both	MCF-7, MDA-MB-231, T47D, A549, Widr, HCT116 WTP53, HCT116 −/−P53, HT29, Colo-205, LoVo, HCT15, SiHa, PC3, U373, HepG2, Hep3B, PLC/PRF/5, SK-HEP-1, A498, HT1080, clinical samples	breast, colon, prostate, colorectal, liver, fibrosarcoma	98 → 93 (∼50 based on heatmap)	93	1%, 24 h	Boidot et al.[86]
25216520	Boidot 2014* (cyclic hypoxia)	both	MCF-7, MDA-MB-231, T47D, A549, Widr, HCT116 WTP53, HCT116 −/−P53, HT29, Colo-205, LoVo, HCT15, SiHa, PC3, U373, HepG2, Hep3B, PLC/PRF/5, SK-HEP-1, A498, HT1080, clinical samples	breast, colon, prostate, colorectal, liver, fibrosarcoma	96 → 90 (∼50 based on heatmap)	90	cycling hypoxia, 30 min 1% O_2 + 30 min normoxia, 24 h	Boidot et al.[86]
25461803	Ragnum 2015*	both	22Rv1, LNCaP, PC-3, DU 145, clinical samples	prostate	32	32	0.2%, 24 h	Ragnum et al.[87]
27012812	Fjeldbo 2016*	clinical	clinical samples	cervix	6	6	–	Fjeldbo et al.[88]
28324887	Suh 2017*	clinical	clinical samples	head and neck	21 (5)	21	–	Suh et al.[89]
28400426	Yang 2017*	clinical	clinical samples	bladder	24	24	–	Yang et al.[90]
30037853	Ye 2018	*in vitro*	MCF-7, MCF10A, MCF12A, MDA-MB-157, MDA-MB-175, MDA-MB-231, MDA-MB-436, MDA-MB-468, SKBR3, SUM1315MO2, SUM185PE, SUM229, SUM149PT, SUM159PT, SUM225CWN, T47D, ZR-75-1	breast	42	42	1%, 24 h	Ye et al.[91]
29729848	Yang 2018* (prostate)	both	PNT2-C2, LNCaP, DU-145, PC-3, clinical samples	prostate	28 (14)	28	1%, 24 h	Yang et al.[92]
29423096	Yang 2018* (sarcoma)	both	HT1080, SKUT1, sNF96.2, 93T449, SW684, SW872, SW982, clinical samples	soft tissue sarcoma	24	24	1%, 24 h	Yang et al.[93]
30257451	Trong 2018	*in vitro*	NCH551b, NCH612, NCH620, NCH645, NCH421k, NCH601, NCH644, NCH660h	brain (glioma)	5 (2)	5	1.5%, 72 h	Dao et al.[94]

(*Continued on next page*)

Table 1. *Continued*

PubMed ID	Signature	Clinical/ *in vitro*	Cell line	Cell/tissue origin*	# Gene symbols	Final num. genes	Experimental conditions	Reference
30973670	Chen 2019	*in vitro*	A549, HCC827	lung (adenocarcinoma)	17	17	1%, 72 h	Chen et al.[95]
31572060	Zou 2019*	clinical	clinical samples	colorectal	14 (9)	14	–	Zou et al.[96]
32887635	Zhang 2020*	both	Huh-7, HepG2, clinical samples	liver	3	3	0%–1%, 24 h	Zhang et al.[51]
32724434	Wang 2020*	clinical	clinical samples	breast	14 (7)	14	–	Wang et al.[97]
33133157	Shou 2020*	clinical	clinical samples	skin (melanoma)	7 (3)	7	–	Shou et al.[98]
32500034	Lin 2020*	clinical	clinical samples	brain (glioma)	5	5	–	Lin et al.[99]
32655624	Mo 2020*	clinical	clinical samples	lung (adenocarcinoma)	4	4	–	Mo et al.[100]
32655701	Sun 2020*	clinical	clinical samples	early-stage lung (adenocarcinoma)	16 (11)	16	–	Sun et al.[101]
32887267	Tardon 2020	*in vitro*	Ge835, Ge898, Ge904, LN18, and LN229	brain (glioblastoma multiforme)	19	19	1%, 48 h	Calvo et al.[102]
35641902	Santamaria 2022*	both	33 cell types, clinical samples (TCGA)	meta-analysis from different datasets (pan-cancer)	16	16	0.1%–5%, 2–48 h	Puente-Santamaría et al.[103]
35155681	Wang 2022*	clinical	clinical samples	glioblastoma multiforme	23	23	–	Wang et al.[104]
35079065	Lane 2022	*in vitro*	A549, NCI-H2122, NCI-H1395, NCI-H1838, NCI-H520, NCI-H1703, NCI-H2170, NCI-H1869	lung adenocarcinoma, lung squamous cell carcinoma	28	28	1%, 24 h	Lane et al.[105]
34868920	Gao 2021*	clinical	clinical samples (TCGA, GTEx)	glioma	7 (1)	7	–	Gao et al.[106]
34093582	Khouzam 2021	*in vitro*	MDA-MB-231, MCF-7, HeLa, SiHa, HT-29, SW-620, A549, H226, TOV-112D, SKOV-3, MIA PaCa-2, Capan-1, BxPC-3, PANC-1	breast, cervical, colorectal, lung, ovarian, pancreatic	8	8	1%, 24 h	Abou Khouzam et al.[107]
33624645	Shou 2021*	clinical	clinical samples (TCGA)	melanoma	4	4	–	Shou et al.[108]
33616276	Zhang 2021	*in vitro*	HUH7, SNU-182, HLF	liver	21	21	0.5%–1%, 3–24 h	Zhang et al.[109]
33754044	Shi 2021*	clinical	clinical samples (TCGA, GEO)	lung adenocarcinoma	10 (7)	10	–	Shi et al.[110]
35769999	Liu 2022*	clinical	clinical samples (TCGA)	cervical cancer	6 (2)	6	–	Liu et al.[111]
35734431	Xu 2022*	clinical	clinical samples (TCGA, GEO)	colon	3	3	–	Xu et al.[50]
34950205	Wei 2021*	clinical	clinical samples (TCGA, GEO)	ovarian	8 (2)	8	–	Wei et al.[112]
34938106	He 2021*	clinical	clinical samples (TCGA)	colon	4 (1)	4	–	He et al.[113]
34722497	Xia 2021*	both	LNCaP, DU145, clinical samples (TCGA)	prostate	7 (1)	7	0.5%, 72 h	Xia et al.[114]
34490098	Sun 2021*	both	MDA-MB-231, clinical samples (TCGA, GEO)	triple-negative breast cancer	3	3	1%–1.5%, 24 h	Sun et al.[49]

(*Continued on next page*)

Table 1. *Continued*

PubMed ID	Signature	Clinical/ *in vitro*	Cell line	Cell/tissue origin*	# Gene symbols	Final num. genes	Experimental conditions	Reference
34194464	Li 2021*	clinical	clinical samples (TCGA, ICGC, GEO)	liver	8 (2)	8	–	Li et al.[115]
33941139	Zhao 2021*	clinical	clinical samples (TCGA, GEO)	oral squamous cell carcinoma	4	4	–	Zhao et al.[116]
36384128	Lombardi 2022	*in vitro*	PC3, T47D, A549 and HCT-116, HepG2, RCC4, HeLa, HUVEC, mel501	pan-cancer	48	48	0.5%, 16 h	Lombardi et al.[117]

Published hypoxia signatures, identified by PubMed ID, first author name, and year of publication. Table indicates originating group, source of tissue, associated malignancy, and hypoxia conditions tested. Signatures derived using clinical samples are marked with asterisks. The number of genes identified in the signature is given in column 5, the arrow symbol "→" indicates the number of genes that survived the reannotation processing step. Downregulated genes (if any) are reported in parentheses.

Most authors selected one such scoring method when applying and assessing their derived hypoxia signature(s), and at present there is no consensus on which score is most appropriate to use for a given context. Thus, we assessed 14 summary scoring methods across the 70 hypoxia signatures through an analytical framework that can be applied to any collection of gene signatures (Table S4).

To illustrate our analytical approach and the impact of the different scoring methods on signature performance we show the performance of one signature in human breast cancer cell lines (the Toustrup signature; Figures 2A–2C). The distribution derived from genes in the Toustrup signature show marked differences when comparing hypoxic and normoxic samples from GEO: GSE29406 (three normoxic and three hypoxic MCF7 cell-line samples, hypoxic = 1% oxygen for 24 h; Figure 2A). This distinction is missing when using RGSs of the same length. Such differences can be summarized using a *p* value, derived from the null distribution estimated using RGS (Figure 2B). These *p* values can be summarized across multiple cell lines and datasets as an "accuracy index" in an approach fundamental to our *in vitro* signature evaluation. This index is defined as the number of pairwise combinations with a significant difference over the total number of pairs analyzed (see STAR Methods). This measure can be used to determine the best score and signature combination. For instance, with the Toustrup signature, interquartile mean (IQM), which excludes outlier influences, reaches the highest accuracy index in breast cancer cell-line experiments (Figure 2C).

To find the most effective signature/scoring combination for measurement of hypoxia across cell lines, we carried out a pan-cancer analysis. Here, the validity of all hypoxia signature/score combinations was investigated across 28 sequencing platforms, 104 cell lines, and 1,198 pairwise comparisons of hypoxic and normoxic samples (Table S5). Results were compared to 54 million RGSs for each of the 14 scores.

As in breast cancer cell lines, the choice of scoring method markedly affected the ability of the signature to reflect hypoxic status. The best-performing signature/score combination was the Tardon signature when using the IQM score (Tardon/IQM). Although derived from glioblastoma multiforme cell lines exposed to 1% oxygen for 48 h, this 19-gene signature was widely effective across multiple cell lines, oxygen tensions, and hypoxic exposures (Figure 3), achieving an accuracy of 94.0%. If not the top-ranking signature/score combination in the individual tumor types tested, Tardon/IQM was always within the top 10 of the 1,050 signature/score combinations tests in each cancer type and within the top three in eight of the 12 tissue types tested. Where not the most accurate, Tardon/IQM was within 0.28%–5% of the most accurate combination (Table S6). Of note, samples that were misclassified by the Tardon signature (blue values for $-\log_{10}(p)$ in Figure 3) tended to be non-significant across most/all signatures (horizontal blue lines). These samples typically originated from experiments where colorectal cancer cell lines were exposed to hypoxia for a short time (i.e., between 1 and 2 h in HCT116, HCT-15, LoVo, WiDr, and COLO-125), which might be too early to observe a marked transcriptional response to hypoxia, and experiments when VHL was reintroduced in hypoxia vs. VHL mutated in normoxia in 786-O cells, which could be expected as reintroduction of VHL might not completely recapitulate the hypoxia response. Some breast cancer samples also were universally misclassified across signatures. This could be due to experimental artifacts, such as residual oxygen being left in plastic tissue culture plates[124] or oxygen being present via other channels. Full details of those misclassified by Tardon/IQM are found in Table S7.

A second key observation was that several scoring methods appeared consistently ineffective at denoting hypoxia in this bulk RNA sequencing (RNA-seq) data, irrespective of the experiment used (for instance, midrange; further examples in Figures S2–S14). Even the best-performing signature, Tardon, dropped to 0.6% accuracy with single-sample gene set enrichment analysis (ssGSEA) or 33.1% if GSVA was used, illustrating the importance of summary score choice. IQM had the highest number of signatures (28) with over 85% accuracy with 22 of these derived solely from *in vitro* cell culture. Other measures of central tendency also performed well, showing over 85% accuracy in a number of signatures: Tukey's trimean (24 signatures), midhinge (21 signatures), and median (16 signatures). Tardon was also the top performer using each of these metrics with 93.7%, 93.2%, and 92.6%, respectively.

The best-performing signature derived using clinical samples with IQM was Yang 2018 sarcoma, achieving 92.8% accuracy

CellPress
OPEN ACCESS

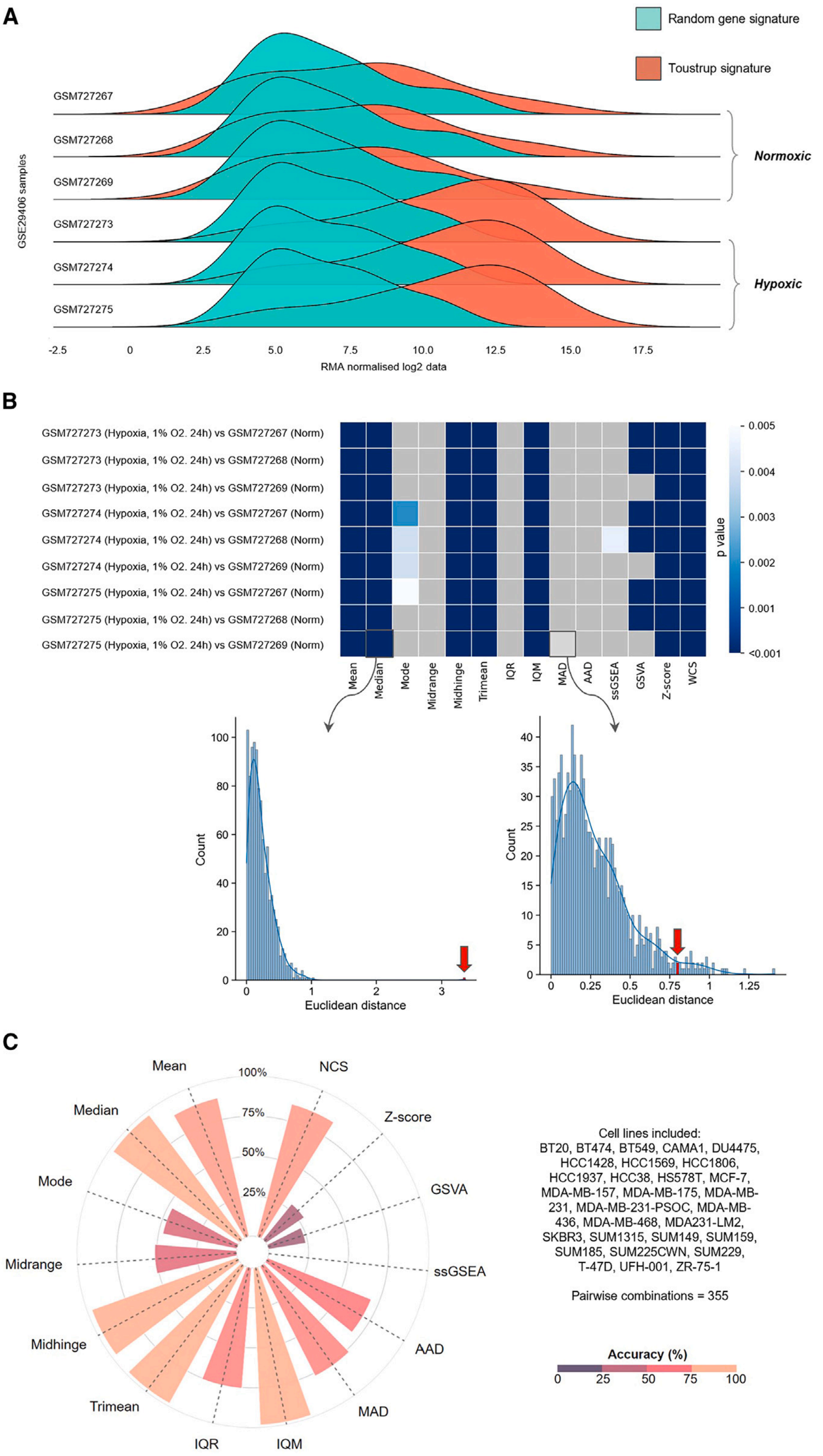

Figure 2. The performance of the top-performing signature in cell-line datasets, the Toustrup signature

A single published hypoxia experiment (GEO: GSE29406) is shown, alongside an analysis of the Toustrup signature performance across all publicly available hypoxia experiments with breast cancer cell lines.

(A) The distribution of genes in three normoxic and three hypoxic replicates of MCF-7 cells. The expression of the genes within the Toustrup hypoxia signature is shown in red and a random gene signature of the same length is shown in green. Hypoxia was defined as cells being placed in 1% oxygen for 24 h. (B) Comparison of 14 scoring methods applied to the Toustrup hypoxia signature in GEO: GSE29406. The darker the shade of blue in the heatmap, the more accurate the score is at differentiating between hypoxic and non-hypoxic samples, compared to random gene signatures (RGSs) of the same length. Gray indicates that the score/signature combination did not significantly outperform RGS ($p > 0.005$). The two density plots below the heatmap show how the p value is calculated for median and mean absolute deviation (MAD) scores using the Euclidean distances from RGS as a null distribution. Within each plot, a bar highlighted by a red arrow marks the specific bin where the Euclidean distance corresponding to the original signature is located. Notably, the Euclidean distance obtained using the median score has a significantly higher value distinctly separating it from the null distribution. This is not the case with the MAD score, and this yields a non-significant result (gray in the heatmap). (C) Summary of the performance of the Toustrup signature using the different scoring methods across publicly available gene expression data from hypoxia experiments using breast cancer cell lines. The radar bar plot outlines the percentage accuracy achieved using the Toustrup signature and the different scoring methods. The larger and more beige the spoke, the more accurate the scoring method (radial axis: percentage accuracy at correctly determining hypoxic samples). The highest accuracy was achieved using IQM (98.3%), followed by trimean (98.0%) and the median score (97.5%).

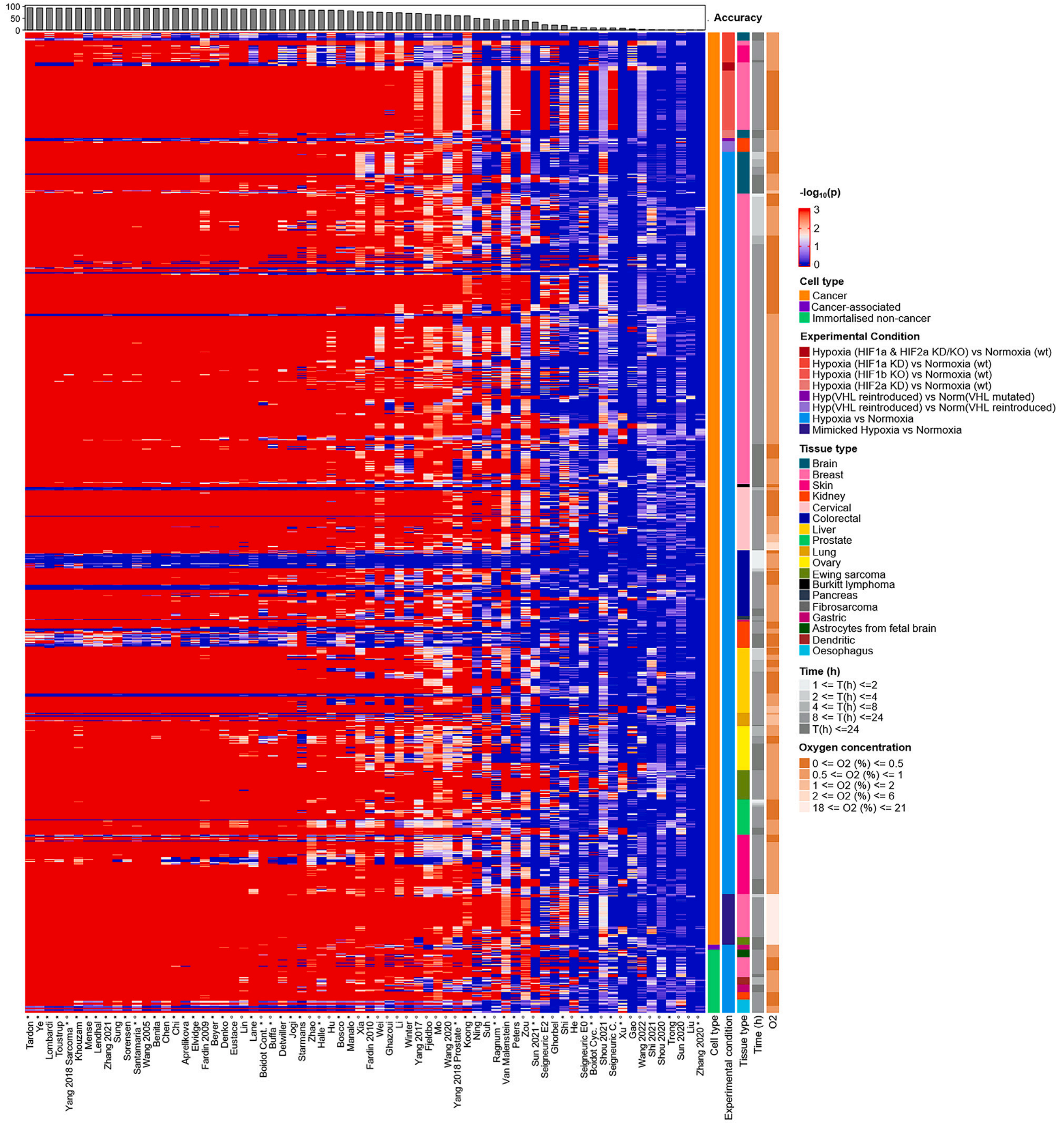

Figure 3. Comparison of the performance of the 70 published hypoxia signatures using the IQM in 104 cancer cell lines

Performance of the 70 hypoxia signatures across all hypoxia experiments identified in the GEO using the IQM. In the main body, the brighter the shade of red, the more accurate the signatures at differentiating between hypoxic and non-hypoxic samples compared to RGSs of the same length. The legend on the right-hand side shows several features for the samples analyzed (legend titles are reported at the start of the x axis). In addition, asterisk (*) denotes signatures derived using cell lines, whereas "°" denotes signatures derived using clinical samples. At the summit of the figure, percentage accuracy is displayed (maximum accuracy: 94%, Tardon).

in our cell-line analysis (Figure 3). This 24-gene signature was derived from seven soft-tissue sarcoma cell lines and refined in clinical cohorts. Some signatures, for instance the 16-gene Sun 2020 signature, six-gene Liu signature, and seven-gene Shou 2020 signature, appeared generally ineffective at identifying hypoxia irrespective of score choice. The highest accuracies achieved with Sun 2020, Liu and Shou 2020 were 3.1% (normalized comulative score, NCS), 2.7% (mean), and 1.4% (NCS), respectively. Interestingly, these signatures were derived using clinical samples only.

We also investigated the performance of the signatures in GEO: GSE30979, which consisted of normoxic/hypoxic comparison of *ex vivo* tumor fragments from non-small cell lung cancer, containing mixed cell types.[125] This analysis showed only 61.1% accuracy with Tardon/IQM, with other signatures performing better (e.g., Starmans obtaining 88.3% using the mean score). How cell-line and *ex vivo* results are transferable to the clinic needs to be elucidated and will be explored later.

To conclude, this analysis of bulk RNA datasets of 104 cell lines demonstrates variability in the performance of both scores and signatures. Thus, it is essential to carefully consider the choice of hypoxia gene expression signature and score used to identify an *in vitro* active hypoxia response.

Hypoxia signatures with efficacy in bulk RNA from cell lines show validity in single-cell data

Single-cell RNA-seq (scRNA-seq) data from hypoxia and normoxia experiments are rare, and so very little is known on how to apply hypoxia signatures to single-cell data. To address this, we interrogated two in-house-generated scRNA-seq datasets from cell lines to investigate whether the performance of hypoxia signatures in scRNA-seq was similar to that in the bulk RNA-seq data. The best-performing signature/score combination in bulk RNA-seq, Tardon/IQM, clearly divides normoxic and hypoxic cells in both cell lines (MCF7 and HCC1806), and, overall, results in bulk RNA-seq platforms appear similar to those in these single-cell datasets (Figure 4). This clear division is also seen in normoxic/hypoxic normal adjacent tissue (NAT) from human kidneys, generated in a previous study[117] (GEO: GSE200207; Figure S15).

One particular signature appeared to have superior performance when applied to our single-cell dataset compared to bulk RNA data (van Malenstein). The van Malenstein signature is a concise four-gene signature derived from HepG2 cells (CCNG2, EGLN3, ERO1A, and WDR45B). In the single-cell datasets, this signature is dominated by the expression of ERO1A and WDR45B (Figure S16). ERO1A appears upregulated in hypoxia and downregulated in normoxia, whereas WDR45B is upregulated in normoxia and downregulated in hypoxia. As these two genes have opposite patterns of expression, the intra-signature correlation is low (Figure S17). The unusual performance of this signature can be traced back to its small set of two dominant features (ERO1A and WDR45B), resulting from the inherent sparsity of single-cell data versus bulk. In single-cell data, not all signature genes contribute equally to the score. For small signatures, a few features can dominate the overall score, skewing analysis results and reducing robustness against technical variation. Indeed, normalized rank of gene expression values for the van Malenstein signature in both scRNA-seq and bulk data revealed higher expression of both ERO1A and WDR45B in single-cell data compared to bulk, particularly in hypoxia. This explains this difference in signature performance between technologies. The impact of single-cell data sparsity on analytical methods is well described.[126] However, despite observable differences between bulk and single-cell data, with van Malenstein serving as a notable example, most signatures exhibit impressive consistency in performance between these two sequencing platforms. The Tardon signature continues to stand out as a leading performer distinctly separating normoxic from hypoxic cells.

Two signatures stand out as the most promising for clinical use across tumor types

Because of the major microenvironmental differences between *in vitro* cellular models and human tumors, it is essential to assess whether hypoxia signatures and scores that work well in cell lines also are the most appropriate in human tumors. There are debates surrounding the most effective type of hypoxia measurements with the advances in modern technology, as perhaps the ‘‘gold standard’’ oxygen electrode measurements have been surpassed. There are no large cohort studies across tumor types to establish the most reliable and relevant indicator of hypoxia in the clinical setting. Further, at present, there are no large datasets that have gene expression data, prognostic information, and hypoxia measurements from a variety of technologies. Thus, one commonly used method of assessing the clinical relevance of a hypoxia signature is by examining prognosis in patient cohorts (i.e., if those individuals with a higher score have a worse prognosis). This assumes that hypoxia is linked with prognosis, which has support both from biological and previous clinical studies (exemplar publications[1,127,128]). However, other factors aside from hypoxia clearly can influence prognosis. The current study is limited to the validation of hypoxia signatures in datasets that have prognostic information. However, we take a novel approach, utilizing tumor and adjacent normal-tissue samples from one of largest cancer datasets available: The Cancer Genome Atlas (TCGA).

To find the most promising signatures for clinical use, we identified signatures that fulfill three criteria: (1) NAT should have a lower hypoxia score than tumor samples (criterion 1), (2) effective hypoxia signatures should be significantly different from random gene signatures of the same length when comparing NAT and tumor tissues (criterion 2), and (3) efficacious hypoxia signatures should confer prognostic information (criterion 3).

Two signatures, Buffa and Ragnum, showed higher scores in tumors compared to NAT across all 10 cancer types (Figure S18). Both signatures were developed using clinical samples: the 51-gene Buffa signature was derived using human head and neck and breast cancer samples,[77] whereas the 32-gene Ragnum signature was developed from four prostate cancer cell lines (22Rv1, LNCaP, PC-3, and DU 145) and honed in clinical samples from prostate patients.[87] Pathway analysis revealed both signatures were significantly enriched for glycolysis/gluconeogenesis, central carbon metabolism in cancer, as well as more generally carbon metabolism (Figure S19). While there is a notable overlap in terms of significantly enriched pathways, with four of the six pathways in the Ragnum signature also enriched in Buffa, the two signatures modestly intersect at the level

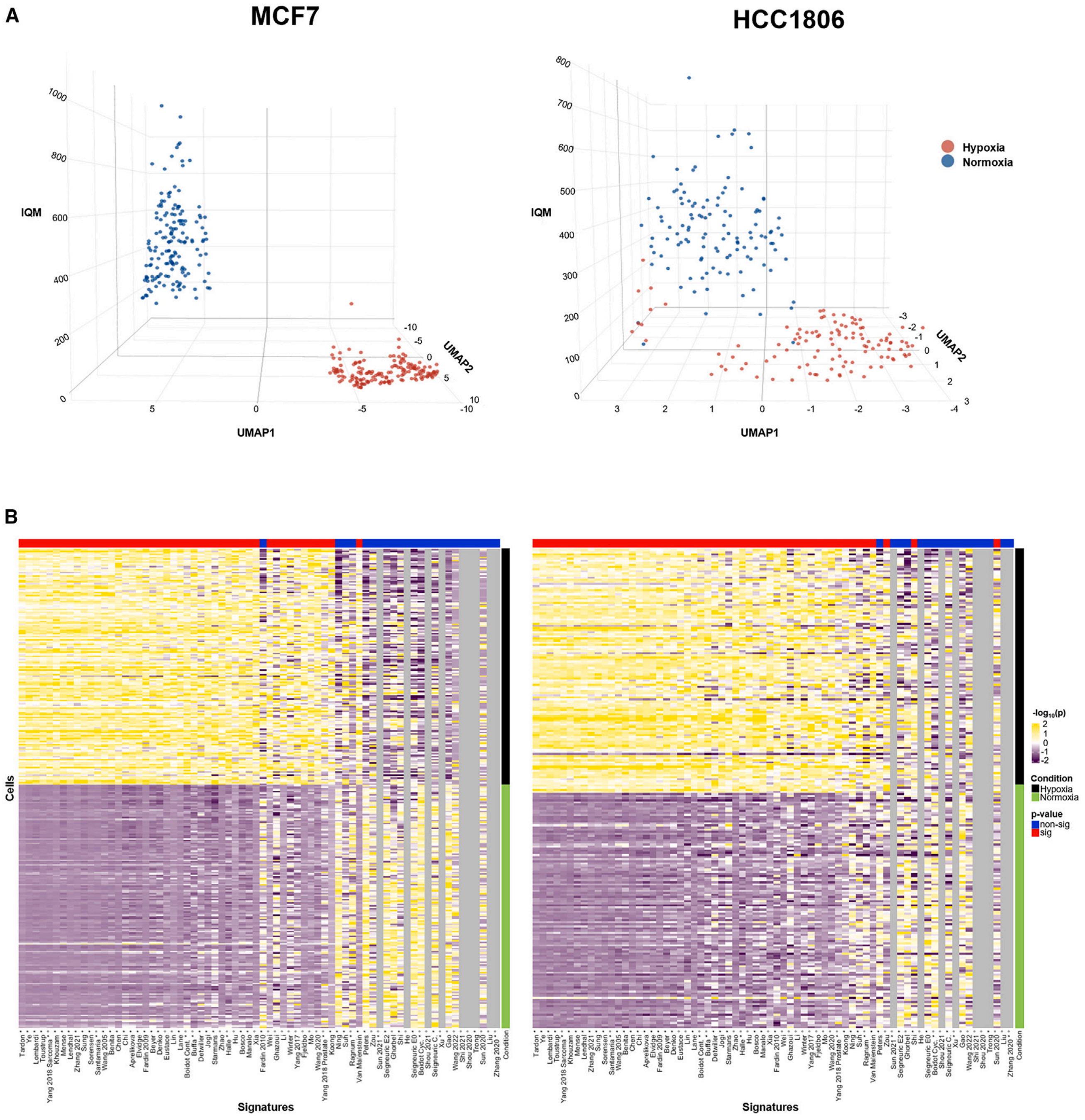

Figure 4. Performance of the Tardon signature using the IQM in scRNA-seq data for MCF7 and HCC1806 cells under normoxic and hypoxic conditions

After reaching maximum accuracy in bulk RNA-seq, Tardon performs extremely well in the more sparse scRNA-seq data. This is shown in all three panels. (A) 3D uniform manifold approximation and projection (UMAP) representation with x and y axes displaying UMAP dimensions and z axis showing the IQM score of the Tardon signature for individual cells. Separation between normoxia and hypoxia is seen in both cell lines. (B) IQM scores across all tested signatures, annotated vertically by experimental condition and horizontally by significance level. The heatmap reveals high concordance between single-cell and bulk data. Gray vertical lines indicate instances where insufficient genes are available for IQM calculation, which requires a minimum of four values.

of individual genes. The Buffa and Ragnum signatures share just a quartet of genes: ADM, DDIT4, P4HA1, and HILPDA.

Buffa and Ragnum achieved higher scores in tumors compared to NAT across studied tumor types using two and six scores respectively (Buffa: mean and NCS. Ragnum : mean, IQM, median, midhinge, trimean, and NCS). When comparing these signature score combinations, they were consistently very different from RGS over 1,000 simulations (illustrated in Figure S20 and shown Table S8). All eight signature/score combinations achieved over 99% signature performance index (SPI), with the exception of Buffa/NCS, which achieved 97.6%. It is noteworthy that Tardon/IQM, the best-performing signature and score combination in cell lines, had a higher average score in tumor compared to normal tissues in six of the 10 cancer types (HNSC, LUSC, COAD, LUAD, THCA, and UCEC) and had an SPI of only 55.9%. Further, in cell lines, Buffa signature demonstrated reasonable effectiveness in cell lines, achieving its peak accuracy of 87.3% with the IQM score. In contrast, the Ragnum signature's highest accuracy was significantly lower, at only 44.3%, also recorded with the IQM score.

Prognostic ability was investigated across 5,401 solid tumor samples in TCGA (for further details, see STAR Methods). The best-performing signature/score combination was Buffa/mean, which was significantly prognostic in seven out of 10 individual tumor types (HNSC, BRCA, LUAD, LIHC, UCEC, THCA, and PRAD; Figure S21). Although Ragnum/IQM was significantly prognostic in six out of 10 tumor types (BRCA, LUAD, PRAD, THCA, LIHC, and UCEC; Figure S22). Neither combination was significantly prognostic on COAD, LUSC, or STAD. Using the median as the cutoff point for high and low hypoxia showed both Buffa/mean with Ragnum/IQM were significantly prognostic in four tumor types, with both being prognostic in BRCA and LUAD (Buffa/mean: HNSC, BRCA, LUAD, and LIHC. Ragnum/IQM: BRCA, LUAD, LIHC, and UCEC). Revisiting the results from the 104 *in vitro* cell lines previously interrogated, Buffa/mean had a superior accuracy compared to Ragnum/IQM (78.5% vs. 44.3%). In colorectal cancer cell lines, Buffa/mean was 56.3% accurate, whereas Ragnum/IQM was only 17.5% accurate. It is hard to comment on the *in vitro* performance of these signature/score combinations in gastric adenocarcinoma and lung squamous cell carcinoma cell lines, as data from hypoxia experiments using these cancer types are very limited (Table S5).

In order to leverage hypoxia signatures to stratify patients to therapies, a threshold to define low and high hypoxia and thus at which to administer the treatment is helpful. Previous studies have employed the median hypoxia score within a distribution to determine eligibility for hypoxia-modifying therapies.[85,129] However, little is known as to whether the choice of median is optimal in individual or across tumor types. This is a complex question to answer. However, using TCGA data, Kaplan-Meier analyses suggest that perhaps a promising and practicable cut point for Buffa/mean at which to stratify patients with LIHC, LUAD, or HNSC is at the top 20th percentile (Figure 5). Multivariate Cox proportional hazard models underscore this (Table S9).

DISCUSSION

Transcriptomic signatures have been transformative in the oncology clinic, for example, in treatment selection for patients with estrogen receptor-positive early breast cancer.[130,131] Hypoxia-targeted therapies have utility across tumor types; however, the inability to select accurately patients for clinical trials has proved a critical impediment. Hypoxia signatures may hold the key, and this work is the largest and most comprehensive analysis and validation of hypoxia signatures to date. This study includes (1) a systematic review of published hypoxia signatures, (2) an unbiased analysis of hypoxia signature performance in all open-access hypoxia cell-line data from GEO spanning 104 different cell lines, and finally (3) a data-driven approach to find the most promising signature for use in clinical samples using the landmark cancer genomics study, TCGA. We also present a new method for signature evaluation using RGSs, which addresses a major need to develop methodology to evaluate gene signatures.

Looking at signatures as a whole, measures of central tendency appear to work better than those using ranking (e.g., GSVA). This indicates that the magnitude of gene expression, rather than just the relative ranking, might be important. The most effective signature and score combination on any cell line tested was Tardon/IQM. Across 1,198 pairwise combinations, Tardon/IQM yields an impressive 94.0% accuracy in identifying hypoxic samples in experiments (i.e., cells in hypoxic chambers vs. normoxic conditions). There were also some samples where most signatures failed, suggesting an experimental issue, either by design or otherwise (e.g., too short a time under hypoxia). However, it must be noted that this analysis is biased with respect to cell type and tissues commonly used in published models.

Although this is the most comprehensive evaluation of hypoxia signatures and scores in cell lines to date, it does not cover all cell lines, oxygen tensions, or durations under hypoxia. Therefore, when applying the results from this study, it is important to consider the experimental data upon which our conclusions are currently based (Table S5). Another consideration is that it is hard to quantify (1) how much weight to place on small percentage increases in accuracy, which may relate to only a handful of samples, and (2) the extent to which overfitting plays a role in tissue types where limited samples exist. Therefore, researchers may choose to use Tardon/IQM alongside the highest-ranking signature/score combination(s) for their cancer type of interest (Table S6) and compare the results. Undoubtedly, as more hypoxia experiments are added to the literature, this work should be revisited. In particular, further experiments on colorectal and kidney cell lines at a range of oxygen tensions/hypoxic exposures would be beneficial.

Tardon/IQM continued to perform well in single-cell data, which is relatively underexplored in this field, achieving excellent separation between hypoxic and normoxic breast cancer cells across two cell lines (MCF7 and HCC1806). Further single-cell validation is needed across datasets from different tissue types, as such data are currently rare.

For clinical samples, one major limitation of this study, and of others in the literature, was the lack of hypoxia measurements by methods alternative to gene expression that could provide a comparative assessment. Thus, we suggested a novel three-stage approach to identify the most promising signature and score for use in clinical samples (see STAR Methods). Throughout

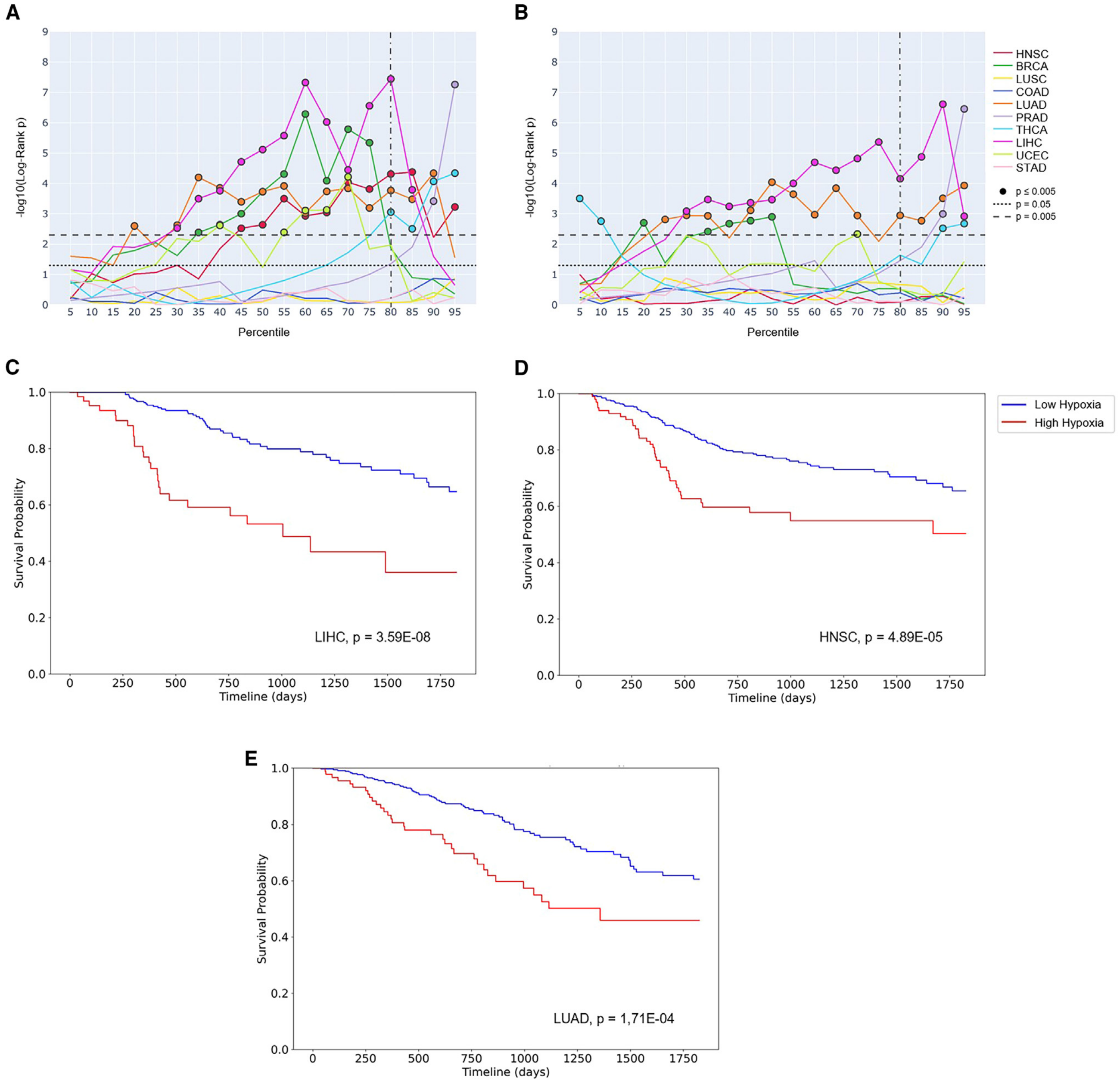

Figure 5. The Buffa signature emerges as a promising signature for clinical use

Survival analysis was conducted to evaluate the prognostic value of Buffa/mean and Ragnum/IQM in 10 cancer types in TCGA. Iterative dichotomization of the cohorts into "high" and "low" hypoxia groups at every fifth percentile threshold aimed to pinpoint the optimal percentile for maximum prognostic effectiveness and suggest a potentially useful threshold for subsequent clinical testing. Line plots for the Buffa/mean (A) and Ragnum/IQM (B) show the changes in the log-rank *p* from the Kaplan-Meier survival analysis across different percentiles for the individual cancer types. The line at the 80th percentile corresponds to 20% of the cohort being in the "high" hypoxia group. Individual Kaplan-Meier plots are shown for this promising cut point using Buffa/mean for LIHC (C), HNSC (D), and LUAD (E).

these three stages, Buffa and Ragnum performed extremely well. Prognostic efficacy across tumor types was best achieved by Buffa/mean and Ragnum/IQM. It should be noted that prognostic analyses were carried out in TCGA, which, like all resources, has limitations, including incomplete annotation of patient outcome and treatment data and its relatively short-term clinical follow-up interval.[132–135] Previous work has already shown that the Buffa signature is prognostic in other cohorts in breast cancer[77,136] (including METABRIC[137–139]), head and neck cancer,[77] and lung cancer.[77,140] Similarly, the Ragnum signature has demonstrated prognostic value in prostate[87] and pancreatic cancers.[141] While these findings highlight the potential of these signatures in

CellPress
OPEN ACCESS

predicting patient outcomes, it is unknown how much weight should be given to prognostic ability when assessing the accuracy of these signatures in measuring hypoxia. Large-scale prospective studies are needed to validate, ideally with multiple potential hypoxic measures, whether these signatures do truly reflect hypoxia (e.g., with [18F]-fluoromisonidazole PET-CT).[142] However, such studies have both methodological and logistical complications. Even if carried out, their results may be challenging to interpret where lack of concordance exists. Perhaps a more practical approach is to test promising signature/score combinations prospectively.

Following our comprehensive analysis, we recommend using Tardon/IQM to confirm hypoxic status in laboratory experiments, perhaps alongside the highest-ranking signature/score combination(s) for the cancer type of interest. For prospective evaluation in clinical trials, Buffa/mean and Ragnum/IQM pass our rigorous three-step evaluation. This work gives much-needed clarity to the field and provides an important reference to laboratory and clinician scientists who seek validation of hypoxic status and/or are considering orchestration of prospective trials.

Limitations of the study

This study represents a significant step forward in understanding the performance of hypoxia signatures across bulk and scRNA-seq data; however, there are some salient limitations. This study's significant reliance on publicly available datasets introduces variability arising from differences in experimental design, oxygen tensions, and durations of hypoxic exposure. This variability impacts the comparability and generalizability of the findings, underscoring the need for more standardized experimental protocols in future studies. While the analysis leverages a diverse range of datasets, the focus remains predominantly on commonly used cell lines publicly available in the GEO. These cell lines may not fully capture the heterogeneity of hypoxic responses observed across diverse tissue types and cancer subtypes. Expanding validations to underrepresented cell lines, particularly those reflective of less-studied malignancies such as colorectal or kidney cancers, would enhance the robustness of these findings. Further, the limited availability of hypoxia versus normoxia experiments with scRNA-seq data is important to note. A concerted effort by the scientific community to contribute more such scRNA-seq data will be valuable.

RESOURCE AVAILABILITY

Lead contact

Requests for further information should be directed to Dr. Benjamin Harris (benjamin.harris@oncology.ox.ac.uk).

Materials availability

This study did not generate new unique reagents.

Data and code availability

Dataset availability is detailed in the table in STAR★Methods. The SigScores package is found on GitHub (https://alebarberis.github.io/sigscores/index.html).

ACKNOWLEDGMENTS

This work was supported by Cancer Research UK Programme grant 23969 and European Research Council Programme grant 772970 to F.M.B. B.H.L.H. was supported by Cancer Research UK, Oxfordshire Health Service Research Committee, and the Thouron Award. M.D.G. received support from Urology Cancer Research and Education (UCARE). We would like to thank Professor Michael B. Fertleman, Dr. Louis J. Koizia, and Dr. Jason L. Walsh for their support during this work.

AUTHOR CONTRIBUTIONS

Conceptualization, M.D.G., F.M.B., and B.H.L.H.; methodology, M.D.G., F.M.B., and B.H.L.H.; data curation, M.D.G., B.E., and A.B.; formal analysis, M.D.G., B.E., F.H., and B.H.L.H.; software, A.B. and B.E.; visualization, M.D.G., B.E., and B.H.L.H.; writing—review & editing, all authors; supervision, F.M.B. and B.H.L.H.; investigation, M.D.G., F.M.B., S.H., A.L.H., and B.H.L.H.; funding acquisition, M.D.G., F.M.B., and B.H.L.H.

DECLARATION OF INTERESTS

The authors declare no competing interests.

STAR★METHODS

Detailed methods are provided in the online version of this paper and include the following:

- KEY RESOURCES TABLE
- METHOD DETAILS
  - Identification of hypoxia gene expression signatures
  - Reannotation of hypoxia signatures
  - Pathway analysis
  - Cell line data
  - Single-cell data
  - Clinical data
  - Calculation of hypoxia signature summary scores
  - Comparison with random gene signatures (RGS) in bulk gene expression data
  - Comparison with random gene signatures (RGS) in single-cell gene expression data
  - Statistical evaluation of tumor VS NAT
  - Comparison with RGS in clinical samples
  - Survival analysis

SUPPLEMENTAL INFORMATION

Supplemental information can be found online at https://doi.org/10.1016/j.xgen.2025.100764.

Received: August 13, 2024
Revised: November 4, 2024
Accepted: January 7, 2025
Published: January 31, 2025

REFERENCES

1. Harris, A.L. (2002). Hypoxia — a key regulatory factor in tumour growth. Nat. Rev. Cancer *2*, 38–47.
2. Petrova, V., Annicchiarico-Petruzzelli, M., Melino, G., and Amelio, I. (2018). The hypoxic tumour microenvironment. Oncogenesis *7*, 10.
3. Brizel, D.M., Dodge, R.K., Clough, R.W., and Dewhirst, M.W. (1999). Oxygenation of head and neck cancer: changes during radiotherapy and impact on treatment outcome. Radiother. Oncol. *53*, 113–117.
4. Nordsmark, M., and Overgaard, J. (2004). Tumor hypoxia is independent of hemoglobin and prognostic for loco-regional tumor control after primary radiotherapy in advanced head and neck cancer. Acta Oncol. *43*, 396–403.
5. Luoto, K.R., Kumareswaran, R., and Bristow, R.G. (2013). Tumor hypoxia as a driving force in genetic instability. Genome Integr. *4*, 5.

6. Noman, M.Z., Hasmim, M., Messai, Y., Terry, S., Kieda, C., Janji, B., and Chouaib, S. (2015). Hypoxia: a key player in antitumor immune response. A Review in the Theme: Cellular Responses to Hypoxia. Am. J. Physiol. Cell Physiol. *309*, C569–C579.
7. Mohyeldin, A., Garzón-Muvdi, T., and Quiñones-Hinojosa, A. (2010). Oxygen in stem cell biology: a critical component of the stem cell niche. Cell Stem Cell *7*, 150–161.
8. Rankin, E.B., and Giaccia, A.J. (2016). Hypoxic control of metastasis. Science *352*, 175–180.
9. Tutzauer, J., Sjöström, M., Holmberg, E., Karlsson, P., Killander, F., Leeb-Lundberg, L.M.F., Malmström, P., Niméus, E., Fernö, M., and Jögi, A. (2022). Breast cancer hypoxia in relation to prognosis and benefit from radiotherapy after breast-conserving surgery in a large, randomised trial with long-term follow-up. Br. J. Cancer *126*, 1145–1156.
10. Theodoropoulos, V.E., Lazaris, A.C., Sofras, F., Gerzelis, I., Tsoukala, V., Ghikonti, I., Manikas, K., and Kastriotis, I. (2004). Hypoxia-Inducible Factor 1a Expression Correlates with Angiogenesis and Unfavorable Prognosis in Bladder Cancer. Eur. Urol. *46*, 200–208.
11. Domènech, M., Hernández, A., Plaja, A., Martínez-Balibrea, E., and Balañà, C. (2021). Hypoxia: The Cornerstone of Glioblastoma. Int. J. Mol. Sci. *22*, 12608.
12. Griffiths, E.A., Pritchard, S.A., Welch, I.M., Price, P.M., and West, C.M. (2005). Is the hypoxia-inducible factor pathway important in gastric cancer? Eur. J. Cancer *41*, 2792–2805.
13. Brizel, D.M., Sibley, G.S., Prosnitz, L.R., Scher, R.L., and Dewhirst, M.W. (1997). Tumor hypoxia adversely affects the prognosis of carcinoma of the head and neck. Int. J. Radiat. Oncol. Biol. Phys. *38*, 285–289.
14. Xiang, Z.-L., Zeng, Z.C., Fan, J., Tang, Z.Y., Zeng, H.Y., and Gao, D.M. (2011). Gene expression profiling of fixed tissues identified hypoxia-inducible factor-1α, VEGF, and matrix metalloproteinase-2 as biomarkers of lymph node metastasis in hepatocellular carcinoma. Clin. Cancer Res. *17*, 5463–5472.
15. Hung, J.-J., Yang, M.H., Hsu, H.S., Hsu, W.H., Liu, J.S., and Wu, K.J. (2009). Prognostic significance of hypoxia-inducible factor-1 , TWIST1 and Snail expression in resectable non-small cell lung cancer. Thorax *64*, 1082–1089.
16. Tanaka, N., Kato, H., Inose, T., Kimura, H., Faried, A., Sohda, M., Nakajima, M., Fukai, Y., Miyazaki, T., Masuda, N., et al. (2008). Expression of carbonic anhydrase 9, a potential intrinsic marker of hypoxia, is associated with poor prognosis in oesophageal squamous cell carcinoma. Br. J. Cancer *99*, 1468–1475.
17. Milosevic, M., Warde, P., Ménard, C., Chung, P., Toi, A., Ishkanian, A., McLean, M., Pintilie, M., Sykes, J., Gospodarowicz, M., et al. (2012). Tumor hypoxia predicts biochemical failure following radiotherapy for clinically localized prostate cancer. Clin. Cancer Res. *18*, 2108–2114.
18. Overgaard, J., Hansen, H.S., Overgaard, M., Bastholt, L., Berthelsen, A., Specht, L., Lindeløv, B., and Jørgensen, K. (1998). A randomized double-blind phase III study of nimorazole as a hypoxic radiosensitizer of primary radiotherapy in supraglottic larynx and pharynx carcinoma. Results of the Danish Head and Neck Cancer Study (DAHANCA) Protocol 5-85. Radiother. Oncol. *46*, 135–146.
19. Vilaplana-Lopera, N., Besh, M., and Moon, E.J. (2021). Targeting Hypoxia: Revival of Old Remedies. Biomolecules *11*, 1604.
20. Rischin, D., Peters, L., Hicks, R., Hughes, P., Fisher, R., Hart, R., Sexton, M., D'Costa, I., and von Roemeling, R. (2001). Phase I trial of concurrent tirapazamine, cisplatin, and radiotherapy in patients with advanced head and neck cancer. J. Clin. Oncol. *19*, 535–542.
21. Aghajanian, C., Brown, C., O'flaherty, C., Fleischauer, A., Curtin, J., Roemeling, R., and Spriggs, D.R. (1997). Phase I study of tirapazamine and cisplatin in patients with recurrent cervical cancer. Gynecol. Oncol. *67*, 127–130.
22. Craighead, P.S., Pearcey, R., and Stuart, G. (2000). A phase I/II evaluation of tirapazamine administered intravenously concurrent with cisplatin and radiotherapy in women with locally advanced cervical cancer. Int. J. Radiat. Oncol. Biol. Phys. *48*, 791–795.
23. Le, Q.T., McCoy, J., Williamson, S., Ryu, J., Gaspar, L.E., Edelman, M.J., Dakhil, S.R., Sides, S.D., Crowley, J.J., and Gandara, D.R.; Southwest oncology group (2004). Phase I study of tirapazamine plus cisplatin/etoposide and concurrent thoracic radiotherapy in limited-stage small cell lung cancer (S0004): a Southwest Oncology Group study. Clin. Cancer Res. *10*, 5418–5424.
24. Johnson, C.A., Kilpatrick, D., von Roemeling, R., Langer, C., Graham, M.A., Greenslade, D., Kennedy, G., Keenan, E., and O'Dwyer, P.J. (1997). Phase I trial of tirapazamine in combination with cisplatin in a single dose every 3 weeks in patients with solid tumors. J. Clin. Oncol. *15*, 773–780.
25. Senan, S., Rampling, R., Graham, M.A., Wilson, P., Robin, H., Jr., Eckardt, N., Lawson, N., McDonald, A., von Roemeling, R., Workman, P., and Kaye, S.B. (1997). Phase I and pharmacokinetic study of tirapazamine (SR 4233) administered every three weeks. Clin. Cancer Res. *3*, 31–38.
26. Shulman, L.N., Buswell, L., Riese, N., Doherty, N., Loeffler, J.S., von Roemeling, R.W., and Coleman, C.N. (1999). Phase I trial of the hypoxic cell cytotoxin tirapazamine with concurrent radiation therapy in the treatment of refractory solid tumors. Int. J. Radiat. Oncol. Biol. Phys. *44*, 349–353.
27. Bedikian, A.Y., Legha, S.S., Eton, O., Buzaid, A.C., Papadopoulos, N., Coates, S., Simmons, T., Neefe, J., and von Roemeling, R. (1997). Phase II trial of tirapazamine combined with cisplatin in chemotherapy of advanced malignant melanoma. Ann. Oncol. *8*, 363–367.
28. Rischin, D., Peters, L., Fisher, R., Macann, A., Denham, J., Poulsen, M., Jackson, M., Kenny, L., Penniment, M., Corry, J., et al. (2005). Tirapazamine, Cisplatin, and Radiation versus Fluorouracil, Cisplatin, and Radiation in patients with locally advanced head and neck cancer: a randomized phase II trial of the Trans-Tasman Radiation Oncology Group (TROG 98.02). J. Clin. Oncol. *23*, 79–87.
29. Lee, D.J., Trotti, A., Spencer, S., Rostock, R., Fisher, C., von Roemeling, R., Harvey, E., and Groves, E. (1998). Concurrent tirapazamine and radiotherapy for advanced head and neck carcinomas: a Phase II study. Int. J. Radiat. Oncol. Biol. Phys. *42*, 811–815.
30. Treat, J., Johnson, E., Langer, C., Belani, C., Haynes, B., Greenberg, R., Rodriquez, R., Drobins, P., Miller, W., Jr., Meehan, L., et al. (1998). Tirapazamine with cisplatin in patients with advanced non-small-cell lung cancer: a phase II study. J. Clin. Oncol. *16*, 3524–3527.
31. Reck, M., von Pawel, J., Nimmermann, C., Groth, G., and Gatzemeier, U. (2004). Phase II-trial of tirapazamine in combination with cisplatin and gemcitabine in patients with advanced non-small-cell-lung-cancer (NSCLC). Pneumologie *58*, 845–849.
32. von Pawel, J., von Roemeling, R., Gatzemeier, U., Boyer, M., Elisson, L.O., Clark, P., Talbot, D., Rey, A., Butler, T.W., Hirsh, V., et al. (2000). Tirapazamine plus cisplatin versus cisplatin in advanced non-small-cell lung cancer: A report of the international CATAPULT I study group. Cisplatin and Tirapazamine in Subjects with Advanced Previously Untreated Non-Small-Cell Lung Tumors. J. Clin. Oncol. *18*, 1351–1359.
33. Shepherd, F., Koschel, G., Von Pawel, J., Gatzmeier, U., Van Zandwiyk, N., Woll, P., Van Klavren, R., Krasko, P., Desimone, P., Nicolson, M., et al. (2000). Comparison of Tirazone (Tirapazamine) and cisplatin vs. etoposide and cisplatin in advanced non-small cell lung cancer (NSCLC): Final results of the international Phase III CATAPULT II Trial. Lung Cancer *29*, S1.
34. Spiegelberg, L., Houben, R., Niemans, R., de Ruysscher, D., Yaromina, A., Theys, J., Guise, C.P., Smaill, J.B., Patterson, A.V., Lambin, P., and Dubois, L.J. (2019). Hypoxia-activated prodrugs and (lack of) clinical progress: The need for hypoxia-based biomarker patient selection in phase III clinical trials. Clin. Transl. Radiat. Oncol. *15*, 62–69.
35. Harris, B., Saleem, S., Cook, N., and Searle, E. (2022). Targeting hypoxia in solid and haematological malignancies. J. Exp. Clin. Cancer Res. *41*, 318.

36. Anduran, E., Dubois, L.J., Lambin, P., and Winum, J.-Y. (2022). Hypoxia-activated prodrug derivatives of anti-cancer drugs: a patent review 2006 – 2021. Expert Opin. Ther. Pat. *32*, 1–12.

37. Wigerup, C., Påhlman, S., and Bexell, D. (2016). Therapeutic targeting of hypoxia and hypoxia-inducible factors in cancer. Pharmacol. Ther. *164*, 152–169.

38. Misry, I.N., Thomas, M., Calder, E.D., Conway, S.J., and Hammond, E.M. (2017). Clinical Advances of Hypoxia-Activated Prodrugs in Combination With Radiation Therapy. Int. J. Radiat. Oncol. Biol. Phys. *98*, 1183–1196.

39. Bernauer, C., Man, Y.K.S., Chisholm, J.C., Lepicard, E.Y., Robinson, S.P., and Shipley, J.M. (2021). Hypoxia and its therapeutic possibilities in paediatric cancers. Br. J. Cancer *124*, 539–551.

40. Wilson, W.R., and Hay, M.P. (2011). Targeting hypoxia in cancer therapy. Nat. Rev. Cancer *11*, 393–410.

41. Harris, B.H.L., Barberis, A., West, C.M.L., and Buffa, F.M. (2015). Gene Expression Signatures as Biomarkers of Tumour Hypoxia. Clin. Oncol. *27*, 547–560.

42. Bhandari, V., Hoey, C., Liu, L.Y., Lalonde, E., Ray, J., Livingstone, J., Lesurf, R., Shiah, Y.J., Vujcic, T., Huang, X., et al. (2019). Molecular landmarks of tumor hypoxia across cancer types. Nat. Genet. *51*, 308–318.

43. Fox, N.S., Starmans, M.H.W., Haider, S., Lambin, P., and Boutros, P.C. (2014). Ensemble analyses improve signatures of tumour hypoxia and reveal inter-platform differences. BMC Bioinf. *15*, 170.

44. Dhawan, A., Barberis, A., Cheng, W.C., Domingo, E., West, C., Maughan, T., Scott, J.G., Harris, A.L., and Buffa, F.M. (2019). Guidelines for using sigQC for systematic evaluation of gene signatures. Nat. Protoc. *14*, 1377–1400.

45. Venet, D., Dumont, J.E., and Detours, V. (2011). Most Random Gene Expression Signatures Are Significantly Associated with Breast Cancer Outcome. PLoS Comput. Biol. *7*, e1002240.

46. Shimoni, Y. (2018). Association between expression of random gene sets and survival is evident in multiple cancer types and may be explained by sub-classification. PLoS Comput. Biol. *14*, e1006026.

47. Manjang, K., Tripathi, S., Yli-Harja, O., Dehmer, M., Glazko, G., and Emmert-Streib, F. (2021). Prognostic gene expression signatures of breast cancer are lacking a sensible biological meaning. Sci. Rep. *11*, 156.

48. Starmans, M.H.W., Chu, K.C., Haider, S., Nguyen, F., Seigneuric, R., Magagnin, M.G., Koritzinsky, M., Kasprzyk, A., Boutros, P.C., Wouters, B.G., and Lambin, P. (2012). The prognostic value of temporal in vitro and in vivo derived hypoxia gene-expression signatures in breast cancer. Radiother. Oncol. *102*, 436–443.

49. Sun, X., Luo, H., Han, C., Zhang, Y., and Yan, C. (2021). Identification of a Hypoxia-Related Molecular Classification and Hypoxic Tumor Microenvironment Signature for Predicting the Prognosis of Patients with Triple-Negative Breast Cancer. Front. Oncol. *11*, 700062.

50. Xu, Y., Cao, C., Zhu, Z., Wang, Y., Tan, Y., and Xu, X. (2022). Novel Hypoxia-Associated Gene Signature Depicts Tumor Immune Microenvironment and Predicts Prognosis of Colon Cancer Patients. Front. Genet. *13*, 901734.

51. Zhang, B., Tang, B., Gao, J., Li, J., Kong, L., and Qin, L. (2020). A hypoxia-related signature for clinically predicting diagnosis, prognosis and immune microenvironment of hepatocellular carcinoma patients. J. Transl. Med. *18*, 342.

52. Koong, A.C., Denko, N.C., Hudson, K.M., Schindler, C., Swiersz, L., Koch, C., Evans, S., Ibrahim, H., Le, Q.T., Terris, D.J., and Giaccia, A.J. (2000). Candidate genes for the hypoxic tumor phenotype. Cancer Res. *60*, 883–887.

53. Scandurro, A.B., Weldon, C.W., Figueroa, Y.G., Alam, J., and Beckman, B.S. (2001). Gene microarray analysis reveals a novel hypoxia signal transduction pathway in human hepatocellular carcinoma cells. Int. J. Oncol. *19*, 129–135.

54. Wykoff, C.C., Pugh, C.W., Maxwell, P.H., Harris, A.L., and Ratcliffe, P.J. (2000). Identification of novel hypoxia dependent and independent target genes of the von Hippel-Lindau (VHL) tumour suppressor by mRNA differential expression profiling. Oncogene *19*, 6297–6305.

55. Denko, N., Schindler, C., Koong, A., Laderoute, K., Green, C., and Giaccia, A. (2000). Epigenetic Regulation of Gene Expression in Cervical Cancer Cells by the Tumor Microenvironment. Clin. Cancer Res. *6*, 480–487.

56. Denko, N.C., Fontana, L.A., Hudson, K.M., Sutphin, P.D., Raychaudhuri, S., Altman, R., and Giaccia, A.J. (2003). Investigating hypoxic tumor physiology through gene expression patterns. Oncogene *22*, 5907–5914.

57. Jögi, A., Vallon-Christersson, J., Holmquist, L., Axelson, H., Borg, A., and Påhlman, S. (2004). Human neuroblastoma cells exposed to hypoxia: induction of genes associated with growth, survival, and aggressive behavior. Exp. Cell Res. *295*, 469–487.

58. Ning, W., Chu, T.J., Li, C.J., Choi, A.M.K., and Peters, D.G. (2004). Genome-wide analysis of the endothelial transcriptome under short-term chronic hypoxia. Physiol. Genomics *18*, 70–78.

59. Manalo, D.J., Rowan, A., Lavoie, T., Natarajan, L., Kelly, B.D., Ye, S.Q., Garcia, J.G.N., and Semenza, G.L. (2005). Transcriptional regulation of vascular endothelial cell responses to hypoxia by HIF-1. Blood *105*, 659–669.

60. Wang, V., Davis, D.A., Haque, M., Huang, L.E., and Yarchoan, R. (2005). Differential gene up-regulation by hypoxia-inducible factor-1alpha and hypoxia-inducible factor-2alpha in HEK293T cells. Cancer Res. *65*, 3299–3306.

61. Detwiller, K.Y., Fernando, N.T., Segal, N.H., Ryeom, S.W., D'Amore, P.A., and Yoon, S.S. (2005). Analysis of hypoxia-related gene expression in sarcomas and effect of hypoxia on RNA interference of vascular endothelial cell growth factor A. Cancer Res. *65*, 5881–5889.

62. Chi, J.-T., Wang, Z., Nuyten, D.S.A., Rodriguez, E.H., Schaner, M.E., Salim, A., Wang, Y., Kristensen, G.B., Helland, A., Børresen-Dale, A.L., et al. (2006). Gene expression programs in response to hypoxia: cell type specificity and prognostic significance in human cancers. PLoS Med. *3*, e47.

63. Mense, S.M., Sengupta, A., Zhou, M., Lan, C., Bentsman, G., Volsky, D.J., and Zhang, L. (2006). Gene expression profiling reveals the profound upregulation of hypoxia-responsive genes in primary human astrocytes. Physiol. Genomics *25*, 435–449.

64. Elvidge, G.P., Glenny, L., Appelhoff, R.J., Ratcliffe, P.J., Ragoussis, J., and Gleadle, J.M. (2006). Concordant regulation of gene expression by hypoxia and 2-oxoglutarate-dependent dioxygenase inhibition: the role of HIF-1alpha, HIF-2alpha, and other pathways. J. Biol. Chem. *281*, 15215–15226.

65. Peters, D.G., Ning, W., Chu, T.J., Li, C.J., and Choi, A.M.K. (2006). Comparative SAGE analysis of the response to hypoxia in human pulmonary and aortic endothelial cells. Physiol. Genomics *26*, 99–108.

66. Aprelikova, O., Wood, M., Tackett, S., Chandramouli, G.V.R., and Barrett, J.C. (2006). Role of ETS transcription factors in the hypoxia-inducible factor-2 target gene selection. Cancer Res. *66*, 5641–5647.

67. Bosco, M.C., Puppo, M., Santangelo, C., Anfosso, L., Pfeffer, U., Fardin, P., Battaglia, F., and Varesio, L. (2006). Hypoxia modifies the transcriptome of primary human monocytes: modulation of novel immune-related genes and identification of CC-chemokine ligand 20 as a new hypoxia-inducible gene. J. Immunol. *177*, 1941–1955.

68. Shi, Y.-F., Fong, C.C., Zhang, Q., Cheung, P.Y., Tzang, C.H., Wu, R.S.S., and Yang, M. (2007). Hypoxia induces the activation of human hepatic stellate cells LX-2 through TGF-beta signaling pathway. FEBS Lett. *581*, 203–210.

69. Sung, F.L., Hui, E.P., Tao, Q., Li, H., Tsui, N.B.Y., Lo, Y.M.D., Ma, B.B.Y., To, K.F., Harris, A.L., and Chan, A.T.C. (2007). Genome-wide expression analysis using microarray identified complex signaling pathways modulated by hypoxia in nasopharyngeal carcinoma. Cancer Lett. *253*, 74–88.

70. Winter, S.C., Buffa, F.M., Silva, P., Miller, C., Valentine, H.R., Turley, H., Shah, K.A., Cox, G.J., Corbridge, R.J., Homer, J.J., et al. (2007). Relation

of a hypoxia metagene derived from head and neck cancer to prognosis of multiple cancers. Cancer Res. *67*, 3441–3449.

71. Seigneuric, R., Starmans, M.H.W., Fung, G., Krishnapuram, B., Nuyten, D.S.A., van Erk, A., Magagnin, M.G., Rouschop, K.M., Krishnan, S., Rao, R.B., et al. (2007). Impact of supervised gene signatures of early hypoxia on patient survival. Radiother. Oncol. *83*, 374–382.
72. Beyer, S., Kristensen, M.M., Jensen, K.S., Johansen, J.V., and Staller, P. (2008). The histone demethylases JMJD1A and JMJD2B are transcriptional targets of hypoxia-inducible factor HIF. J. Biol. Chem. *283*, 36542–36552.
73. Hu, Z., Fan, C., Livasy, C., He, X., Oh, D.S., Ewend, M.G., Carey, L.A., Subramanian, S., West, R., Ikpatt, F., et al. (2009). A compact VEGF signature associated with distant metastases and poor outcomes. BMC Med. *7*, 9.
74. Benita, Y., Kikuchi, H., Smith, A.D., Zhang, M.Q., Chung, D.C., and Xavier, R.J. (2009). An integrative genomics approach identifies Hypoxia Inducible Factor-1 (HIF-1)-target genes that form the core response to hypoxia. Nucleic Acids Res. *37*, 4587–4602.
75. Fardin, P., Barla, A., Mosci, S., Rosasco, L., Verri, A., and Varesio, L. (2009). The l1-l2 regularization framework unmasks the hypoxia signature hidden in the transcriptome of a set of heterogeneous neuroblastoma cell lines. BMC Genom. *10*, 474.
76. Lendahl, U., Lee, K.L., Yang, H., and Poellinger, L. (2009). Generating specificity and diversity in the transcriptional response to hypoxia. Nat. Rev. Genet. *10*, 821–832.
77. Buffa, F.M., Harris, A.L., West, C.M., and Miller, C.J. (2010). Large meta-analysis of multiple cancers reveals a common, compact and highly prognostic hypoxia metagene. Br. J. Cancer *102*, 428–435.
78. Ghorbel, M.T., Cherif, M., Jenkins, E., Mokhtari, A., Kenny, D., Angelini, G.D., and Caputo, M. (2010). Transcriptomic analysis of patients with tetralogy of Fallot reveals the effect of chronic hypoxia on myocardial gene expression. J. Thorac. Cardiovasc. Surg. *140*, 337–345.e26.
79. Sørensen, B.S., Toustrup, K., Horsman, M.R., Overgaard, J., and Alsner, J. (2010). Identifying pH independent hypoxia induced genes in human squamous cell carcinomas in vitro. Acta Oncol. *49*, 895–905.
80. van Malenstein, H., Gevaert, O., Libbrecht, L., Daemen, A., Allemeersch, J., Nevens, F., Van Cutsem, E., Cassiman, D., De Moor, B., Verslype, C., and van Pelt, J. (2010). A seven-gene set associated with chronic hypoxia of prognostic importance in hepatocellular carcinoma. Clin. Cancer Res. *16*, 4278–4288.
81. Fardin, P., Barla, A., Mosci, S., Rosasco, L., Verri, A., Versteeg, R., Caron, H.N., Molenaar, J.J., Ora, I., Eva, A., et al. (2010). A biology-driven approach identifies the hypoxia gene signature as a predictor of the outcome of neuroblastoma patients. Mol. Cancer *9*, 185.
82. Ghazoui, Z., Buffa, F.M., Dunbier, A.K., Anderson, H., Dexter, T., Detre, S., Salter, J., Smith, I.E., Harris, A.L., and Dowsett, M. (2011). Close and Stable Relationship between Proliferation and a Hypoxia Metagene in Aromatase Inhibitor–Treated ER-Positive Breast Cancer. Clin. Cancer Res. *17*, 3005–3012.
83. Toustrup, K., Sørensen, B.S., Nordsmark, M., Busk, M., Wiuf, C., Alsner, J., and Overgaard, J. (2011). Development of a hypoxia gene expression classifier with predictive impact for hypoxic modification of radiotherapy in head and neck cancer. Cancer Res. *71*, 5923–5931.
84. Halle, C., Andersen, E., Lando, M., Aarnes, E.K., Hasvold, G., Holden, M., Syljuåsen, R.G., Sundfør, K., Kristensen, G.B., Holm, R., et al. (2012). Hypoxia-induced gene expression in chemoradioresistant cervical cancer revealed by dynamic contrast-enhanced MRI. Cancer Res. *72*, 5285–5295.
85. Eustace, A., Mani, N., Span, P.N., Irlam, J.J., Taylor, J., Betts, G.N.J., Denley, H., Miller, C.J., Homer, J.J., Rojas, A.M., et al. (2013). A 26-gene hypoxia signature predicts benefit from hypoxia-modifying therapy in laryngeal cancer but not bladder cancer. Clin. Cancer Res. *19*, 4879–4888.
86. Boidot, R., Branders, S., Helleputte, T., Rubio, L.I., Dupont, P., and Feron, O. (2014). A generic cycling hypoxia-derived prognostic gene signature: application to breast cancer profiling. Oncotarget *5*, 6947–6963.
87. Ragnum, H.B., Vlatkovic, L., Lie, A.K., Axcrona, K., Julin, C.H., Frikstad, K.M., Hole, K.H., Seierstad, T., and Lyng, H. (2015). The tumour hypoxia marker pimonidazole reflects a transcriptional programme associated with aggressive prostate cancer. Br. J. Cancer *112*, 382–390.
88. Fjeldbo, C.S., Julin, C.H., Lando, M., Forsberg, M.F., Aarnes, E.K., Alsner, J., Kristensen, G.B., Malinen, E., and Lyng, H. (2016). Integrative Analysis of DCE-MRI and Gene Expression Profiles in Construction of a Gene Classifier for Assessment of Hypoxia-Related Risk of Chemoradiotherapy Failure in Cervical Cancer. Clin. Cancer Res. *22*, 4067–4076.
89. Suh, Y.-E., Lawler, K., Henley-Smith, R., Pike, L., Leek, R., Barrington, S., Odell, E.W., Ng, T., Pezzella, F., Guerrero-Urbano, T., and Tavassoli, M. (2017). Association between hypoxic volume and underlying hypoxia-induced gene expression in oropharyngeal squamous cell carcinoma. Br. J. Cancer *116*, 1057–1064.
90. Yang, L., Taylor, J., Eustace, A., Irlam, J.J., Denley, H., Hoskin, P.J., Alsner, J., Buffa, F.M., Harris, A.L., Choudhury, A., and West, C.M.L. (2017). A Gene Signature for Selecting Benefit from Hypoxia Modification of Radiotherapy for High-Risk Bladder Cancer Patients. Clin. Cancer Res. *23*, 4761–4768.
91. Ye, I.C., Fertig, E.J., DiGiacomo, J.W., Considine, M., Godet, I., and Gilkes, D.M. (2018). Molecular Portrait of Hypoxia in Breast Cancer: A Prognostic Signature and Novel HIF-Regulated Genes. Mol. Cancer Res. *16*, 1889–1901.
92. Yang, L., Roberts, D., Takhar, M., Erho, N., Bibby, B.A., Thiruthaneeswaran, N., Bhandari, V., Cheng, W.C., Haider, S., McCorry, A.M., et al. (2018). Development and Validation of a 28-gene Hypoxia-related Prognostic Signature for Localized. Prostate Cancer. EBioMedicine *31*, 182–189.
93. Yang, L., Forker, L., Irlam, J.J., Pillay, N., Choudhury, A., and West, C.M.L. (2018). Validation of a hypoxia related gene signature in multiple soft tissue sarcoma cohorts. Oncotarget *9*, 3946–3955.
94. Dao, T.P., Rösch, S., Mairbäurl, H., Pusch, S., Unterberg, A., Herold-Mende, C., and Warta, R. (2018). Identification of a Prognostic Hypoxia-Associated Gene Set in IDH-Mutant Glioma. Int. J. Mol. Sci. *19*, 2903.
95. Chen, Y.L., Zhang, Y., Wang, J., Chen, N., Fang, W., Zhong, J., Liu, Y., Qin, R., Yu, X., Sun, Z., and Gao, F. (2019). A 17 gene panel for non-small-cell lung cancer prognosis identified through integrative epigenomic-transcriptomic analyses of hypoxia-induced epithelial-mesenchymal transition. Mol. Oncol. *13*, 1490–1502.
96. Zou, Y.F., Rong, Y.M., Tan, Y.X., Xiao, J., Yu, Z.L., Chen, Y.F., Ke, J., Li, C.H., Chen, X., Wu, X.J., et al. (2019). A signature of hypoxia-related factors reveals functional dysregulation and robustly predicts clinical outcomes in stage I/II colorectal cancer patients. Cancer Cell Int. *19*, 243.
97. Wang, J., Wang, Y., Xing, P., Liu, Q., Zhang, C., Sui, Y., and Wu, C. (2020). Development and validation of a hypoxia-related prognostic signature for breast cancer. Oncol. Lett. *20*, 1906–1914.
98. Shou, Y., Yang, L., Yang, Y., Zhu, X., Li, F., and Xu, J. (2020). Identification of Signatures of Prognosis Prediction for Melanoma Using a Hypoxia Score. Front. Genet. *11*, 570530.
99. Lin, W., Wu, S., Chen, X., Ye, Y., Weng, Y., Pan, Y., Chen, Z., Chen, L., Qiu, X., and Qiu, S. (2020). Characterization of Hypoxia Signature to Evaluate the Tumor Immune Microenvironment and Predict Prognosis in Glioma Groups. Front. Oncol. *10*, 796.
100. Mo, Z., Yu, L., Cao, Z., Hu, H., Luo, S., and Zhang, S. (2020). Identification of a Hypoxia-Associated Signature for Lung Adenocarcinoma. Front. Genet. *11*, 647.
101. Sun, J., Zhao, T., Zhao, D., Qi, X., Bao, X., Shi, R., and Su, C. (2020). Development and validation of a hypoxia-related gene signature to

predict overall survival in early-stage lung adenocarcinoma patients. Ther. Adv. Med. Oncol. *12*, 1758835920937904.

102. Calvo, T.M., Marinari, E., Migliorini, D., Bes, V., Tankov, S., Charrier, E., McKee, T.A., Dutoit, V., Dietrich, P.Y., Cosset, E., and Walker, P.R. (2020). An Experimentally Defined Hypoxia Gene Signature in Glioblastoma and Its Modulation by Metformin. Biology *9*, 264.

103. Puente-Santamaría, L., Sanchez-Gonzalez, L., Ramos-Ruiz, R., and Del Peso, L. (2022). Hypoxia classifier for transcriptome datasets. BMC Bioinf. *23*, 204.

104. Wang, K., Lu, Y., Liu, Z., Diao, M., and Yang, L. (2022). Establishment and External Validation of a Hypoxia-Derived Gene Signature for Robustly Predicting Prognosis and Therapeutic Responses in Glioblastoma Multiforme. BioMed Res. Int. *2022*, 7858477.

105. Lane, B., Khan, M.T., Choudhury, A., Salem, A., and West, C.M.L. (2022). Development and validation of a hypoxia-associated signature for lung adenocarcinoma. Sci. Rep. *12*, 1290.

106. Gao, F., Wang, Z., Gu, J., Zhang, X., and Wang, H. (2021). A Hypoxia-Associated Prognostic Gene Signature Risk Model and Prognosis Predictors in Gliomas. Front. Oncol. *11*, 726794.

107. Abou Khouzam, R., Rao, S.P., Venkatesh, G.H., Zeinelabdin, N.A., Buart, S., Meylan, M., Nimmakayalu, M., Terry, S., and Chouaib, S. (2021). An Eight-Gene Hypoxia Signature Predicts Survival in Pancreatic Cancer and Is Associated With an Immunosuppressed Tumor Microenvironment. Front. Immunol. *12*, 680435.

108. Shou, Y., Yang, L., Yang, Y., Zhu, X., Li, F., and Xu, J. (2021). Determination of hypoxia signature to predict prognosis and the tumor immune microenvironment in melanoma. Mol. Omics *17*, 307–316.

109. Zhang, Q., Qiao, L., Liao, J., Liu, Q., Liu, P., and Liu, L. (2021). A novel hypoxia gene signature indicates prognosis and immune microenvironments characters in patients with hepatocellular carcinoma. J. Cell Mol. Med. *25*, 3772–3784.

110. Shi, R., Bao, X., Unger, K., Sun, J., Lu, S., Manapov, F., Wang, X., Belka, C., and Li, M. (2021). Identification and validation of hypoxia-derived gene signatures to predict clinical outcomes and therapeutic responses in stage I lung adenocarcinoma patients. Theranostics *11*, 5061–5076.

111. Liu, L., Zhu, H., Wang, P., and Wu, S. (2022). Construction of a Six-Gene Prognostic Risk Model Related to Hypoxia and Angiogenesis for Cervical Cancer. Front. Genet. *13*, 923263.

112. Wei, C., Liu, X., Wang, Q., Li, Q., and Xie, M. (2021). Identification of Hypoxia Signature to Assess the Tumor Immune Microenvironment and Predict Prognosis in Patients with Ovarian Cancer. Int. J. Endocrinol. *2021*, 4156187.

113. He, X., Ding, J., Cheng, X., and Xiong, M. (2021). Hypoxia-Related Gene-Based Signature Can Evaluate the Tumor Immune Microenvironment and Predict the Prognosis of Colon Adenocarcinoma Patients. Int. J. Gen. Med. *14*, 9853–9862.

114. Xia, H., Wang, J., Guo, X., Lv, Z., Liu, J., Yan, Q., Liu, M., and Wang, J. (2021). Identification of a Hypoxia-Related Gene Signature for Predicting Systemic Metastasis in Prostate Cancer. Front. Cell Dev. Biol. *9*, 696364.

115. Li, Q., Jin, L., and Jin, M. (2021). Novel Hypoxia-Related Gene Signature for Risk Stratification and Prognosis in Hepatocellular Carcinoma. Front. Genet. *12*, 613890.

116. Zhao, C., Zhou, Y., Ma, H., Wang, J., Guo, H., and Liu, H. (2021). A four-hypoxia-genes-based prognostic signature for oral squamous cell carcinoma. BMC Oral Health *21*, 232.

117. Lombardi, O., Li, R., Halim, S., Choudhry, H., Ratcliffe, P.J., and Mole, D.R. (2022). Pan-cancer analysis of tissue and single-cell HIF-pathway activation using a conserved gene signature. Cell Rep. *41*, 111652.

118. Kaelin, W.G. (2007). The von Hippel-Lindau tumor suppressor protein and clear cell renal carcinoma. Clin. Cancer Res. *13*, 680s–684s.

119. Yorimitsu, T., Nair, U., Yang, Z., and Klionsky, D.J. (2006). Endoplasmic reticulum stress triggers autophagy. J. Biol. Chem. *281*, 30299–30304.

120. Catrina, S.B., and Zheng, X. (2021). Hypoxia and hypoxia-inducible factors in diabetes and its complications. Diabetologia *64*, 709–716.

121. LaGory, E.L., and Giaccia, A.J. (2017). Long-range hypoxia signaling in NAFLD. Nat. Med. *23*, 1251–1252.

122. Pinilla, L.L., Ugun-Klusek, A., Rutella, S., and De Girolamo, L.A. (2021). Hypoxia Signaling in Parkinson's Disease: There Is Use in Asking 'What HIF? Biology *10*.

123. Ramljak, S., Herlyn, H., and Zerr, I. (2016). Cellular Prion Protein (PrPc) and Hypoxia: True to Each Other in Good Times and in Bad, in Sickness, and in Health. Front. Cell. Neurosci. *10*, 292.

124. Rzymski, T., Milani, M., Pike, L., Buffa, F., Mellor, H.R., Winchester, L., Pires, I., Hammond, E., Ragoussis, I., and Harris, A.L. (2010). Regulation of autophagy by ATF4 in response to severe hypoxia. Oncogene *29*, 4424–4435.

125. Leithner, K., Wohlkoenig, C., Stacher, E., Lindenmann, J., Hofmann, N.A., Gallé, B., Guelly, C., Quehenberger, F., Stiegler, P., Smolle-Jüttner, F.M., et al. (2014). Hypoxia increases membrane metallo-endopeptidase expression in a novel lung cancer ex vivo model - role of tumor stroma cells. BMC Cancer *14*, 40.

126. Lähnemann, D., Köster, J., Szczurek, E., McCarthy, D.J., Hicks, S.C., Robinson, M.D., Vallejos, C.A., Campbell, K.R., Beerenwinkel, N., Mahfouz, A., et al. (2020). Eleven grand challenges in single-cell data science. Genome Biol. *21*, 1–35.

127. Semenza, G.L. (2010). Defining the role of hypoxia-inducible factor 1 in cancer biology and therapeutics. Oncogene *29*, 625–634.

128. Jing, X., Yang, F., Shao, C., Wei, K., Xie, M., Shen, H., and Shu, Y. (2019). Role of hypoxia in cancer therapy by regulating the tumor microenvironment. Mol. Cancer *18*, 157.

129. Thomson, D.J., Slevin, N.J., Baines, H., Betts, G., Bolton, S., Evans, M., Garcez, K., Irlam, J., Lee, L., Melillo, N., et al. (2024). Randomized Phase 3 Trial of the Hypoxia Modifier Nimorazole Added to Radiation Therapy With Benefit Assessed in Hypoxic Head and Neck Cancers Determined Using a Gene Signature (NIMRAD). Int. J. Radiat. Oncol. Biol. Phys. *119*, 771–782.

130. Sparano, J.A., Gray, R.J., Ravdin, P.M., Makower, D.F., Pritchard, K.I., Albain, K.S., Hayes, D.F., Geyer, C.E., Jr., Dees, E.C., Goetz, M.P., et al. (2019). Clinical and Genomic Risk to Guide the Use of Adjuvant Therapy for Breast Cancer. N. Engl. J. Med. *380*, 2395–2405.

131. Piccart, M., van 't Veer, L.J., Poncet, C., Lopes Cardozo, J.M.N., Delaloge, S., Pierga, J.Y., Vuylsteke, P., Brain, E., Vrijaldenhoven, S., Neijenhuis, P.A., et al. (2021). 70-gene signature as an aid for treatment decisions in early breast cancer: updated results of the phase 3 randomised MINDACT trial with an exploratory analysis by age. Lancet Oncol. *22*, 476–488.

132. Liu, J., Lichtenberg, T., Hoadley, K.A., Poisson, L.M., Lazar, A.J., Cherniack, A.D., Kovatich, A.J., Benz, C.C., Levine, D.A., Lee, A.V., et al. (2018). An Integrated TCGA Pan-Cancer Clinical Data Resource to Drive High-Quality Survival Outcome Analytics. Cell *173*, 400–416.e11.

133. Hoadley, K.A., Yau, C., Wolf, D.M., Cherniack, A.D., Tamborero, D., Ng, S., Leiserson, M.D.M., Niu, B., McLellan, M.D., Uzunangelov, V., et al. (2014). Multiplatform analysis of 12 cancer types reveals molecular classification within and across tissues of origin. Cell *158*, 929–944.

134. Huo, D., Hu, H., Rhie, S.K., Gamazon, E.R., Cherniack, A.D., Liu, J., Yoshimatsu, T.F., Pitt, J.J., Hoadley, K.A., Troester, M., et al. (2017). Comparison of Breast Cancer Molecular Features and Survival by African and European Ancestry in The Cancer Genome Atlas. JAMA Oncol. *3*, 1654–1662.

135. Miller, L.D., Smeds, J., George, J., Vega, V.B., Vergara, L., Ploner, A., Pawitan, Y., Hall, P., Klaar, S., Liu, E.T., and Bergh, J. (2005). An expression signature for p53 status in human breast cancer predicts mutation status, transcriptional effects, and patient survival. Proc. Natl. Acad. Sci. USA *102*, 13550–13555.

136. Wang, Y., Klijn, J.G.M., Zhang, Y., Sieuwerts, A.M., Look, M.P., Yang, F., Talantov, D., Timmermans, M., Meijer-van Gelder, M.E., Yu, J., et al. (2005). Gene-expression profiles to predict distant metastasis of lymph-node-negative primary breast cancer. Lancet *365*, 671–679.

137. Kawashima, M., Bensaad, K., Zois, C.E., Barberis, A., Bridges, E., Wigfield, S., Lagerholm, C., Dmitriev, R.I., Tokiwa, M., Toi, M., et al. (2020). Disruption of hypoxia-inducible fatty acid binding protein 7 induces beige fat-like differentiation and thermogenesis in breast cancer cells. Cancer Metab. *8*, 13.

138. Kyerewah-Kersi, S.M., Khan, E., Venkatesh, G.H., and Abou Khouzam, R. (2023). Hypoxia is Associated with Increased Expression of APOBEC Genes in Breast Cancer. Hamdan Medical Journal *16*, 94–100.

139. Xiong, Z., Huang, W., Zhong, W., Fu, J., Feng, J., Wang, X., and Ling, F. (2022). Breast Cancer Subtypes Based on Hypoxia-Related Gene Sets Identify Potential Therapeutic Agents. Front. Mol. Biosci. *9*, 900005.

140. Beer, D.G., Kardia, S.L.R., Huang, C.C., Giordano, T.J., Levin, A.M., Misek, D.E., Lin, L., Chen, G., Gharib, T.G., Thomas, D.G., et al. (2002). Gene-expression profiles predict survival of patients with lung adenocarcinoma. Nat. Med. *8*, 816–824.

141. Zhuang, H., Wang, S., Chen, B., Zhang, Z., Ma, Z., Li, Z., Liu, C., Zhou, Z., Gong, Y., Huang, S., et al. (2021). Prognostic Stratification Based on HIF-1 Signaling for Evaluating Hypoxic Status and Immune Infiltration in Pancreatic Ductal Adenocarcinomas. Front. Immunol. *12*, 790661.

142. Xu, Z., Li, X.-F., Zou, H., Sun, X., and Shen, B. (2017). 18F-Fluoromisonidazole in tumor hypoxia imaging. Oncotarget *8*, 94969–94979.

143. Mo, F., Lin, D., Takhar, M., Ramnarine, V.R., Dong, X., Bell, R.H., Volik, S.V., Wang, K., Xue, H., Wang, Y., et al. (2018). Stromal Gene Expression is Predictive for Metastatic Primary Prostate Cancer. Eur. Urol. *73*, 524–532.

144. Zare, A., Postovit, L.-M., and Githaka, J.M. (2021). Robust inflammatory breast cancer gene signature using nonparametric random forest analysis. Breast Cancer Res. *23*, 92.

145. Dawany, N., Showe, L.C., Kossenkov, A.V., Chang, C., Ive, P., Conradie, F., Stevens, W., Sanne, I., Azzoni, L., and Montaner, L.J. (2014). Identification of a 251 gene expression signature that can accurately detect M. tuberculosis in patients with and without HIV co-infection. PLoS One *9*, e89925.

146. Durinck, S., Spellman, P.T., Birney, E., and Huber, W. (2009). Mapping identifiers for the integration of genomic datasets with the R/Bioconductor package biomaRt. Nat. Protoc. *4*, 1184–1191.

147. Multi-symbol checker. https://www.genenames.org/tools/multi-symbol-checker/.

148. Stelzer, G., Rosen, R., Plaschkes, I., Zimmerman, S., Twik, M., Fishilevich, S., Iny Stein, T., Nudel, R., Lieder, R., Mazor, Y., et al. (2016). GeneCards – the human gene database (www.genecards.org). Curr. Protoc. Bioinformatics *54*, 1.30.1–1.30.33.

149. Ge, S.X., Jung, D., and Yao, R. (2020). ShinyGO: a graphical gene-set enrichment tool for animals and plants. Bioinformatics *36*, 2628–2629.

150. Clough, E., and Barrett, T. (2016). The Gene Expression Omnibus Database. Methods Mol. Biol. *1418*, 93–110.

151. Ledaki, I., McIntyre, A., Wigfield, S., Buffa, F., McGowan, S., Baban, D., Li, J.L., and Harris, A.L. (2015). Carbonic anhydrase IX induction defines a heterogeneous cancer cell response to hypoxia and mediates stem cell-like properties and sensitivity to HDAC inhibition. Oncotarget *6*, 19413–19427.

152. Picelli, S., Björklund, Å.K., Faridani, O.R., Sagasser, S., Winberg, G., and Sandberg, R. (2013). Smart-seq2 for sensitive full-length transcriptome profiling in single cells. Nat. Methods *10*, 1096–1098.

153. De Sena Brandine, G., and Smith, A.D. (2019). Falco: high-speed FastQC emulation for quality control of sequencing data. F1000Res. *8*, 1874.

154. Martin, M. (2011). Cutadapt removes adapter sequences from high-throughput sequencing reads. EMBnet. j. *17*, 10–12.

155. Dobin, A., Davis, C.A., Schlesinger, F., Drenkow, J., Zaleski, C., Jha, S., Batut, P., Chaisson, M., and Gingeras, T.R. (2013). STAR: ultrafast universal RNA-seq aligner. Bioinformatics *29*, 15–21.

156. Liao, Y., Smyth, G.K., and Shi, W. (2014). featureCounts: an efficient general purpose program for assigning sequence reads to genomic features. Bioinformatics *30*, 923–930.

157. Ewels, P., Magnusson, M., Lundin, S., and Käller, M. (2016). MultiQC: summarize analysis results for multiple tools and samples in a single report. Bioinformatics *32*, 3047–3048.

158. Köster, J., and Rahmann, S. (2012). Snakemake–a scalable bioinformatics workflow engine. Bioinformatics *28*.

159. Hao, Y., Hao, S., Andersen-Nissen, E., Mauck, W.M., 3rd, Zheng, S., Butler, A., Lee, M.J., Wilk, A.J., Darby, C., Zager, M., et al. (2021). Integrated analysis of multimodal single-cell data. Cell *184*, 3573–3587.e29.

160. McInnes, L., Healy, J., Saul, N., and Großberger, L. (2018). UMAP: Uniform Manifold Approximation and Projection. J. Open Source Softw. *3*, 861.

161. Zappia, L., and Oshlack, A. (2018). Clustering trees: a visualization for evaluating clusterings at multiple resolutions. GigaScience *7*, giy083.

162. Tran, H.T.N., Ang, K.S., Chevrier, M., Zhang, X., Lee, N.Y.S., Goh, M., and Chen, J. (2020). A benchmark of batch-effect correction methods for single-cell RNA sequencing data. Genome Biol. *21*, 12.

163. Cancer Genome Atlas Research Network; Weinstein, J.N., Collisson, E.A., Mills, G.B., Shaw, K.R.M., Ozenberger, B.A., Ellrott, K., Shmulevich, I., Sander, C., and Stuart, J.M. (2013). The Cancer Genome Atlas Pan-Cancer analysis project. Nat. Genet. *45*, 1113–1120.

164. Boutros, P.C., Lau, S.K., Pintilie, M., Liu, N., Shepherd, F.A., Der, S.D., Tsao, M.S., Penn, L.Z., and Jurisica, I. (2009). Prognostic gene signatures for non-small-cell lung cancer. Proc. Natl. Acad. Sci. USA *106*, 2824–2828.

165. Starmans, M.H.W., Fung, G., Steck, H., Wouters, B.G., and Lambin, P. (2011). A simple but highly effective approach to evaluate the prognostic performance of gene expression signatures. PLoS One *6*, e28320.

166. Venet, D., Dumont, J.E., and Detours, V. (2011). Most random gene expression signatures are significantly associated with breast cancer outcome. PLoS Comput. Biol. *7*, e1002240.

167. Knijnenburg, T.A., Wessels, L.F.A., Reinders, M.J.T., and Shmulevich, I. (2009). Fewer permutations, more accurate P-values. Bioinformatics *25*, i161–i168.

168. Schefzik, R., Flesch, J., and Goncalves, A. (2021). Fast identification of differential distributions in single-cell RNA-sequencing data with waddR. Bioinformatics *37*, 3204–3211.

CellPress
OPEN ACCESS

STAR★METHODS

KEY RESOURCES TABLE

REAGENT or RESOURCE	SOURCE	IDENTIFIER
Deposited data		
Bulk gene expression from hypoxia/normoxia experiments (Breast, MCF-7)	Elvidge et al.	GEO: GSE3188
Bulk gene expression from hypoxia/normoxia experiments (Lung, A549)	Moreno Leon et al.	GEO: GSE117036
Bulk gene expression from hypoxia/normoxia experiments (Lung, A549)	Moreno Leon et al.	GEO: GSE117041
Bulk gene expression from hypoxia/normoxia experiments (Kidney, HKC8, RCC4; Liver, HepG2)	Smythies JA et al.<br>Schmid V et al.<br>Lauer V et al.	GEO: GSE120886
Bulk gene expression from hypoxia/normoxia experiments (Cervical, HeLa, SW756, C-33, C-41, ME-180, HT-3, SiHa, CaSki)	Fjeldbo CS et al.<br>Jonsson M et al.	GEO: GSE72723
Bulk gene expression from hypoxia/normoxia experiments (Cervical, HeLa, SiHa, CaSki)	Halle C et al.	GEO: GSE36562
Bulk gene expression from hypoxia/normoxia experiments (Breast, MDA-MB-231, LM2)	Goodarzi H et al.	GEO: GSE63562
Bulk gene expression from hypoxia/normoxia experiments (Liver, HepG2, Brain, U87, Breast, MDA-MB-231)	Xia X et al.	GEO: GSE18494
Bulk gene expression from hypoxia/normoxia experiments (Cervical, HeLa)	Lee DC et al.	GEO: GSE55211
Bulk gene expression from hypoxia/normoxia experiments (Liver, Huh-7)	Lee DC et al.	GEO: GSE55212
Bulk gene expression from hypoxia/normoxia experiments (Liver, Huh-7)	Lee DC et al.	GEO: GSE59729
Bulk gene expression from hypoxia/normoxia experiments (Breast, BT20, BT474, BT549, CAMA1, DU4475, HBL100, HCC1428, HCC1569, HCC1806, HCC1937, HCC38, HME2, HS578T, hTERT-HME, MCF10A, MCF12A, MCF-7, MDA-MB-157, MDA-MB-175, MDA-MB-231-PSOC, MDA-MB-436, MDA-MB-468, SKBR3, SUM1315, SUM149, SUM159, SUM185, SUM225CWN, SUM229, T-47D, ZR-75-1)	Ye IC et al.<br>Godet I et al.	GEO: GSE111653
Bulk gene expression from hypoxia/normoxia experiments (Colorectal, HCT116)	Galbraith MD et al.	GEO: GSE38061
Bulk gene expression from hypoxia/normoxia experiments (Breast, MCF10A, MDA-MB-231)	Sesé M et al.	GEO: GSE104193
Bulk gene expression from hypoxia/normoxia experiments (Prostate, PC-3 LNCaP)	Guo H et al.	GEO: GSE106305
Bulk gene expression from hypoxia/normoxia experiments (Breast MCF-7)	Ho JC et al.	GEO: GSE89891
Bulk gene expression from hypoxia/normoxia experiments (Prostate, PC-3, Ovarian, SK-OV-3, Skin, WM793B)	Olbryt M. et al.	GEO: GSE53012
Bulk gene expression from hypoxia/normoxia experiments (Colorectal, HCT116, Liver, HepG2)	Koritzinsky M et al.	GEO: GSE41666
Bulk gene expression from hypoxia/normoxia experiments (Prostate, DU145, Colorectal, HT29, Breast, MCF-7)	Koritzinsky M et al.	GEO: GSE41491
Bulk gene expression from hypoxia/normoxia experiments (Prostate, DU145, Colorectal, HT29, Breast, MCF-7)	Starmans MH et al.	GEO: GSE29641
Bulk gene expression from hypoxia/normoxia experiments (Breast, MDA-MB-231)	Chen Y et al.	GEO: GSE108833
Bulk gene expression from hypoxia/normoxia experiments (Gastric, Cancer-associated myofibroblasts gastric tumor,<br>Normal gastric myofibroblasts)	Najgebauer H et al.	GEO: GSE125177
Bulk gene expression from hypoxia/normoxia experiments (Breast, MCF-7)	Jarman EJ et al.	GEO: GSE111246
Bulk gene expression from hypoxia/normoxia experiments (Ovarian, SKOV3ip.1)	Wilson C et al.	GEO: GSE66894
Bulk gene expression from hypoxia/normoxia experiments (Breast, MCF-7)	Flamant L et al.	GEO: GSE39042
Bulk gene expression from hypoxia/normoxia experiments (Brain, NCH421k, NCH601, NCH644, NCH660h)	Dao Trong P et al.	GEO: GSE118683
Bulk gene expression from hypoxia/normoxia experiments (Colorectal, HCT116)	Memon D et al.	GEO: GSE81513
Bulk gene expression from hypoxia/normoxia experiments (Brain, U87)	Kucharzewska P et al.	GEO: GSE45301

(*Continued on next page*)

Continued

REAGENT or RESOURCE	SOURCE	IDENTIFIER
Bulk gene expression from hypoxia/normoxia experiments (Breast, MCF-7)	Tang X et al.	GEO: GSE29406
Bulk gene expression from hypoxia/normoxia experiments (Pancreas, FG, L3.6pL)	Camaj P et al.	GEO: GSE9350
Bulk gene expression from hypoxia/normoxia experiments (Ewing’s sarcoma, TC-252)	Aryee DN et al.	GEO: GSE19197
Bulk gene expression from hypoxia/normoxia experiments (Breast, MCF-7)	Yang J et al.	GEO: GSE61799
Bulk gene expression from hypoxia/normoxia experiments (Breast, MCF-7)	Lee JS et al.	GEO: GSE15530
Bulk gene expression from hypoxia/normoxia experiments (Esophagus, EPC2)	Lee JJ et al.	GEO: GSE17353
Bulk gene expression from hypoxia/normoxia experiments (Kidney, 786-O)	Chen et al.	GEO: GSE107848
Bulk gene expression from hypoxia/normoxia experiments (Lung, PC-9)	An SM et al.	GEO: GSE69599
Bulk gene expression from hypoxia/normoxia experiments (Lung, H460)	Ellinghaus P et al.	GEO: GSE42791
Bulk gene expression from hypoxia/normoxia experiments (Breast MCF-7, ZR-75-1)	Ellinghaus P et al.	GEO: GSE33438
Bulk gene expression from hypoxia/normoxia experiments (Kidney, A498)	Ackerman D et al.	GEO: GSE117775
Bulk gene expression from hypoxia/normoxia experiments (Gastric, MKN28)	Lim MMK et al.	GEO: GSE71280
Bulk gene expression from hypoxia/normoxia experiments (Brain, DAOY)	Mutvei AP et al.	GEO: GSE113353
Bulk gene expression from hypoxia/normoxia experiments (Prostate, LNCaP)	Labrecque MP et al.	GEO: GSE78245
Bulk gene expression from hypoxia/normoxia experiments (Colorectal, HCT116)	Skowronski K et al.	GEO: GSE58049
Bulk gene expression from hypoxia/normoxia experiments (Breast, MCF-7)	Thienpont B et al.	GEO: GSE71401
Bulk gene expression from hypoxia/normoxia experiments (Prostate, 22Rv1, LNCaP)	Ragnum HB et al.	GEO: GSE42868
Bulk gene expression from hypoxia/normoxia experiments (Breast, MCF-7)	Ikeda K et al.	GEO: GSE124524
Bulk gene expression from hypoxia/normoxia experiments (Colorectal, HCT116)	Bruno T et al.	GEO: GSE90599
Bulk gene expression from hypoxia/normoxia experiments (Breast, MB231RN-LM)	Krutilina R et al.	GEO: GSE45362
Bulk gene expression from hypoxia/normoxia experiments (Pancreas, PANC-1)	Dekervel J et al.	GEO: GSE82104
Bulk gene expression from hypoxia/normoxia experiments (Astrocytes from fetal brain)	Mense SM et al.	GEO: GSE3045
Bulk gene expression from hypoxia/normoxia experiments (Cervical, HeLa)	Mense SM et al.	GEO: GSE3051
Bulk gene expression from hypoxia/normoxia experiments (Breast, MCF-7)	Camps C et al.	GEO: GSE47533
Bulk gene expression from hypoxia/normoxia experiments (Colorectal, DKO3)	Wang L et al.	GEO: GSE35973
Bulk gene expression from hypoxia/normoxia experiments (Pancreas, AsPC-1)	Markolin P et al.	GEO: GSE139673
Bulk gene expression from hypoxia/normoxia experiments (Breast, UFH-001)	Mboge MY et al.	GEO: GSE123856
Bulk gene expression from hypoxia/normoxia experiments (Pancreas, A10.7, A125, A13D, A2.4, A32.4, A38.41, A38.44, A38.5, A6L)	Zong Y et al.	GEO: GSE67549
Bulk gene expression from hypoxia/normoxia experiments (Breast: MCF-7, MDA-MB-231, T-47D; Lung: A549; Colorectal: WiDr, HCT116, HT29, COLO-205, LoVo, HCT-15; Cervical: SiHa<br>Prostate: PC-3; Brain: U373; Liver: HepG2, Hep3B, PLC-PRF-5, SK-HEP-1; Kidney: A498; Fibrosarcoma: HT1080	Boidot R et al.	GEO: GSE42416
Bulk gene expression from hypoxia/normoxia experiments (Breast, MCF10A)	De Troyer L et al.	GEO: GSE129344
Bulk gene expression from hypoxia/normoxia experiments (Ovarian, A2780)	Rupaimoole R et al.	GEO: GSE52695
Bulk gene expression from hypoxia/normoxia experiments (Burkitt lymphoma, P493-6)	Kim JW et al.	GEO: GSE4086
Bulk gene expression from hypoxia/normoxia experiments (Breast, MCF-7)	Kreuzer M et al.	GEO: GSE111259
Bulk gene expression from hypoxia/normoxia experiments (Cervical, HeLa, SiHa)	Hillestad T et al.	GEO: GSE147384
Bulk gene expression from hypoxia/normoxia experiments (Breast, HCC1806, MCF-7)	Ahuja N et al.	GEO: GSE147516
Bulk gene expression from hypoxia/normoxia experiments (Breast, MCF-7)	Wu X et al.	GEO: GSE153291
Bulk gene expression from hypoxia/normoxia experiments (Breast, MCF-7; Skin, SK-MEL-28; Kidney: RCC4)	D’Anna F et al.	GEO: GSE85353
Bulk gene expression from hypoxia/normoxia experiments (Breast, T-47D)	Jewer M et al.	GEO: GSE149132

(*Continued on next page*)

Continued

REAGENT or RESOURCE	SOURCE	IDENTIFIER
Bulk gene expression from hypoxia/normoxia experiments (Liver, SMMC-7721)	Hou J et al.	GEO: GSE120611
Bulk gene expression from hypoxia/normoxia experiments (Kidney, 786-O)	Leisz S et al.	GEO: GSE65168
Bulk gene expression from hypoxia/normoxia experiments (Cervical, HeLa)	Tello D et al.	GEO: GSE33521
Bulk gene expression from hypoxia/normoxia experiments (Brain, LN229)	Koh MY et al.	GEO: GSE27523
Bulk gene expression from hypoxia/normoxia experiments (Liver, Hep3B)	Sena JA et al.	GEO: GSE57613
Bulk gene expression from hypoxia/normoxia experiments (Dendritic cells)	Fliesser M et al.	GEO: GSE60729
Bulk gene expression from hypoxia/normoxia experiments (Skin: 501mel, IGR39)	Louphrasitthiphol P et al.	GEO: GSE95280
Single cell RNA-seq analysis of gene expression in normoxic/hypoxic primary normal kidney cultures	Lombardi O et al.	GEO: GSE200207
Single cell gene expression from hypoxia/normoxia experiments (Breast, HCC1806, MCF-7)	This paper	Contact authors
Pan cancer RNA-seq analysis of gene expression in tumor and patient-matched normal tissue from the TCGA database	http://cancergenome.nih.gov/	N/A
**Software and algorithms**		
SigScores	This paper	SigScores
biomaRt	Durinck S et al.	biomaRt
Multi-symbol checker	Seal RL et al.	Multi-symbol checker
GeneCards	Stelzer G et al.	GeneCards
ShinyGO	Ge SX et al.	ShinyGO
GeoTcgaData	https://bioconductor.org/packages/release/bioc/html/GeoTcgaData.html	GeoTcgaData
FastQC	Andrews, S.	FastQC
Cutadapt	Martin, M	Cutadapt
STAR	Dobin, A. et al.	STAR
featureCounts	Liao, Y et al.	featureCounts
MultiQC	Ewels, P et al.	MultiQC
Snakemake	Köster, J et al.	Snakmake
Seurat	Hao, Y. et al.	Seurat
Uniform Manifold Approximation and Projection	McInnes et al.	Umap
clustree	Zappia, L et al.	Clustree
SigQC	Dhawan, A et al.	SigQC
waddR	Schefzik, R et al.	waddR
lifelines	N/A	Lifelines
R (4.3.0)	N/A	R
Python 3.11.5	N/A	Python
**Experimental models: Cell lines**		
MCF7	ECACC; Sigma-Aldrich	RRID: CVCL_0031 Cat# 86012803-1VL
HCC1806	Cytion	RRID: CVCL_1258 Cat# 300467-1VL
MBA-231	ECACC; Sigma-Aldrich	RRID: CVCL_0062 Cat# 92020424-1VL
**Critical commercial assays**		
INVIVO2 400 Physoxia workstation	Pro-Lab Diagnostics	N/A
SMARTer Ultra Low Input RNA Kit for Sequencing	Clontech	Cat# 634848
Nextera DNA Sample Preparation Kit	Illumina	Cat# FC-121-1030
Qubit High-Sensitivity DNA Kit	Invitrogen	Cat# Q32854
RNeasy Mini Kit	Qiagen	Cat# 74104
Agilent RNA Nano 6000 Chip	Agilent Technologies	Cat# 5067-1511

(*Continued on next page*)

Continued

REAGENT or RESOURCE	SOURCE	IDENTIFIER
Advantage 2 Polymerase Mix	Clontech	Cat# 639201
KAPA HiFi DNA Polymerase HotStart ReadyMix	Roche Sequencing	Cat# 07958935001
AMPure XP beads	Beckman Coulter	Cat# A63880
Chemicals, peptides, and recombinant proteins		
TrypLE Express	Thermo Fisher	Cat# 12604013
Phosphate buffered saline, pH 7.4	Thermo Fisher	Cat# 10010023
Triton X-100	Sigma-Aldrich	Cat# X100
Nuclease-Free Water	Qiagen	Cat# 129117
RNase inhibitor	Clontech	Cat# NC1471728
dNTP mix	Thermo Scientific	Cat# FERR0241
SuperScript II Reverse Transcriptase	Invitrogen	Cat# 18064022
USB Dithiothreitol (DTT)	Invitrogen	Cat# 707265ML
Betaine	Sigma-Aldrich	Cat# B0300
Magnesium chloride	Sigma-Aldrich	Cat# M8266
Buffer EB (Tris-Cl, pH 8.5)	Qiagen	Cat# 19086
IS PCR Primer	Clontech	Cat# 634946
SAHA	Sigma-Aldrich	Cat# SML0061
NaBu	Sigma-Aldrich	Cat# TR1008
Fetal bovine serum	Sigma-Aldrich	Cat# F7524-500ML

METHOD DETAILS

Identification of hypoxia gene expression signatures

A gene expression signature consists of a collection of one or more genes whose expression levels reflect a specific biological status or phenotype of the sample under examination. Gene expression signatures can be derived in a number of different ways, from the comparison of two samples (with or without relevant condition) with a simple two-samples statistical test, to machine learning techniques such as Generalized Linear Models (GLM),[143] Random Forest[144] or Support Vector Machines.[145] Published hypoxia gene expression signatures were identified from two main sources.

(1) A previously published extensive literature review from *Harris* et al.[41] containing 32 signatures
(2) A systematic review interrogating Web of Science, Scopus and Pubmed databases using the query: *("hypoxia signature"*) OR (*"hypoxia"* AND *"signature"*)

Articles resulting from the initial query were further filtered to exclude those not directly relevant to hypoxia signatures. Due to the methodology employed in this study, only genes identified as upregulated were incorporated into the final signatures. An exception was made for two specific signatures: Boidot 2014 Continuous Hypoxia and Boidot 2014 Cyclic Hypoxia, for which upregulation information was not specified.

Reannotation of hypoxia signatures

Gene expression signatures have been developed using a variety of sequencing methodologies, from microarrays to Next Generation Sequencing (NGS). Over the last decade, microarrays were still widely used to derive gene expression signatures. However, the most recent signatures tend to rely on RNA sequencing data, thanks to the decreasing costs of this technology. Often, the authors of the signatures mainly report the genes in their publications as Gene Name (or HGNC symbol) and rarely include more specific and stable identifiers, such as microarray Probe ID or the Ensembl/Entrez Gene ID. This provides a challenge as biological understanding and gene nomenclature and definition evolve over time. A commonly used way to standardise gene annotations across signatures involves converting gene symbols from the articles into stable identifiers using the biomaRt software.[146] biomaRt is a federated database system (FDBS) providing unified access to disparate data sources. The European Bioinformatics Institute provides access to Ensembl genomes through a biomaRt implementation, accessible via browser and several software packages.

However, this ID conversion might still be challenging for two reasons:

(1) Gene names change over time: for example, the Vascular Endothelial Growth Factor A (VEGFA) gene, which plays a key role in hypoxia, was previously reported as VEGF. Thus any signatures that contain VEGF, will lose this gene during the conversion process using the most recent Ensembl BiomaRt versions.

(2) Ensembl annotation database change over time: a new release of the Ensembl dataset could result in loss of information since Ensembl does not include outdated annotations in its relational database. For example, the above-mentioned VEGF gene name is not available on the recent Ensembl release.

Therefore, in order to convert gene symbols from papers into Ensembl gene IDs, it is critical to reannotate them into their most updated version first. For this task, the multi-symbol checker dataset from HGNC was used.[147] Each gene expression signature was reannotated using the multi-symbol checker to convert each gene symbol to the latest "approved symbol". If more alias symbols were available, the relevance score from GeneCards[148] was used to select the most relevant annotation. Gene names annotated as protein coding were prioritised.

Once updated to the most recent and relevant gene symbol, genes in the signature were converted into both Ensembl and Entrez Gene IDs using biomaRt[146] (Ensembl 107: Jul 2022), an R interface to the BioMart software suite. Finally, a conversion dictionary was created to match each Ensembl Gene ID with any other external annotation such as Probe IDs or Ensembl transcript IDs, useful to select the correct gene from the gene expression datasets included in this study. A list of all gene symbols and Ensembl Gene IDs is available in *Appendix 1*.

Pathway analysis

Over-representation pathway analysis was performed using ShinyGO[149] v0.77 to identify gene pathways that were predominantly enriched in the signatures. A pathway was deemed significantly enriched if a False Discovery Rate (FDR) of less than 0.05 was observed.

Cell line data

Publicly available gene expression datasets consisting of at least one hypoxic and normoxic control were retrieved from The Gene Expression Omnibus (GEO) archive.[150] GEO data is organised in Series, Samples, and Platforms.

- *Series*: a Series record provides a general description of the whole study, and links together a group of related Samples. Each Series is identified by a unique accession number having "GSE" as prefix (i.e., GSExxx).
- *Platforms*: a Platform record is composed of a summary description of the array or sequencer. A Platform ID is reported with the letters "GPL" and the platform number. For example, GPL570 is the corresponding ID for the Affymetrix Human Genome U133 Plus 2.0 Array. One platform can be assigned to multiple Series. Each microarray Platform record includes a manifest file, a dataset reporting the microarray probe IDs annotated with the corresponding gene information such as gene symbol(s) or transcript(s). Bulk RNAseq platforms do not contain a manifest file and gene expression IDs are reported in the gene expression dataset for each Series.
- *Samples*: a Sample record describes all the conditions under which an individual sample was handled. Each Sample is identified with a unique ID, the letters "GSM" followed by the sample number (e.g., GSM71498). A Sample can be part of multiple Series, but must reference only one Platform.

Batch effects can occur due to differences in the sequencing technology used, but also different technical and experimental conditions, such as the lab users and the consumables utilised. Combining all samples from different Series together in a unique dataset could mask underlying batch effects and correcting them could be challenging, leading to inaccurate conclusions. To obviate this problem, the performance of hypoxia signatures was assessed by evaluating the difference between paired hypoxic/normoxic samples (defined as pairwise combinations) according to the following rules.

(1) Both samples must be part of the same Series and sequenced using the same technology
(2) Both samples are from the same cell line (for example, MDA-MB-231 samples cannot be combined with MCF-7 samples)

Unfortunately, the GEO archive collects most of the sample-related information as *unstructured* data (e.g., free text). Thus, all the information such as oxygen concentration, time under hypoxia, cell type or additional treatments had to be extracted through manual curation.

In the current study, the GEO database was queried to identify cell line experiments testing hypoxic conditions at different time points and oxygen concentrations. Other exogenous conditions in addition to hypoxia, such as exposure to a low glucose environment or experiments involving hypoxia mimicking agents were also identified. The following criteria were included in the query.

- Series must contain the word "*Hypoxia"* and in any of the fields in the Series description
- Experiments must have been performed using human-derived biological samples
- Gene expression profiles were obtained using high-throughput sequencing or arrays

The query identified 204 Series and a total number of 2,134 Samples. Three sequential filtering rules were then applied.

(1) Rule #1: The first filter was performed on each Series by looking at its experimental description. Series containing at least one *in vitro* hypoxic sample and one normoxic control were selected leaving 103 Series and 1511 samples after the filtering

(2) Rule #2: The second filter consisted of removing all Series that had not been published or referenced in any peer-reviewed article, leaving 97 Series and 1423 samples after this step.
(3) Rule #3: The third filter was performed by manual curation of all the Series and their corresponding papers, retrieving experimental information such as cell lines, oxygen tensions used, etc. Series were excluded if any samples in the Series reported different annotation between the Series information in GEO and the information in the scientific article.

A total number of 73 Series and 767 samples were finally selected. Series used are found in Table S5. Both RNA-seq and microarray gene expression data were used in their post-processed form, as used in their published reference papers. A further normalisation step was included for RNA-seq data to allow comparison of gene expression data across different samples, converting each gene expression value into Transcript Per Million (TPM) using GeoTcgaData.

For each microarray dataset, the corresponding manifest included in the GEO Series Platform was used to intersect the gene IDs from the signatures with their respective probeset IDs available in the platform manifest using a conversion dictionary. For bulk RNAseq data, the gene or transcript ID in the gene expression dataset was used to match the corresponding gene ID from the hypoxia signatures.

After selecting only up-regulated genes from hypoxia gene expression signatures, a final quality check was performed on all the GEO Series. For each dataset, the overlap between genes in the signature and available genes in each series was evaluated. All the platforms and RNAseq data where >20% of the signatures had >20% of genes missing from the dataset were excluded. This was done in order to keep the number of genes as consistent as possible across all the platforms and resulted in the exclusion of three platforms for microarray data and four datasets for bulk RNAseq (Figures S23 and S24).

Single-cell data

MCF7 and HCC1806 cells were maintained as previously described.[151] Hypoxia was defined as 0.1% oxygen concentration. The choice of hypoxia duration was guided by CA9 expression levels which were measured by FACS and western blotting after 24 h and 72 h of hypoxia. Maximal induction was reached at 72 h for MCF7 and 24 h for HCC1806. Thus, the MCF7 and HCC1806 cells were cultured in hypoxia for these respective durations.

SmartSeq single-cell sequencing was carried out as in previous studies.[152] Pre-processing was carried out using the following: FastQC for quality control,[153] Cutadapt for read trimming,[154] STAR for read alignment,[155] and featureCounts feature counting.[156] MultiQC was used for aggregation of all quality control metrics associated with every single step in the pipeline.[157] Snakemake was the pipeline language used to chain and execute the entire pre-processing pipeline.[158]

The primary analysis pipeline was executed in the R programming language and the analysis was done in the Seurat package for scRNA-Seq analysis.[159] Dimensionality reduction was applied to facilitate the visualisation of the data and the detection of underlying clusters. The first dimensionality reduction step was principal component analysis (PCA), and the second dimensionality reduction step was Uniform Manifold Approximation and Projection for Dimension Reduction (UMAP).[160]

Clustering analysis was done using the louvain algorithm of community detection. The choice of the number of principal components and clustering resolution parameters was based on elbow plots and clustering trees respectively (generated from the clustree package[161]).

Batch effects are a common issue in scRNA-Seq datasets, and integration methods are commonly used to correct for them.[162] In the present study, integration methods were avoided due to their tendency to eliminate all biological variability in the data, a well-known limitation of such approaches. Signature quality control metrics, e.g., intra signature correlation, feature expression heatmaps, radar plots, etc., were generated using SigQC.[44]

Clinical data

The TCGA gene expression data was retrieved from the Broad GDAC Firehose. TCGA is a “landmark cancer genomics program”, coordinated by the National Cancer Institute and National Human Genome Research (USA).[163] The project includes profiling of 20,000 primary cancer samples from 33 cancer types with, in some cases, matched normal samples. The TCGA dataset has been made publicly available in an anonymised fashion for the scientific community. In this work, mRNA expression in TCGA samples across 10 key solid tumors is examined, with a focus on those where at least 30 normal adjacent tissue (NAT) samples were available. The performance of hypoxia signatures was evaluated against these samples. Standard TCGA annotation is used throughout this manuscript, but for clarity here are the acronyms used for tumor types investigated: Breast invasive carcinoma: BRCA, Colon adenocarcinoma: COAD, Head and Neck squamous cell carcinoma: HNSC, Liver hepatocellular carcinoma: LIHC, Lung adenocarcinoma: LUAD, Lung squamous cell carcinoma: LUSC, Prostate adenocarcinoma: PRAD, Stomach adenocarcinoma: STAD, Thyroid carcinoma: THCA, Uterine Corpus Endometrial Carcinoma: UCEC.

Calculation of hypoxia signature summary scores

Gene expression signatures can be reduced to a single measure, which is referred to as a score. This score reflects the status or strength of the phenotype they represent and can be determined through various methods.[44] With regards to hypoxia signatures, hypoxia scores are single values indicating the level of hypoxia in biological samples. Fourteen scores have been evaluated in this study, including common measures of central tendency such as mean or median, and composite measures for differential

gene expression analysis, such as Gene Set Variation Analysis (GSVA) or single-sample Gene Set Enrichment Analysis (ssGSEA). Further information about these scores can be found in Table S4. Scores included in the study are.

- Mean
- Median
- Mode
- Midrange
- Midhinge
- Trimean
- Interquartile Range
- Interquartile Mean
- Mean Absolute Deviation
- Average Absolute Deviation
- *Z* score
- GSVA
- ssGSEA
- Normalised Cumulative Score

Since the proposed methodology consists of measuring and comparing differences across hypoxic and normoxic samples based on their hypoxia score, only up-regulated genes from gene expression signatures were selected.

The calculation of hypoxia scores was performed using SigScores, an extended version of SigQC,[44] a previously developed tool by our lab for evaluating the quality of gene expression signatures and calculating gene signature scores. SigScores is an R package that is designed to offer a simple function to compute all available scores. The intention behind its creation is to offer different summary measures in a single software, facilitating effortless computation and comparison of the metrics. Specifically for this study, SigScores was developed and is now accessible via GitHub (https://alebarberis.github.io/sigscores/index.html) and Zenodo (https://doi.org/10.5281/zenodo.14608695). Using the SigScores package, hypoxia scores in both bulk and single-cell data were calculated.

Comparison with random gene signatures (RGS) in bulk gene expression data

The performance of hypoxia signatures was assessed by comparing them to Random Gene Signatures (RGS) of the same length. This evaluation aimed to assess whether the hypoxia signatures significantly outperformed random sets of genes. Similar approaches have been used elsewhere in other contexts.[164,165] It is particularly important to take such an approach, as previous work has highlighted that unrelated random gene signatures may have been wrongly associated with clinical outcomes.[166] To avoid this, in this study, we used a permutation-based analysis approach. Each Series was tested separately to avoid experimental biases and batch effects, i.e., samples from GSE15530 were not compared to samples from GSE3188, even if belonging to the same cell line.

The first step in our study was to define how the hypoxia scores can be evaluated in controlled cell line experiments. These scores have been assessed by looking at their value according to the oxygen status of the samples (hypoxic or normoxic). For example, if the scores truly reflect hypoxia, a hypoxic sample would be expected to have a higher hypoxia score than a normoxic sample since downregulated genes have been excluded from each gene expression signature.

Each GEO Series contains multiple hypoxic and normoxic samples, often including replicates. However, most of the samples do not include information on how the replicates were processed (e.g., in parallel, paired or all together). Therefore, to obtain comprehensive insights, we compared all possible pairwise combinations of hypoxic and normoxic samples within the same GEO Series. This strategy enabled the evaluation of every hypoxic/normoxic sample pairing without *a priori* selection. Thus, each hypoxic sample was matched against every normoxic sample. For instance, a Series comprising two hypoxic and two normoxic samples resulted 4 pairwise combinations (calculated as $\#\, hypoxic\ samples \times \#\, normoxic\ samples$).

One way of measuring how a signature differentiates between pairwise hypoxia/normoxia combinations consists of calculating the absolute value between their two hypoxia scores. Measuring this distance, and evaluating it against RGS, is the core element of the method presented in this study. Given a hypoxia signature and a score method, the Euclidean score distance $d_{SIG}(hyp, norm)$ is calculated as follows, with h_{score} and n_{score} representing the score of the hypoxic and normoxic samples respectively:

$$d_{SIG}(hyp, norm) = \sqrt{(h_{score} - n_{score})^2}$$

If a hypoxia signature and score combination is predictive of an active hypoxia response, one would expect a large distance $d_{SIG}(hyp, norm)$. The same approach also is carried out using an RGS and this too gives a score distance, defined as $d_{RGS}(hyp, norm)$, which is expected to be distributed around zero. Thus, if the hypoxia signature is separating the samples correctly, the $d_{SIG}(hyp, norm)$ will be greater than $d_{RGS}(hyp, norm)$ (Figure S25). To further enhance statistical robustness, 1000 different RGS have been used to calculate multiple $d_{RGS}(hyp, norm)$. These distances from multiple RGS obtained with a permutation test can then be used to derive a null distribution and can be compared to the hypoxia signature $d_{SIG}(hyp, norm)$ on each individual pair of normoxic and hypoxic samples. This is demonstrated in Figure 2B on one pair of samples: d_{SIG} was compared to the d_{RGS} from

1000 different RGS with the red bar indicating where the the d_{SIG} falls in the distribution. When d_{SIG} registers the highest value within the distribution, it indicates superior performance of the corresponding hypoxia signature and score compared to all evaluated RGS. This can be measured by calculating a significance value (p) as below (with $\varepsilon = 0.0009$ representing the correction factor[167]) and this was repeated for all pairwise combinations of hypoxic/normoxic samples in each GEO *Series*:

$$p = 1 - (\textit{percentile of score} \div 100) + \varepsilon$$

The percentile of score is the number of distances (expressed as percentage) in the distribution that are lower than d_{SIG}. For example, a percentile of score of 100% means that all the 1000 d_{RGS} are lower than the d_{SIG}. However, if a score in the normoxic sample is greater than that in the hypoxic sample using the hypoxia gene expression signature, the significance between the two samples is automatically made non-significant ($p = 1$). The correction factor ε is used in the permutation test to prevent a zero p-value, occurring when no RGS outperforms the original signature, reflecting the uncertainty inherent in any statistical test. This correction factor is calculated as the lowest possible p obtainable from the permutation test when only one random signature performs better than the original signature ($1/1001 = 0.0009$).

The last step of the analysis consists of calculating an accuracy index for each signature as the ratio between the number of significant pairwise combinations (threshold $p \leq 0.005$) over the total number of combinations for each cancer type or experimental condition. The p-value corresponds to the probability of rejecting the null hypothesis that RGS perform equally to the hypoxia signature. Therefore, a stringent threshold of $p = 0.005$ was chosen in order to allow a maximum of five RGS to outperform the original signature and allow the original signature to still remain significant ($5/1001 = 0.0049$). The accuracy index is reported as the percentage of correct classifications. For example, if 80 hypoxic/normoxic pairwise combinations out of 100 tested on all the breast cancer cell lines are significant compared to their respective RGS (as described above), the accuracy is stated as 80% in breast cancer. This approach was repeated for each of the fourteen scoring metrics and seventy signatures.

Finally, other common gene expression scores such as the first Principal Component (PC1) from Principal Component Analysis (PCA) or Pathway Level Gene Expression Analysis (PLAGE) were not considered in the analysis. These scores cannot be assessed using the presented method, as there would be no way to determine if the direction of the implied variance using the hypoxia signature is the same as in any RGS.

Comparison with random gene signatures (RGS) in single-cell gene expression data

To assess the efficacy of hypoxia gene expression signatures in single-cell data, an approach analogous to that applied to bulk RNA-seq data was adapted. Single-cell data necessitate a modification in the statistical procedure due to the provision of a score for each individual cell as opposed to one for an entire sample. In particular, the following aspects need to be considered.

- Pairwise combinations and P-value calculation: using a pairwise combination approach in single-cell data would be inappropriate, given that each hypoxic cell would be compared with all normoxic cells. This is not only computationally intensive but also potentially misleading, as each cell is likely to manifest a unique gene expression profile. For example, a pair of hypoxic and normoxic samples, each comprising 100 cells, would result in 10,000 pairwise combinations, necessitating approximately 10 million permutations (1000 RGS for each combination) to derive p-values for a single signature and score. Therefore, quantifying the hypoxic response of a single pairwise combination of cells in comparison to all other pairs would be challenging and difficult to interpret.
- Calculation of signatures accuracy: In the context of single-cell data, the determination of accuracy must be performed at the individual cell level, making traditional sample-wide metrics less suitable.

Given these complexities, an alternative approach was employed compared to bulk data analysis.

For each signature and gene expression score, hypoxia scores were calculated for each cell within normoxic and hypoxic samples. These scores were utilised to form two distinct distributions: one for hypoxic cells and another for normoxic cells. The aim is to evaluate whether the score distribution originating from hypoxic cells is statistically different from that derived from normoxic cells. To statistically compare these distributions, the Wasserstein metric was employed as a robust measure of distance between the distributions. Following calculation of the Wasserstein metric for the original hypoxia signature, the same permutation-based analysis was applied to generate a null distribution from 1000 random gene signatures, facilitating statistical comparisons between the original hypoxia signature and RGS. Significance was determined at a p-value <0.005, consistent with the approach for bulk data. The Wasserstein distance metric was computed using the R function wasserstein.test() from the package waddR.[168]

Notably, in contrast to bulk data, where the accuracy of each signature could be calculated, only the p-value was used here to identify signatures with statistically significant differences. This statistical framework offers a solid methodology for assessing hypoxia gene signature performance in single-cell data, taking into account its unique challenges while preserving the essential attributes of single-cell analysis.

Statistical evaluation of tumor VS NAT

It is generally expected that tumor tissue is, on average, more hypoxic than normal tissue due to the imbalance between metabolic demand and the insufficient supply from aberrant blood vessels. Normoxic tumors, in fact, should have hypoxia scores comparable

to NAT. In this analysis, we examined differences across solid tumors in TCGA for each signature. The distributions of hypoxia signature scores in cancer tissues were compared to those in NAT using the nonparametric Mann-Whitney U test. An alternative hypothesis was defined based on the assumption that the average hypoxia score in cancer samples exceeds that in NAT (but not vice versa). Specifically, let T(u) and N(u) represent the cumulative distribution functions for the distributions underlying x and y, respectively. The hypothesis asserts that the distribution underlying x (tumor hypoxia scores) is stochastically greater than that underlying y (NAT hypoxia scores), i.e., T(u) > N(u) for all u.

For instance, if the hypoxia signature scores in the cancer group are on average lower than those in NAT, the assumption is violated, and the difference is considered non-significant. A significance threshold of $p < 7.14\text{E}{-}05$ was applied to identify signatures that met the criteria, corrected using the Bonferroni method, accounting for the number of signatures (70) and cancer types (10) evaluated.

Comparison with RGS in clinical samples

A similar approach used for comparing hypoxia signatures in *in-vitro* samples can be applied to clinical samples. However, instead of evaluating pairs of samples, this analysis focuses on determining whether significant differences exist between the distributions of hypoxia scores in tumors compared to NAT. The analysis involves two main steps, performed for each signature, score, and cancer type.

- The non-parametric Mann-Whitney U test is performed to derive a p-value, assessing whether a statistically significant difference exists in hypoxia signature scores between tumor and NAT samples. This statistical evaluation is repeated on 1000 RGS, and for each, a p-value is calculated as described previously.
- A Signature Performance Index (SPI), expressed as a percentage, is then derived. This is done by calculating how often the p-value from the original hypoxia signature is lower than any of the 1000 RGS p-values. For example, if 990 out of 1000 RGS have a higher p-value than the original hypoxia signature, the SPI will be 99%.

It is noteworthy to mention that the sample size of both tumor and adjacent normal tissues will be the same in each of the 1000 RGS. This approach enables the comparison of 1000 *p*-values from RGS with the *p*-value from the original signature to derive the SPI and determine if the hypoxia signature is statistically significant compared to the RGS.

Survival analysis

Pan-cancer prognostic performance was investigated by using the Kaplan-Meier (KM) estimator at five years in TCGA data followed by a multivariate analysis using the Cox Proportional Hazard (CPH) model. The KM estimator is a non-parametric statistic used to derive the survival function from lifetime data. A total of 5221 out of 5407 samples were included in the prognostic analysis using Disease-Specific Survival (DSS), with 186 samples being excluded due to missing DSS information (Table S10). A point of contention in the field is what type of thresholds should be used across tumor types to denote low and high hypoxic samples. Previous works have used above and below the median, exemplar ref. [60], however this is not necessarily the most comprehensive approach. Thus, to be more thorough we investigated the prognostic ability of the signature/score combinations comparing every fifth percentile using DSS, as different tumor types have different degrees of hypoxia. The log-rank test was used to compare the survival distribution between samples and the KM estimator was calculated using the Python module lifelines v.0.26.4. A signature/score at a percentile was defined as significantly prognostic if the log-rank $p \leq 0.005$, threshold Bonferroni-corrected by the 10 incremental thresholds tested starting from the 50th percentile, with an incremental step of 5 (e.g., 50th, 55th, 60th, until 95th).

Confirmatory CPH analyses were conducted using R (version 4.3.0), with the proportional hazards assumption tested via Schoenfeld residuals. Observation time was measured until the date of death or the end of the five-year monitoring period. Hypoxia signature/score combinations of interest were analyzed by dividing them into five quantiles in multivariable-adjusted models. These models were adjusted for: age (in 10-year age groups), stage, grade, gender (sex), ethnicity, and smoking history for all cancers. HPV status and alcohol consumption was included for HNSC, eGFR/ALK mutation status for LUAD, and Hepatitis B, Hepatitis C, non-alcoholic fatty liver disease, haemochromatosis, alpha-1-antitrypsin deficiency, alcohol consumption and vascular invasion for LIHC.

Cell Genomics, Volume *5*

Supplemental information

Defining hypoxia in cancer: A landmark evaluation of hypoxia gene expression signatures

Matteo Di Giovannantonio, Fiona Hartley, Badran Elshenawy, Alessandro Barberis, Dan Hudson, Hana S. Shafique, Vincent E.S. Allott, David A. Harris, Simon R. Lord, Syed Haider, Adrian L. Harris, Francesca M. Buffa, and Benjamin H.L. Harris

Supplemental information

Defining hypoxia in cancer: a landmark evaluation of hypoxia gene expression signatures

Matteo Di Giovannantonio, Fiona Hartley, Badran Elshenawy, Alessandro Barberis, Dan Hudson, Hana Shafique, Vincent E.S Allott, David A. Harris, Simon R. Lord, Syed Haider, Adrian L. Harris, Francesca M. Buffa, Benjamin H.L Harris

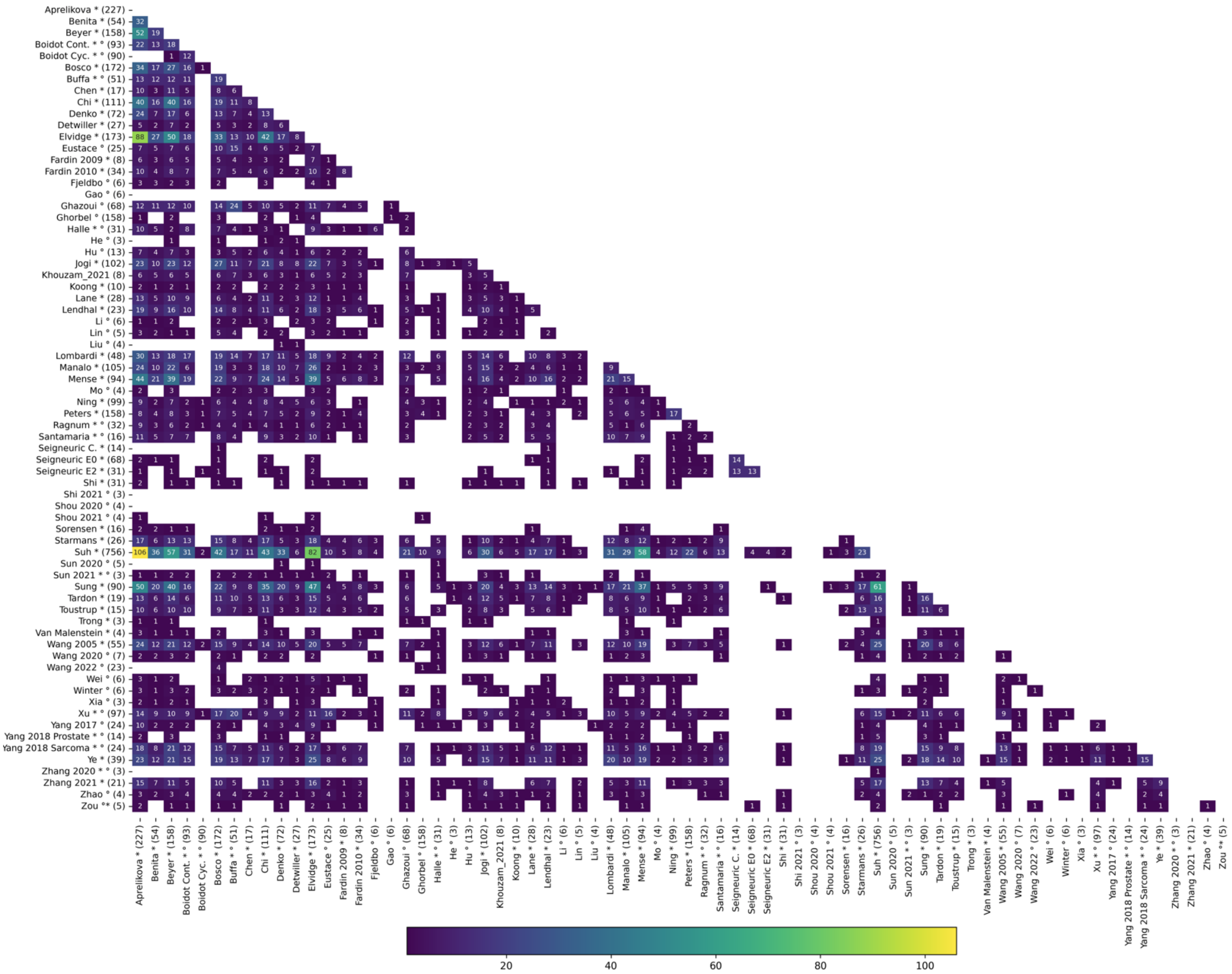
Aprelikova * (227)
Benita * (54)
Beyer * (158)
Boidot Cont. * ° (93)
Boidot Cyc. * ° (90)
Bosco * (172)
Buffa * ° (51)
Chen * (17)
Chi * (111)
Denko * (72)
Detwiller * (27)
Elvidge * (173)
Eustace ° (25)
Fardin 2009 * (8)
Fardin 2010 * (34)
Fjeldbo ° (6)
Gao ° (6)
Ghazoui ° (68)
Ghorbel ° (158)
Halle * ° (31)
He ° (3)
Hu ° (13)
Jogi * (102)
Khouzam_2021 (8)
Koong * (10)
Lane * (28)
Lendhal * (23)
Li ° (6)
Lin ° (5)
Liu ° (4)
Lombardi * (48)
Manalo * (105)
Mense * (94)
Mo ° (4)
Ning * (99)
Peters * (158)
Ragnum * ° (32)
Santamaria * ° (16)
Seigneuric C. * (14)
Seigneuric E0 * (68)
Seigneuric E2 * (31)
Shi * (31)
Shi 2021 ° (3)
Shou 2020 ° (4)
Shou 2021 ° (4)
Sorensen * (16)
Starmans * (26)
Suh * (756)
Sun 2020 ° (5)
Sun 2021 * ° (3)
Sung * (90)
Tardon * (19)
Toustrup * (15)
Trong * (3)
Van Malenstein * (4)
Wang 2005 * (55)
Wang 2020 ° (7)
Wang 2022 ° (23)
Wei ° (6)
Winter ° (6)
Xia ° (3)
Xu * ° (97)
Yang 2017 ° (24)
Yang 2018 Prostate * ° (14)
Yang 2018 Sarcoma * ° (24)
Ye * (39)
Zhang 2020 * ° (3)
Zhang 2021 * (21)
Zhao ° (4)
Zou °* (5)
20
40
60
80
100

Supplementary Figure S1: The number of overlapping genes between signatures

Heatmap of gene overlap across investigated signatures. The colour intensity indicates the number of shared genes between each pair of signatures. Signatures with no overlapping genes are represented in white. The numbers in parentheses following each signature name report the final gene count for that signature after quality check, related to Figure 1.

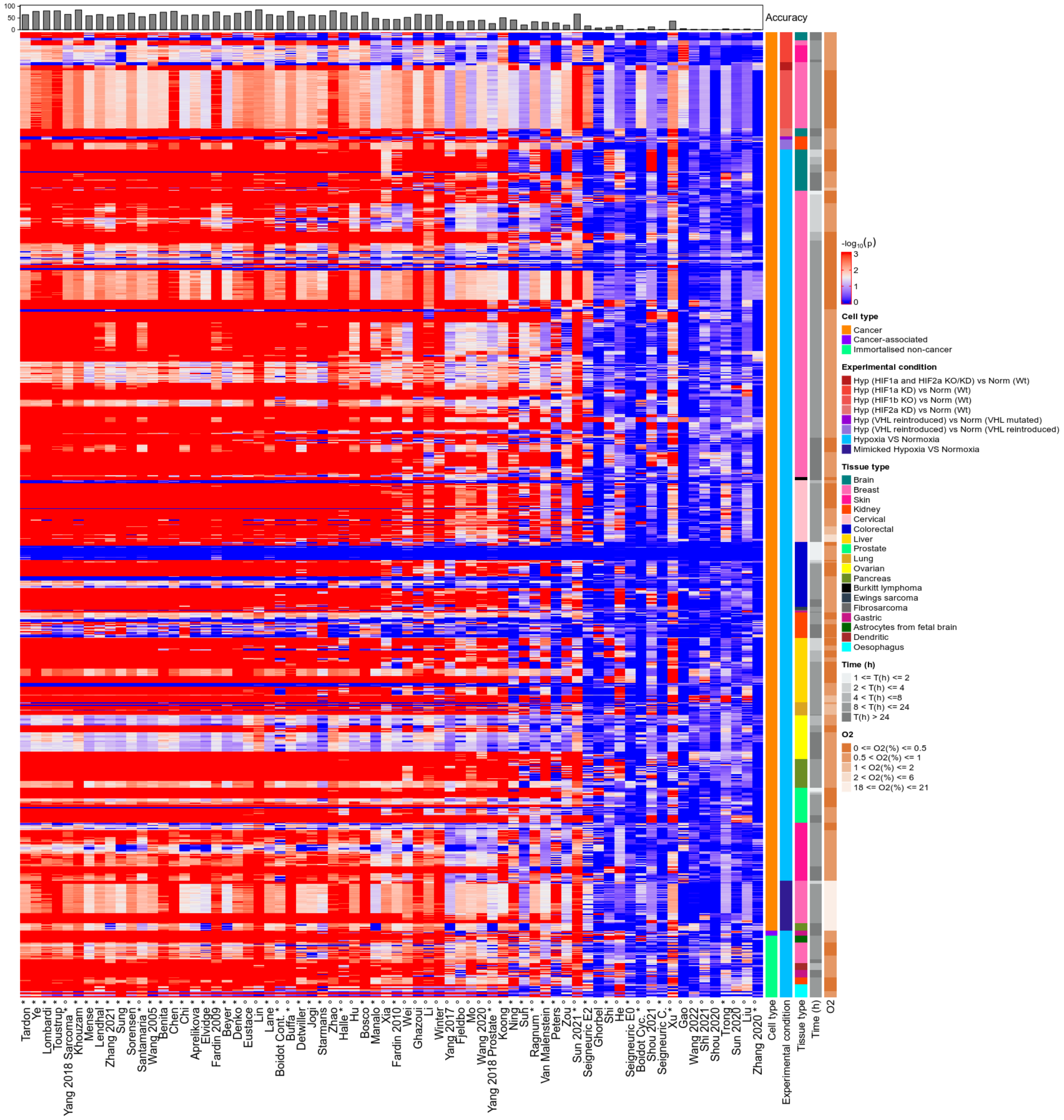

100
50
0
Accuracy
Tardon
Ye
Lombardi
Toustrup
Yang 2018 Sarcoma
Khouzam
Mense
Lendhal
Zhang 2021
Sung
Sorensen
Santamaria
Wang 2005
Benita
Chen
Chi
Aprelikova
Elvidge
Fardin 2009
Beyer
Denko
Eustace
Lin
Lane
Boidot Cont.
Buffa
Detwiler
Jogi
Starmans
Zhao
Halle
Hu
Bosco
Manalo
Xia
Fardin 2010
Wei
Ghazoui
Li
Winter
Yang 2017
Fjeldbo
Mo
Wang 2020
Yang 2018 Prostate
Koong
Ning
Suh
Ragnum
Van Malenstein
Peters
Zou
Sun 2021
Seigneuric E2
Ghorbel
Shi
He
Seigneuric E0
Boidot Cyc.
Shou 2021
Seigneuric C.
Xu
Gao
Wang 2022
Shi 2021
Shou 2020
Trong
Sun 2020
Liu
Zhang 2020
Cell type
Experimental condition
Tissue type
Time (h)
O2
-log10(p)
3
2
1
0
Cell type
Cancer
Cancer-associated
Immortalised non-cancer
Experimental condition
Hyp (HIF1a and HIF2a KO/KD) vs Norm (Wt)
Hyp (HIF1a KD) vs Norm (Wt)
Hyp (HIF1b KO) vs Norm (Wt)
Hyp (HIF2a KD) vs Norm (Wt)
Hyp (VHL reintroduced) vs Norm (VHL mutated)
Hyp (VHL reintroduced) vs Norm (VHL reintroduced)
Hypoxia VS Normoxia
Mimicked Hypoxia VS Normoxia
Tissue type
Brain
Breast
Skin
Kidney
Cervical
Colorectal
Liver
Prostate
Lung
Ovarian
Pancreas
Burkitt lymphoma
Ewings sarcoma
Fibrosarcoma
Gastric
Astrocytes from fetal brain
Dendritic
Oesophagus
Time (h)
1 <= T(h) <= 2
2 < T(h) <= 4
4 < T(h) <=8
8 < T(h) <= 24
T(h) > 24
O2
0 <= O2(%) <= 0.5
0.5 < O2(%) <= 1
1 < O2(%) <= 2
2 < O2(%) <= 6
18 <= O2(%) <= 21

Supplementary Figure S2: Complex heatmap (mean)

Complex heatmaps of signatures p-values in hypoxic vs. non-hypoxic samples across GEO cell-line conditions in bulk RNAseq and microarray data. Shades of red and blue denote low and high p-values, respectively, as derived from permutation tests and represent the likelihood that a random gene signature would outperform a hypoxia signature. The top bar plot summarises signature accuracy, calculated as the number of pairwise combinations with p-values < 0.05 over the total number of pairwise combinations. The right-hand legend provides additional sample features, with titles located at the beginning of the x-axis, related to Figure 3.

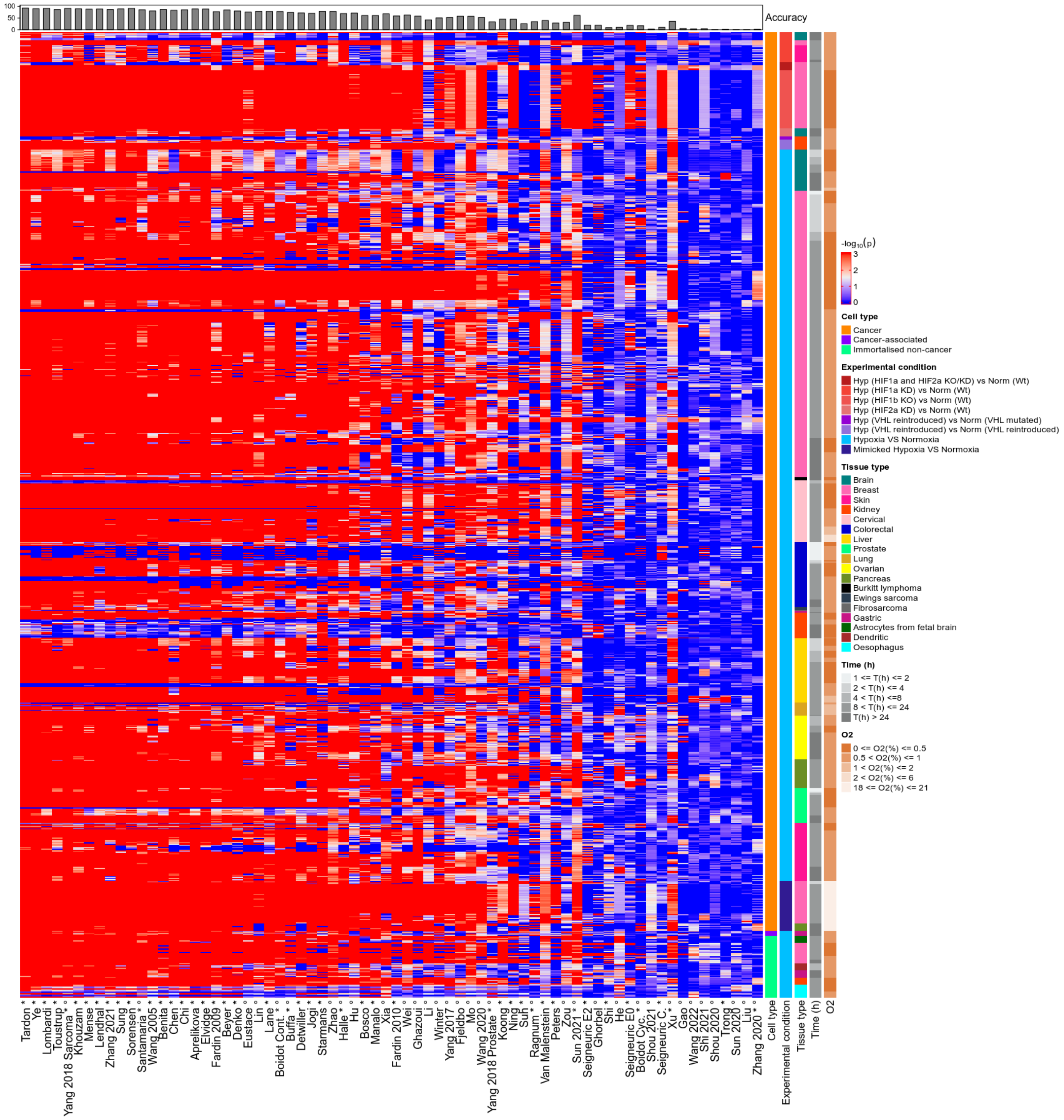

Accuracy
100
50
0
Tardon
Ye
Lombardi
Toustrup
Yang 2018 Sarcoma
Khouzam
Mense
Lendhal
Zhang 2021
Sung
Sorensen
Santamaria
Wang 2005
Benita
Chen
Chi
Aprelikova
Elvidge
Fardin 2009
Beyer
Denko
Eustace
Lin
Lane
Boidot Cont.
Buffa
Detwiller
Jogi
Starmans
Zhao
Halle
Hu
Bosco
Manalo
Xia
Fardin 2010
Wei
Ghazoui
Li
Winter
Yang 2017
Fjeldbo
Mo
Wang 2020
Yang 2018 Prostate
Koong
Ning
Suh
Ragnum
Van Malenstein
Peters
Zou
Sun 2021
Seigneuric E2
Ghorbel
Shi
He
Seigneuric E0
Boidot Cyc.
Shou 2021
Seigneuric C.
Xu
Gao
Wang 2022
Shi 2021
Shou 2020
Trong
Sun 2020
Liu
Zhang 2020
Cell type
Experimental condition
Tissue type
Time (h)
O2
-log10(p)
3
2
1
0
Cell type
Cancer
Cancer-associated
Immortalised non-cancer
Experimental condition
Hyp (HIF1a and HIF2a KO/KD) vs Norm (Wt)
Hyp (HIF1a KD) vs Norm (Wt)
Hyp (HIF1b KO) vs Norm (Wt)
Hyp (HIF2a KD) vs Norm (Wt)
Hyp (VHL reintroduced) vs Norm (VHL mutated)
Hyp (VHL reintroduced) vs Norm (VHL reintroduced)
Hypoxia VS Normoxia
Mimicked Hypoxia VS Normoxia
Tissue type
Brain
Breast
Skin
Kidney
Cervical
Colorectal
Liver
Prostate
Lung
Ovarian
Pancreas
Burkitt lymphoma
Ewings sarcoma
Fibrosarcoma
Gastric
Astrocytes from fetal brain
Dendritic
Oesophagus
Time (h)
1 <= T(h) <= 2
2 < T(h) <= 4
4 < T(h) <=8
8 < T(h) <= 24
T(h) > 24
O2
0 <= O2(%) <= 0.5
0.5 < O2(%) <= 1
1 < O2(%) <= 2
2 < O2(%) <= 6
18 <= O2(%) <= 21

Supplementary Figure S3: Complex heatmap (median)

Complex heatmap of signature p-values in hypoxic vs. non-hypoxic samples across GEO cell-line conditions in bulk RNAseq and microarray data, related to Figure 3. Conventions as in S2.

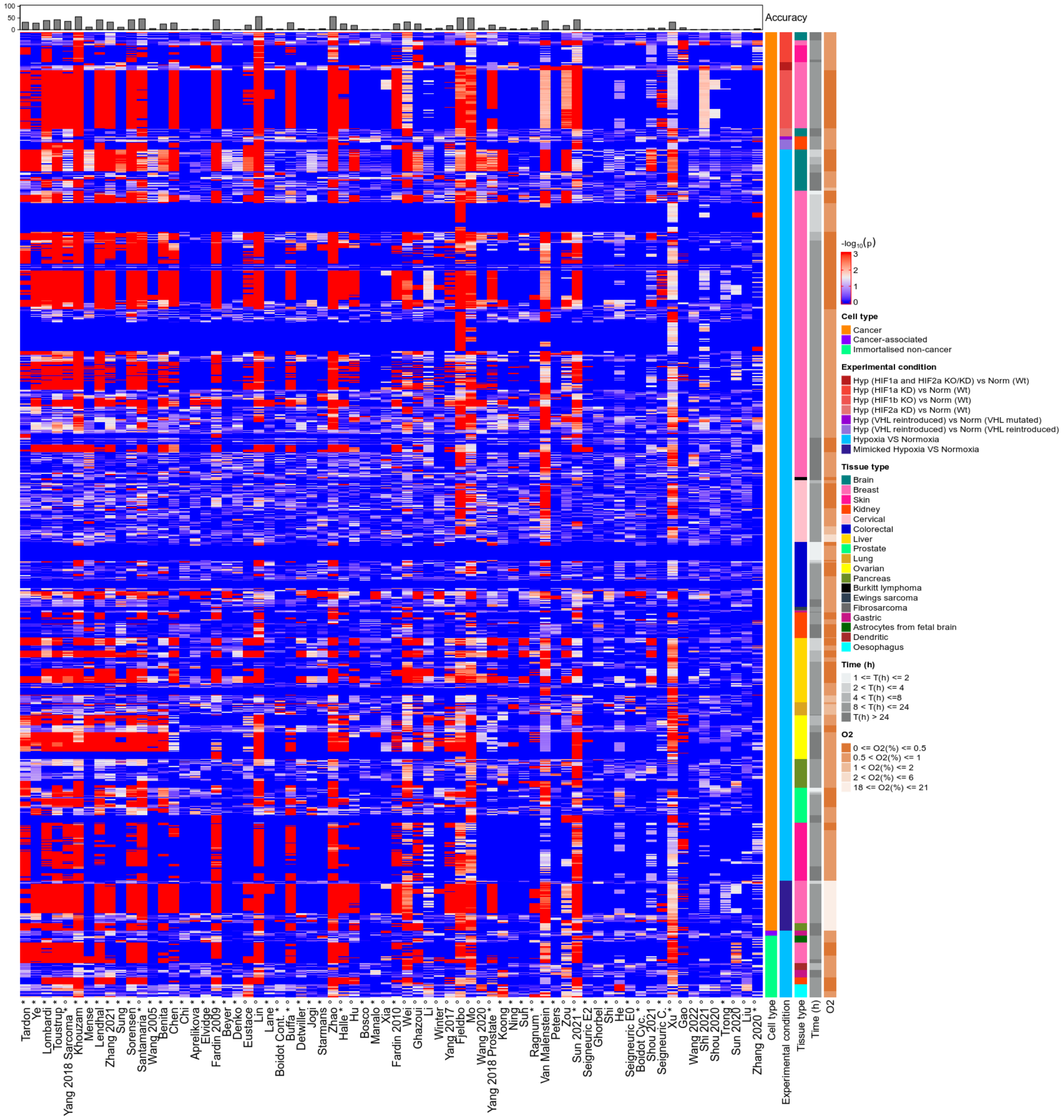

100
50
0
Accuracy
-log10(p)
3
2
1
0
Cell type
Cancer
Cancer-associated
Immortalised non-cancer
Experimental condition
Hyp (HIF1a and HIF2a KO/KD) vs Norm (Wt)
Hyp (HIF1a KD) vs Norm (Wt)
Hyp (HIF1b KO) vs Norm (Wt)
Hyp (HIF2a KD) vs Norm (Wt)
Hyp (VHL reintroduced) vs Norm (VHL mutated)
Hyp (VHL reintroduced) vs Norm (VHL reintroduced)
Hypoxia VS Normoxia
Mimicked Hypoxia VS Normoxia
Tissue type
Brain
Breast
Skin
Kidney
Cervical
Colorectal
Liver
Prostate
Lung
Ovarian
Pancreas
Burkitt lymphoma
Ewings sarcoma
Fibrosarcoma
Gastric
Astrocytes from fetal brain
Dendritic
Oesophagus
Time (h)
1 <= T(h) <= 2
2 < T(h) <= 4
4 < T(h) <=8
8 < T(h) <= 24
T(h) > 24
O2
0 <= O2(%) <= 0.5
0.5 < O2(%) <= 1
1 < O2(%) <= 2
2 < O2(%) <= 6
18 <= O2(%) <= 21
Tardon
Ye
Lombardi
Toustrup
Yang 2018 Sarcoma
Khouzam
Mense
Lendhal
Zhang 2021
Sung
Sorensen
Santamaria
Wang 2005
Benita
Chen
Chi
Aprelikova
Elvidge
Fardin 2009
Beyer
Denko
Eustace
Lin
Lane
Boidot Cont.
Buffa
Detwiller
Jogi
Starmans
Zhao
Halle
Hu
Bosco
Manalo
Xia
Fardin 2010
Wei
Ghazoui
Li
Winter
Yang 2017
Fjeldbo
Mo
Wang 2020
Yang 2018 Prostate
Koong
Ning
Suh
Ragnum
Van Malenstein
Peters
Zou
Sun 2021
Seigneuric E2
Ghorbel
Shi
He
Seigneuric E0
Boidot Cyc.
Shou 2021
Seigneuric C.
Xu
Gao
Wang 2022
Shi 2021
Shou 2020
Trong
Sun 2020
Liu
Zhang 2020
Cell type
Experimental condition
Tissue type
Time (h)
O2

Supplementary Figure S4: Complex heatmap (mode)

Complex heatmap of signature p-values in hypoxic vs. non-hypoxic samples across GEO cell-line conditions in bulk RNAseq and microarray data, related to Figure 3. Conventions as in S2.

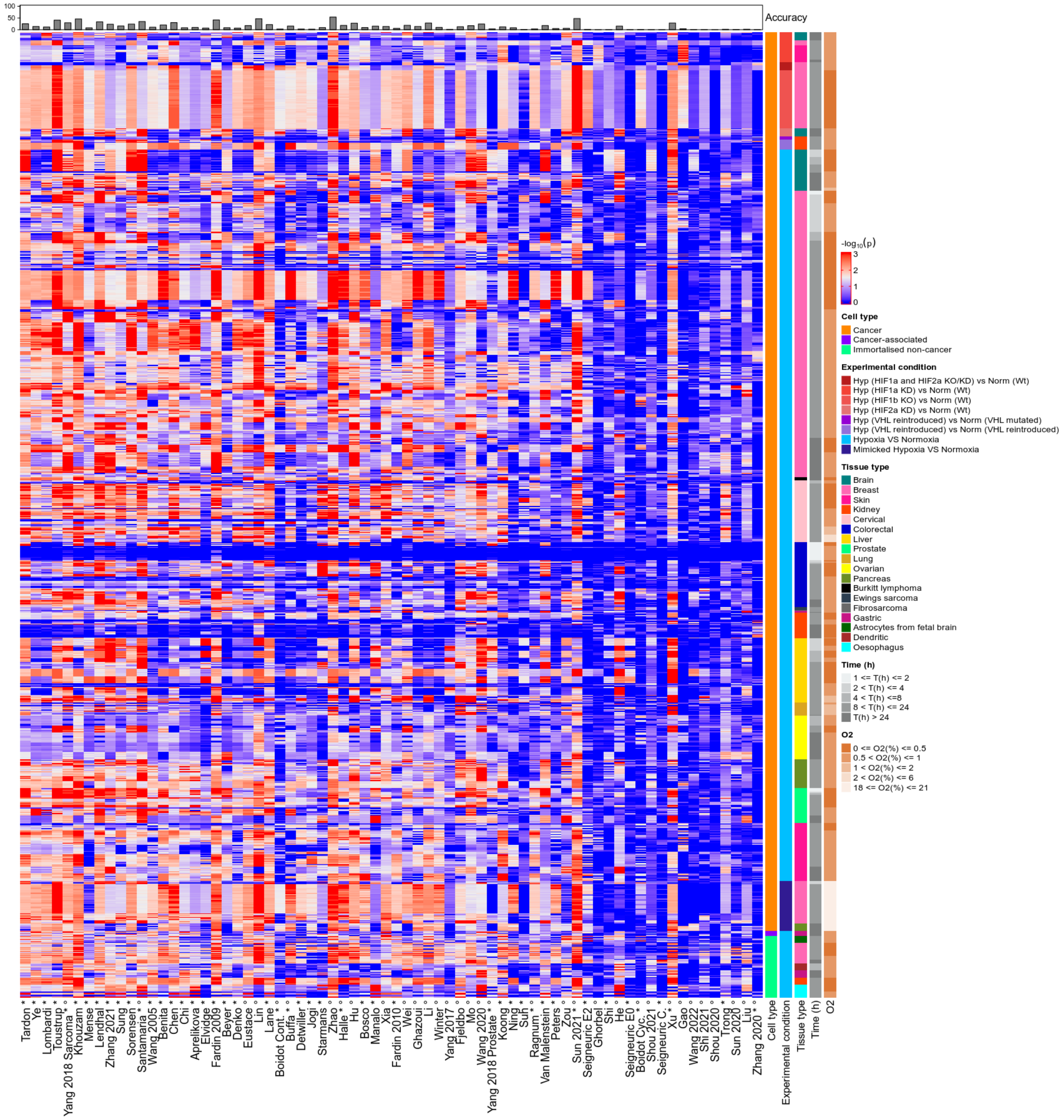
100
50
0
Accuracy
-log10(p)
3
2
1
0
Cell type
Cancer
Cancer-associated
Immortalised non-cancer
Experimental condition
Hyp (HIF1a and HIF2a KO/KD) vs Norm (Wt)
Hyp (HIF1a KD) vs Norm (Wt)
Hyp (HIF1b KO) vs Norm (Wt)
Hyp (HIF2a KD) vs Norm (Wt)
Hyp (VHL reintroduced) vs Norm (VHL mutated)
Hyp (VHL reintroduced) vs Norm (VHL reintroduced)
Hypoxia VS Normoxia
Mimicked Hypoxia VS Normoxia
Tissue type
Brain
Breast
Skin
Kidney
Cervical
Colorectal
Liver
Prostate
Lung
Ovarian
Pancreas
Burkitt lymphoma
Ewings sarcoma
Fibrosarcoma
Gastric
Astrocytes from fetal brain
Dendritic
Oesophagus
Time (h)
1 <= T(h) <= 2
2 < T(h) <= 4
4 < T(h) <=8
8 < T(h) <= 24
T(h) > 24
O2
0 <= O2(%) <= 0.5
0.5 < O2(%) <= 1
1 < O2(%) <= 2
2 < O2(%) <= 6
18 <= O2(%) <= 21
Tardon *
Ye *
Lombardi *
Toustrup *
Yang 2018 Sarcoma *
Khouzam *
Mense
Lendhal *
Zhang 2021 *
Sung *
Sorensen *
Santamaria *
Wang 2005 *
Benita *
Chen *
Chi *
Aprelikova *
Elvidge *
Fardin 2009 *
Beyer *
Denko *
Eustace
Lin *
Lane *
Boidot Cont. *
Buffa *
Detwiller *
Jogi *
Starmans
Zhao *
Halle *
Hu
Bosco *
Manalo *
Xia
Fardin 2010
Wei
Ghazoui
Li
Winter
Yang 2017
Fjeldbo
Mo
Wang 2020
Yang 2018 Prostate *
Koong *
Ning *
Suh *
Ragnum *
Van Malenstein *
Peters *
Zou
Sun 2021 *
Seigneuric E2 *
Ghorbel
Shi *
He
Seigneuric E0 *
Boidot Cyc. *
Shou 2021
Seigneuric C. *
Xu *
Gao
Wang 2022
Shi 2021
Shou 2020
Trong *
Sun 2020
Liu
Zhang 2020 *
Cell type
Experimental condition
Tissue type
Time (h)
O2

Supplementary Figure S5: Complex heatmap (midrange)

Complex heatmap of signature p-values in hypoxic vs. non-hypoxic samples across GEO cell-line conditions in bulk RNAseq and microarray data, related to Figure 3. Conventions as in S2.

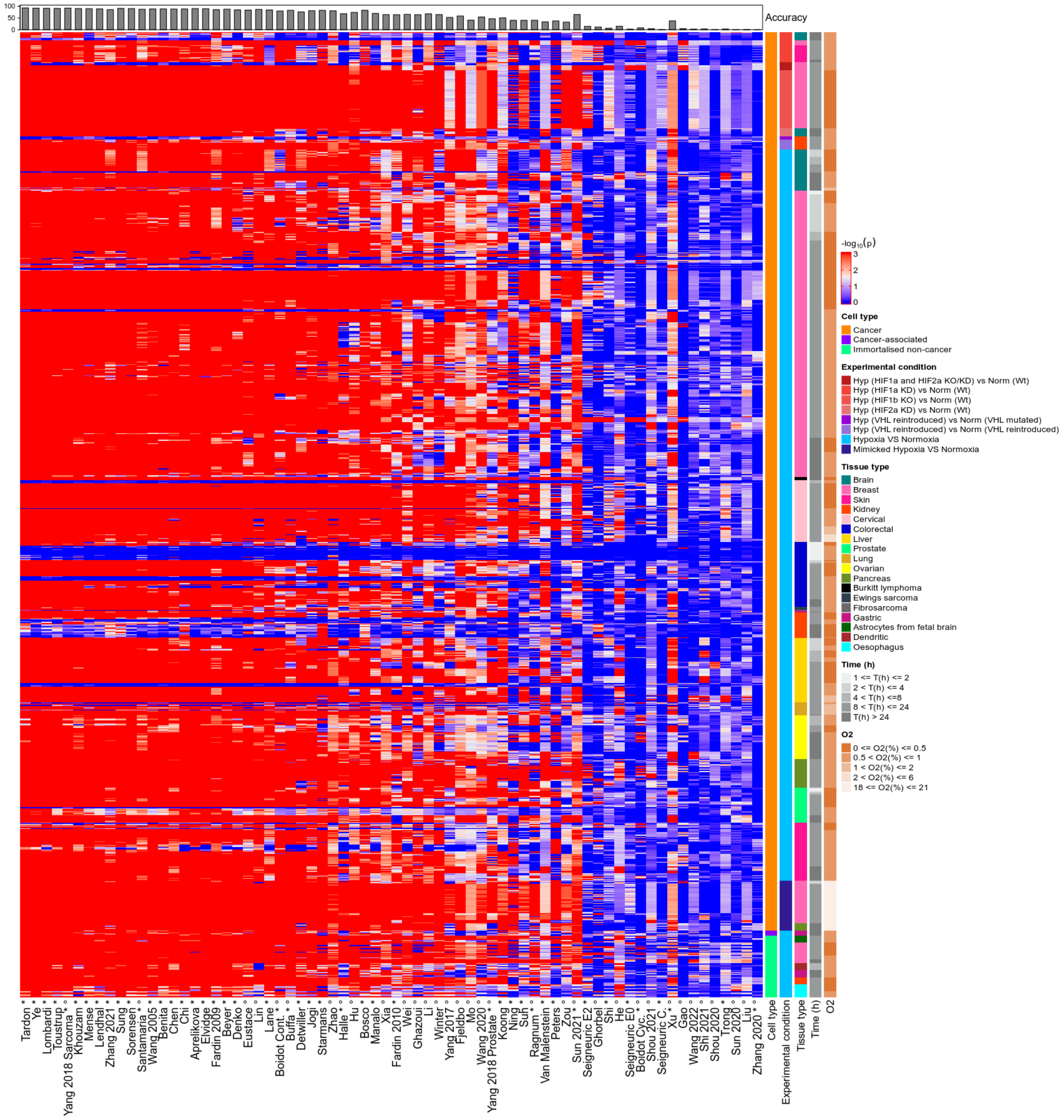
100
50
0
Accuracy
-log10(p)
3
2
1
0
Cell type
Cancer
Cancer-associated
Immortalised non-cancer
Experimental condition
Hyp (HIF1a and HIF2a KO/KD) vs Norm (Wt)
Hyp (HIF1a KD) vs Norm (Wt)
Hyp (HIF1b KO) vs Norm (Wt)
Hyp (HIF2a KD) vs Norm (Wt)
Hyp (VHL reintroduced) vs Norm (VHL mutated)
Hyp (VHL reintroduced) vs Norm (VHL reintroduced)
Hypoxia VS Normoxia
Mimicked Hypoxia VS Normoxia
Tissue type
Brain
Breast
Skin
Kidney
Cervical
Colorectal
Liver
Prostate
Lung
Ovarian
Pancreas
Burkitt lymphoma
Ewings sarcoma
Fibrosarcoma
Gastric
Astrocytes from fetal brain
Dendritic
Oesophagus
Time (h)
1 <= T(h) <= 2
2 < T(h) <= 4
4 < T(h) <=8
8 < T(h) <= 24
T(h) > 24
O2
0 <= O2(%) <= 0.5
0.5 < O2(%) <= 1
1 < O2(%) <= 2
2 < O2(%) <= 6
18 <= O2(%) <= 21
Tardon *
Ye *
Lombardi *
Toustrup *
Yang 2018 Sarcoma * ∘
Khouzam *
Mense *
Lendhal *
Zhang 2021 *
Sung *
Sorensen *
Santamaria * ∘
Wang 2005 *
Benita *
Chen *
Chi *
Aprelikova *
Elvidge *
Fardin 2009 *
Beyer *
Denko *
Eustace ∘
Lin ∘
Lane *
Boidot Cont. * ∘
Buffa * ∘
Detwiller *
Jogi *
Starmans *
Zhao ∘
Halle * ∘
Hu ∘
Bosco *
Manalo *
Xia ∘
Fardin 2010 *
Wei ∘
Ghazoui ∘
Li ∘
Winter ∘
Yang 2017 ∘
Fjeldbo ∘
Mo ∘
Wang 2020 ∘
Yang 2018 Prostate * ∘
Koong *
Ning *
Suh *
Ragnum * ∘
Van Malenstein *
Peters *
Zou ∘
Sun 2021 * ∘
Seigneuric E2 *
Ghorbel ∘
Shi *
He ∘
Seigneuric E0 *
Boidot Cyc. * ∘
Shou 2021 ∘
Seigneuric C. *
Xu * ∘
Gao ∘
Wang 2022 ∘
Shi 2021 ∘
Shou 2020 ∘
Trong *
Sun 2020 ∘
Liu ∘
Zhang 2020 * ∘
Cell type
Experimental condition
Tissue type
Time (h)
O2

Supplementary Figure S6: Complex heatmap (midhinge)

Complex heatmap of signature p-values in hypoxic vs. non-hypoxic samples across GEO cell-line conditions in bulk RNAseq and microarray data, related to Figure 3. Conventions as in S2.

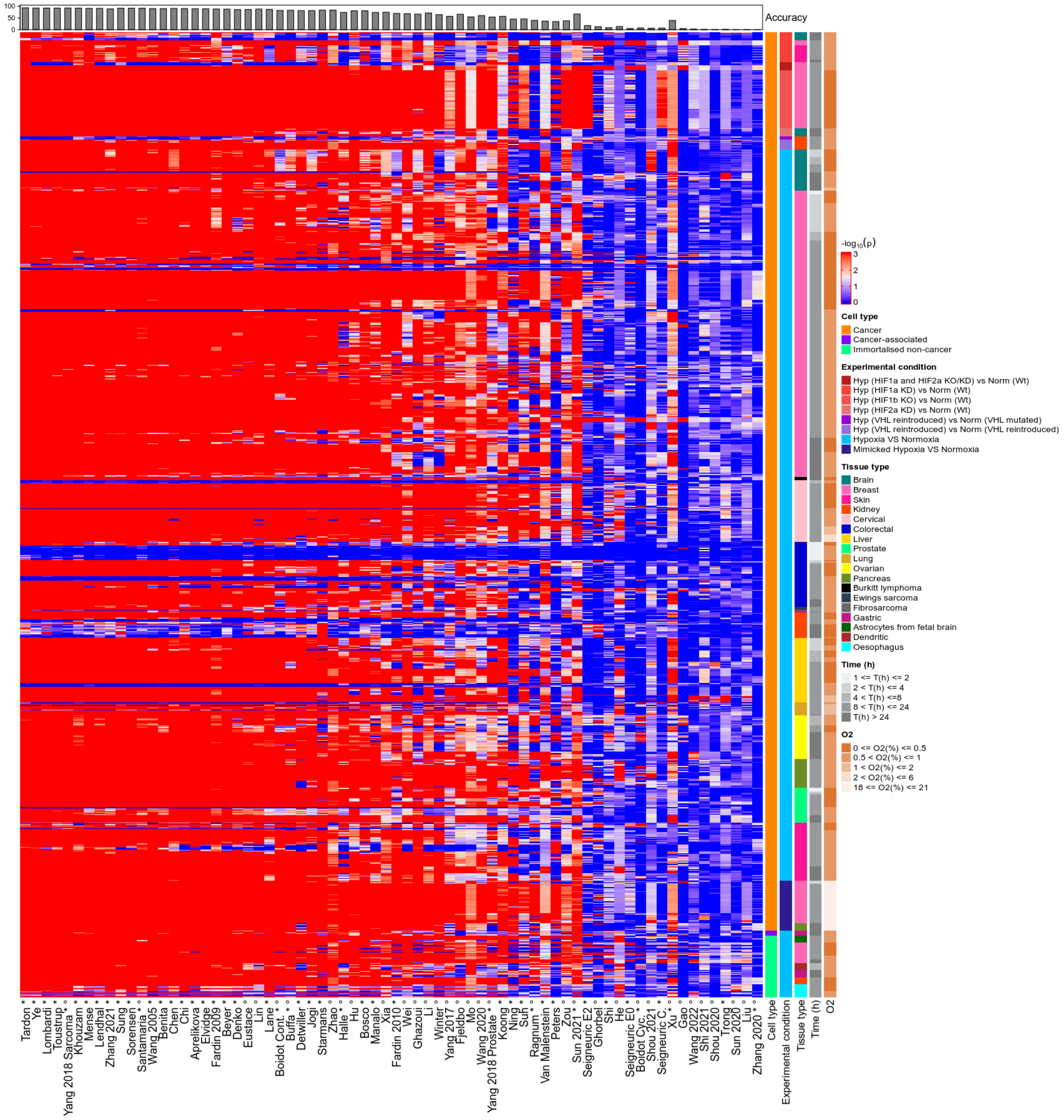
100
50
0
Accuracy
-log10(p)
3
2
1
0
Cell type
Cancer
Cancer-associated
Immortalised non-cancer
Experimental condition
Hyp (HIF1a and HIF2a KO/KD) vs Norm (Wt)
Hyp (HIF1a KD) vs Norm (Wt)
Hyp (HIF1b KO) vs Norm (Wt)
Hyp (HIF2a KD) vs Norm (Wt)
Hyp (VHL reintroduced) vs Norm (VHL mutated)
Hyp (VHL reintroduced) vs Norm (VHL reintroduced)
Hypoxia VS Normoxia
Mimicked Hypoxia VS Normoxia
Tissue type
Brain
Breast
Skin
Kidney
Cervical
Colorectal
Liver
Prostate
Lung
Ovarian
Pancreas
Burkitt lymphoma
Ewings sarcoma
Fibrosarcoma
Gastric
Astrocytes from fetal brain
Dendritic
Oesophagus
Time (h)
1 <= T(h) <= 2
2 < T(h) <= 4
4 < T(h) <=8
8 < T(h) <= 24
T(h) > 24
O2
0 <= O2(%) <= 0.5
0.5 < O2(%) <= 1
1 < O2(%) <= 2
2 < O2(%) <= 6
18 <= O2(%) <= 21
Tardon *
Ye *
Lombardi *
Toustrup *
Yang 2018 Sarcoma °
Khouzam *
Mense *
Lendhal *
Zhang 2021 *
Sung *
Sorensen *
Santamaria °
Wang 2005 *
Benita *
Chen *
Chi *
Aprelikova *
Elvidge *
Fardin 2009 *
Beyer *
Denko *
Eustace °
Lin °
Lane *
Boidot Cont. °
Buffa °
Detwiller *
Jogi *
Starmans *
Zhao °
Halle °
Hu °
Bosco *
Manalo *
Xia °
Fardin 2010 *
Wei °
Ghazoui °
Li °
Winter °
Yang 2017 °
Fjeldbo °
Mo °
Wang 2020 °
Yang 2018 Prostate °
Koong *
Ning *
Suh *
Ragnum °
Van Malenstein *
Peters *
Zou °
Sun 2021 °
Seigneuric E2 *
Ghorbel °
Shi *
He °
Seigneuric E0 *
Boidot Cyc. °
Shou 2021 °
Seigneuric C. *
Xu °
Gao °
Wang 2022 °
Shi 2021 °
Shou 2020 °
Trong *
Sun 2020 °
Liu °
Zhang 2020 °
Cell type
Experimental condition
Tissue type
Time (h)
O2

Supplementary Figure S7: Complex heatmap (trimean)

Complex heatmap of signature p-values in hypoxic vs. non-hypoxic samples across GEO cell-line conditions in bulk RNAseq and microarray data, related to Figure 3. Conventions as in S2.

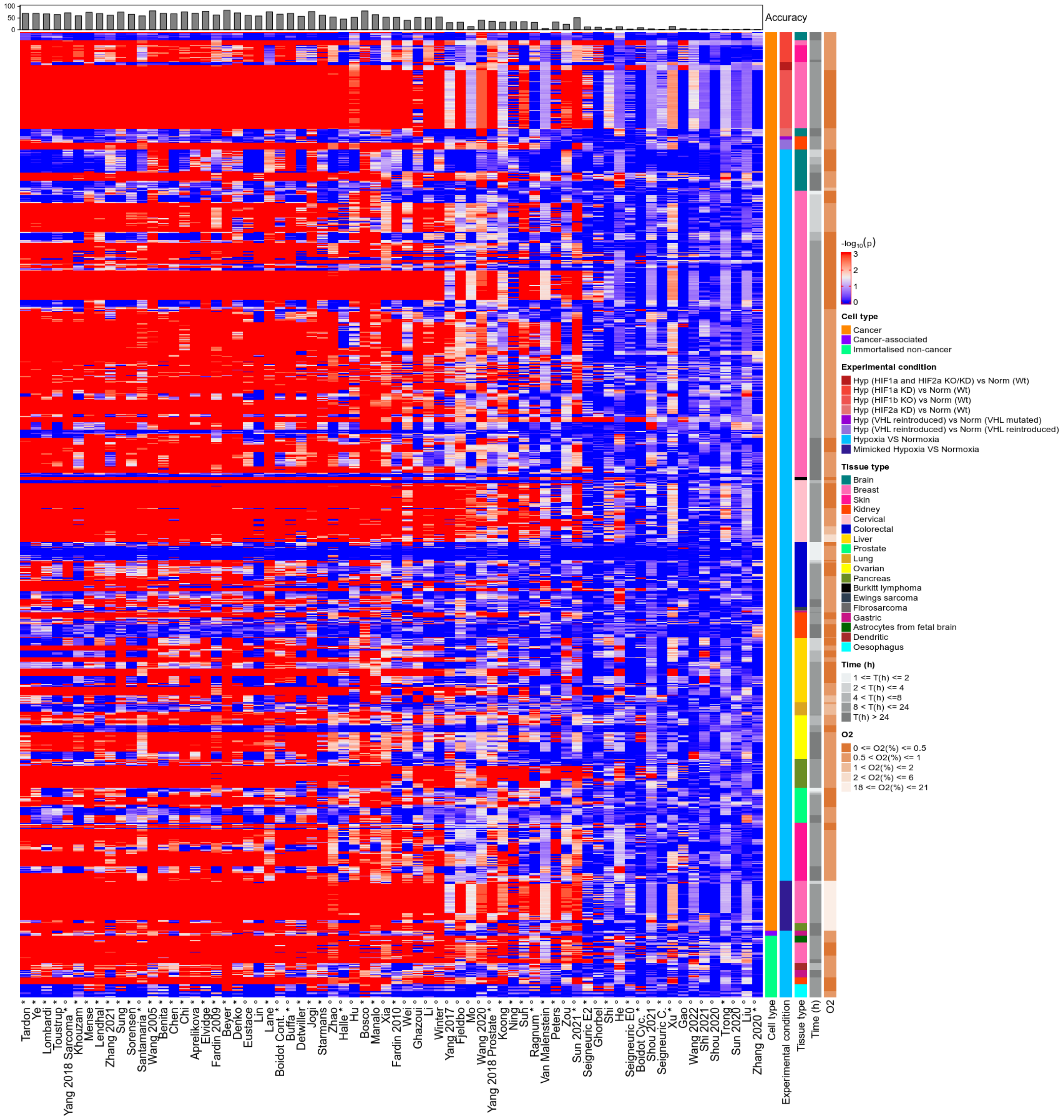
Accuracy
100
50
0
Tardon *
Ye *
Lombardi *
Toustrup *
Yang 2018 Sarcoma *
Khouzam *
Mense *
Lendhal *
Zhang 2021 *
Sung *
Sorensen *
Santamaria *
Wang 2005 *
Benita *
Chen *
Chi *
Aprelikova *
Elvidge *
Fardin 2009 *
Beyer *
Denko *
Eustace
Lin *
Lane *
Boidot Cont. *
Buffa *
Detwiller *
Jogi *
Starmans
Zhao
Halle *
Hu
Bosco *
Manalo *
Xia
Fardin 2010
Wei
Ghazoui
Li
Winter
Yang 2017
Fjeldbo
Mo
Wang 2020
Yang 2018 Prostate *
Koong *
Ning *
Suh *
Ragnum *
Van Malenstein *
Peters *
Zou
Sun 2021 *
Seigneuric E2 *
Ghorbel
Shi *
He
Seigneuric E0 *
Boidot Cyc. *
Shou 2021
Seigneuric C. *
Xu *
Gao
Wang 2022
Shi 2021
Shou 2020
Trong *
Sun 2020
Liu
Zhang 2020 *
Cell type
Experimental condition
Tissue type
Time (h)
O2
-log10(p)
3
2
1
0
Cell type
Cancer
Cancer-associated
Immortalised non-cancer
Experimental condition
Hyp (HIF1a and HIF2a KO/KD) vs Norm (Wt)
Hyp (HIF1a KD) vs Norm (Wt)
Hyp (HIF1b KO) vs Norm (Wt)
Hyp (HIF2a KD) vs Norm (Wt)
Hyp (VHL reintroduced) vs Norm (VHL mutated)
Hyp (VHL reintroduced) vs Norm (VHL reintroduced)
Hypoxia VS Normoxia
Mimicked Hypoxia VS Normoxia
Tissue type
Brain
Breast
Skin
Kidney
Cervical
Colorectal
Liver
Prostate
Lung
Ovarian
Pancreas
Burkitt lymphoma
Ewings sarcoma
Fibrosarcoma
Gastric
Astrocytes from fetal brain
Dendritic
Oesophagus
Time (h)
1 <= T(h) <= 2
2 < T(h) <= 4
4 < T(h) <=8
8 < T(h) <= 24
T(h) > 24
O2
0 <= O2(%) <= 0.5
0.5 < O2(%) <= 1
1 < O2(%) <= 2
2 < O2(%) <= 6
18 <= O2(%) <= 21

Supplementary Figure S8: Complex heatmap (IQR)

Complex heatmap of signature p-values in hypoxic vs. non-hypoxic samples across GEO cell-line conditions in bulk RNAseq and microarray data, related to Figure 3. Conventions as in S2.

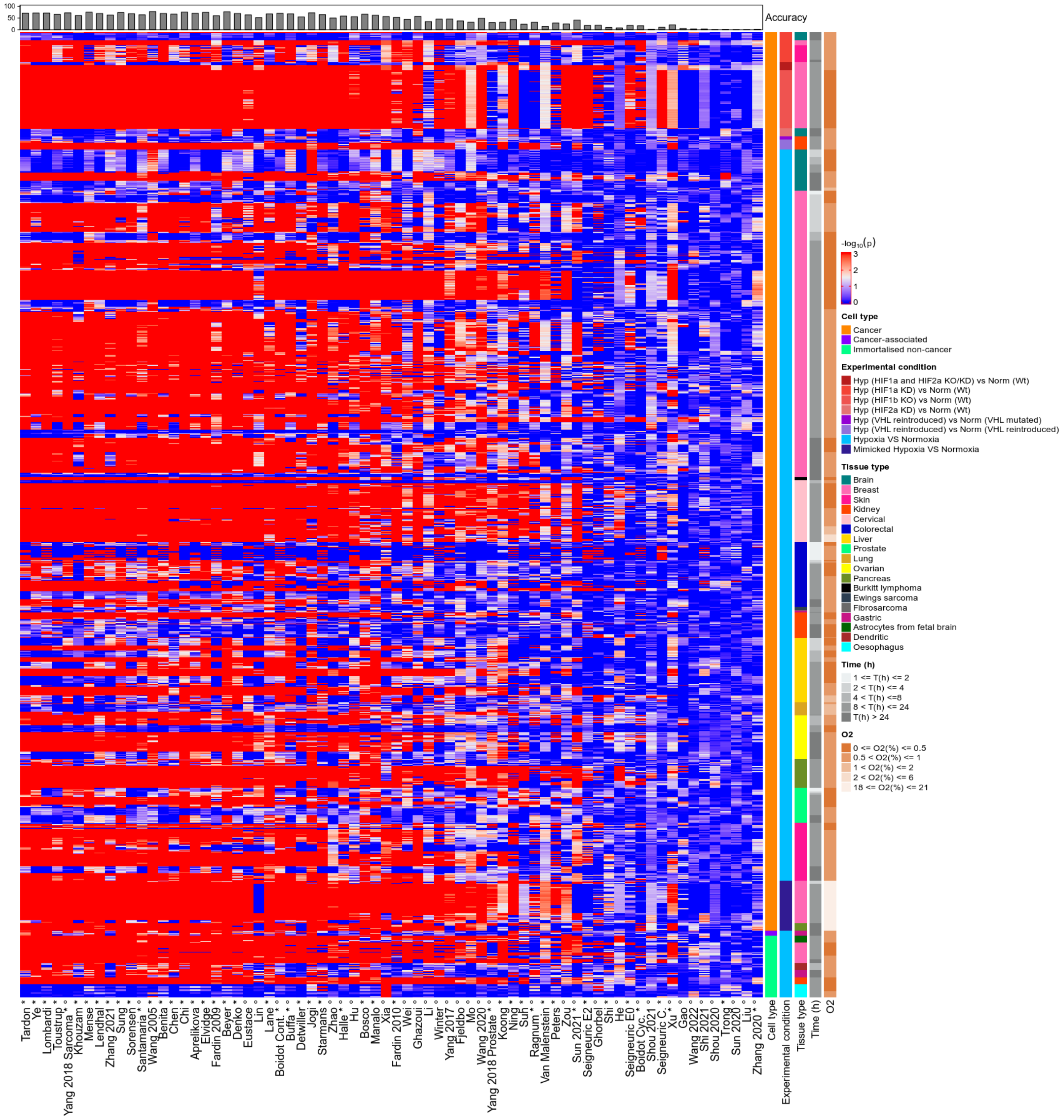

100
50
0
Accuracy
Tardon
Ye
Lombardi
Toustrup
Yang 2018 Sarcoma
Khouzam
Mense
Lendhal
Zhang 2021
Sung
Sorensen
Santamaria
Wang 2005
Benita
Chen
Chi
Aprelikova
Elvidge
Fardin 2009
Beyer
Denko
Eustace
Lin
Lane
Boidot Cont.
Buffa
Detwiller
Jogi
Starmans
Zhao
Halle
Hu
Bosco
Manalo
Xia
Fardin 2010
Wei
Ghazoui
Li
Winter
Yang 2017
Fjeldbo
Mo
Wang 2020
Yang 2018 Prostate
Koong
Ning
Suh
Ragnum
Van Malenstein
Peters
Zou
Sun 2021
Seigneuric E2
Ghorbel
Shi
He
Seigneuric E0
Boidot Cyc.
Shou 2021
Seigneuric C.
Xu
Gao
Wang 2022
Shi 2021
Shou 2020
Trong
Sun 2020
Liu
Zhang 2020
Cell type
Experimental condition
Tissue type
Time (h)
O2
-log10(p)
3
2
1
0
Cell type
Cancer
Cancer-associated
Immortalised non-cancer
Experimental condition
Hyp (HIF1a and HIF2a KO/KD) vs Norm (Wt)
Hyp (HIF1a KD) vs Norm (Wt)
Hyp (HIF1b KO) vs Norm (Wt)
Hyp (HIF2a KD) vs Norm (Wt)
Hyp (VHL reintroduced) vs Norm (VHL mutated)
Hyp (VHL reintroduced) vs Norm (VHL reintroduced)
Hypoxia VS Normoxia
Mimicked Hypoxia VS Normoxia
Tissue type
Brain
Breast
Skin
Kidney
Cervical
Colorectal
Liver
Prostate
Lung
Ovarian
Pancreas
Burkitt lymphoma
Ewings sarcoma
Fibrosarcoma
Gastric
Astrocytes from fetal brain
Dendritic
Oesophagus
Time (h)
1 <= T(h) <= 2
2 < T(h) <= 4
4 < T(h) <=8
8 < T(h) <= 24
T(h) > 24
O2
0 <= O2(%) <= 0.5
0.5 < O2(%) <= 1
1 < O2(%) <= 2
2 < O2(%) <= 6
18 <= O2(%) <= 21

Supplementary Figure S9: Complex heatmap (MAD)

Complex heatmap of signature p-values in hypoxic vs. non-hypoxic samples across GEO cell-line conditions in bulk RNAseq and microarray data, related to Figure 3. Conventions as in S2.

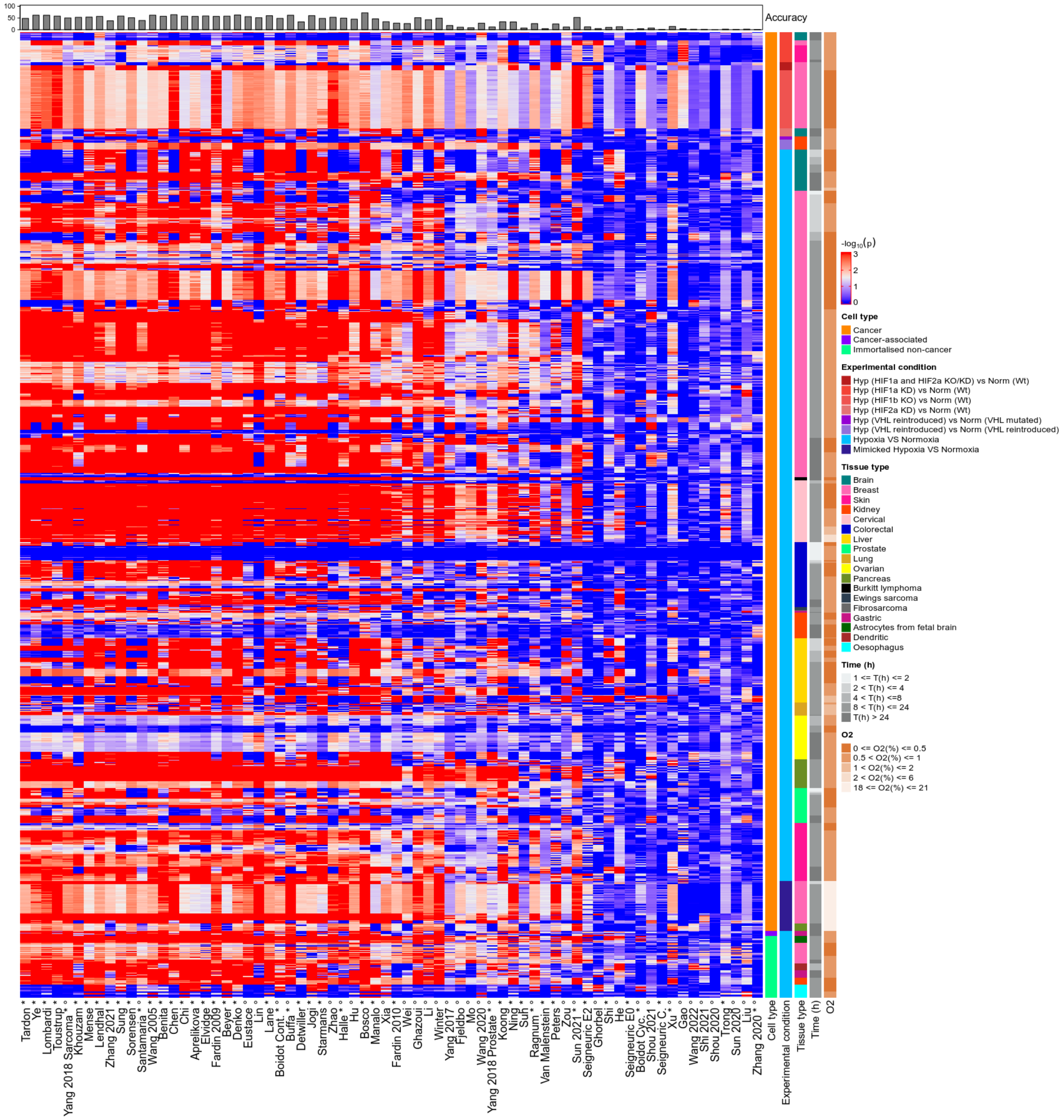
100
50
0
Accuracy
-log10(p)
3
2
1
0
Cell type
Cancer
Cancer-associated
Immortalised non-cancer
Experimental condition
Hyp (HIF1a and HIF2a KO/KD) vs Norm (Wt)
Hyp (HIF1a KD) vs Norm (Wt)
Hyp (HIF1b KO) vs Norm (Wt)
Hyp (HIF2a KD) vs Norm (Wt)
Hyp (VHL reintroduced) vs Norm (VHL mutated)
Hyp (VHL reintroduced) vs Norm (VHL reintroduced)
Hypoxia VS Normoxia
Mimicked Hypoxia VS Normoxia
Tissue type
Brain
Breast
Skin
Kidney
Cervical
Colorectal
Liver
Prostate
Lung
Ovarian
Pancreas
Burkitt lymphoma
Ewings sarcoma
Fibrosarcoma
Gastric
Astrocytes from fetal brain
Dendritic
Oesophagus
Time (h)
1 <= T(h) <= 2
2 < T(h) <= 4
4 < T(h) <=8
8 < T(h) <= 24
T(h) > 24
O2
0 <= O2(%) <= 0.5
0.5 < O2(%) <= 1
1 < O2(%) <= 2
2 < O2(%) <= 6
18 <= O2(%) <= 21
Tardon
Ye
Lombardi
Toustrup
Yang 2018 Sarcoma
Khouzam
Mense
Lendhal
Zhang 2021
Sung
Sorensen
Santamaria
Wang 2005
Benita
Chen
Chi
Aprelikova
Elvidge
Fardin 2009
Beyer
Denko
Eustace
Lin
Lane
Boidot Cont.
Buffa
Detwiller
Jogi
Starmans
Zhao
Halle
Hu
Bosco
Manalo
Xia
Fardin 2010
Wei
Ghazoui
Li
Winter
Yang 2017
Fjeldbo
Mo
Wang 2020
Yang 2018 Prostate
Koong
Ning
Suh
Ragnum
Van Malenstein
Peters
Zou
Sun 2021
Seigneuric E2
Ghorbel
Shi
He
Seigneuric E0
Boidot Cyc.
Shou 2021
Seigneuric C.
Xu
Gao
Wang 2022
Shi 2021
Shou 2020
Trong
Sun 2020
Liu
Zhang 2020
Cell type
Experimental condition
Tissue type
Time (h)
O2

Supplementary Figure S10: Complex heatmap (AAD)

Complex heatmap of signature p-values in hypoxic vs. non-hypoxic samples across GEO cell-line conditions in bulk RNAseq and microarray data, related to Figure 3. Conventions as in S2.

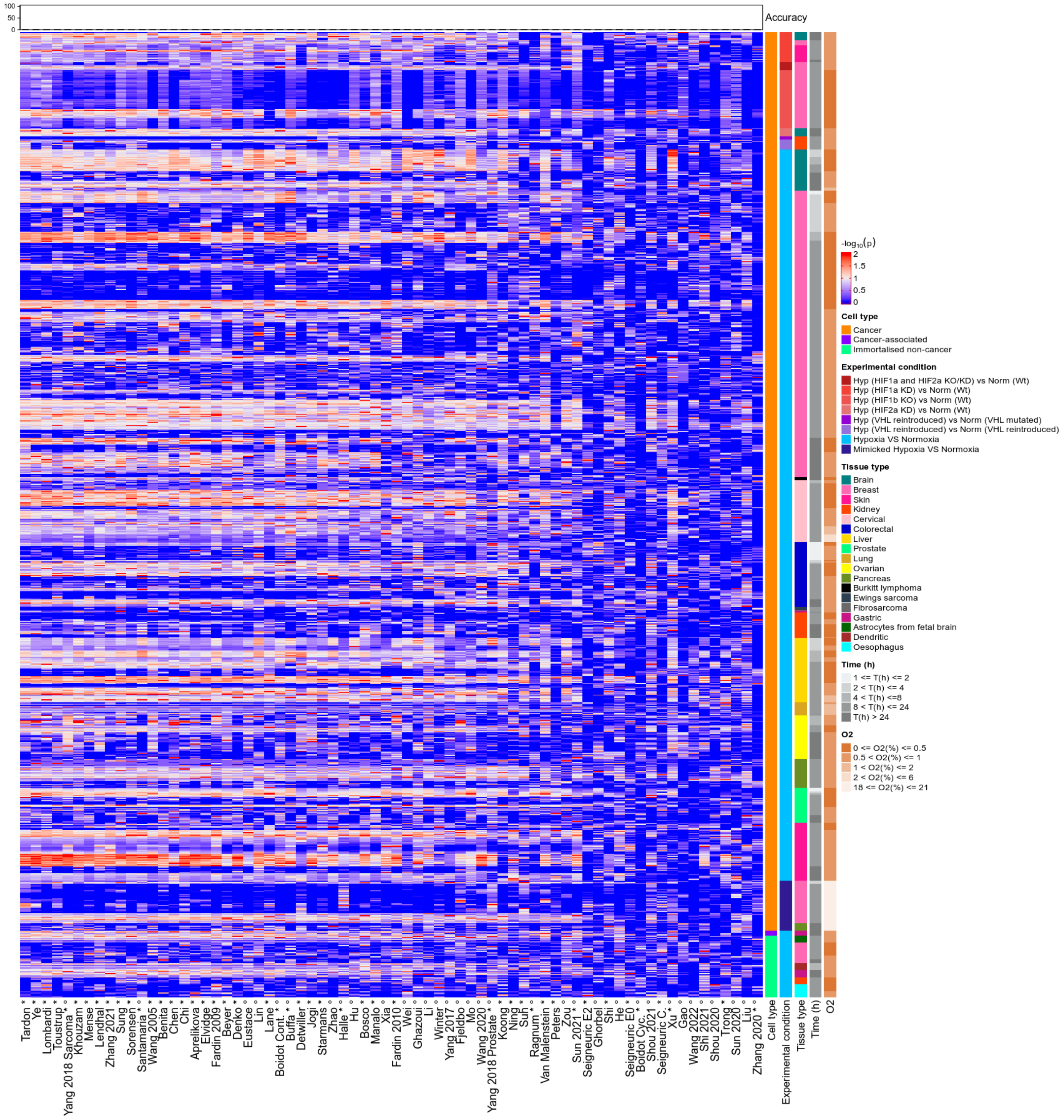

100
50
0
Accuracy
-log10(p)
2
1.5
1
0.5
0
Cell type
Cancer
Cancer-associated
Immortalised non-cancer
Experimental condition
Hyp (HIF1a and HIF2a KO/KD) vs Norm (Wt)
Hyp (HIF1a KD) vs Norm (Wt)
Hyp (HIF1b KO) vs Norm (Wt)
Hyp (HIF2a KD) vs Norm (Wt)
Hyp (VHL reintroduced) vs Norm (VHL mutated)
Hyp (VHL reintroduced) vs Norm (VHL reintroduced)
Hypoxia VS Normoxia
Mimicked Hypoxia VS Normoxia
Tissue type
Brain
Breast
Skin
Kidney
Cervical
Colorectal
Liver
Prostate
Lung
Ovarian
Pancreas
Burkitt lymphoma
Ewings sarcoma
Fibrosarcoma
Gastric
Astrocytes from fetal brain
Dendritic
Oesophagus
Time (h)
1 <= T(h) <= 2
2 < T(h) <= 4
4 < T(h) <=8
8 < T(h) <= 24
T(h) > 24
O2
0 <= O2(%) <= 0.5
0.5 < O2(%) <= 1
1 < O2(%) <= 2
2 < O2(%) <= 6
18 <= O2(%) <= 21
Tardon *
Ye *
Lombardi *
Toustrup *
Yang 2018 Sarcoma *
Khouzam *
Mense *
Lendhal *
Zhang 2021 *
Sung *
Sorensen *
Santamaria °
Wang 2005 *
Benita *
Chen *
Chi *
Aprelikova *
Elvidge *
Fardin 2009 *
Beyer *
Denko *
Eustace °
Lin *
Lane *
Boidot Cont. *
Buffa *
Detwiller *
Jogi *
Starmans *
Zhao °
Halle *
Hu °
Bosco *
Manalo *
Xia *
Fardin 2010 °
Wei °
Ghazoui °
Li °
Winter °
Yang 2017 °
Fjeldbo °
Mo °
Wang 2020 °
Yang 2018 Prostate *
Koong *
Ning *
Suh °
Ragnum *
Van Malenstein *
Peters *
Zou °
Sun 2021 *
Seigneuric E2 *
Ghorbel °
Shi *
He °
Seigneuric E0 *
Boidot Cyc. *
Shou 2021 °
Seigneuric C. *
Xu *
Gao °
Wang 2022 °
Shi 2021 °
Shou 2020 °
Trong *
Sun 2020 °
Liu °
Zhang 2020 *
Cell type
Experimental condition
Tissue type
Time (h)
O2

Supplementary Figure S11: Complex heatmap (ssGSEA)

Complex heatmap of signature p-values in hypoxic vs. non-hypoxic samples across GEO cell-line conditions in bulk RNAseq and microarray data, related to Figure 3. Conventions as in S2.

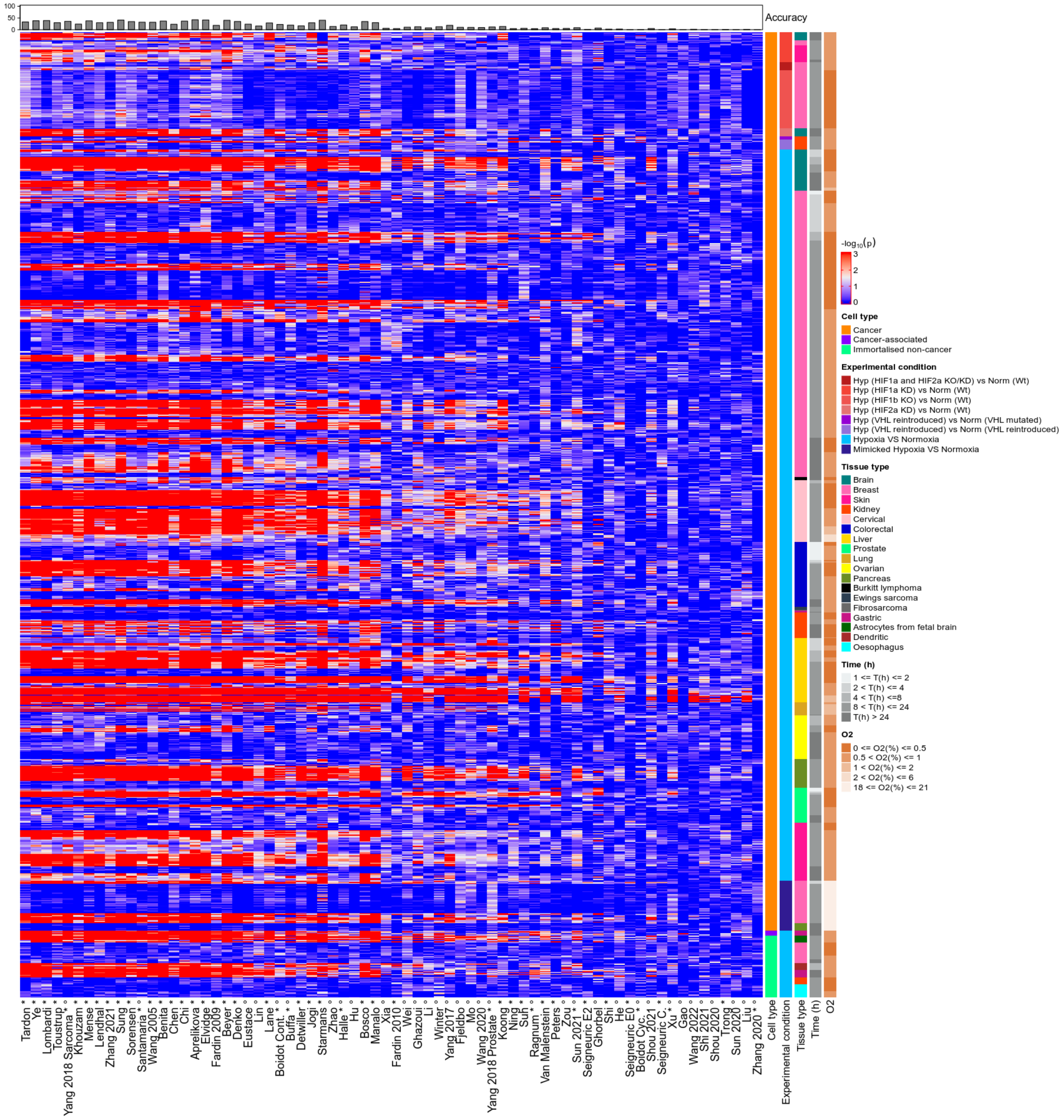

100
50
0
Accuracy
-log10(p)
3
2
1
0
Cell type
Cancer
Cancer-associated
Immortalised non-cancer
Experimental condition
Hyp (HIF1a and HIF2a KO/KD) vs Norm (Wt)
Hyp (HIF1a KD) vs Norm (Wt)
Hyp (HIF1b KO) vs Norm (Wt)
Hyp (HIF2a KD) vs Norm (Wt)
Hyp (VHL reintroduced) vs Norm (VHL mutated)
Hyp (VHL reintroduced) vs Norm (VHL reintroduced)
Hypoxia VS Normoxia
Mimicked Hypoxia VS Normoxia
Tissue type
Brain
Breast
Skin
Kidney
Cervical
Colorectal
Liver
Prostate
Lung
Ovarian
Pancreas
Burkitt lymphoma
Ewings sarcoma
Fibrosarcoma
Gastric
Astrocytes from fetal brain
Dendritic
Oesophagus
Time (h)
1 <= T(h) <= 2
2 < T(h) <= 4
4 < T(h) <=8
8 < T(h) <= 24
T(h) > 24
O2
0 <= O2(%) <= 0.5
0.5 < O2(%) <= 1
1 < O2(%) <= 2
2 < O2(%) <= 6
18 <= O2(%) <= 21
Tardon
Ye
Lombardi
Toustrup
Yang 2018 Sarcoma
Khouzam
Mense
Lendhal
Zhang 2021
Sung
Sorensen
Santamaria
Wang 2005
Benita
Chen
Chi
Aprelikova
Elvidge
Fardin 2009
Beyer
Denko
Eustace
Lin
Lane
Boidot Corr.
Buffa
Detwiller
Jogi
Starmans
Zhao
Halle
Hu
Bosco
Manalo
Xia
Fardin 2010
Wei
Ghazoui
Li
Winter
Yang 2017
Fjeldbo
Mo
Wang 2020
Yang 2018 Prostate
Koong
Ning
Suh
Ragnum
Van Malenstein
Peters
Zou
Sun 2021
Seigneuric E2
Ghorbel
Shi
He
Seigneuric E0
Boidot Cyc.
Shou 2021
Seigneuric C.
Xu
Gao
Wang 2022
Shi 2021
Shou 2020
Trong
Sun 2020
Liu
Zhang 2020
Cell type
Experimental condition
Tissue type
Time (h)
O2

Supplementary Figure S12: Complex heatmap (GSVA)

Complex heatmap of signature p-values in hypoxic vs. non-hypoxic samples across GEO cell-line conditions in bulk RNAseq and microarray data, related to Figure 3. Conventions as in S2.

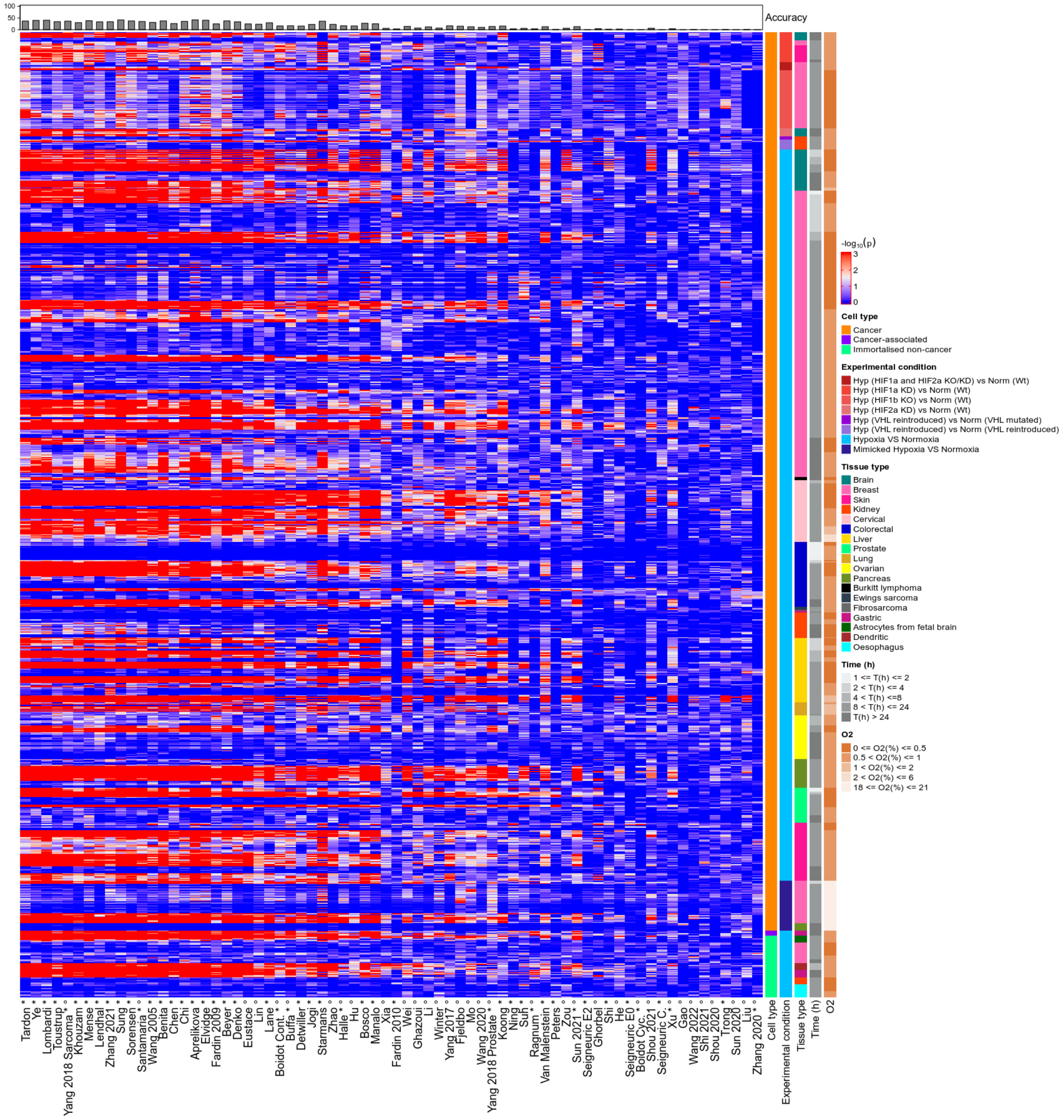

Accuracy
100
50
0
-log10(p)
3
2
1
0
Cell type
Cancer
Cancer-associated
Immortalised non-cancer
Experimental condition
Hyp (HIF1a and HIF2a KO/KD) vs Norm (Wt)
Hyp (HIF1a KD) vs Norm (Wt)
Hyp (HIF1b KO) vs Norm (Wt)
Hyp (HIF2a KD) vs Norm (Wt)
Hyp (VHL reintroduced) vs Norm (VHL mutated)
Hyp (VHL reintroduced) vs Norm (VHL reintroduced)
Hypoxia VS Normoxia
Mimicked Hypoxia VS Normoxia
Tissue type
Brain
Breast
Skin
Kidney
Cervical
Colorectal
Liver
Prostate
Lung
Ovarian
Pancreas
Burkitt lymphoma
Ewings sarcoma
Fibrosarcoma
Gastric
Astrocytes from fetal brain
Dendritic
Oesophagus
Time (h)
1 <= T(h) <= 2
2 < T(h) <= 4
4 < T(h) <=8
8 < T(h) <= 24
T(h) > 24
O2
0 <= O2(%) <= 0.5
0.5 < O2(%) <= 1
1 < O2(%) <= 2
2 < O2(%) <= 6
18 <= O2(%) <= 21
Tardon *
Ye *
Lombardi *
Yang 2018 Sarcoma *
Toustrup *
Khouzam *
Mense *
Lendhal *
Zhang 2021 *
Sung *
Sorensen *
Santamaria *
Wang 2005 *
Benita *
Chen °
Chi *
Aprelikova *
Elvidge *
Fardin 2009 *
Beyer *
Denko *
Eustace °
Lin *
Lane *
Boidot Cort. *
Buffa *
Detwiller *
Jogi *
Starmans *
Zhao °
Halle *
Hu °
Bosco *
Manalo *
Xia °
Fardin 2010 *
Wei °
Ghazoui °
Li °
Winter °
Yang 2017 °
Fjeldbo °
Mo °
Wang 2020 °
Yang 2018 Prostate *
Koong *
Ning *
Suh *
Ragnum *
Van Malenstein *
Peters *
Zou °
Sun 2021 *
Seigneuric E2 *
Ghorbel °
Shi *
He °
Seigneuric E0 *
Boidot Cyc. *
Shou 2021 °
Seigneuric C. *
Xu *
Gao °
Wang 2022 °
Shi 2021 °
Shou 2020 °
Trong *
Sun 2020 °
Liu °
Zhang 2020 *
Cell type
Experimental condition
Tissue type
Time (h)
O2

Supplementary Figure S13: Complex heatmaps (Z-score)

Complex heatmap of signature p-values in hypoxic vs. non-hypoxic samples across GEO cell-line conditions in bulk RNAseq and microarray data, related to Figure 3. Conventions as in S2.

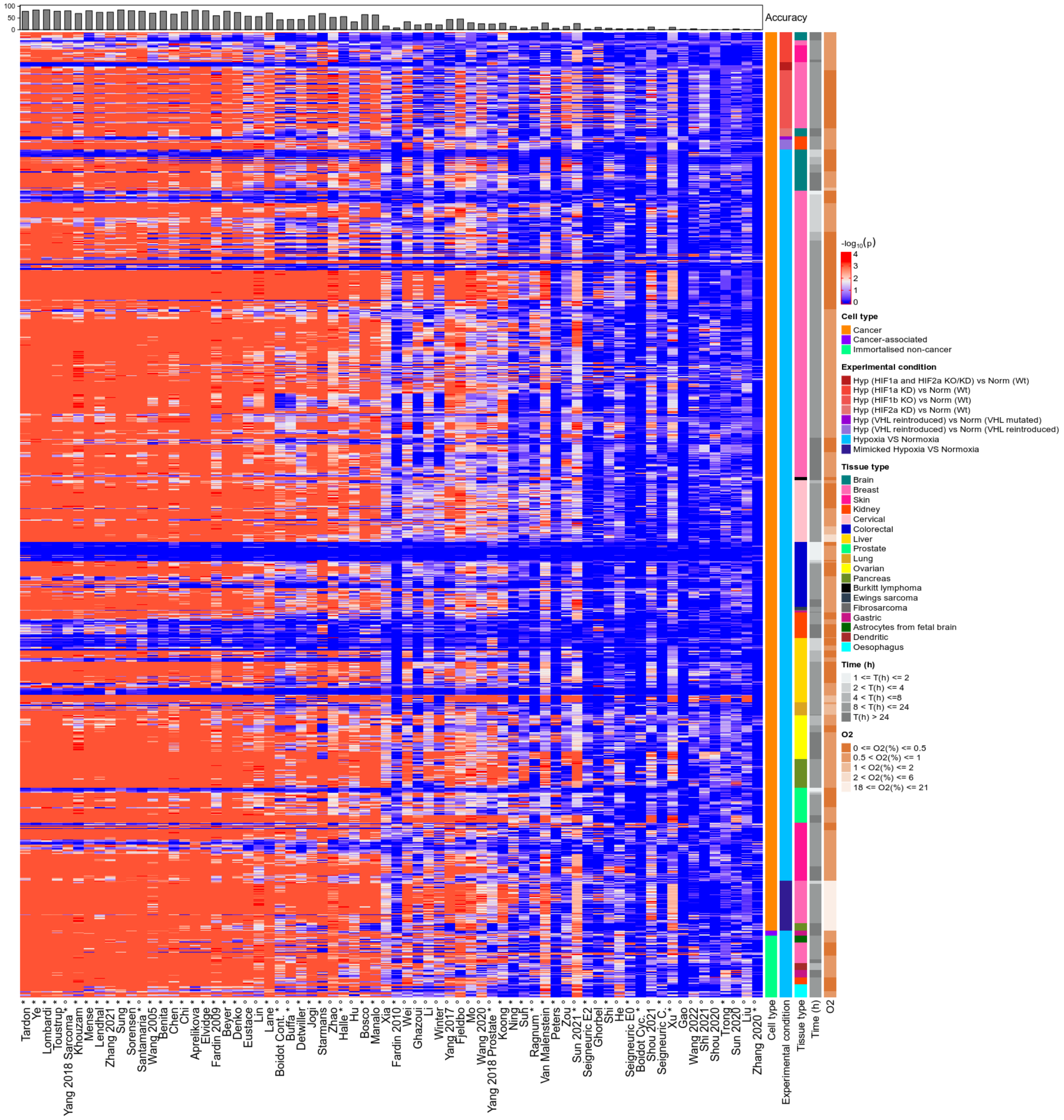
100
50
0
Accuracy
Tardon
Ye
Lombardi
Toustrup
Yang 2018 Sarcoma
Khouzam
Mense
Lendhal
Zhang 2021
Sung
Sorensen
Santamaria
Wang 2005
Benita
Chen
Chi
Aprelikova
Elvidge
Fardin 2009
Beyer
Denko
Eustace
Lin
Lane
Boidot Cort.
Buffa
Detwiller
Jogi
Starmans
Zhao
Halle
Hu
Bosco
Manalo
Xia
Fardin 2010
Wei
Ghazoui
Li
Winter
Yang 2017
Fjeldbo
Mo
Wang 2020
Yang 2018 Prostate
Koong
Ning
Suh
Ragnum
Van Malenstein
Peters
Zou
Sun 2021
Seigneuric E2
Ghorbel
Shi
He
Seigneuric E0
Boidot Cyc.
Shou 2021
Seigneuric C.
Xu
Gao
Wang 2022
Shi 2021
Shou 2020
Trong
Sun 2020
Liu
Zhang 2020
Cell type
Experimental condition
Tissue type
Time (h)
O2
-log10(p)
4
3
2
1
0
Cell type
Cancer
Cancer-associated
Immortalised non-cancer
Experimental condition
Hyp (HIF1a and HIF2a KO/KD) vs Norm (Wt)
Hyp (HIF1a KD) vs Norm (Wt)
Hyp (HIF1b KO) vs Norm (Wt)
Hyp (HIF2a KD) vs Norm (Wt)
Hyp (VHL reintroduced) vs Norm (VHL mutated)
Hyp (VHL reintroduced) vs Norm (VHL reintroduced)
Hypoxia VS Normoxia
Mimicked Hypoxia VS Normoxia
Tissue type
Brain
Breast
Skin
Kidney
Cervical
Colorectal
Liver
Prostate
Lung
Ovarian
Pancreas
Burkitt lymphoma
Ewings sarcoma
Fibrosarcoma
Gastric
Astrocytes from fetal brain
Dendritic
Oesophagus
Time (h)
1 <= T(h) <= 2
2 < T(h) <= 4
4 < T(h) <=8
8 < T(h) <= 24
T(h) > 24
O2
0 <= O2(%) <= 0.5
0.5 < O2(%) <= 1
1 < O2(%) <= 2
2 < O2(%) <= 6
18 <= O2(%) <= 21

Supplementary Figure S14: Complex heatmap (NCS)

Complex heatmap of signature p-values in hypoxic vs. non-hypoxic samples across GEO cell-line conditions in bulk RNAseq and microarray data, related to Figure 3. Conventions as in S2.

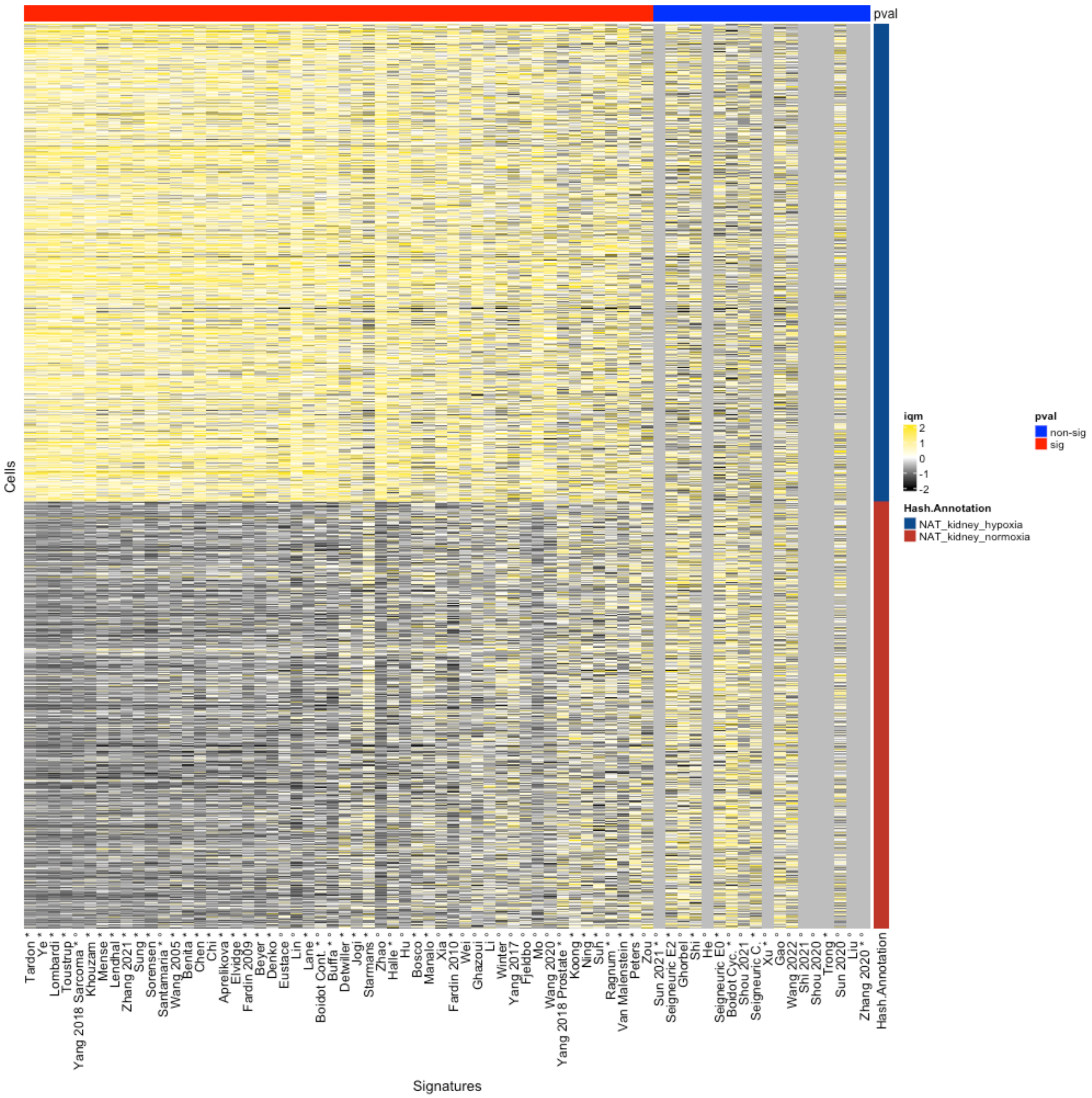
pval
Cells
Signatures
iqm
2
1
0
-1
-2
pval
non-sig
sig
Hash.Annotation
NAT_kidney_hypoxia
NAT_kidney_normoxia
Tardon
Ye
Lombardi
Toustrup
Yang 2018 Sarcoma
Khouzam
Mense
Lendhal
Zhang 2021
Sung
Sorensen
Santamaria
Wang 2005
Benita
Chen
Chi
Aprelikova
Elvidge
Fardin 2009
Beyer
Denko
Eustace
Lin
Lane
Boidot Cont.
Buffa
Detwiller
Jogi
Starmans
Zhao
Halle
Hu
Bosco
Manalo
Xia
Fardin 2010
Wei
Ghazoui
Li
Winter
Yang 2017
Fjeldbo
Mo
Wang 2020
Yang 2018 Prostate
Koong
Ning
Sun
Ragnum
Van Malenstein
Peters
Zou
Sun 2021
Seigneuric E2
Ghorbel
Shi
He
Seigneuric E0
Boidot Cyc.
Shou 2021
Seigneuric C.
Xu
Gao
Wang 2022
Shi 2021
Shou 2020
Trong
Sun 2020
Liu
Zhang 2020
Hash.Annotation

Supplementary Figure S15: Performance of the Tardon signature using the IQM in scRNA-Seq data for NAT human kidney (GSE200207) under normoxic and hypoxic conditions

Complex heatmap of IQM hypoxia score for hypoxic vs. normoxic cells in Normal Adjacent Tissue (NAT) from human kidney (GSE200207). Signatures on the x-axis are ordered based on their accuracy in the IQM score from Figure 3 for ease of comparison, related to Figure 4. The heatmap displays ranked-normalised hypoxia score values for each signature.

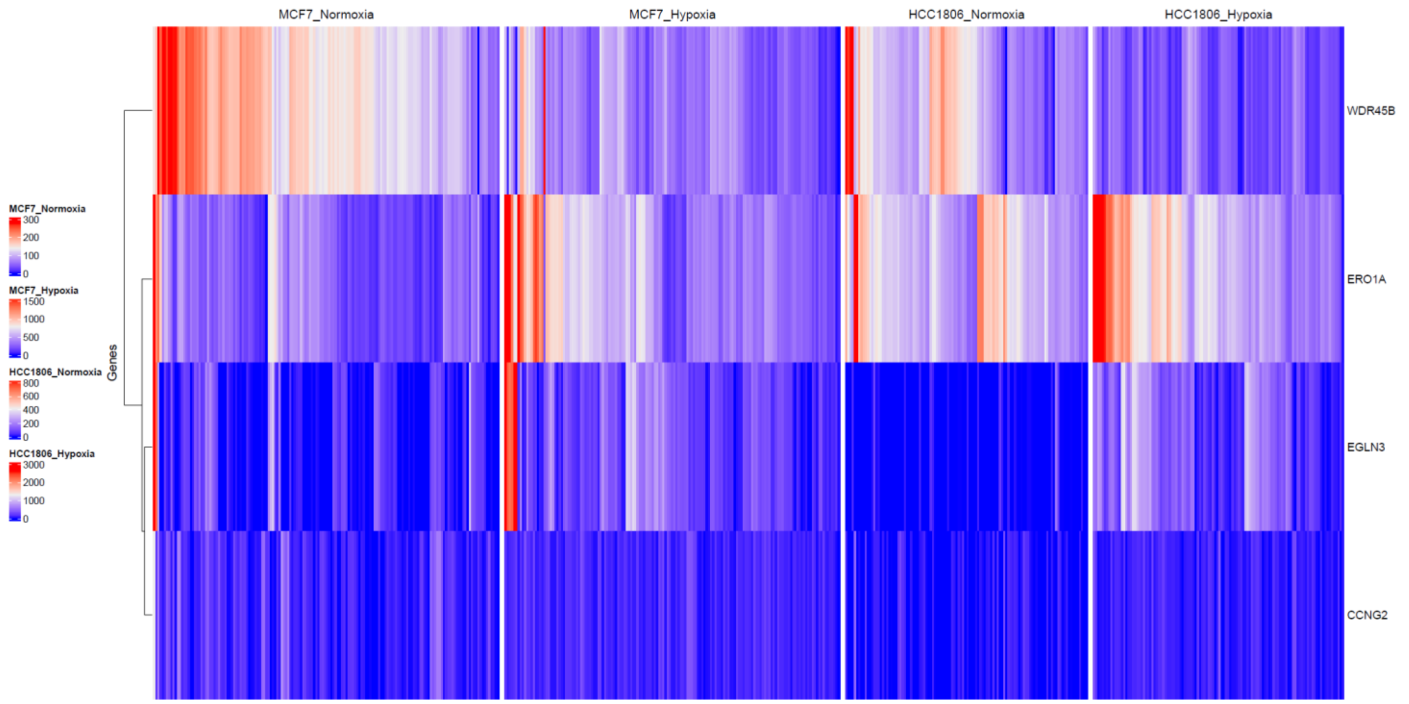
MCF7_Normoxia
MCF7_Hypoxia
HCC1806_Normoxia
HCC1806_Hypoxia
WDR45B
ERO1A
EGLN3
CCNG2
Genes
MCF7_Normoxia
300
200
100
0
MCF7_Hypoxia
1500
1000
500
0
HCC1806_Normoxia
800
600
400
200
0
HCC1806_Hypoxia
3000
2000
1000
0

Supplementary Figure S16: van Malenstein expression heatmaps clearly show dominance of ERO1A and WDR45B

Heatmaps depicting expression levels of van Malestein features in normoxic and hypoxic subsets from MCF7 and HCC1806 single-cell datasets. Rows represent signature features, columns represent individual cells, and colour intensities indicate library size-adjusted expression values. Notably, ERO1A exhibits elevated expression in hypoxia and reduced in normoxia, while WDR45B displays the inverse. This contrasting expression pattern leads to a diminished intra-signature correlation, affecting the signature's performance in single-cell data, related to Figure 4.

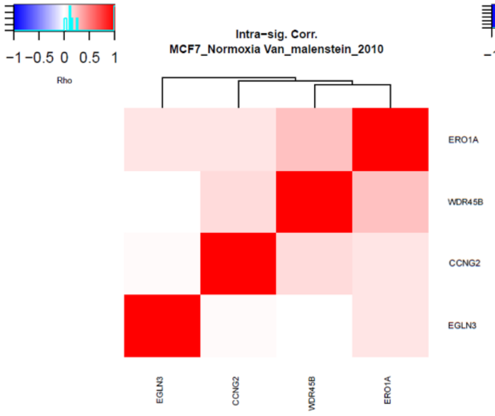

Intra−sig. Corr.
MCF7_Normoxia Van_malenstein_2010
−1 −0.5 0 0.5 1
Rho
ERO1A
WDR45B
CCNG2
EGLN3
EGLN3
CCNG2
WDR45B
ERO1A

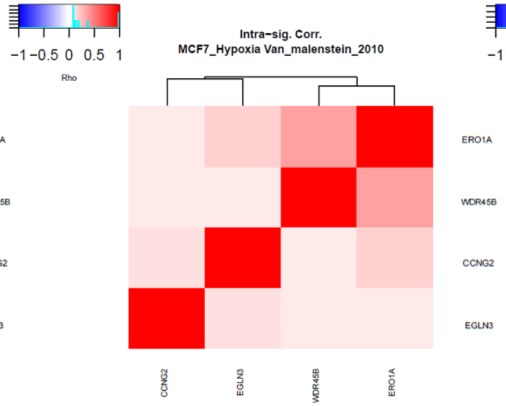

Intra−sig. Corr.
MCF7_Hypoxia Van_malenstein_2010
−1 −0.5 0 0.5 1
Rho
ERO1A
WDR45B
CCNG2
EGLN3
CCNG2
EGLN3
WDR45B
ERO1A

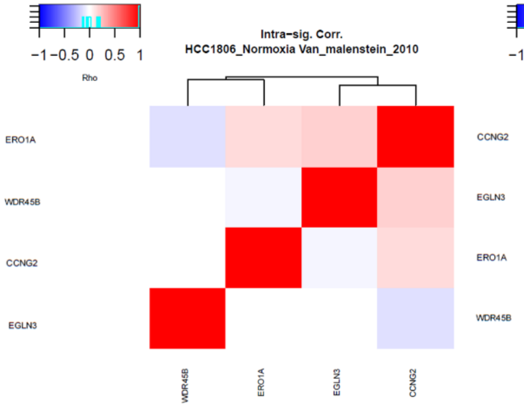

Intra−sig. Corr.
HCC1806_Normoxia Van_malenstein_2010
−1 −0.5 0 0.5 1
Rho
CCNG2
EGLN3
ERO1A
WDR45B
WDR45B
ERO1A
EGLN3
CCNG2

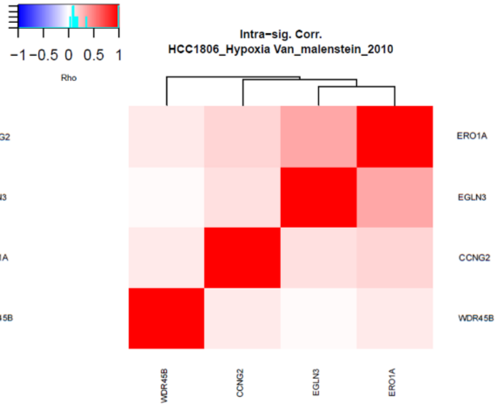

Intra−sig. Corr.
HCC1806_Hypoxia Van_malenstein_2010
−1 −0.5 0 0.5 1
Rho
ERO1A
EGLN3
CCNG2
WDR45B
WDR45B
CCNG2
EGLN3
ERO1A

Supplementary Figure S17: Poor intra-signature correlation is the primary driver of aberrant signature performance in single-cell data.

Heatmaps displaying intra-signature auto-correlation for normoxic and hypoxic subsets from MCF7 and HCC1806 single-cell datasets. Both rows and columns represent van Malestein signature features, while colour intensities indicate correlation levels. The evident weak intra-signature correlation greatly influences the disparity in signature performance between bulk and single-cell datasets, related to Figure 4.

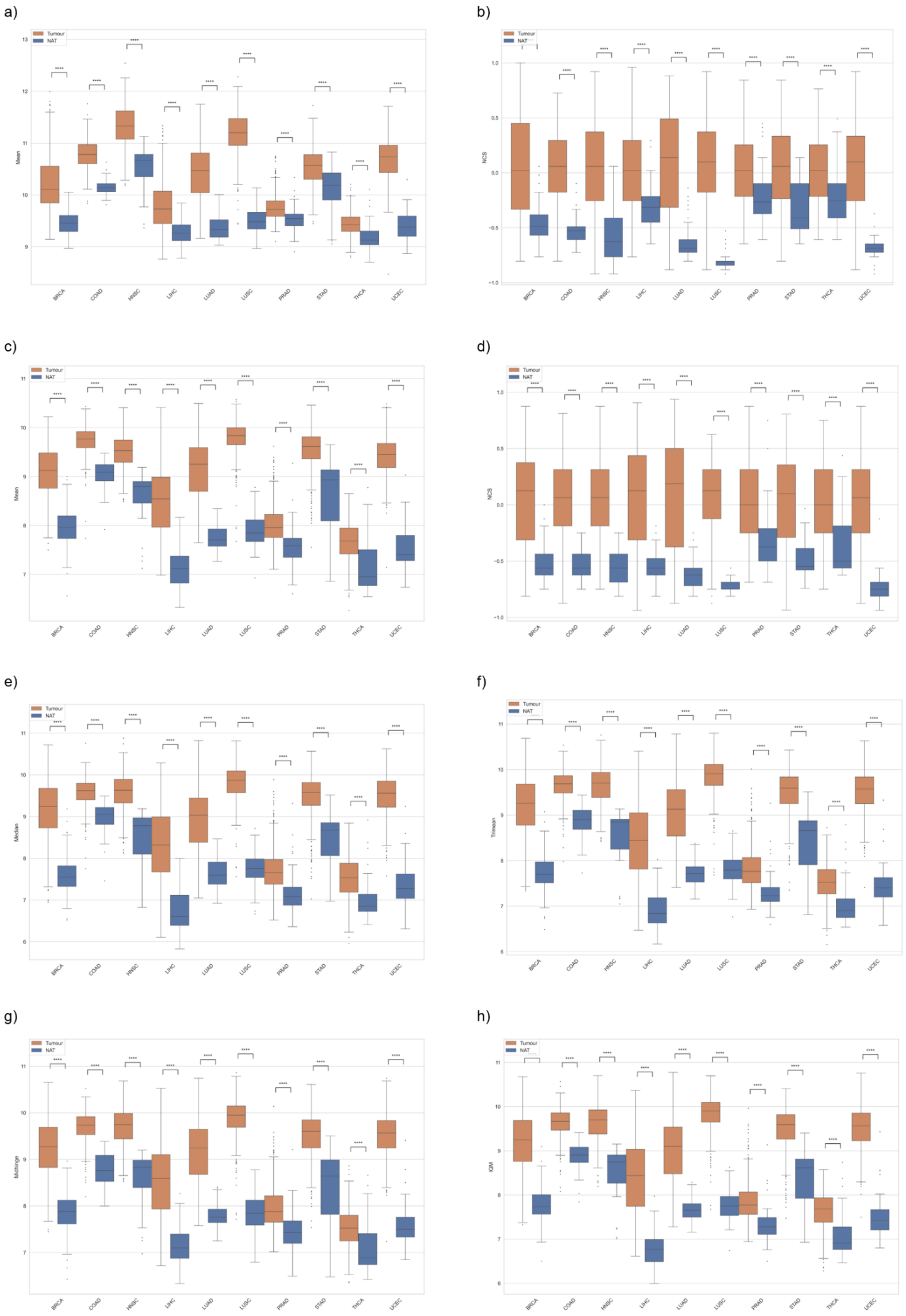
a)
b)
c)
d)
e)
f)
g)
h)
Tumour
NAT
Mean
NCS
Median
Trimean
Midhinge
IQM
BRCA
COAD
HNSC
LIHC
LUAD
LUSC
PRAD
STAD
THCA
UCEC

Supplementary Fig. S18: Two signatures, Buffa and Ragnum, showed higher scores in tumours compared to NAT across all 10 cancer types

Boxplots depicting hypoxia score differences in Buffa and Ragnum hypoxia signatures in 10 tumour types and normal adjacent tissue (NAT) in the The Cancer Genome Atlas (TCGA), related to Figure 5. Two signatures, Buffa and Ragnum, showed higher scores in tumours compared to NAT across all 10 cancer types using six scoring metrics. These results are shown in the eight panels, with orange denoting tumours and blue NAT: a) Buffa mean, b) Buffa NCS, c) Ragnum mean, d) Ragnum NCS, e) Ragnum median, f) Ragnum trimean, g) Ragnum midhinge, h) Ragnum NCS. The p-values were calculated using the Mann-Whitney-Wilcoxon test (****, p <= 1.00E-04).

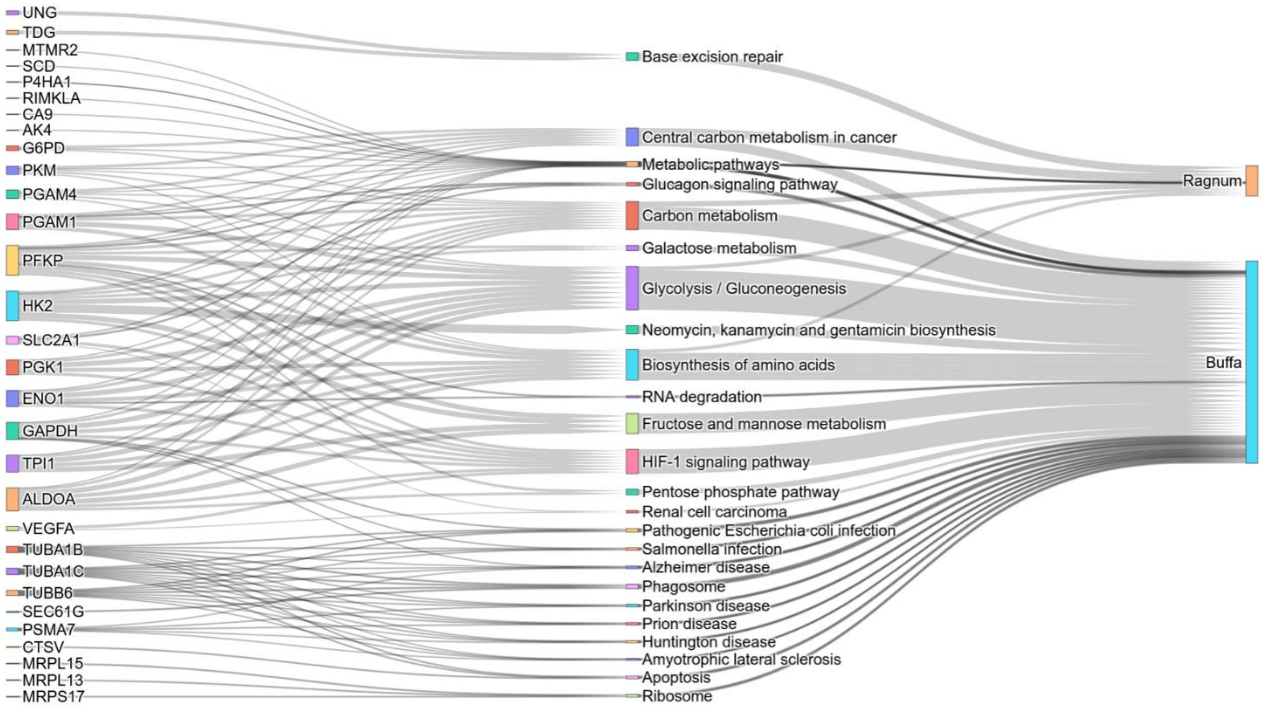

UNG
TDG
MTMR2
SCD
P4HA1
RIMKLA
CA9
AK4
G6PD
PKM
PGAM4
PGAM1
PFKP
HK2
SLC2A1
PGK1
ENO1
GAPDH
TPI1
ALDOA
VEGFA
TUBA1B
TUBA1C
TUBB6
SEC61G
PSMA7
CTSV
MRPL15
MRPL13
MRPS17
Base excision repair
Central carbon metabolism in cancer
Metabolic pathways
Glucagon signaling pathway
Carbon metabolism
Galactose metabolism
Glycolysis / Gluconeogenesis
Neomycin, kanamycin and gentamicin biosynthesis
Biosynthesis of amino acids
RNA degradation
Fructose and mannose metabolism
HIF-1 signaling pathway
Pentose phosphate pathway
Renal cell carcinoma
Pathogenic Escherichia coli infection
Salmonella infection
Alzheimer disease
Phagosome
Parkinson disease
Prion disease
Huntington disease
Amyotrophic lateral sclerosis
Apoptosis
Ribosome
Ragnum
Buffa

Supplementary Fig. S19: Gene-pathway associations in the Buffa and Ragnum hypoxia signatures

The Sankey diagram delineates the relationships between individual genes from Buffa and Ragnum hypoxia signatures and the biological pathways they are involved in. Each gene is linked to one or more pathways, denoting its functional role within the cellular processes. The pathways, in turn, converge into the hypoxia signatures of Buffa or Ragnum, highlighting the contribution of each pathway to the respective signature. The width of the bands reflects the number of genes shared between the entities, emphasising the significance of each gene and pathway to the hypoxia signatures' composition. The diagram reveals a significant enrichment of both signatures in pathways such as glycolysis/gluconeogenesis, central carbon metabolism in cancer, and broader carbon metabolism processes, related to Figure 5.

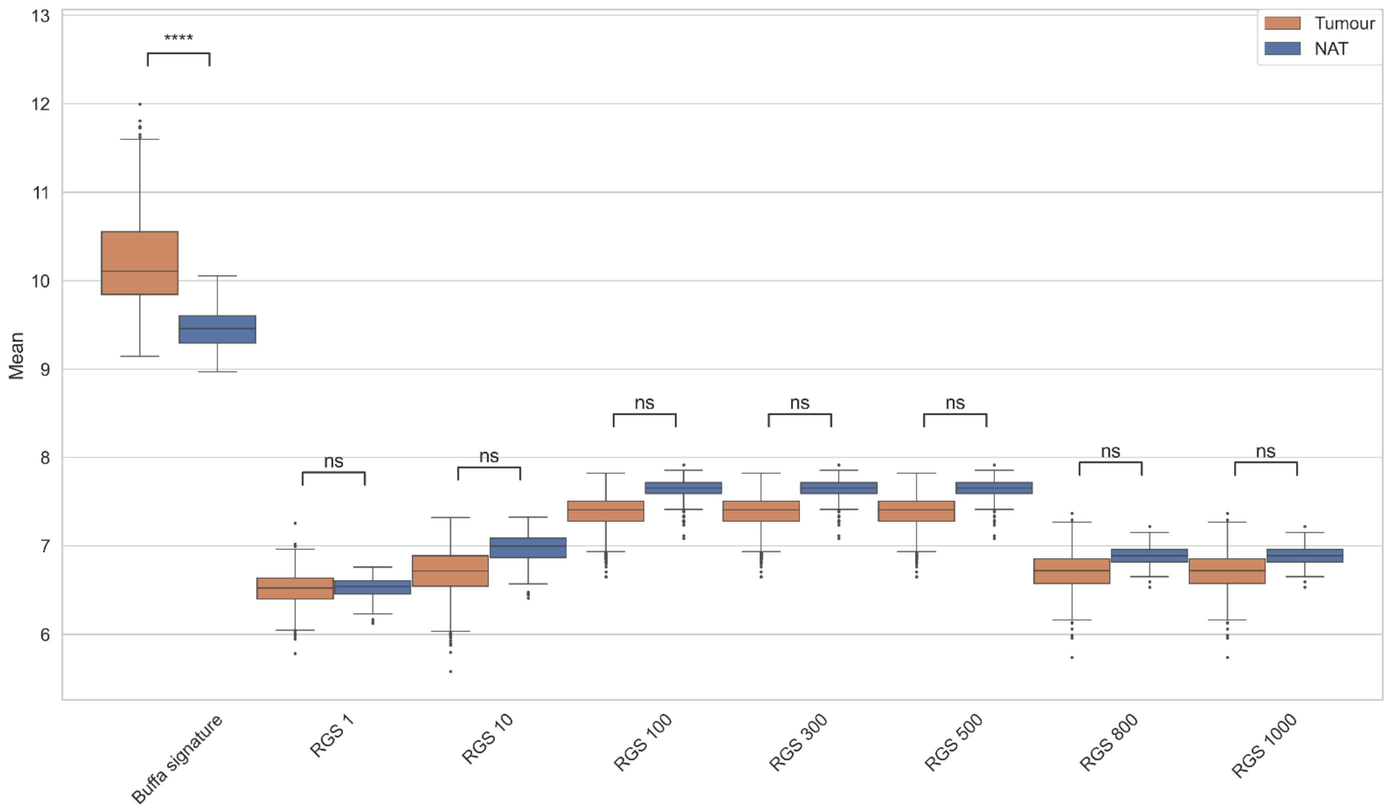

Tumour
NAT
Mean
****
ns
ns
ns
ns
ns
ns
ns
13
12
11
10
9
8
7
6
Buffa signature
RGS 1
RGS 10
RGS 100
RGS 300
RGS 500
RGS 800
RGS 1000

Supplementary Fig. S20: Hypoxia scores differences between hypoxia signatures and random gene sets in clinical samples

Boxplots depict the differences in hypoxia score distributions between Buffa signature and RGS using mean score in the BRCA cohort in TGCA. Hypoxia scores from RGS in tumour samples vs. normal adjacent tissue (NAT) show a lower score and a non-significant p-value as opposed to the original signature. The p-values were calculated using the Mann-Whitney-Wilcoxon test (****, $p <= 1.00E-04$), related to Figure 5.

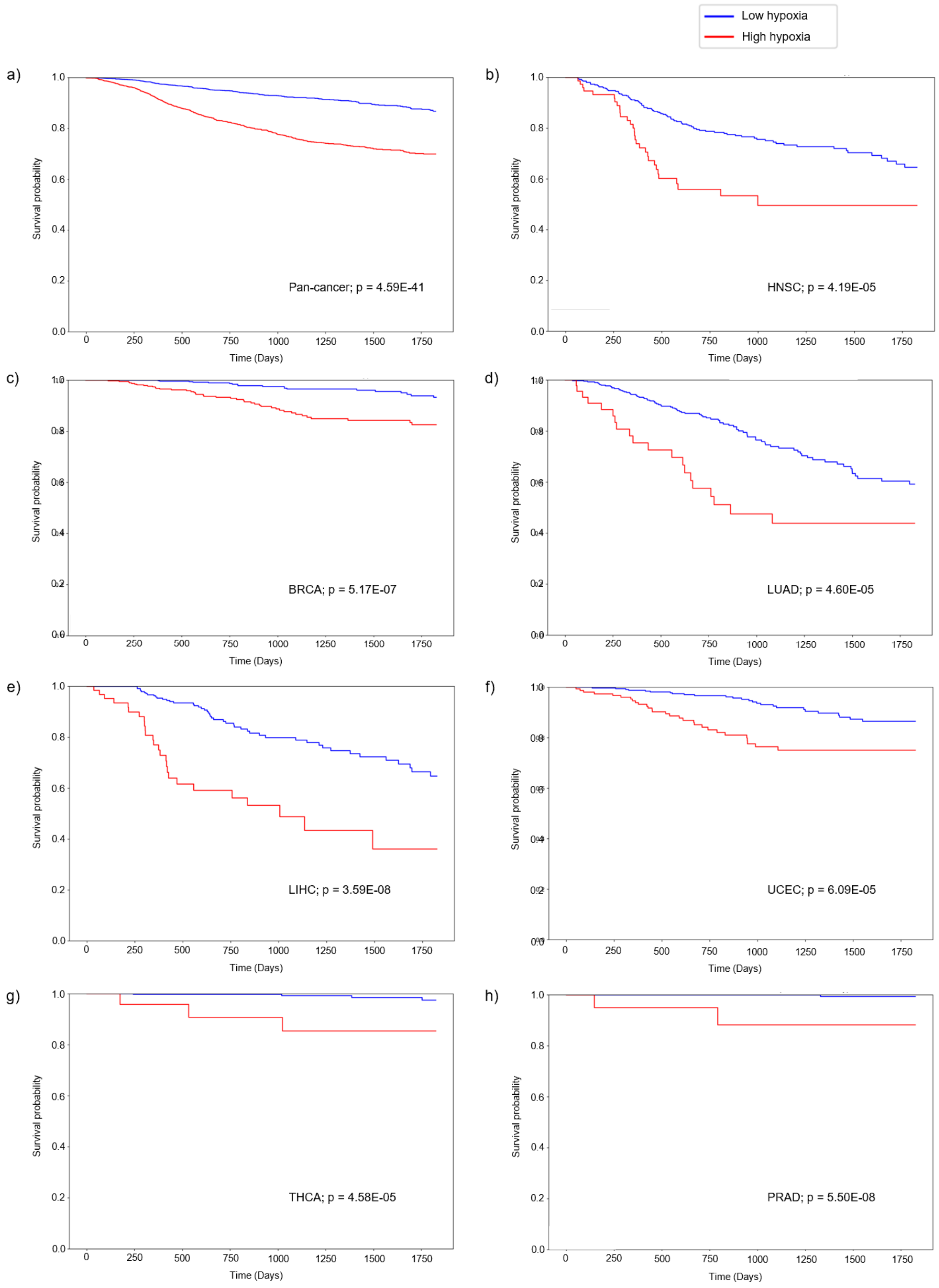
Low hypoxia
High hypoxia
a)
Pan-cancer; p = 4.59E-41
b)
HNSC; p = 4.19E-05
c)
BRCA; p = 5.17E-07
d)
LUAD; p = 4.60E-05
e)
LIHC; p = 3.59E-08
f)
UCEC; p = 6.09E-05
g)
THCA; p = 4.58E-05
h)
PRAD; p = 5.50E-08
Survival probability
Time (Days)
0.0
0.2
0.4
0.6
0.8
1.0
0
250
500
750
1000
1250
1500
1750

Supplementary Fig. S21: Buffa/mean shows prognostic efficacy across the TCGA

Kaplan–Meier survival estimates using the Buffa hypoxia signature and the mean score were investigated across pan-cancer and across ten individual cancer types. A point of contention in the field is what type of thresholds should be used across tumour types to denote low and high hypoxic samples. Previous works have used above and below the median however this is not necessarily the most comprehensive approach. Thus, to be more thorough, the prognostic ability of Buffa/mean was examined every fifth percentile using disease specific survival (DSS). The cohorts are dichotomised into high (red) and low (blue) hypoxia groups according to the percentile that yields the most significant Log-rank p from the Kaplan-Meier survival analysis. Buffa/mean was significantly prognostic in 7/10 tumour types, including (b) HNSC [85th percentile], (c) BRCA [60th percentile], (d) LUAD [90th percentile], (e) LIHC [75th percentile], (f) UCEC [70th percentile], (g) THCA [95th percentile] and (h) PRAD [90th percentile]. Furthermore, Buffa/mean signature maintained its prognostic value in a combined pan-cancer dataset (a) [50th percentile], related to Figure 5.

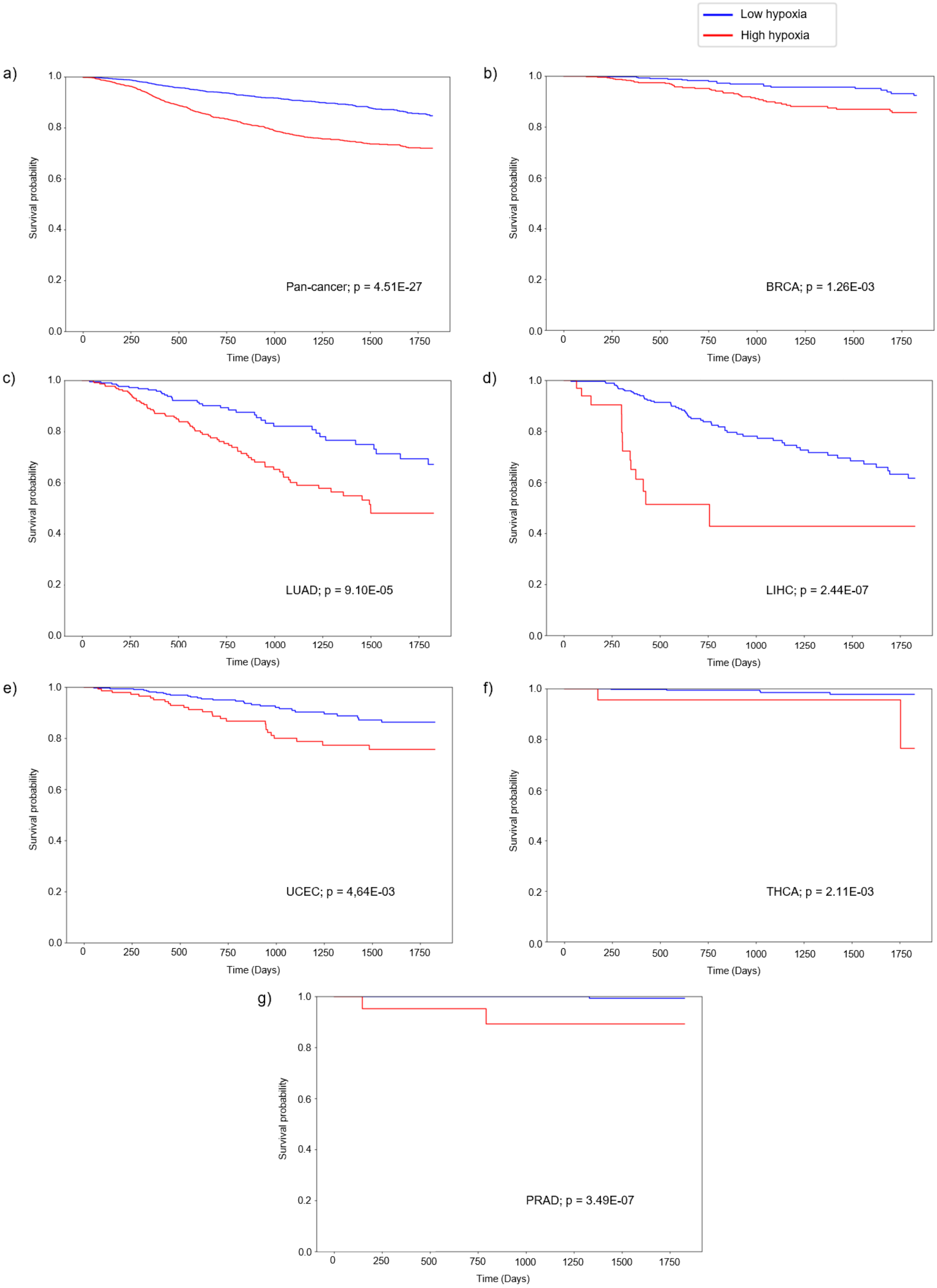
Low hypoxia
High hypoxia
a)
Pan-cancer; p = 4.51E-27
b)
BRCA; p = 1.26E-03
c)
LUAD; p = 9.10E-05
d)
LIHC; p = 2.44E-07
e)
UCEC; p = 4,64E-03
f)
THCA; p = 2.11E-03
g)
PRAD; p = 3.49E-07
Survival probability
Time (Days)
0.0
0.2
0.4
0.6
0.8
1.0
0
250
500
750
1000
1250
1500
1750

Supplementary Fig. S22: Ragnum/IQM shows prognostic efficacy across the TCGA

Kaplan–Meier survival estimates using the Ragnum hypoxia signature and the IQM score were investigated across pan-cancer and across ten individual cancer types across ten cancer types (conventions as in S47). Ragnum/IQM was significantly prognostic in 6/10 tumour types, including (b) BRCA [50th percentile], (c) LUAD [50th percentile], (d) LIHC [90th percentile], (e) UCEC [70th percentile], (f) THCA [50th percentile] and (g) PRAD [95th percentile]. Ragnum/IQM also maintained its prognostic value in a combined pan-cancer dataset (a) [50th percentile], related to Figure 5.

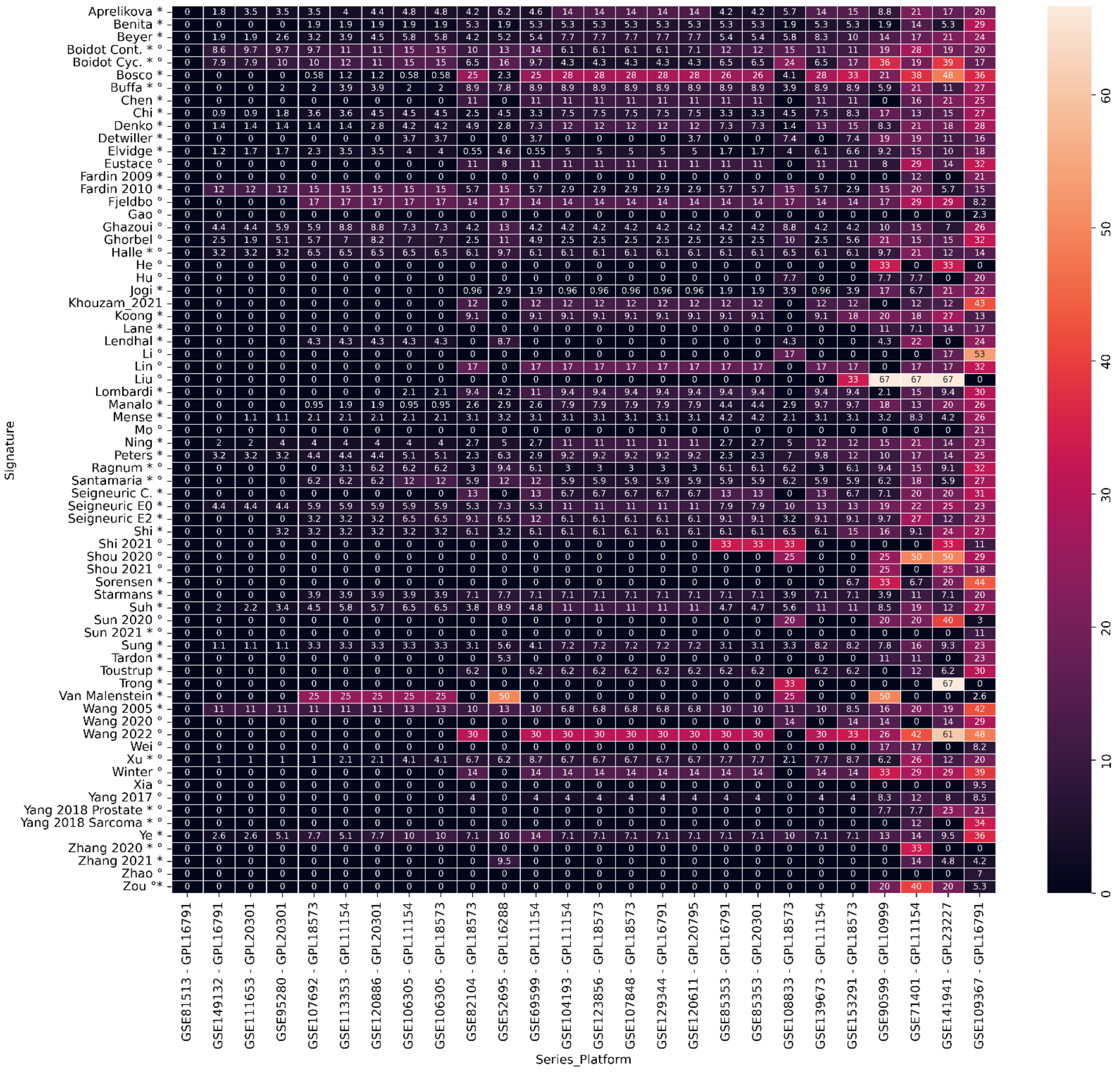
Signature
Aprelikova *
Benita *
Beyer *
Boidot Cont. * °
Boidot Cyc. * °
Bosco *
Buffa * °
Chen *
Chi *
Denko *
Detwiller *
Elvidge *
Eustace °
Fardin 2009 *
Fardin 2010 *
Fjeldbo °
Gao °
Ghazoui °
Ghorbel °
Halle * °
He °
Hu °
Jogi *
Khouzam_2021
Koong *
Lane *
Lendhal *
Li °
Lin °
Liu °
Lombardi *
Manalo *
Mense *
Mo °
Ning *
Peters *
Ragnum * °
Santamaria * °
Seigneuric C. *
Seigneuric E0 *
Seigneuric E2 *
Shi *
Shi 2021 °
Shou 2020 °
Shou 2021 °
Sorensen *
Starmans *
Suh *
Sun 2020 °
Sun 2021 * °
Sung *
Tardon *
Toustrup *
Trong *
Van Malenstein *
Wang 2005 *
Wang 2020 °
Wang 2022 °
Wei °
Xu * °
Winter °
Xia °
Yang 2017 °
Yang 2018 Prostate * °
Yang 2018 Sarcoma * °
Ye *
Zhang 2020 * °
Zhang 2021 *
Zhao °
Zou °*
GSE81513 - GPL16791
GSE149132 - GPL16791
GSE111653 - GPL20301
GSE95280 - GPL20301
GSE107692 - GPL18573
GSE113353 - GPL11154
GSE120886 - GPL20301
GSE106305 - GPL11154
GSE106305 - GPL18573
GSE82104 - GPL18573
GSE52695 - GPL16288
GSE69599 - GPL11154
GSE104193 - GPL11154
GSE123856 - GPL18573
GSE107848 - GPL18573
GSE129344 - GPL16791
GSE120611 - GPL20795
GSE85353 - GPL16791
GSE85353 - GPL20301
GSE108833 - GPL18573
GSE139673 - GPL11154
GSE153291 - GPL18573
GSE90599 - GPL10999
GSE71401 - GPL11154
GSE141941 - GPL23227
GSE109367 - GPL16791
Series_Platform
0
10
20
30
40
50
60

Supplementary Fig. S23: Percentage of genes missing in each signature in hypoxia-related bulk RNASeq experiments

Summary of the percentage of missing genes in each signature for all the bulk RNASeq platforms in GEO hypoxia experiments. Signature names are displayed on the y-axis, while GEO Platform (GPL) and Series (GSE) IDs are located on the x-axis. Lighter square colours indicate a higher percentage of missing genes for the respective signature, platform, and series. Individual missing-gene percentages for each signature and series are provided in the corresponding box, related to Table 1.

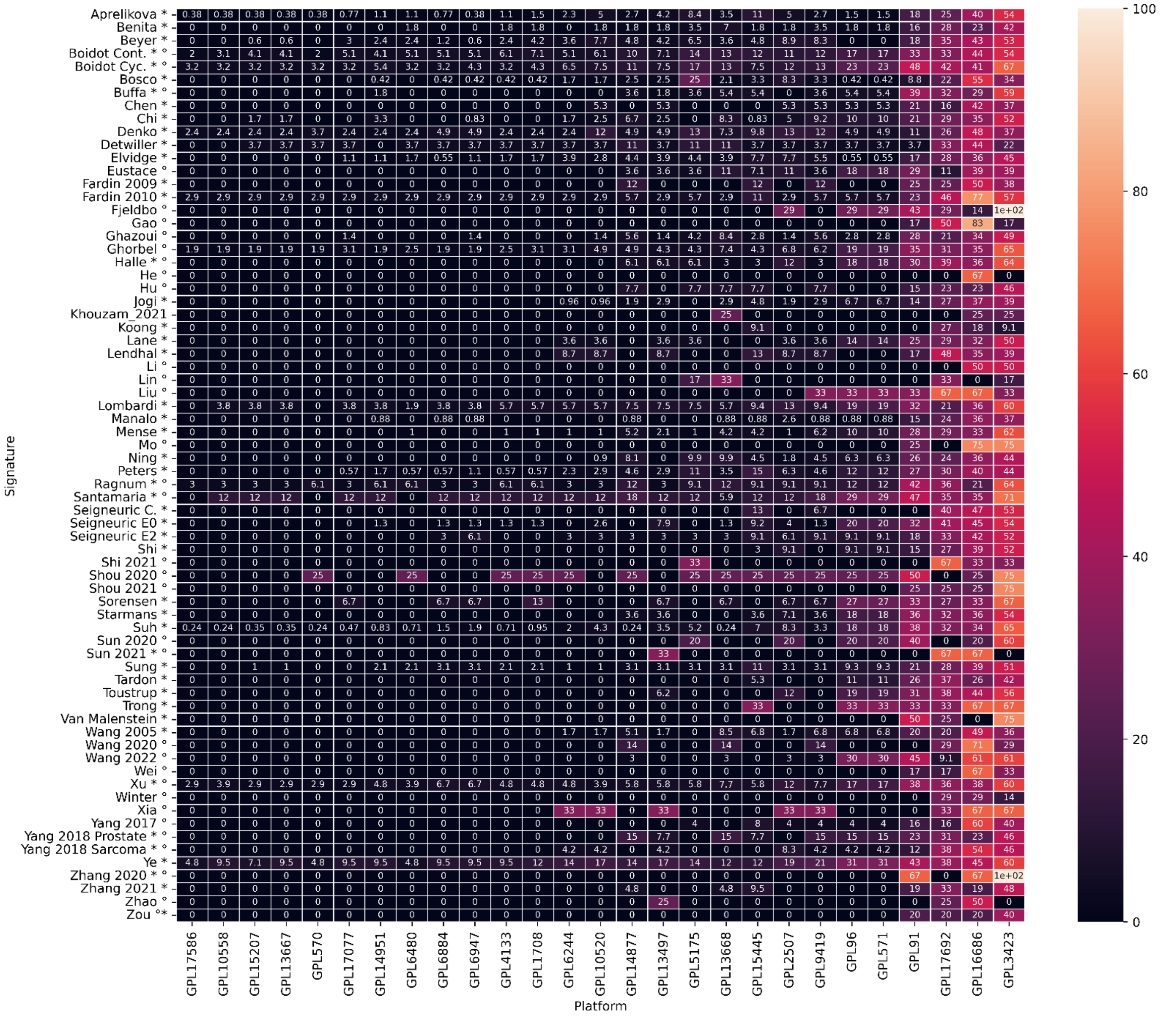
Signature
Aprelikova *
Benita *
Beyer *
Boidot Cont. * °
Boidot Cyc. * °
Bosco *
Buffa * °
Chen *
Chi *
Denko *
Detwiller *
Elvidge *
Eustace °
Fardin 2009 *
Fardin 2010 *
Fjeldbo °
Gao °
Ghazoui °
Ghorbel °
Halle * °
He °
Hu °
Jogi *
Khouzam_2021
Koong *
Lane *
Lendhal *
Li °
Lin °
Liu °
Lombardi *
Manalo *
Mense *
Mo °
Ning *
Peters *
Ragnum * °
Santamaria * °
Seigneuric C. *
Seigneuric E0 *
Seigneuric E2 *
Shi *
Shi 2021 °
Shou 2020 °
Shou 2021 °
Sorensen *
Starmans *
Suh *
Sun 2020 °
Sun 2021 * °
Sung *
Tardon *
Toustrup *
Trong *
Van Malenstein *
Wang 2005 *
Wang 2020 °
Wang 2022 °
Wei °
Xu * °
Winter °
Xia °
Yang 2017 °
Yang 2018 Prostate * °
Yang 2018 Sarcoma * °
Ye *
Zhang 2020 * °
Zhang 2021 *
Zhao °
Zou °*
GPL17586
GPL10558
GPL15207
GPL13667
GPL570
GPL17077
GPL14951
GPL6480
GPL6884
GPL6947
GPL4133
GPL1708
GPL6244
GPL10520
GPL14877
GPL13497
GPL5175
GPL13668
GPL15445
GPL2507
GPL9419
GPL96
GPL571
GPL91
GPL17692
GPL16686
GPL3423
Platform
100
80
60
40
20
0

Supplementary Fig. S24: Percentage of genes missing in each signature in hypoxia-related microarray experiments

Summary of percentage missing genes in each signature for all the microarray platforms in GEO hypoxia experiments. Signature names are displayed on the y-axis, while GEO Platform (GPL) IDs are located on the x-axis. Since microarray data for each platform share the same manifest, Series (GSE) IDs are not reported. Lighter square colours indicate a higher percentage of missing genes for the respective signature and platform. Individual missing-gene percentages for each signature and series are provided in the corresponding box, related to Table 1.

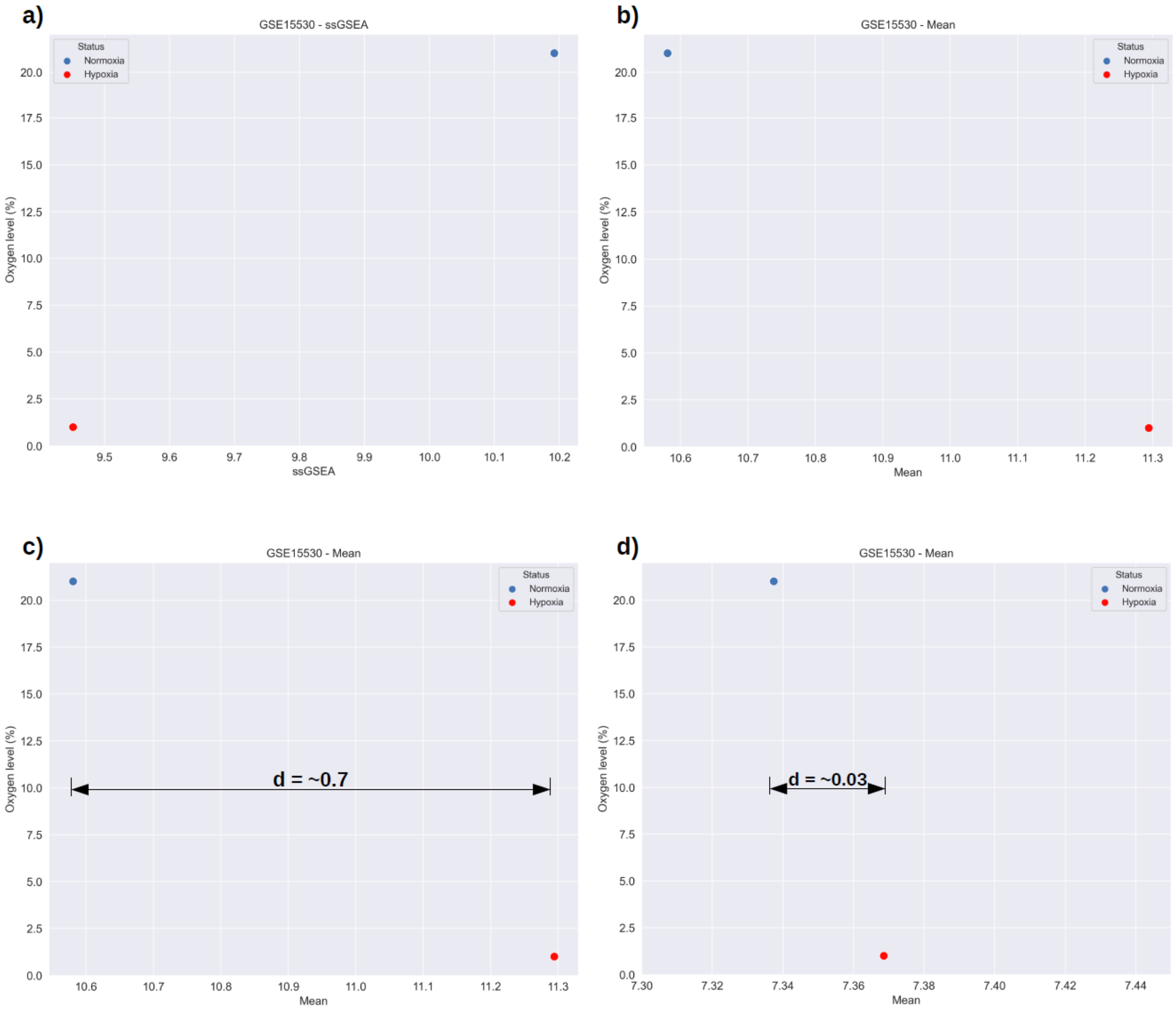

a)
GSE15530 - ssGSEA
Status
Normoxia
Hypoxia
Oxygen level (%)
ssGSEA
b)
GSE15530 - Mean
Mean
c)
GSE15530 - Mean
d = ~0.7
d)
GSE15530 - Mean
d = ~0.03

Supplementary Fig. S25: Hypoxia scores and distance calculation against RGS

The scatterplots were generated using Buffa signature on one normoxic (GSM390200, highlighted in blue) and one hypoxic (GSM390196, highlighted in red) sample from the Series GSE15530. In *a)* the mean and b) the ssGSEA scores are plotted on the x-axis against the percentage of oxygen. The mean score of the hypoxic sample is greater than the score of the normoxic sample. Viceversa, the ssGSEA score of the hypoxic sample is lower than the score of the normoxic sample, resulting in a wrong classification. Panel *c)* shows that the distance $d_{SIG}(hyp, norm)$ calculated using the mean score between the two samples is ~24x higher (left image, $d_{SIG} = \sim 0.7$) than the distance observed between the same samples using a RGS of the same length (Panel d, $d_{RGS} = \sim 0.03$), related to Figure 2.
